# Understanding the aliya pulsed electric field dose-response relationship: Implications for ablation size, thermal load, and immune response in an orthotopic murine breast cancer model

**DOI:** 10.1371/journal.pone.0318440

**Published:** 2025-02-13

**Authors:** Ebtesam H. O. Nafie, Chiara Pastori, Rupsa Acharya, Rosa Kaviani, David Hunter, Waleed Shalaby, William S. Krimsky, Robert E. Neal

**Affiliations:** Galvanize Therapeutics, Redwood City, CA, United States of America; Sichuan University, CHINA

## Abstract

Aliya^®^Pulsed Electric Field (PEF) technology is an emerging strategy in the field of cancer treatment, offering a novel approach to ablation therapy that does not rely on thermal mechanisms. By employing a multi-stage experimental setup, including potato tuber, porcine liver, and murine breast cancer models, we explored the dose-response relationship on ablation and immune modulation by varying the pulse packets delivered from 20 to 100. The biologic response observed with 60 packets represented a minimum effective dose yielding reproducible ablation parameters, immune response, and efficacy which could be augmented with immune checkpoint blockade. This pre-clinical analysis provides a first step toward understanding the therapeutic index for PEF technology beyond ablation, a consideration that will require robust clinical validation in well-designed prospective studies.

## Introduction

Modern treatments in solid tumor oncology have evolved to incorporate rational dose-response relationships such as dose-dense chemotherapy, hypofractionated radiotherapy (HFRT), and stereotactic body radiation therapy (SBRT) to improve patient tolerability while maintaining efficacy [1–3]. Many of these strategies were originally proposed in animal models and later validated through prospective clinical investigation. Like radiotherapy, a number of thermal ablative modalities have been studied with the hope of stimulating systemic immune-mediated or abscopal responses beyond focal tumor ablation [4], where the emphasis has shifted towards potential synergies with systemic treatments. It can be argued that ablation is not a binary event and may reflect dose-dependent parameters. In combination with systemic therapies, complex interdependencies may arise within the tumor microenvironment that rely on the mechanism of cell death and injury response.

A promising avenue in the evolution of ablation is the Aliya^®^Pulsed Electric Field (PEF) technology. Unlike traditional modalities, PEF operates by delivering electrical pulses that alter cellular transmembrane potentials, subsequently instigating a myriad of cell death processes without reliance on thermal mechanisms [5]. This approach mitigates local temperature increases to minimize extracellular protein denaturation from coagulation necrosis and improves retention of the extracellular matrix, enhancing its safety profile near sensitive structures.

Beyond local ablation, evidence is emerging that ablation by PEF technology can cause downstream immune-modulation, producing systemic anti-tumor responses, where extracellular protein retention which may be critical to immune cell infiltration and remodeling [6]. Preclinical evidence demonstrated this effect to be superior to thermal ablation [7] and its ability to augment outcomes from systemic therapies [6]. While promising, understanding the PEF dose-response relationship is a critical first step toward defining therapeutic indices for PEF. Here, we explore a basic waveform parameter, the number of PEF pulse packets delivered to determine its influence on ablation effects and immune modulation as a function of biologic response.

## Materials and methods

### Study schema

**Fig 1:**
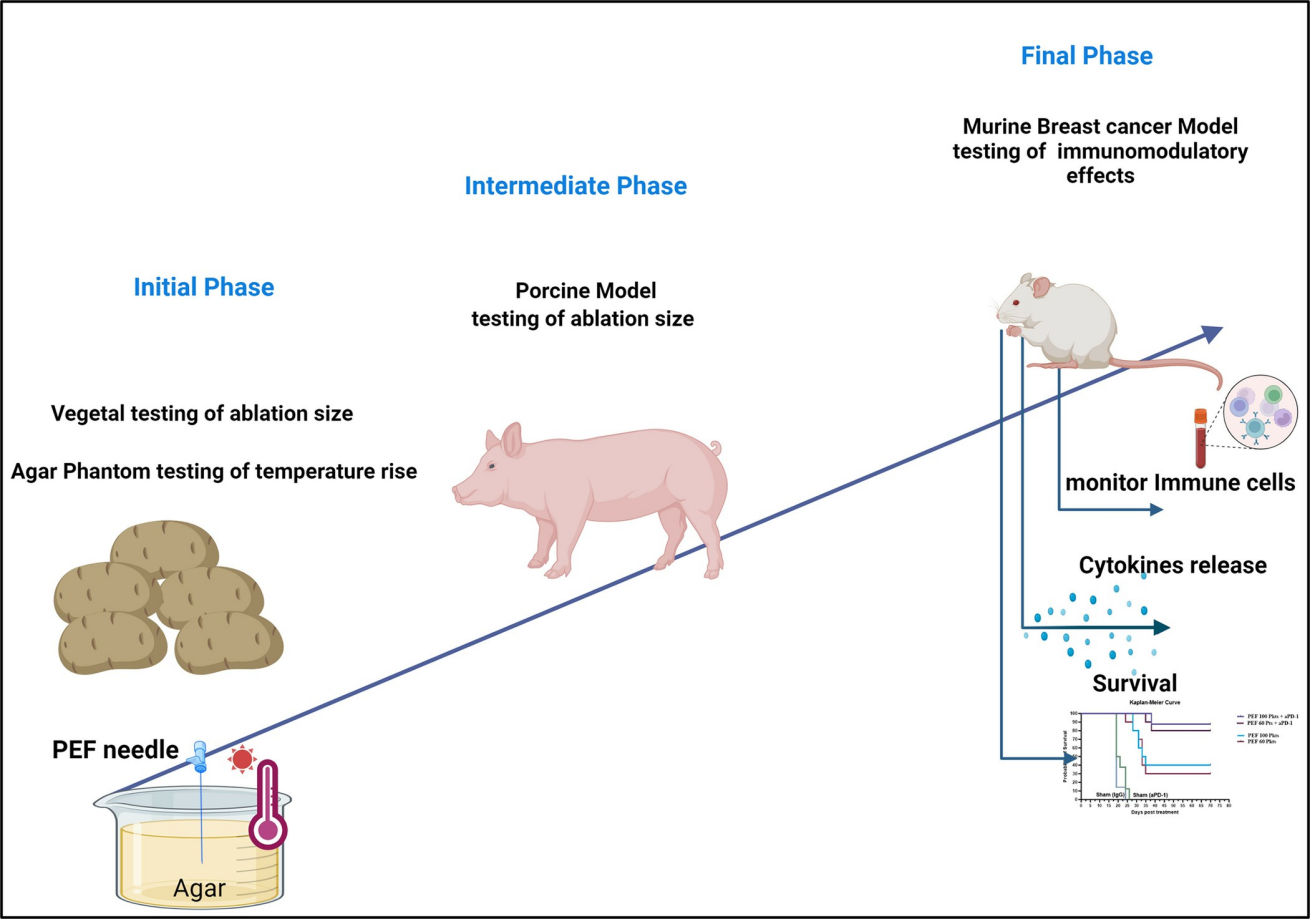
Study schema. Schematic representation of the experimental workflow for testing PEF (Pulsed Electric Fields) treatment across different models: 1. Tuber & Agar Model: Initial phase involving the testing of different PEF packets configurations, effect sizes, and temperature effects using a tuber-based and agar model. 2. Porcine Model: Intermediate phase focusing on determining the ablation size for 60 and 100 PEF packets in a porcine model to bridge the gap between vegetal and murine models. 3. Murine Breast Cancer Model: Final phase assessing the immunomodulatory effects of 60 and 100 PEF packets in a murine breast cancer model. This phase includes monitoring immune cell activity, cytokine release, and survival, depicted by a Kaplan-Meier survival curve.

A series of experiments were undertaken to assess the relative contribution of PEF packets dose to both ablation size and biologic response (Fig 1). This schema of experiments targeted evaluating the interrelationship between ablation size, thermal load, and implications on the immune response. The initial step used Vegetal (Potato tuber) models to determine the effect size as a function of packets delivered and select which packet counts should be evaluated to represent a minimum effective dose and a maximum general dose. Next, a limited set of ablation experiments were performed in porcine liver to compare selected packets doses with the tuber model. Finally, the immune response with the same selected packet doses were studied in a murine orthotopic breast cancer model after partial ablation of a primary tumor in the presence of secondary disease, including outcomes and interactions with and without immune checkpoint blockade.

#### 1.Tuber model

The vegetal tuber model provides a cost-effective, high-throughput approach to preliminarily examine effect size relative to packet count where the changes closely approximate animal models [8,9]. Using a PEF waveform comparable to the Aliya system for clinical use, PEFs were delivered with voltage adjusted to best evaluate effect changes in response to packet number. A tank of 38 x 10 cm was filled with 3.6 L of 0.36% NaCl solution. Russet potatoes (> 5 cm diameter) were sourced from a local grocer. The top and bottoms were trimmed and a 1 mm diameter needle with a 1 cm exposed electric surface was inserted perpendicularly into the top until reaching a tip-depth of 3.0 cm. This provided ≥ 2 cm distance from the needle to any surface of the potato. The potato was fully submerged at the far end of the tank while a custom circular 10 cm diameter electrode was submerged at the opposing end to serve as the electric ground. Both the active needle and ground electrode were connected to a generator that delivered a representative PEF waveform at a voltage shown in pilot studies to evoke an effect diameter in potato of approximately 17 mm for 100 packets. This size was deemed large enough to detect differences without incurring significant boundary effects (>0.5cm margin of normal tuber). The same waveform and voltage were used to deliver 20, 40, 60, and 80 to compare with the 100 packets, respectively (all n = 5).

After PEF delivery, the needle and potatoes were removed, and the potato was left to rest in air for at least 60 min. Potatoes were then sliced into 3.1 mm thick slices perpendicular to the direction of needle insertion and submerged into 0.9% NaCl with 0.5% blue food dye solution for staining to improve visualization, as described in [10]. After at least 30 min, slices were removed, rinsed with 0.9% NaCl solution, and photographed with a ruler to analyze short and long diameter treatment effect using ImageJ (NIH, USA). Only short diameter measurements were used for comparison to eliminate any bias from off-axis potato slicing. To minimize variability between model condition, all testing was performed in a single day with a single potato supply.

After measuring the affected zone across all packets, it was found that 60 packets produced an equivalent effect size to 80 and 100 packets. Thus, a 60 packet dose was identified as the minimum effective dose for additional analyses in comparison with 100 packets.

#### 2. Porcine ablation

A set of porcine liver ablations were performed to compare the ablation diameters with that observed from the tuber model. The methods used are similar to those described in [11] and described briefly here. All animal experiments were performed in accordance with the animal facility’s Institutional Animal Care and Use Committee (IACUC) under protocol number IAC-0015 at Bayside Preclinical Services, Inc. Efforts were made to minimize animal suffering, including the use of anesthetics and analgesics where appropriate. Humane endpoints were predefined and implemented to ensure animals were euthanized when they displayed early markers associated with death or poor prognosis of quality of life, or specific signs of severe suffering or distress. Induction anesthesia involved intramuscular Telazol (4 mg/kg) and Xylazine (2 mg/kg), and atropine (0.02 mg/kg), followed by Isoflurane (0–5%) gas anesthesia as needed. Neuromuscular paralytic was not required for any of the PEF ablation studies.

A laparotomy was performed to visualize the liver followed by direct insertion of a 19 Ga needle with a 1 cm electrically exposed tip. The needle was inserted to a depth of approximately 5 cm in a region at least 4 cm thick to prevent boundary effects on treatment size. A custom-built generator was then used to deliver PEFs that represent those delivered by the Aliya System. Based on results from the tuber testing, either 100 packets (n = 11) or 60 packets (n = 5) doses of PEF were delivered to the liver, respectively. Following ablation delivery, the needle was removed, and the ablation site was marked with electrocautery. The laparotomy was then closed with suture and the pigs were then recovered for three days.

On post-PEF day 3, the animals received intravenous 5% 2,3,5-triphenyltetrazolium chloride (TTC) solution for approximately 10 minutes prior to euthanasia to improve ablation lesion visualization. Animals were euthanized using a Euthasol euthanasia solution at a dosage of approximately 1 mL/4 kg given intravenously. Death was confirmed by auscultation prior to necropsy. The livers were excised, and a necropsy was performed. Individual ablation sites were isolated and sliced into approximately 2 mm thick sections perpendicular to the direction of needle insertion. TTC staining is used as a redox indicator to differentiate between metabolically active and inactive tissues, grossly staining metabolically active cells and tissue regions red. Thus, the ablation zone was identified as the region absent of TTC staining. The slice of the largest ablation zone was measured for short and long-axis measurements, where only short diameters were used for comparison to eliminate any bias from off-axis slicing.

#### 3. Temperature effects

PEF delivery results in a small rise in temperature due to Joule heating from electric current passing through a resistive medium. The extent of temperature rise is a function of the delivered electric current, duration of each packet, number of packets, and packets delivery rate. To determine whether the different packet counts may influence ablation or alter the favorable safety profile afforded to PEF by exceeding temperatures implicated in thermal coagulative necrosis to the extracellular matrix protein denaturation, which occurs rapidly for tissues once reaching the 70–80°C range [12,13], the influence of packet count on temperature rise was evaluated using agar phantoms.

Agar phantoms were formulated with 1% agar (w/v) and 0.24% (w/v) with deionized water, producing a phantom with approximately 0.5 S/m electrical conductivity. Individual aluminum foil strips were placed into the tank prior to agar introduction, providing multiple dispersive electrodes. A 1 mm diameter needle with 1 cm exposed electric surface was inserted into the phantom to a tip-depth of 2 cm, at a distance of approximately 15 cm from the return electrode. A fiberoptic thermal probe (STB PROBE with m920 fluoroptic thermometer (Advanced Energy, CO, USA) was inserted to matching depth using a guide that maintained a distance of 2.5 mm from the needle.

Based on the prior data, a total of 60 or 100 packets were delivered with a 2 second delay between packets to represent a worst-case temperature rise relative to the 3 second delay used in the commercial PEF system. Following packet delivery, the temperature continued to be recorded for 3 min until reaching nearly baseline before moving to a naïve agar site to perform the next trial.

A valuable tool for comparing thermal effects beyond absolute temperature can be performed by calculating the equivalent time (in seconds) that matches the amount of thermal effects the tissue would experience at 43°C, a value known as t_43_ [8]. This value is calculated according to the formula:

$$t_{43}=\overset{T_{\mathrm{final}}}{\underset{T_{0}}{\sum }}R^{\left(43-t_{t}\right)}\Delta t$$ (1)

where T_0_ and T_final_ are the initial and final temperatures (in °C) between the given time step, Δt. R is a constant that equals 0.25 when T ≤ 43°C and 0.5 when T > 43°C. This value was calculated relative to all packet count experiments, with temperatures initially normalized to a baseline of 37°C. To include the contributions to t_43_ of the temperature decay back to baseline, a temperature normalization curve was fit as a function of maximum temperature rise for the 100 packets trials. Because of the passive thermal properties of the agar model used, this function was added to the temperature profiles at the time corresponding to delivery of the 60 packets.

#### 4. Immune response in breast cancer murine model

**Fig 2:**
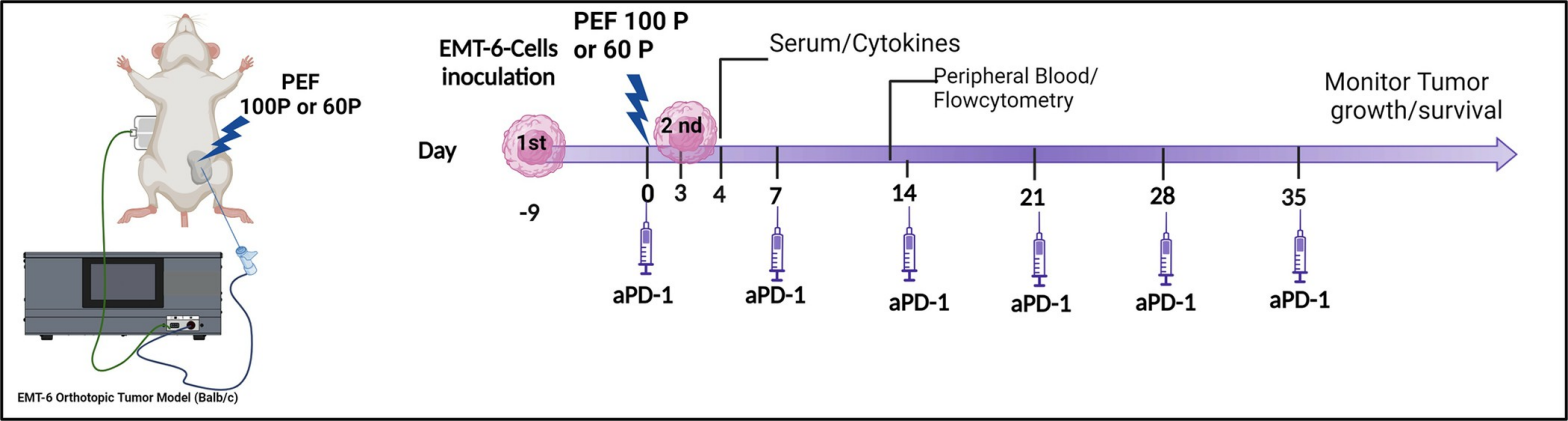
PEF immune study design. EMT6 cells were inoculated into the 4th mammary pad of Balb/c mice. On day 0, a monopolar 25G needle delivered a single PEF treatment, connected to a modified Galvanize Aliya generator with a grounding pad. Treatment groups (1–4) received Aliya PEF parameters with 100 or 60 packets. Groups 2 and 4 were injected intraperitoneally with aPD-1 once per week for a total of 6 weeks. Group 5 and 6 were injected with IgG and aPD-1, respectively. This was followed by inoculation with 2nd EMT6 on day 3 for abscopal effect monitoring. On day 4, serum was collected for 34 cytokine measurements, and on day 13, blood was collected for systemic immune cell analysis.

*4*.*1 Study Design and Treatment Cohorts*. The study design for characterizing the immune response as a function of packets count is provided in (Fig 2). Based on early optimization studies (unpublished) that evaluated immune response characteristics at 1, 3, 4, 7, and 10-days post-PEF, timepoints of 4 days post-PEF for evaluating systemic cytokines and 14-days for evaluating systemic adaptive immune response changes. Immunostimulatory cytokines, cell populations, and tumor growth previously studied for Aliya PEF [6] also used these timepoints, enabling consistency across studies.

All animal studies were performed in accordance with the protocols and animal care guidelines approved by the IACUC, protocol number 2023-05-01. The studies were carried out by an independent contract research organization (CRO), Bayside Biosciences, Inc., located in Santa Clara, CA. EMT6 cells, a triple negative breast cancer cell line (Triple Negative American Type Culture Collection; CRL-2536; Manassas, VA, USA), were cultured in RPMI 1640 medium (MT10040CV, Corning, Manassas, VA, USA) containing 10% fetal bovine serum (092910154, MP Biomedicals, Irvine, CA, USA) and 1% Antibiotic-Antimycotic (15240–062, Gibco Life Technologies Corporation, Grand Island, NY, USA) at 37°C and 5% CO2. A total of 200,000 EMT-6 cells (resuspended in PBS) were inoculated into the mammary fat pad of 4-to 6-week-old BALB/c mice (Charles River Laboratories, Boson, MA) to promote the formation of an orthotopic tumor in the mammary glands.

Once the tumors reached 5–7 mm in size 10 days, mice were randomly assigned to the experimental groups and interventions began (Day 0) (Table 1 and Fig 2). On day 0, orthotopically implanted tumors received either 60 or 100 packet doses of PEF with no other difference between PEF protocols. These two doses were selected based on the equivalent ablation characteristics observed from the potato and porcine experiments. The Aliya PEF was delivered with a waveform intensity titrated to target approximately 80% of the tumor volume to enable determination of the local role of the immune system in tumor clearance, mimicking the study designs of previous preclinical immune investigations [7].

Three days after PEF delivery, a second inoculation of EMT6 cells was performed in the contralateral mammary fat pad to evaluate off-target responses. Lastly, selected groups received weekly intraperitoneal injections of mouse aPD-1or an isotype-control (IgG) from day 0 through day 35 to investigate the influence of immune checkpoint blockade in this model as function of packet dose (Fig 2). Monoclonal antibody αPD-1 (Groups 2, 4, and 6) or isotype-control IgG (Groups 1, 3, and 5) (BioXCell, BE0146 and BE0089, Lebanon, NH, USA) was administered intraperitoneally (i.p.) at 200 μg per injection (100μl volume of 2μg/μl diluted in PBS) once per week for 6 weeks. Blood was drawn on days 4 and 13 for cytokine and flow cytometry analyses, respectively. Tumor growth and survival were then monitored until the end of the study on day 70.

Immunostimulatory markers included systemic cytokines, systemic immune cell populations, tumor growth curves, and overall mouse survival, all of which have been previously described for Aliya PEF in preclinical models [6].

**Table 1:** Study group for breast cancer murine model experiment.

Group #	packets Count	aPD-1	N
**1**	**100**	**No**	**10**
**2**	**100**	**Yes**	**10**
**3**	**60**	**No**	**10**
**4**	**60**	**Yes**	**10**
**5**	**Sham**	**Sham (IgG)**	**7**
**6**	**Sham**	**Yes**	**8**

*4*.*2 Tumor Growth and Mouse Survival*. Tumor growth and survival were then monitored until the end of the study on day 70. Tumors were monitored and measured with electronic calipers three times per week, and tumor volumes were calculated according to the following formula: Tumor Volume = (long dimension x short dimension) x (short dimension/2).

Humane endpoints were established to minimize suffering, including criteria for euthanasia such as maximal tumor volume greater than 1500 mm^3^, excessive metastatic tumor burden, significant weight loss (> 15%), and signs of lethargy. Mice were euthanized using methods approved by the (IACUC: protocol number 2023-05-01), ensuring rapid and humane sacrifice.

*4*.*3 Experimental Groups Controls*. To minimize bias, data were managed by third parties and blinded when possible. Mice were randomly assigned to treatment groups by cage number. An independent contract research organization (CRO) (Bayside Biosciences, Inc., Santa Clara, CA) managed mouse husbandry, performed tumor inoculations and tumor measurements, managed euthanasia decisions, administered αPD-1 & IgG, and collected all biological samples (blood and serum) for analyses. Cytokine analysis was performed by a CRO blinded to mouse ablation conditions. Animal welfare was prioritized throughout the study. The CRO ensured that all procedures adhered to humane endpoints and employed methods to alleviate suffering, including the use of anesthetics and analgesics as needed.

### Cytokines Analysis

A panel of 32 cytokines were selected to assess injury, including T-helper type 1 (Th1) and T-helper type 2 (Th2) immune responses after PEF delivery (Eve Technologies, Alberta, Canada). Th1 responses are typically involved in the activation of macrophages, mediate antimicrobial defense, tissue destruction, and antitumor resistance [14], while Th2 responses are crucial in promoting antibody production, immunoregulation and resolution of inflammation, exhibit tissue remodeling and repair functions, promote wound healing [15]. Serum samples were collected on day 4 and analyzed for the injury and pro-inflammatory (VEGF, Eotaxin, G-CSF, GM-CSF, M-CSF, IL-1α, IL-1β, KC, LIF, LIX, MCP-1, MIP-1α, MIP-1β, MIP-2, LIF, LIX, RANTES); Th-1(IL-2, IL-10, IFN- γ, TNF-α, IP-10, MIG, IL-12p40, IL-12p70) and Th-2 (IL-3, IL-4, IL-5, IL-6, IL-10, IL-13, IL-17).

### Flow Cytometry to Characterize Immune Cell Populations

Flow cytometry was performed using a CytoFLEX3 flow cytometer (Beckman Coulter, CA, USA) on blood samples collected at +13 days from PEF treatment to characterize innate and adaptive immune response S1 Table. Cell populations included CD45+ lymphocytes, B-cells, Natural Killer (NK) cells, CD3+ T cells as well as CD4 and CD8 T cells with mature subtypes. Raw data were analyzed with Kaluza (Beckman Coulter).

### Statistical Analysis

All statistical analyses were performed using Prism software (GraphPad, CA, USA). Differences between means of two unpaired samples were analyzed using Student’s t-test. Cytokine analyses were conducted using multiple comparison t-tests, with q-values replacing traditional p-values to control the false discovery rate (FDR). This adjustment enhances the reliability of our results by reducing the risk of false positives in multiple testing. Flow cytometry data were analyzed using one-way ANOVA. Survival curve significance was determined using the Kaplan-Meier method and the Log-rank test. Tumor volume comparisons between two groups were performed using Student’s t-test. Statistical significance was set at p < 0.05.

## Results

### Ablation size evaluation

#### Tuber model

**Fig 3:**
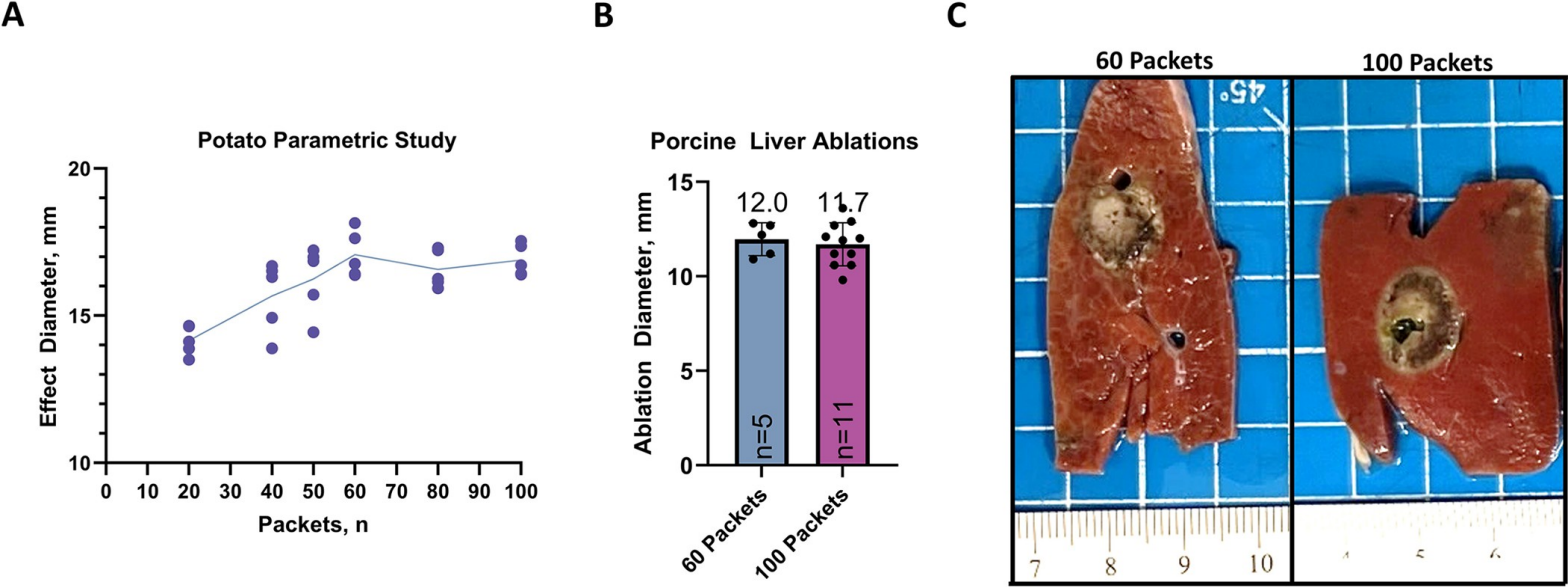
Effect size and gross pathology. A) Potato Parametric Study: The graph shows the relationship between the number of packets (n) and the effect size in a potato model. The trend line indicates an initial increase in diameter with more packets, followed by a plateau. B) Porcine Liver Ablations: Bar graph comparing ablation diameters in porcine liver tissue using 60 and 100 packets. The average ablation diameters are 12.0 mm for 60 packets (n = 5) and 11.7 mm for 100 packets (n = 11). C) Gross Pathology of Porcine Liver Ablations: Images of ablated porcine liver tissue slices show similar appearance between ablations performed with 60 packets (left) and 100 packets (right).

Affected zone size from the tuber parametric study on packets count can be found in (Fig 3A). The effect diameter increased incrementally from 20 to 60 packets. Between 60 and 100 packets, there were no statistically significant differences observed relative to effect diameter. Based on these findings, the 60 packets count was selected as the minimally effective PEF dose for validation in the porcine liver model.

#### Porcine liver

All porcine liver ablations were performed without incident with all animals surviving until the day 3 euthanasia timepoint. After euthanasia, all delivered ablations were identified relative to their coded ablation number. The mean ablation diameters for the 60 and 100 packets counts were 12.0 ± 0.9 mm and 11.7± 1.1 mm, respectively (Fig 3B). Gross inspection of representative liver specimens appeared to show comparable injury characteristics between 60 and 100 packets (Fig 3C). Therefore, the 60 packets appear equivalent to the 100 packet dose in terms of ablation size and gross pathological characteristics.

#### Temperature rise

**Fig 4:**
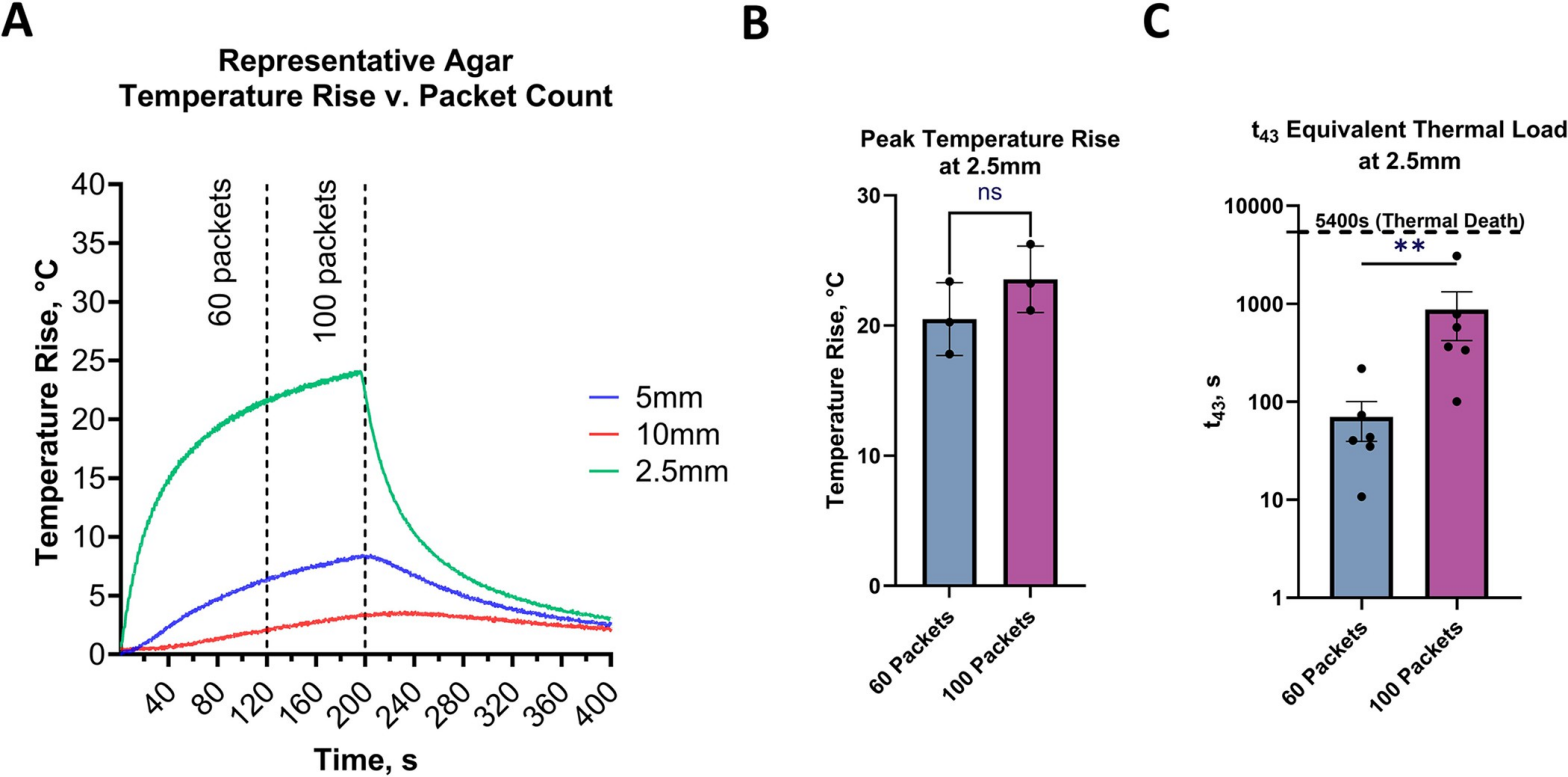
Temperature rise in Agar. A) Representative Agar Temperature Rise vs. packets Count: The graph illustrates temperature rise at various depths (2.5 mm, 5 mm, and 10 mm) in agar as a function of the number of packets (n). The 2.5 mm depth shows the highest temperature rise, peaking at around 60 packets before declining. B) Peak Temperature Rise at 2.5 mm: Bar graph comparing the peak temperature rise at 2.5 mm depth for 60 and 100 packets. The average temperature rise is approximately 19°C for 60 packets and 21°C for 100 packets, with no significant difference.C) t_43_ Equivalent Thermal Load at 2.5 mm: Bar graph comparing the thermal load (*t*_43_,s) at 2.5 mm depth for 60 and 100 packets. The *t*_43_ values are significantly higher for 100 packets compared to 60 packets, indicating a greater thermal load with increased packets count. The thermally induced death threshold (5400s) is marked for reference.

The agar testing had an average impedance of 122 ± 2.9 Ω. Representative temperature rise profiles at 2.5, 5.0, and 10.0 mm from the needle electrode can be seen in (Fig 4A). At 2.5 mm from the electrode, the average temperature rise for the 60 and 100 packet protocols were 17.8° and 21.2°C, respectively (Fig 4B). At 5 mm from the needle, the temperature rise did not exceed 6.4°C for 60 packets and 8.3°C for 100 packets. Further, the t_43_ equivalent thermal load at 2.5mm from the needle was 70s for the 60 packet dose as opposed to 874s for 100 packets (Fig 4C). Notably, while there were small differences in t_43_, neither thermal load exceeded the 5400s considered sufficient to induce thermal-based cell death, and neither had max temperatures exceed the 70 to 80°C levels of concern for extracellular protein coagulation. It should be noted that all experiments represent a worst-case agar phantom condition (no blood perfusion heat sink), and all t_43_ values remain well below the 5400s threshold associated with cancer cell death from thermal ablation modalities [16,17].

*Tumor Response Characteristics*

### Primary and Secondary Tumor Growth

**Fig 5:**
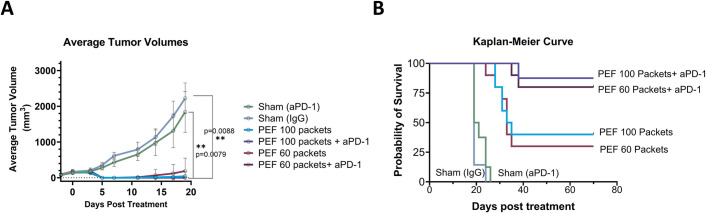
Tumor growth and survival probability in experimental groups. A) The graph illustrates the average tumor growth curve of experimental groups up to day 20, with all mice surviving. B) The Kaplan-Meier curve shows survival probability for mice with aPD-1 therapy and PEF (100 or 60 packets).

Fig 5A illustrates the average growth curves for the primary tumors as function of treatment group (S3 Fig shows individual tumor growth curves). As expected, primary tumor growth declined rapidly independent of PEF packets count (60 vs 100) in the presence or absence of aPD-1. This decline was observed when compared to both the aPD-1 and IgG controls (p < 0.0001). Only 3/10 and 2/10 mice had persistent primary tumors with PEF alone using 60 or 100 packets, respectively, which was not statistically significant (p = 0.22). Further no residual primary tumor was observed when either PEF packet dose was combined with aPD-1 (0/18).

**Fig 6:**
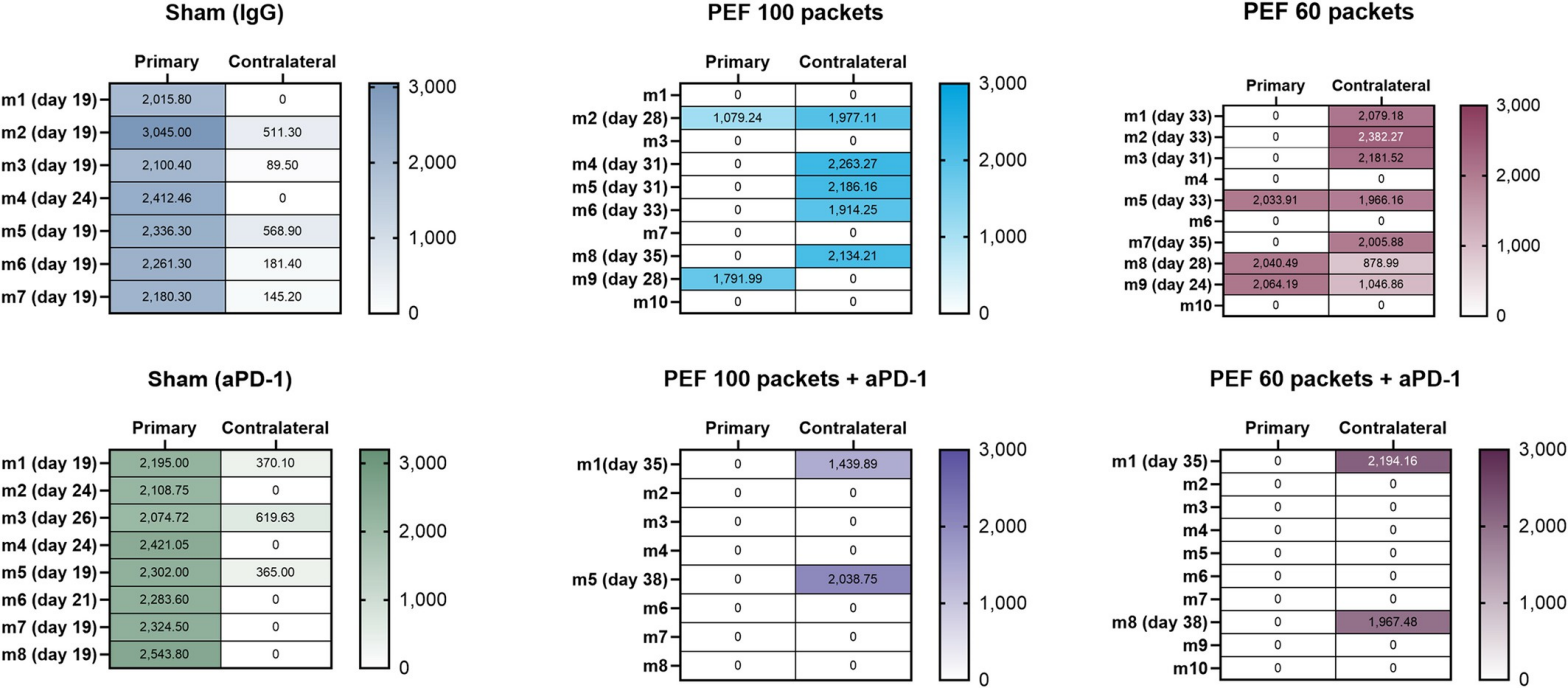
Primary and contralateral tumor response. The colored heat map presents group response scores, depicting tumor growth and complete response in primary and secondary tumors to assess the abscopal effect. The control group, devoid of Aliya PEF treatment, exhibits contralateral tumor growth or death to the rapid expansion of the primary tumor. Conversely, groups treated with PEF (100 or 60 packets) demonstrate a decelerated growth rate or complete inhibition of secondary tumors. Notably, 3/10 PEF (100P) mice and 2/10 PEF (60P) mice achieve complete response. In group 100P+aPD-1, 6/8 mice attain complete response, and in 60 packets + aPD-1, 8/10 mice achieve complete response, a response sustained until day 80.

Contralateral tumor growth representing non-PEF treated tumors is shown in (Fig 6). In the IgG and aPD-1 control groups, 2/7 and 4/8 mice required euthanasia due to excessive tumor burden, respectively. There were no statistically significant differences in contralateral growth between these two control groups (p = 0.226). Comparison between 60 and 100 PEF packets alone showed persistent contralateral tumor growth in 8/10 and 7/10 mice; however, tumor was cleared in 2/10 and 3/10 mice suggestive of an off-target response. Of note, there was a trend that resolution of the primary tumor was more likely to have no residual contralateral disease. When PEF was combined with aPD-1, contralateral disease was cleared in 8/10 and 6/8 mice when the primary tumors received 60 and 100 packets, respectively (Fig 6). This dramatic effect on secondary tumor growth was not statistically significant between the 60 and 100 packet doses (p = 0.936). Most importantly, the findings are consistent with a reproducible systemic response that was partially observed with aPD-1 administration alone, although the primary tumor growth often required euthanasia earlier than secondary tumors may grow, similar to what occurred for IgG-only controls.

Kaplan-Meier survival curves reflect the growth curves and outcomes observed in Figs 5A and 6 for primary and secondary tumors, respectively (Fig 5B). PEF-alone (60 vs 100 packets) had a moderate survival benefit as compared to controls with no difference as a function of dose (p = 0.816) (Fig 6). When combined with aPD-1, survival was increased for both the 60 and 100 packet doses, reaching a log-rank p-value for the Kaplan-Meier survival curve of (p<0.0001) relative to PEF doses alone or aPD-1-alone. Thus, while these findings reflect disease resolution of an ablated primary tumor and contralateral unablated disease, the survival curves between 60 versus 100 packets were not statistically different, with the only delineation in responses occurring via survival improvements when aPD-1 was added to the ablation.

### Cytokine Expression

**Fig 7:**
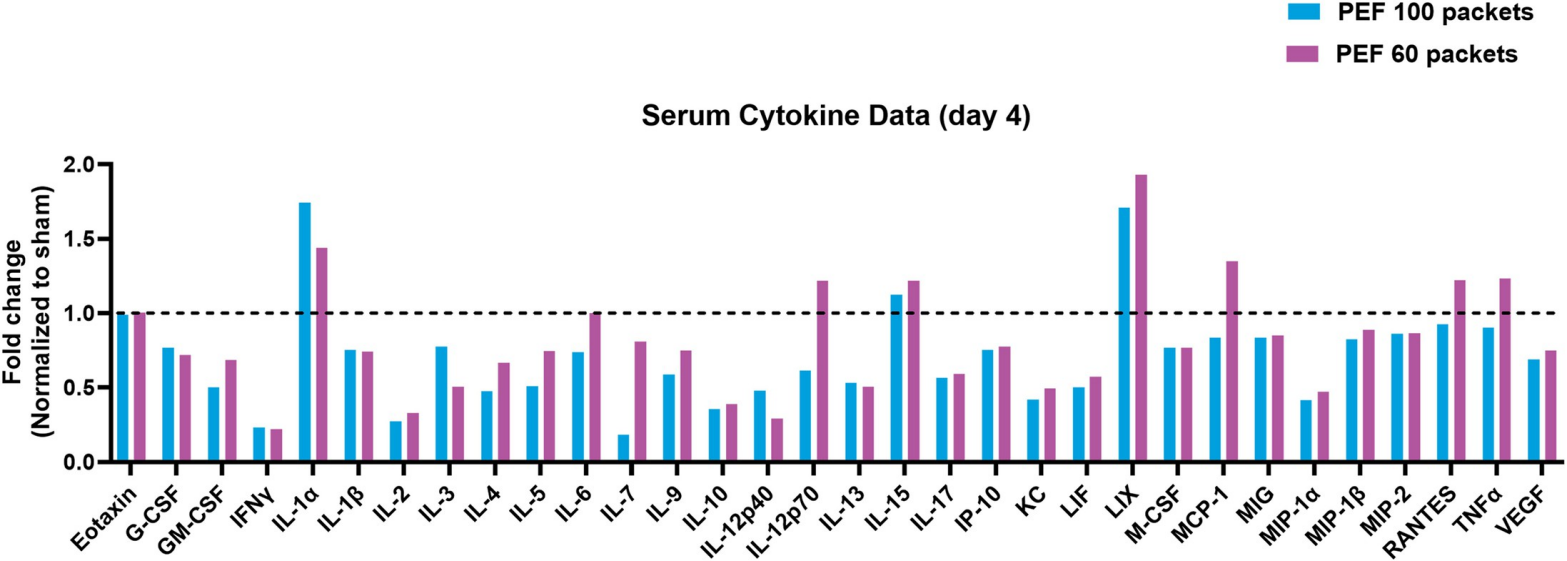
Serum cytokine data on day 4 post-treatment. The bar graph displays the fold change in cytokine levels, normalized to the sham group, for two different PEF treatments: 100P (blue bars) and 60P (purple bars). There are no Significant differences in cytokine levels between the two treatments.

**Fig 8:**
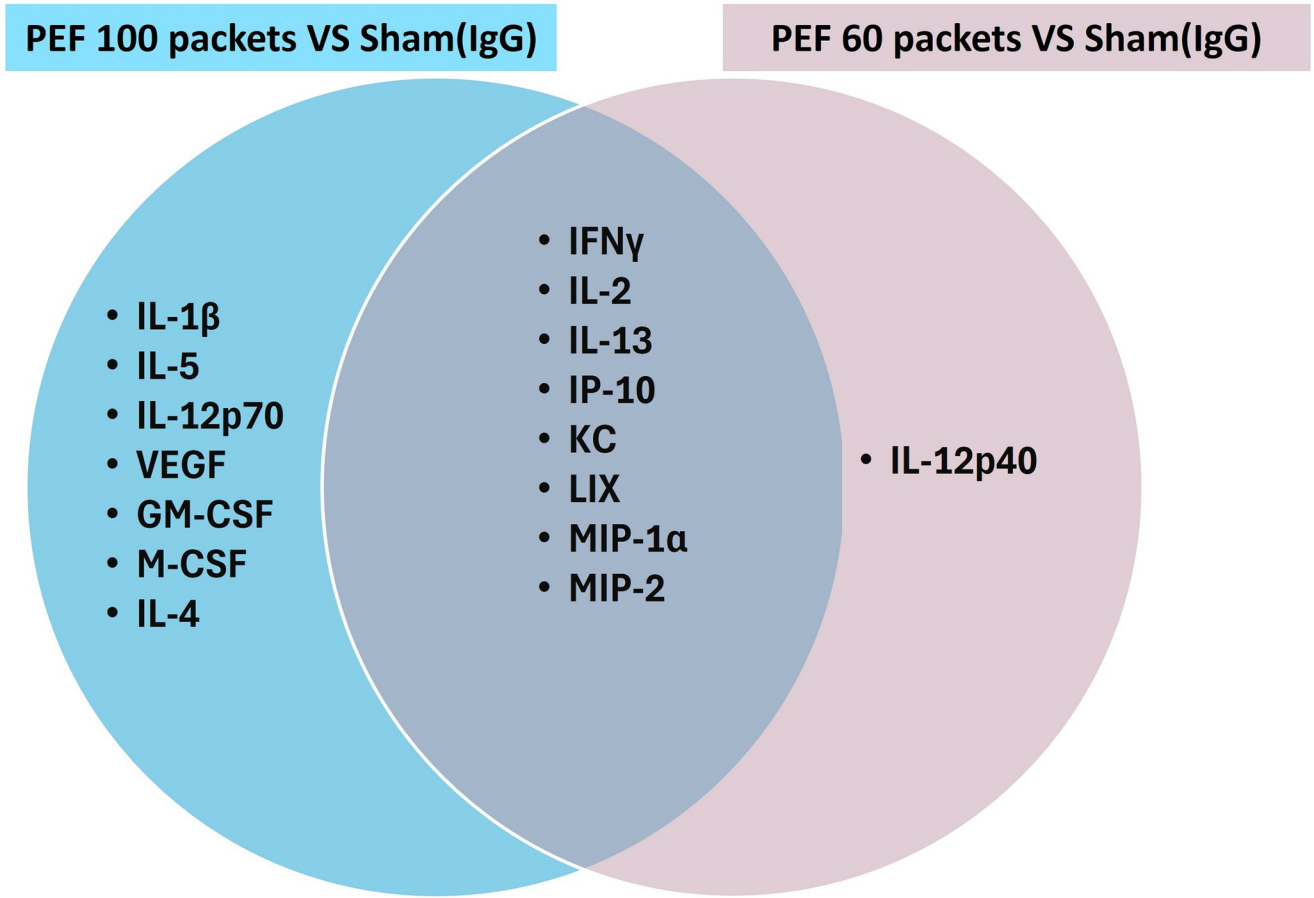
Comparative analysis of significant cytokine differences. This Venn diagram depicts the cytokines that demonstrated statistically significant differences between two treatment groups in a multiple comparison t-test analysis. The left circle (blue) represents cytokines with significant differences after treatment with PEF 100 packets (PEF 100P) compared to the Sham (IgG) group. The right circle (purple) represents cytokines with significant differences following treatment with PEF 60 packets (PEF 60P) compared to the Sham (IgG) group. The overlapping region indicates cytokines that were significantly different in both PEF 100P vs. Sham and PEF 60P vs. Sham comparisons, highlighting the common cytokines affected by both PEF treatments when compared to the Sham group. Cytokines exclusive to each treatment are specified within their respective circles, while the shared cytokines are listed in the intersection.

The study conducted an in-depth analysis of 32 cytokine levels in response to PEF treatment, comparing the effects of different treatment intensities and controls, with analyses profiled on mouse serum day 4 post-treatment of PEF (Fig 7). Overall, cytokine expression profiles observed on day 4 showed considerable consistency between the 60 and 100 packets PEF doses as compared to the IgG controls. Both the 60 and 100 packet doses showed statistically significant differences with cytokines associated with pro-inflammatory (GM-CSF, M-CSF, LIX, KC, MIP-1α, MIP-2), Th1 (IL-2, IFN-γ, IL-12p70, IP-10), and Th2 (IL-13) responses Table 2. However, unlike the 100 packets dose, no statistically significant differences with pro-inflammatory injury (VEGF, IL-1β) and Th2 (IL-4) associated cytokines were observed with the 60 packets dose compared to the IgG control. The results show that 15 and 9 cytokine levels were statistically different for the 100 and 60 packets groups relative to IgG sham control, respectively Table 2, with 8 overlapping for both groups, as shown in the Venn diagram (Fig 8).

**Table 2:** Comparative analysis of cytokine levels between PEF 100 packets and PEF 60 packets.

Cytokines	Mean of Sham	Mean of PEF (100P)	q. value Sham V PEF (100 P)	Mean of PEF(60P)	q value Sham V.PEF(60 P)	q value PEF (100P)V.PEF(60 P)	Significance Between PEF (100 P. Vs.60P)
**Eotaxin**	186.9	185.3	0.738	188.000	0.808	0.976087	No
**G-CSF**	288.3	221.3	0.380	208.000	0.277	0.976087	No
**GM-CSF**	10.7	5.356	0.010	7.355	0.066	0.776393	No
**IFN-γ**	7.349	1.708	0.000	1.621	0.000	0.976087	No
**IL-1α**	133.5	232.6	0.114	191.800	0.434	0.776393	No
**IL-1β**	4.68	3.521	0.082	3.466	0.180	0.976087	No
**IL-2**	16.32	4.46	0.000	5.387	0.000	0.776393	No
**IL-3**	0.5114	0.396	0.366	1.319	0.536	0.776393	No
**IL-4**	0.4529	0.216	0.002	0.302	0.116	0.776393	No
**IL-5**	6.317	3.214	0.048	4.723	0.344	0.195294	No
**IL-6**	9.066	6.68	0.297	9.062	0.820	0.815109	No
**IL-7**	7.086	1.317	0.533	5.745	0.715	0.776393	No
**IL-9**	83.46	49.19	0.159	62.630	0.549	0.776393	No
**IL-10**	8.051	2.868	0.130	42.360	0.549	0.776393	No
**IL-12p40**	14.18	6.809	0.114	4.179	0.003	0.776393	No
**IL-12p70**	17.22	10.57	0.003	20.980	0.647	0.776393	No
**IL-13**	28.59	15.22	0.001	14.410	0.000	0.776393	No
**IL-15**	81.02	91.14	0.719	98.760	0.715	0.976087	No
**IL-17**	1.04	0.591	0.114	2.776	0.571	0.776393	No
**IP-10**	42.68	32.13	0.010	33.140	0.027	0.889345	No
**KC**	65.72	27.58	0.003	32.450	0.021	0.776393	No
**LIF**	3.819	1.911	0.533	2.188	0.434	0.776393	No
**LIX**	1634	2790	0.045	3153.000	0.000	0.776393	No
**M-CSF**	9.796	7.535	0.045	7.520	0.076	0.987192	No
**MCP-1**	43.61	36.36	0.366	58.780	0.596	0.776393	No
**MIG**	1096	917.7	0.122	933.500	0.434	0.976087	No
**MIP-1α**	63.38	26.4	0.001	29.820	0.001	0.776393	No
**MIP-1β**	53.91	44.53	0.104	47.890	0.165	0.776393	No
**MIP-2**	158.8	136.6	0.016	137.200	0.035	0.976087	No
**RANTES**	18.63	17.21	0.533	22.740	0.596	0.776393	No
**TNFα**	6.053	5.477	0.156	7.458	0.596	0.776393	No
**VEGF**	0.7771	0.537	0.022	0.582	0.125	0.776393	No

Direct comparison between the 100 and 60 packet doses found no statistically significant differences across the entire 32-cytokine panel. This indicates a strong correlation in the PEF-induced systemic cytokine changes regardless of which packets count was used. These correlations in trend relative to sham further reinforce the similarities in cytokine changes and response to the PEF ablation. Overall, these data suggest that the sub-cellular responses to PEF injury are very similar between the 60 and 100 packets groups, suggesting consistency in the downstream immune effect that may be produced.

Table 2 presents the comparative cytokine profiles between two intensities of PEF treatment, 100 packets (100P) and 60 packets (60P), based on serum analysis of 32 different cytokines. The significance of differences was assessed using a two-tailed t-test. Columns labeled ’Mean of PEF (100P)’ and ’Mean of PEF (60P)’ show the average levels of cytokines for the respective treatment groups. The ‘q value’ column provides the precise q values obtained from the t-test, adjusted for multiple comparisons. The ’Significance’ column reflects the presence of statistically significant differences. As indicated by the uniform ’No’ in the ’Significance’ column, this study found no statistically significant differences in the cytokine profiles when comparing PEF 100 packets to PEF 60 packets.

### Flow Cytometry Immune Cell Populations

Flow cytometry analysis of blood drawn +13 days post-PEF delineated several key immune cell populations for each of the treatment groups compared to the IgG and aPD-1 controls. S2 Fig provides representative gating data for delineating the detection of T cells, B cells, and NK cells, while S3 Fig provides the representative gating strategy for subpopulations of CD3 T cells, including CD4 helper T cells, CD8 cytotoxic T cells, and the CD4 and CD8 subpopulations of effector memory (EM), central memory (CM), and double-negative (DN) T cells.

**Fig 9:**
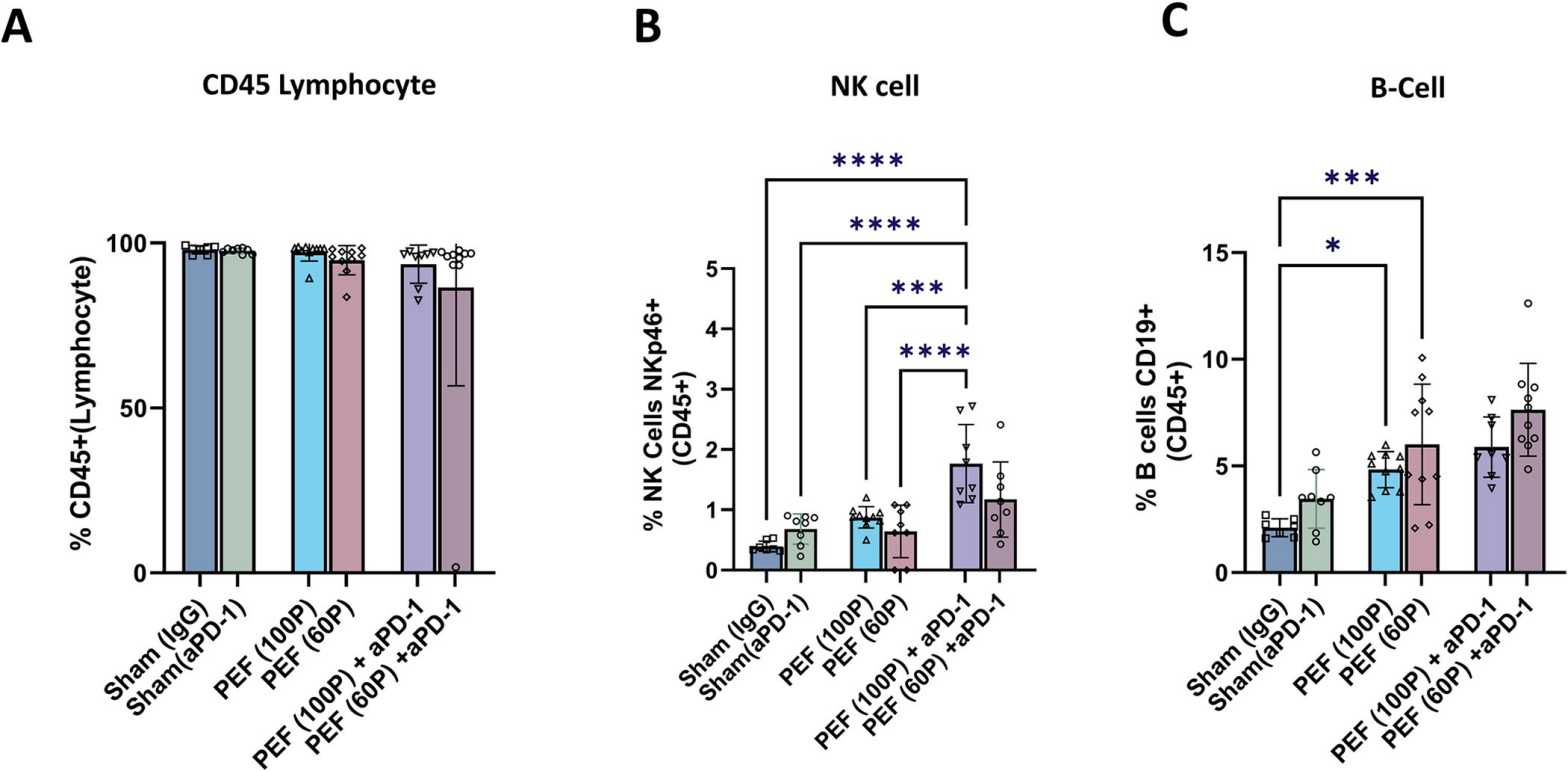
Profiling immune cell percentages in peripheral blood 13 days post-treatment with PEF (100 P) and PEF (60 P) packets. where ’p’ indicates the number of packets. A. Illustrates the CD45% population in all groups. B. Presents the NK cell population dynamics. C. Depicts B cell variations across the experimental cohorts. Statistical significance, determined through one-way ANOVA analysis by Prism, is denoted by P values, with symbols indicating significance levels: *** P < 0.001, ** P < 0.01, * P < 0.05, and ’ns’ for non-significant results.

By day 13, there were no significant differences observed involving total populations of CD45+ lymphocyte percentages as compared to controls (aPD-1and IgG) across all treatment groups (PEF, PEF + aPD1) (Fig 9A). While the percentage of NK cells did not differ between the 60 and 100 packet doses with PEF-alone (p = 0.68), the addition of aPD-1 to PEF resulted in a statistically significant increase which was more pronounced with the 100 packets dose in comparison with 100 packets PEF (p = 0.008) (Fig 9B).

The percentage of CD19+ B cells did not reveal any differences when comparing the IgG to aPD-1 controls (p = 0.426) (Fig 9C). Significant increases (p = 0.01) were noted in the percentage of CD19+ B-cells in both the PEF-alone and PEF + aPD-1 treatment groups that were neither treatment-dependent (PEF alone vs PEF + aPD-1) or dose-dependent (60 vs 100 packets).

**Fig 10:**
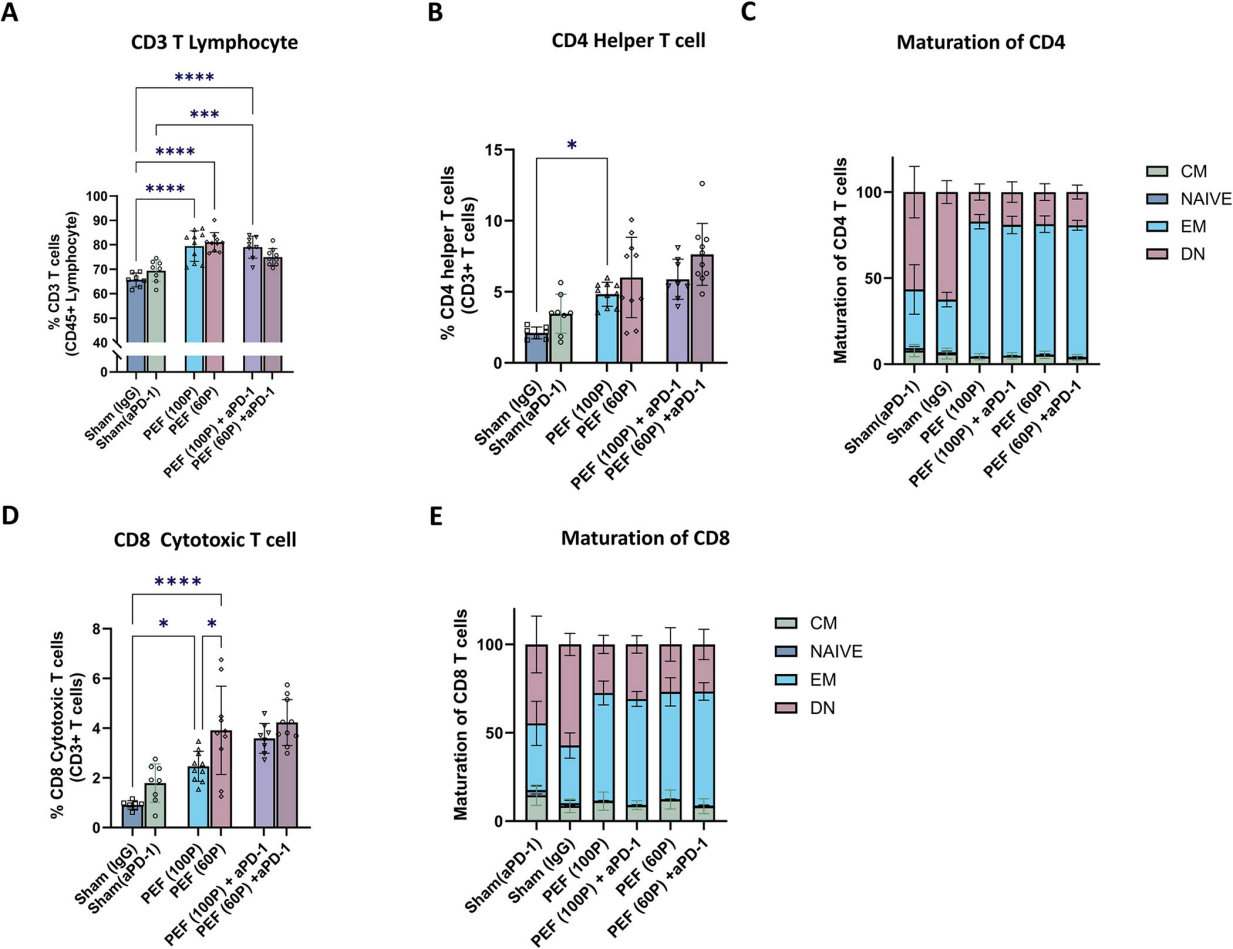
Profiling immune cell percentages in peripheral blood 13 days post-treatment with PEF (100 P) packets and PEF (60 P) packets. where ’p’ indicates the number of packets. A. Illustrates the CD4 helper T cells percentage in all groups. B. Illustrates CD8 cytotoxic T cell percentage across the experimental cohorts. C. Presents the CD4 subpopulation, including EM, CM, DN, and naïve, in all treated groups. D. Presents the CD8 subpopulation, including EM, CM, DN, and naïve, in all treated groups statistical significance, determined through one-way ANOVA analysis by Prism, is denoted by P values, with symbols indicating significance levels: *** P < 0.001, ** P < 0.01, * P < 0.05, and ’ns’ for non-significant results.

Compared to sham controls, CD3+ T cell proportions increased in both the PEF alone (p = 0.002) and PEF + aPD-1 (p = 0.008) treatment groups that were neither treatment-dependent (PEF alone vs PEF + aPD-1) or dose-dependent (60 vs 100 packets) (Fig 10A). Assessment of CD4 helper and CD8 cytotoxic T cells is shown in (Fig 10B). No significant differences were seen between the IgG and aPD-1 controls. The incorporation of PEF-alone or in combination with aPD-1 exhibited significant proportional increases in both CD4 helper (p = 0.001) (Fig 10B) and CD8 cytotoxic (p = 0.006) (Fig 10D) T cells. The only significant dose-dependent difference was an increase in the percentage of CD8 T cells observed in the PEF-only 60 packets compared with 100 packets doses (p = 0.016) (Fig 10D).

Within the T cell subpopulations, significant increases in effector memory CD4 (p = 0.001) (Fig 10C) and CD8 (p = 0.001) (Fig 10D) T cells were observed with the incorporation of PEF-alone or in combination aPD-1, which was not dose-dependent (60 vs 100 packets) (p = 0.723). This also resulted in a corresponding decrease in the double negative CD4 (Fig 10C) and CD8 (Fig 10D) T cell sub-populations. Lastly, central memory T cells were relatively unchanged across all controls and treatment groups.

Collectively, these flow cytometry findings, with some noted exceptions, support that PEF invokes an evolution from innate to adaptive Th1 and Th2 immune responses, which is enhanced when PEF is combined with aPD-1, in a manner that is independent between the two packet counts delivered in this study. These outcomes suggest that increasing the packets count from 60 to 100 packets does not progressively increase the CD4 and CD8 T cell populations, nor their subpopulations, indicating a plateau in the immunomodulatory effect may be reached by 60 packets, with no marked increases up to 100 packets. This stability suggests that the treatment, regardless of the packets dosage in this range, does not markedly affect these crucial components of the adaptive immune system.

## Discussion

A key development in the field of PEFs is the preclinical evidence demonstrating immunostimulatory downstream effects beyond the ablation due to the blend of regulated cell death mechanisms and maintaining the structural integrity of the extracellular matrix and its tertiary structures [5,18]. Therefore, it was important to understand if interdependencies between packets count, ablation dimensions, and immune-modulation existed. The relationship of PEF packet dose in terms of ablation and biologic response was evaluated through increasingly complex experiments. A 60 packets dose of PEF was identified as a minimally effective dose that elicited comparable ablation and biologic characteristics as compared to a 100 packets benchmark.

The ablation parameters investigated in most of the studies were first identified in the tuber model and validated by the porcine liver studies using TTC staining. The only discernible difference was noted in the agar phantom experiments, where the increased thermal load observed from 100 packets relative to the 60 packets dose. In that evaluation, the 60 packets dose reduced the maximum agar phantom temperature reached 2.5 mm from the needle electrode by 16% and decreased the thermal equivalent t_43_ dose by 92%. Notably, the thermal dose for both 60 and 100 packets doses at 2.5 mm from the needle electrode remained well below the 5400 s equivalent dose associated with cell death of mammary carcinoma [19], and the calibrated max temperatures remain below the 70–80°C threshold for rapid onset of extracellular protein coagulation that may affect the safety profile of the technology [13]. Thus, while there were subtle differences in temperature rise between the two doses used in the rest of the study, these differences are reflected in the actual use, and did not appear to influence the outcomes on ablation size nor immune response characteristics.

The agar phantom model is a non-perfused “worst case” scenario, which further supports mechanisms of cell death beyond thermal ablation. In clinical practice, the specific Aliya PEF waveform and parameters were pre-determined to attenuate the extent of temperature rise to remain below the approximately 70°C where accelerated protein denaturation may compromise extracellular matrix integrity, as commonly encountered in thermal ablation [7,16,17]. It is also postulated that protein denaturation caused by Joule heating may compromise the tumor microenvironment and immunocyte infiltration, adversely impacting biologic responses after PEF injury [7]. Thus, by remaining below the realm of coagulation necrosis, it may be possible for PEF to generate stronger immune responses than thermal ablation, as demonstrated [7].

A limitation of the study is that gross pathology was used to compare in vivo porcine liver ablation sizes, but histological characterization of ablated tissue characteristics was not performed. Future studies may seek to determine the histological and immunohistological tissue changes within and adjacent to the ablation zone for potential differences in the downstream immune responses. Further, this study used relatively low trial numbers in the reported potato and in vivo liver ablation sizes.

This study only examined the influence of a single, controllable, parameter for the PEF delivery used, with only two settings used for the most complex evaluations of influence on outcomes of ablation in healthy tissue and immune response in a murine cancer model. The key measurable difference for this parameter at the minimum effective dose (60 packets) and maximum dose (100 packets) was in temperature change. The packet count setting was targeted in this study because it was deemed less likely to influence the complex biological interactions for PEF dosing. Further, this evaluation was only performed for the baseline waveform that represents PEFs for a single commercially available system, and that similar evaluations of packet count optimization and influence on immune response should be performed for other technologies.

The immune response implications of different packet counts were evaluated with the EMT6 orthotopic breast tumor model, a model generally regarded as mildly to unresponsive to immunotherapy. It should be noted that the highly diverse nature of different clinical cancers, including their response to injury and interactions with the immune system may influence the relative immune response findings for the different packet doses evaluated in this study. While similar observations of general immune upregulation have been reported in non-small cell lung cancer [20], future studies are required to evaluate the immune response in different tumor models and in combination with different standard of care systemic therapies, particularly with respect to the role of dose on these effects.

When evaluating biologic effect and immune response implications, the similarities between the 60 and 100 packet parameter sets resulted in equivalent growth kinetics of treated and untreated contralateral tumors, as well as mouse survival from tumor burden, which were all significantly improved for both PEF doses relative to sham or aPD-1-alone. Further, these improvements were enhanced when aPD-1 was added to the PEF, but not different between the 60 packets versus 100 packets combinations. This also contributed to the most significant survival benefit, suggesting that the 60 packets dose may be a minimally effective dose, especially when combined with aPD-1 checkpoint inhibitor.

The EMT6 tumor model demonstrated the ability of PEF to elicit off-target tumor responses with partial ablation of the primary tumor in the mammary fat pad. While the off-target responses were less impressive, the PEF-alone cohorts provided the best measure to assess dose-dependent differences. Both 60 and 100 packets cleared the majority of the primary tumors. Further, both the 60 and 100 packets doses yielded comparable survival benefits as compared to controls. The average tumor burden in mice with persistent disease was numerically lower with the 100- versus the 60 packets dose. This could represent a slight improvement in the ablation characteristics of the 100 packet dose or maybe attributed to the limited number of mice studied. Such comparisons may be better quantified by expanding the number mice per cohort, as well as obtaining specimens from PEF-ablated tumors over time for histologic evaluation. More compelling than baseline PEF outcomes was the enhanced primary and off-target tumor responses observed when PEF was combined with aPD-1 as compared to aPD-1 alone which showed no primary tumor control. In this context, there were no apparent differences observed between the 60 and 100 packet doses.

Mechanisms governing PEF biologic response beyond ablation were indirectly evaluated through serum cytokine profiles (Day 4) and flow cytometry of immune cells (+13 days). From the cytokine analysis, considerable overlap between the 60 packet and 100 packet PEF doses was observed with respect to cytokines associated with pro-inflammatory, Th1, and Th2 responses as compared to controls. With all other characteristic parameters held equal, only 1 of the 32 explored demonstrating a statistically significant difference between the two packet count doses. However, unlike the 100 packet dose, the 60 packets did not show a statistically significant difference with VEGF or IL-1 (pro-inflammatory) and IL-4 or IL-5 (Th2) expression relative to controls. As this was a single measurement in time, it is difficult to draw any conclusions if this dose- or time-dependent. Future studies should assess time-dependent changes before and after day 4 to determine if these were dose-dependent differences or temporal artifacts. What can be inferred is that both the 60- and 100 packets doses were predictive biomarkers of subsequent circulating immune cells characterized by flow cytometry.

At day 13, flow cytometry revealed unique immune cells and sub-populations associated with the innate and adaptive Th1 and Th2 immune responses predictive of survival. The significant cytokine similarities correlated with equivalent CD45+ immune populations, as well as equivalent B cells, NK cells, CD4 T cells and subpopulations, and CD8 T cells and subpopulations. The equivalent immune cell populations between the two packets counts were maintained regardless of whether it was a PEF-alone condition, or if the PEF packet count groups included aPD-1 checkpoint blockade. While these responses were observed with PEF-alone, they were enhanced in combination with aPD-1 and not dose-dependent (60 vs 100 packets). While this data provides some insight in the proposed immune-mediated mechanisms, a limitation is that it only represents immunological response from a single point in time from peripheral blood. Future studies should examine local cellular infiltrates within the tumor microenvironment over time with additional assessment of secondary lymphoid organs such as the spleen. Lastly, the mechanisms underpinning the ability of PEF to overcome the inherent resistance to aPD-1 therapy in combination is a critical area of future investigation not addressed in these experiments. In the case of recurrent NSCLC, understanding and overcoming acquired resistance to aPD-L1 therapy is an area of intense investigation [21]. It should be noted that these observations are preliminary and specific to the EMT6 tumor model. While the current Aliya PEF commercial system recommends a 100 packet dose, the observations made in this work require further validation, not only in additional preclinical models, but also in prospective clinical studies, when appropriate, before any adjustments to current recommendations would be appropriate.

## Conclusion

This work provides an early assessment of the PEF dose-response relationship in terms of ablation and immune modulation. Preliminarily, a PEF dose of 60 packets was identified as a minimally effective dose that maintains both the basic ablation and murine immune-modulating characteristics as compared to the 100 packet benchmark. Both doses demonstrated similar effects on immune cell populations and tumor control, particularly when combined with aPD-1 checkpoint blockade. These similarities reflect the equivalent primary tumor volume reduction and unablated contralateral tumor control found for both doses. While there remain questions that require further investigation, this works provides a basis from which to optimize PEF ablation doses and outcomes for different disease-specific clinical priorities.

## Supporting information

S1 Table Cell surface markers used for characterization of immune cell types in flow cytometric analysis. (PDF)

S1 Fig Gating strategy for detection of T cells, B cells, and NK cells. All samples were processed and analyzed on the Cytoflex instrument by Beckman Coulter. Gating steps included selecting all leukocytes, singlet selection, live cell gating, CD45 population selection, exclusion of CD3-positive population for T cells, and subsequent identification of NK cells by selecting cells expressing anti-mouse NKp46. Additionally, CD19-positive cells were selected using anti-mouse CD19 antibodies for the detection of B cells.
(TIF)

S2 Fig Gating strategy for detection of T cell subpopulations on Cytoflex. Gating strategy employed for the detection of CD3 T cells, CD4 helper T cells, CD8 cytotoxic T cells, and subpopulations of CD4 and CD8, including effector memory (EM), naïve, central memory (CM), and double-negative (DN) cells. All samples were processed and analyzed on the Cyto flex instrument by Beckman Coulter. Gating steps included selecting all leukocytes, singlet selection, live cell gating, CD45 population selection, followed by the identification of CD3e T cells, CD4 helper T cells (CD3e+CD4+), and CD8 cytotoxic T cells (CD3e+CD8a+). Within the CD4 or CD8 gate, subpopulations were further specified as EM (CD44+ CD62L-), CM (CD44+ CD62L+), naïve cells (CD62L+ CD44-), and double-negative (DN) cells (CD44- CD62L-).
(TIF)

S3 Fig Individual tumor growth curves for primary tumor across treatment groups. (TIF)

S1 Raw data (ZIP)
