# Supplementary material for: Understanding the aliya pulsed electric field dose-response relationship: Implications for ablation size, thermal load, and immune response in an orthotopic murine breast cancer model
Source: PLoS One. 2025 Feb 13;20(2):e0318440. doi: 10.1371/journal.pone.0318440 (PMC11824980; doi:10.1371/journal.pone.0318440)
Supplement: S1 Table — (PDF) [file pone.0318440.s001.pdf]

**Antibody**	**Vendor and Catalog number**
Anti-mouse CD45	BioLegend, 103126
Anti-Mouse CD3ε	BioLegend, 100216
Anti-Mouse CD8a	BioLegend, 100753
Anti-Mouse CD4	BD Biosciences, 130308
Anti-mouse CD44	BioLegend, 103032
Anti-Mouse CD62L	BD Biosciences, 553152
NKp46	Biolegend,137604
CD19	Biolegend,115512
