# Supplementary figures and images for: Understanding the aliya pulsed electric field dose-response relationship: Implications for ablation size, thermal load, and immune response in an orthotopic murine breast cancer model
Source: PLoS One. 2025 Feb 13;20(2):e0318440. doi: 10.1371/journal.pone.0318440 (PMC11824980; doi:10.1371/journal.pone.0318440)

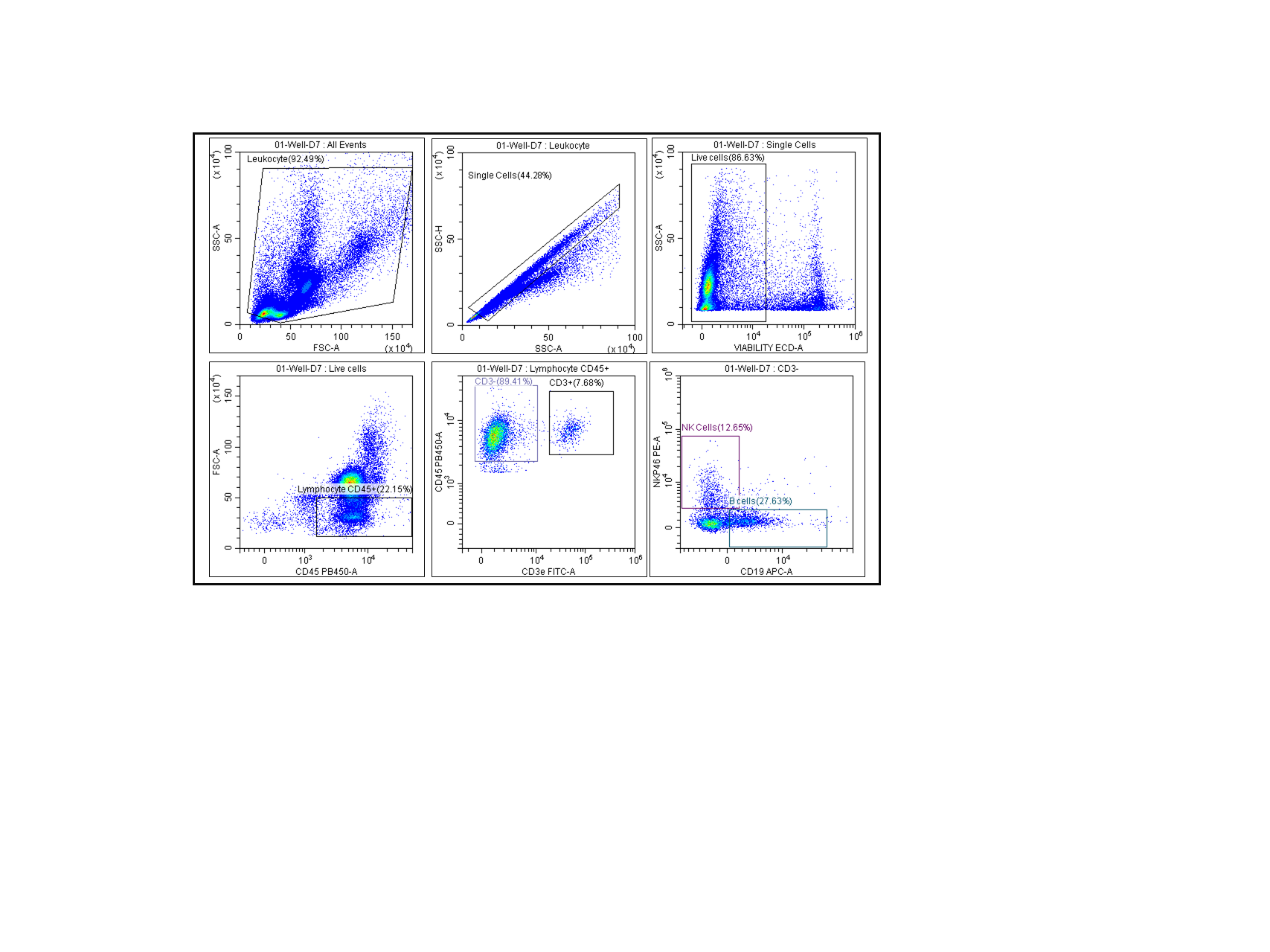

Supplement: S1 Fig — All samples were processed and analyzed on the Cytoflex instrument by Beckman Coulter. Gating steps included selecting all leukocytes, singlet selection, live cell gating, CD45 population selection, exclusion of CD3-positive population for T cells, and subsequent identification of NK cells by selecting cells expressing anti-mouse NKp46. Additionally, CD19-positive cells were selected using anti-mouse CD19 antibodies for the detection of B cells. (TIF) [file pone.0318440.s002.tif]

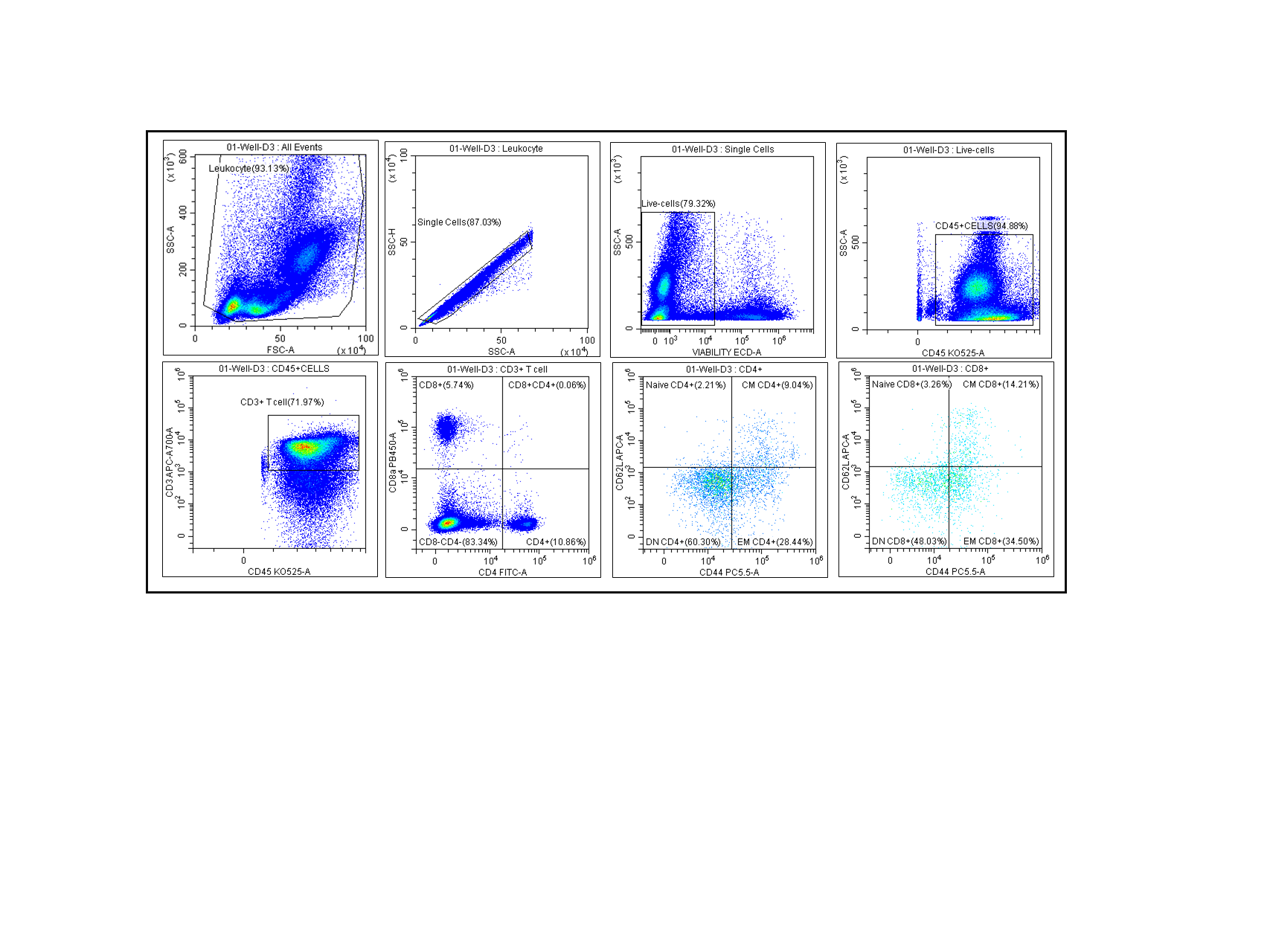

Supplement: S2 Fig — Gating strategy employed for the detection of CD3 T cells, CD4 helper T cells, CD8 cytotoxic T cells, and subpopulations of CD4 and CD8, including effector memory (EM), naïve, central memory (CM), and double-negative (DN) cells. All samples were processed and analyzed on the Cyto flex instrument by Beckman Coulter. Gating steps included selecting all leukocytes, singlet selection, live cell gating, CD45 population selection, followed by the identification of CD3e T cells, CD4 helper T cells (CD3e+CD4+), and CD8 cytotoxic T cells (CD3e+CD8a+). Within the CD4 or CD8 gate, subpopulations were further specified as EM (CD44+ CD62L-), CM (CD44+ CD62L+), naïve cells (CD62L+ CD44-), and double-negative (DN) cells (CD44- CD62L-). (TIF) [file pone.0318440.s003.tif]

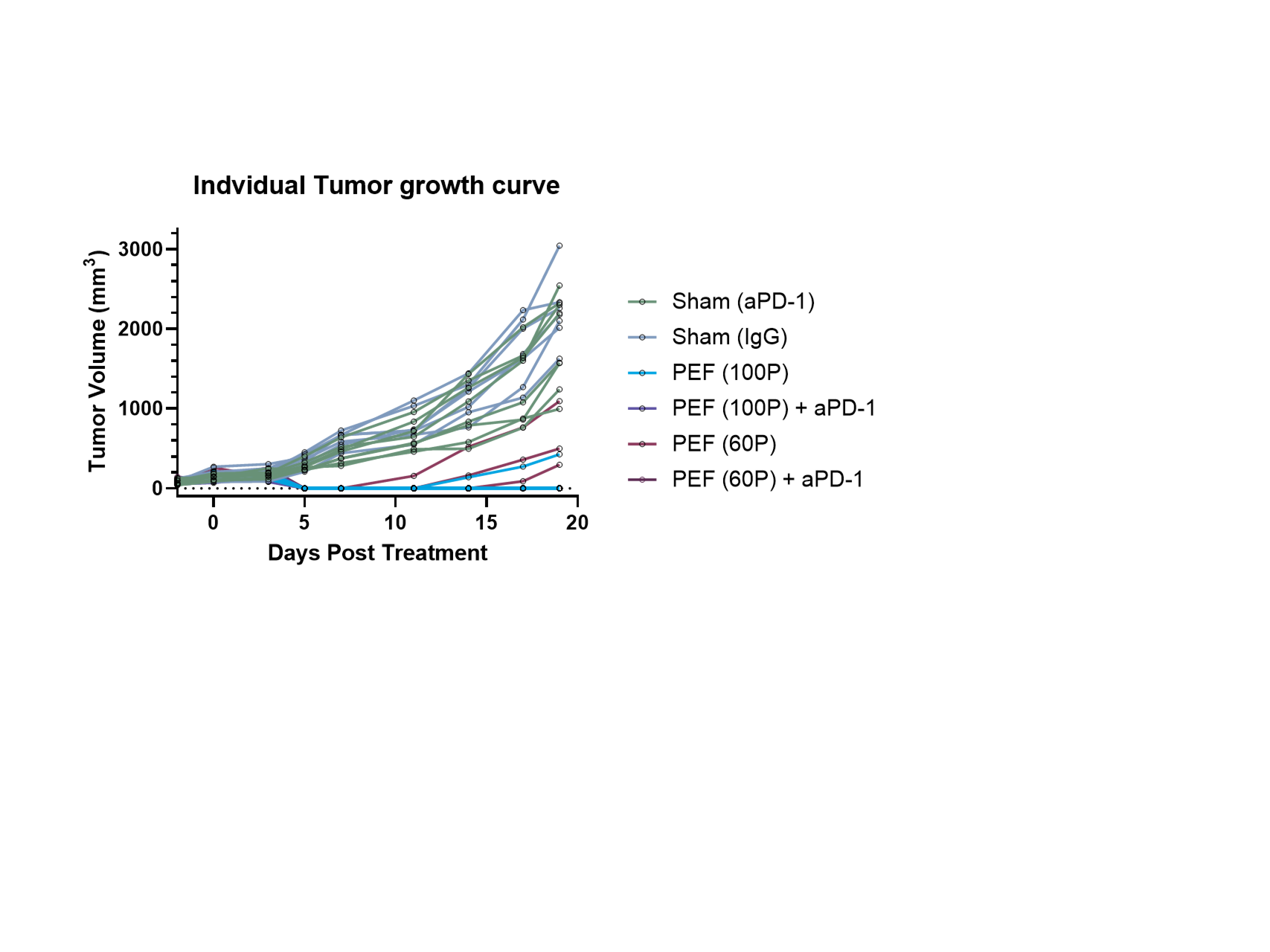

Supplement: S3 Fig — (TIF) [file pone.0318440.s004.tif]
