# Supplementary material for: Understanding the aliya pulsed electric field dose-response relationship: Implications for ablation size, thermal load, and immune response in an orthotopic murine breast cancer model
Source: PLoS One. 2025 Feb 13;20(2):e0318440. doi: 10.1371/journal.pone.0318440 (PMC11824980; doi:10.1371/journal.pone.0318440)
Supplement: S1 Raw data — (ZIP) [file pone.0318440.s005.zip › S4 Fig raw data.pdf]

Figure S4 supplement

Days Post Treatment				Sham (aPD-1)				
-2	62.96	54.01	40.75	40.6	67.01	128.01	97.75	98.5
0	149.91	179.82	85.29	99.59	105.3	139.08	186.53	146.51
3	164.68	158.44	143.94	114.03	189.73	251.42	198.38	186.28
5	284.97	264.43	231.28	233.08	405.2	225.67	267.62	326.26
7	488.75	282.35	316.24	373.76	637.55	375.2	462.23	518.01
11	835.58	490.89	460.89	556.35	956.27	569.71	716.85	646.31
14	1263.41	494.74	578.73	837.62	1351.94	788.77	1433.21	1091.69
17	1636.73	761.48	876.61	1080.41	1666.26	861.42	2020.39	1602.91
19	2195	1241.9	994.6	1572.3	2302	1569.6	2324.5	2543.8

Days Post Treatment				Sham (IgG)			
-2	64.13	66.71	75.37	47.72	88.4	125.06	102.6
0	106.73	205.94	171.21	79.58	269.22	202.3	127.67
3	174.83	200.84	219.87	84.52	302.48	248.98	208.53
5	305.46	451.75	361.97	208.04	387.59	427.25	321.75
7	547.39	725.01	585.05	440.64	668.43	665.29	672.88
11	738.55	1035.8	670.97	548.35	1102.31	728.09	722.05
14	1213.1	1299	764.59	954.77	1442.03	1256.58	1020.7
17	1625.3	2118.65	1268.66	1137.6	2234.18	2004.57	1685.83
19	2015.8	3045	2100.4	1625.4	2336.3	2261.3	2180.3

Days Post Treatment					PEF (100P)					
-2	83.32	71.21	62.61	88.18	72.44	52.81	75.15	80.61	66.65	85.96
0	98	166.86	117.42	210.3	125.61	105.85	122.95	168.91	111.36	182.68
3	114.2	123.43	116.51	155.56	185.48	172.54	120.96	144.69	140.43	192.48
5	0	0	0	0	0	0	0	0	0	0
7	0	0	0	0	0	0	0	0	0	0
11	0	0	0	0	0	0	0	0	0	0
14	0	137.56	0	0	0	0	0	0	0	0
17	0	271.98	0	0	0	0	0	0	0	0

19	0	425.1	0	0	0	0	0	0	0	0

Days Post Treatment					PEF (100P) + aPD-1					
-2		53.32	100.34	63.02	109.55		67.8	70.54	98.96	83.03
0		68.6	154.36	181.05	197.72		202.7	178.07	129.08	184
3		134.19	172.24	221.78	202.69		118.06	79.52	161.47	131.6
5		0	0	0	0		0	0	0	0
7		0	0	0	0		0	0	0	0
11		0	0	0	0		0	0	0	0
14		0	0	0	0		0	0	0	0
17		0	0	0	0		0	0	0	0
19		0	0	0	0		0	0	0	0

Days Post Treatment					PEF (60P)					
-2	55.76	112.54	103.45	104.36	84.73	99.43	82.38	87.81	107.65	102.96
0	185.2	146.11	148.94	195.7	180.71	138.92	126	257.4	194.04	220.89
3	112.1	149.82	109.66	158.23	88.69	110.06	115.91	141.31	179.51	154.4
5	0	0	0	0	0	0	0	0	0	0
7	0	0	0	0	0	0	0	0	0	0
11	0	0	0	0	0	0	0	0	154.48	0
14	0	0	0	0	0	0	0	160.44	520.25	0
17	0	0	0	0	87.42	0	0	359.15	765.17	0
19	0	0	0	0	294.8	0	0	499.4	1092.6	0

Days Post Treatment					PEF (60P) + aPD-1					
-2	93.86	76.19	91.99	98.86	85.67	143.08	94.96	75.91	98.47	77.16
0	176.28	119.38	170.11	155.24	142.88	99.76	139.99	119.59	183.41	133.1
3	166.65	144.48	245.98	221.72	218.85	108.81	110.01	140.68	208	259.25
5	0	0	0	0	0	0	0	0	0	0
7	0	0	0	0	0	0	0	0	0	0
11	0	0	0	0	0	0	0	0	0	0
14	0	0	0	0	0	0	0	0	0	0

17	0	0	0	0	0	0	0	0	0	0
19	0	0	0	0	0	0	0	0	0	0
