# Supplementary material for: Understanding the aliya pulsed electric field dose-response relationship: Implications for ablation size, thermal load, and immune response in an orthotopic murine breast cancer model
Source: PLoS One. 2025 Feb 13;20(2):e0318440. doi: 10.1371/journal.pone.0318440 (PMC11824980; doi:10.1371/journal.pone.0318440)
Supplement: S1 Raw data — (ZIP) [file pone.0318440.s005.zip › Fig 3 raw data.pdf]

Fig 3A

Packet	Short Axis				
	Trial 1	Trial 2	Trial 3	Trial 4	Trial 5
20	14.65	13.88	14.64	13.5	14.12
40	16.31	16.52	14.93	16.69	13.89
50	14.44	17.22	16.85	15.71	16.99
60	16.42	17.63	16.76	16.38	18.15
80	17.22	17.3	15.93	16.15	16.25
100	16.71	17.54	16.43	17.36	16.4

Fig 3B

Ablation Diameter, mm	
60 Packets	100 Packets
12.2	13.6
10.9	12.1
11.2	9.8
12.8	11.2
12.7	11.2
	12
	10.6
	10.6
	11.9
	12.7
	12.9
