# Supplementary material for: Understanding the aliya pulsed electric field dose-response relationship: Implications for ablation size, thermal load, and immune response in an orthotopic murine breast cancer model
Source: PLoS One. 2025 Feb 13;20(2):e0318440. doi: 10.1371/journal.pone.0318440 (PMC11824980; doi:10.1371/journal.pone.0318440)
Supplement: S1 Raw data — (ZIP) [file pone.0318440.s005.zip › Fig 4 raw data.pdf]

Figure 4A raw data

Time, s	5mm	10mm	2.5mm
0.057142	0.15	0.36	0.04
0.114286	0.12	0.36	0.05
0.171428	0.1	0.36	0.06
0.228572	0.12	0.33	0.1
0.285714	0.11	0.34	0.1
0.342858	0.12	0.31	0.08
0.4	0.09	0.3	0.11
0.457142	0.07	0.32	0.13
0.514286	0.07	0.32	0.18
0.571428	0.06	0.35	0.2
0.628572	0.09	0.35	0.25
0.685714	0.08	0.33	0.29
0.742858	0.1	0.36	0.36
0.8	0.09	0.38	0.39
0.857142	0.09	0.39	0.47
0.914286	0.07	0.43	0.56
0.971428	0.08	0.43	0.64
1.028572	0.07	0.39	0.67
1.085714	0.06	0.4	0.72
1.142858	0.04	0.42	0.77
1.2	0.01	0.45	0.83
1.257142	0.03	0.47	0.89
1.314286	0.04	0.45	0.95
1.371428	0.07	0.46	0.96
1.428572	0.08	0.44	0.98
1.485714	0.07	0.42	1.01
1.542858	0.06	0.45	1.02
1.6	0.07	0.49	1.03
1.657142	0.07	0.51	1.04
1.714286	0.07	0.5	1.05
1.771428	0.08	0.52	1.03
1.828572	0.11	0.5	1
1.885714	0.11	0.51	0.99
1.942858	0.09	0.49	1.01
2	0.09	0.49	1.01
2.057142	0.12	0.5	1.02
2.114286	0.13	0.45	1.03
2.171428	0.13	0.43	1.05
2.228572	0.11	0.42	1.05
2.285714	0.12	0.42	1.09
2.342858	0.12	0.41	1.12
2.4	0.1	0.43	1.15
2.457142	0.11	0.41	1.2
2.514286	0.12	0.41	1.24

2.571428	0.12	0.41	1.31
2.628572	0.09	0.4	1.38
2.685714	0.11	0.44	1.45
2.742858	0.09	0.45	1.53
2.8	0.13	0.46	1.62
2.857142	0.13	0.47	1.69
2.914286	0.13	0.46	1.74
2.971428	0.13	0.46	1.82
3.028572	0.15	0.47	1.88
3.085714	0.16	0.48	1.93
3.142858	0.17	0.47	2
3.2	0.16	0.47	2.06
3.257142	0.15	0.45	2.11
3.314286	0.19	0.44	2.15
3.371428	0.18	0.44	2.19
3.428572	0.19	0.43	2.21
3.485714	0.21	0.44	2.22
3.542858	0.21	0.42	2.22
3.6	0.22	0.41	2.23
3.657142	0.19	0.41	2.26
3.714286	0.18	0.41	2.27
3.771428	0.22	0.37	2.29
3.828572	0.25	0.35	2.3
3.885714	0.25	0.35	2.3
3.942858	0.26	0.33	2.3
4	0.26	0.34	2.3
4.057142	0.25	0.34	2.3
4.114286	0.25	0.34	2.3
4.171428	0.22	0.33	2.32
4.228572	0.24	0.33	2.34
4.285714	0.26	0.35	2.38
4.342858	0.2	0.4	2.41
4.4	0.2	0.4	2.45
4.457142	0.21	0.37	2.53
4.514286	0.18	0.4	2.61
4.571428	0.2	0.37	2.69
4.628572	0.21	0.34	2.75
4.685714	0.21	0.35	2.81
4.742858	0.25	0.37	2.9
4.8	0.26	0.35	2.96
4.857142	0.27	0.33	3.03
4.914286	0.25	0.35	3.11
4.971428	0.26	0.37	3.16
5.028572	0.23	0.4	3.23
5.085714	0.25	0.39	3.28
5.142858	0.22	0.39	3.34
5.2	0.21	0.4	3.4

5.257142	0.23	0.36	3.46
5.314286	0.21	0.37	3.49
5.371428	0.19	0.41	3.53
5.428572	0.18	0.4	3.55
5.485714	0.24	0.35	3.55
5.542858	0.22	0.34	3.58
5.6	0.2	0.35	3.59
5.657142	0.24	0.32	3.6
5.714286	0.25	0.3	3.6
5.771428	0.27	0.31	3.66
5.828572	0.25	0.38	3.68
5.885714	0.27	0.39	3.71
5.942858	0.26	0.38	3.72
6	0.27	0.38	3.76
6.057142	0.28	0.38	3.76
6.114286	0.3	0.38	3.74
6.171428	0.31	0.37	3.77
6.228572	0.29	0.37	3.75
6.285714	0.27	0.42	3.77
6.342858	0.25	0.43	3.81
6.4	0.28	0.41	3.86
6.457142	0.29	0.39	3.89
6.514286	0.3	0.39	3.96
6.571428	0.29	0.39	4.02
6.628572	0.27	0.39	4.09
6.685714	0.25	0.4	4.12
6.742858	0.29	0.4	4.19
6.8	0.3	0.41	4.25
6.857142	0.3	0.4	4.3
6.914286	0.3	0.43	4.36
6.971428	0.26	0.44	4.44
7.028572	0.27	0.43	4.52
7.085714	0.29	0.46	4.57
7.142858	0.25	0.48	4.63
7.2	0.27	0.49	4.66
7.257142	0.29	0.49	4.69
7.314286	0.26	0.5	4.7
7.371428	0.25	0.49	4.72
7.428572	0.27	0.49	4.74
7.485714	0.29	0.44	4.76
7.542858	0.29	0.44	4.76
7.6	0.28	0.46	4.79
7.657142	0.23	0.44	4.81
7.714286	0.27	0.43	4.81
7.771428	0.25	0.44	4.82
7.828572	0.26	0.45	4.82
7.885714	0.27	0.45	4.83

7.942858	0.29	0.44	4.84
8	0.3	0.45	4.85
8.057142	0.29	0.45	4.86
8.114286	0.28	0.45	4.87
8.171428	0.29	0.44	4.88
8.228572	0.31	0.45	4.91
8.285714	0.32	0.45	4.95
8.342858	0.32	0.44	4.96
8.4	0.34	0.44	5.01
8.457142	0.31	0.44	5.08
8.514286	0.29	0.44	5.13
8.571428	0.28	0.43	5.18
8.628572	0.3	0.45	5.24
8.685714	0.33	0.43	5.31
8.742858	0.33	0.45	5.36
8.8	0.31	0.44	5.4
8.857142	0.3	0.45	5.48
8.914286	0.31	0.45	5.53
8.971428	0.29	0.44	5.62
9.028572	0.28	0.46	5.67
9.085714	0.28	0.46	5.71
9.142858	0.24	0.5	5.74
9.2	0.24	0.52	5.79
9.257142	0.24	0.52	5.8
9.314286	0.27	0.51	5.83
9.371428	0.3	0.49	5.84
9.428572	0.3	0.48	5.84
9.485714	0.27	0.48	5.84
9.542858	0.29	0.49	5.86
9.6	0.33	0.47	5.86
9.657142	0.33	0.48	5.86
9.714286	0.38	0.45	5.87
9.771428	0.37	0.4	5.87
9.828572	0.38	0.42	5.88
9.885714	0.35	0.41	5.89
9.942858	0.32	0.45	5.88
10	0.31	0.44	5.9
10.05714	0.35	0.43	5.91
10.11429	0.33	0.43	5.91
10.17143	0.32	0.44	5.93
10.22857	0.33	0.43	5.94
10.28571	0.31	0.42	5.97
10.34286	0.29	0.46	6.02
10.4	0.28	0.44	6.07
10.45714	0.31	0.43	6.11
10.51429	0.33	0.4	6.17
10.57143	0.38	0.4	6.21

10.62857	0.4	0.38	6.27
10.68571	0.42	0.36	6.34
10.74286	0.44	0.35	6.4
10.8	0.45	0.35	6.45
10.85714	0.48	0.36	6.5
10.91429	0.49	0.35	6.55
10.97143	0.51	0.37	6.63
11.02857	0.5	0.36	6.69
11.08571	0.5	0.37	6.75
11.14286	0.47	0.39	6.78
11.2	0.48	0.4	6.8
11.25714	0.44	0.41	6.79
11.31429	0.41	0.41	6.8
11.37143	0.41	0.42	6.83
11.42857	0.39	0.42	6.82
11.48571	0.38	0.43	6.84
11.54286	0.39	0.41	6.83
11.6	0.39	0.43	6.86
11.65714	0.39	0.46	6.85
11.71429	0.37	0.45	6.84
11.77143	0.36	0.45	6.85
11.82857	0.38	0.46	6.86
11.88571	0.38	0.48	6.86
11.94286	0.39	0.48	6.88
12	0.39	0.49	6.89
12.05714	0.42	0.46	6.92
12.11429	0.43	0.47	6.95
12.17143	0.41	0.5	6.98
12.22857	0.42	0.51	7.02
12.28571	0.45	0.5	7.06
12.34286	0.45	0.49	7.13
12.4	0.44	0.48	7.2
12.45714	0.47	0.47	7.24
12.51429	0.46	0.47	7.26
12.57143	0.46	0.45	7.33
12.62857	0.47	0.48	7.38
12.68571	0.46	0.48	7.43
12.74286	0.48	0.47	7.48
12.8	0.49	0.45	7.53
12.85714	0.51	0.46	7.57
12.91429	0.5	0.46	7.61
12.97143	0.49	0.46	7.64
13.02857	0.5	0.45	7.67
13.08571	0.54	0.44	7.7
13.14286	0.55	0.46	7.73
13.2	0.53	0.44	7.72
13.25714	0.54	0.44	7.71

13.31429	0.55	0.43	7.71
13.37143	0.55	0.43	7.71
13.42857	0.52	0.44	7.71
13.48571	0.52	0.45	7.69
13.54286	0.53	0.47	7.7
13.6	0.51	0.46	7.7
13.65714	0.48	0.48	7.7
13.71429	0.48	0.49	7.69
13.77143	0.48	0.51	7.69
13.82857	0.48	0.51	7.72
13.88571	0.45	0.53	7.75
13.94286	0.45	0.54	7.76
14	0.47	0.53	7.77
14.05714	0.5	0.55	7.75
14.11429	0.5	0.54	7.77
14.17143	0.52	0.58	7.78
14.22857	0.54	0.54	7.82
14.28571	0.55	0.53	7.88
14.34286	0.55	0.5	7.92
14.4	0.53	0.5	7.99
14.45714	0.52	0.5	8.05
14.51429	0.54	0.49	8.11
14.57143	0.52	0.5	8.18
14.62857	0.49	0.49	8.25
14.68571	0.49	0.49	8.31
14.74286	0.52	0.45	8.33
14.8	0.49	0.47	8.36
14.85714	0.5	0.48	8.41
14.91429	0.5	0.51	8.45
14.97143	0.52	0.49	8.52
15.02857	0.53	0.48	8.59
15.08571	0.5	0.5	8.61
15.14286	0.51	0.49	8.62
15.2	0.53	0.49	8.63
15.25714	0.53	0.5	8.64
15.31429	0.52	0.55	8.64
15.37143	0.51	0.56	8.64
15.42857	0.55	0.55	8.62
15.48571	0.57	0.54	8.61
15.54286	0.57	0.54	8.63
15.6	0.6	0.54	8.63
15.65714	0.62	0.54	8.64
15.71429	0.63	0.53	8.65
15.77143	0.65	0.52	8.66
15.82857	0.66	0.53	8.65
15.88571	0.67	0.5	8.64
15.94286	0.68	0.48	8.67

16	0.66	0.49	8.68
16.05714	0.67	0.49	8.68
16.11429	0.69	0.5	8.69
16.17143	0.68	0.51	8.74
16.22857	0.68	0.49	8.77
16.28571	0.69	0.51	8.81
16.34286	0.69	0.53	8.85
16.4	0.68	0.49	8.89
16.45714	0.69	0.52	8.92
16.51429	0.72	0.51	8.97
16.57143	0.73	0.5	9.02
16.62857	0.7	0.51	9.04
16.68571	0.67	0.52	9.08
16.74286	0.68	0.52	9.13
16.8	0.69	0.51	9.18
16.85714	0.71	0.49	9.2
16.91429	0.71	0.45	9.22
16.97143	0.69	0.47	9.26
17.02857	0.7	0.45	9.31
17.08571	0.68	0.45	9.33
17.14286	0.69	0.44	9.34
17.2	0.69	0.46	9.35
17.25714	0.69	0.49	9.36
17.31429	0.69	0.47	9.33
17.37143	0.68	0.5	9.33
17.42857	0.65	0.5	9.31
17.48571	0.66	0.53	9.3
17.54286	0.69	0.51	9.29
17.6	0.67	0.53	9.33
17.65714	0.69	0.54	9.33
17.71429	0.7	0.55	9.33
17.77143	0.69	0.56	9.32
17.82857	0.71	0.53	9.35
17.88571	0.72	0.56	9.36
17.94286	0.75	0.55	9.36
18	0.78	0.57	9.36
18.05714	0.78	0.56	9.38
18.11429	0.79	0.56	9.4
18.17143	0.79	0.55	9.44
18.22857	0.79	0.56	9.51
18.28571	0.74	0.58	9.55
18.34286	0.74	0.57	9.61
18.4	0.77	0.54	9.67
18.45714	0.75	0.53	9.71
18.51429	0.75	0.52	9.72
18.57143	0.76	0.48	9.77
18.62857	0.78	0.48	9.82

18.68571	0.79	0.46	9.86
18.74286	0.84	0.46	9.92
18.8	0.82	0.47	9.96
18.85714	0.86	0.46	9.98
18.91429	0.89	0.43	10.01
18.97143	0.87	0.45	10.05
19.02857	0.9	0.46	10.08
19.08571	0.89	0.45	10.09
19.14286	0.88	0.48	10.09
19.2	0.86	0.49	10.08
19.25714	0.85	0.5	10.07
19.31429	0.84	0.51	10.07
19.37143	0.85	0.5	10.08
19.42857	0.85	0.49	10.09
19.48571	0.82	0.51	10.07
19.54286	0.85	0.52	10.08
19.6	0.87	0.48	10.1
19.65714	0.91	0.49	10.1
19.71429	0.91	0.51	10.09
19.77143	0.91	0.51	10.09
19.82857	0.94	0.51	10.09
19.88571	0.92	0.49	10.1
19.94286	0.94	0.5	10.07
20	0.93	0.47	10.06
20.05714	0.93	0.46	10.05
20.11428	0.92	0.45	10.08
20.17142	0.91	0.49	10.12
20.22858	0.92	0.5	10.16
20.28572	0.91	0.49	10.19
20.34286	0.9	0.48	10.23
20.4	0.87	0.49	10.27
20.45714	0.88	0.5	10.32
20.51428	0.89	0.49	10.36
20.57142	0.89	0.5	10.39
20.62858	0.92	0.49	10.42
20.68572	0.96	0.48	10.46
20.74286	0.98	0.47	10.5
20.8	0.95	0.45	10.52
20.85714	0.95	0.46	10.56
20.91428	1	0.44	10.6
20.97142	1.01	0.47	10.64
21.02858	1.01	0.46	10.69
21.08572	1	0.46	10.69
21.14286	0.99	0.46	10.69
21.2	0.99	0.48	10.69
21.25714	0.96	0.53	10.69
21.31428	0.94	0.53	10.71

21.37142	0.96	0.56	10.69
21.42858	0.96	0.56	10.67
21.48572	0.95	0.6	10.67
21.54286	0.95	0.58	10.67
21.6	0.96	0.58	10.69
21.65714	0.96	0.59	10.69
21.71428	0.99	0.57	10.7
21.77142	1.01	0.58	10.7
21.82858	1.04	0.56	10.71
21.88572	1.04	0.53	10.69
21.94286	1.03	0.51	10.66
22	1.04	0.49	10.67
22.05714	1.04	0.48	10.68
22.11428	1.06	0.46	10.71
22.17142	1.07	0.44	10.75
22.22858	1.07	0.45	10.8
22.28572	1.06	0.48	10.82
22.34286	1.05	0.48	10.85
22.4	1.03	0.49	10.93
22.45714	1.02	0.52	10.98
22.51428	1.06	0.53	11.02
22.57142	1.05	0.53	11.04
22.62858	1.03	0.57	11.09
22.68572	1.02	0.57	11.12
22.74286	1	0.6	11.16
22.8	1.02	0.58	11.23
22.85714	1.04	0.57	11.29
22.91428	1.07	0.57	11.33
22.97142	1.07	0.55	11.35
23.02858	1.09	0.52	11.36
23.08572	1.08	0.53	11.36
23.14286	1.06	0.53	11.37
23.2	1.07	0.49	11.37
23.25714	1.08	0.49	11.36
23.31428	1.1	0.48	11.34
23.37142	1.12	0.49	11.3
23.42858	1.09	0.52	11.3
23.48572	1.06	0.52	11.29
23.54286	1.1	0.52	11.25
23.6	1.09	0.52	11.24
23.65714	1.11	0.53	11.23
23.71428	1.12	0.53	11.21
23.77142	1.17	0.54	11.22
23.82858	1.18	0.54	11.2
23.88572	1.18	0.52	11.18
23.94286	1.17	0.49	11.17
24	1.18	0.47	11.19

24.05714	1.23	0.46	11.21
24.11428	1.23	0.47	11.24
24.17142	1.24	0.5	11.28
24.22858	1.25	0.47	11.31
24.28572	1.26	0.46	11.37
24.34286	1.28	0.47	11.4
24.4	1.25	0.46	11.45
24.45714	1.26	0.48	11.48
24.51428	1.25	0.5	11.52
24.57142	1.26	0.51	11.57
24.62858	1.25	0.49	11.6
24.68572	1.23	0.48	11.63
24.74286	1.22	0.49	11.67
24.8	1.21	0.52	11.71
24.85714	1.21	0.54	11.74
24.91428	1.19	0.54	11.77
24.97142	1.2	0.56	11.76
25.02858	1.22	0.55	11.77
25.08572	1.25	0.52	11.75
25.14286	1.29	0.51	11.75
25.2	1.27	0.53	11.74
25.25714	1.29	0.57	11.73
25.31428	1.28	0.56	11.71
25.37142	1.28	0.54	11.72
25.42858	1.28	0.5	11.75
25.48572	1.27	0.51	11.74
25.54286	1.27	0.49	11.76
25.6	1.26	0.52	11.78
25.65714	1.23	0.57	11.78
25.71428	1.2	0.57	11.77
25.77142	1.21	0.56	11.77
25.82858	1.19	0.56	11.78
25.88572	1.19	0.56	11.76
25.94286	1.22	0.55	11.76
26	1.25	0.56	11.82
26.05714	1.25	0.56	11.87
26.11428	1.23	0.57	11.9
26.17142	1.23	0.59	11.96
26.22858	1.22	0.56	12.01
26.28572	1.23	0.6	12.04
26.34286	1.22	0.6	12.07
26.4	1.27	0.58	12.1
26.45714	1.28	0.56	12.14
26.51428	1.29	0.58	12.15
26.57142	1.28	0.59	12.18
26.62858	1.28	0.58	12.23
26.68572	1.32	0.58	12.28

26.74286	1.33	0.55	12.32
26.8	1.35	0.57	12.37
26.85714	1.36	0.54	12.39
26.91428	1.38	0.55	12.38
26.97142	1.37	0.56	12.38
27.02858	1.36	0.56	12.37
27.08572	1.36	0.59	12.35
27.14286	1.34	0.6	12.36
27.2	1.35	0.6	12.35
27.25714	1.37	0.59	12.34
27.31428	1.41	0.58	12.37
27.37142	1.43	0.54	12.35
27.42858	1.45	0.54	12.35
27.48572	1.45	0.53	12.35
27.54286	1.4	0.53	12.35
27.6	1.4	0.53	12.33
27.65714	1.38	0.52	12.31
27.71428	1.39	0.53	12.31
27.77142	1.41	0.52	12.3
27.82858	1.42	0.52	12.29
27.88572	1.41	0.53	12.29
27.94286	1.41	0.54	12.31
28	1.39	0.56	12.32
28.05714	1.39	0.56	12.33
28.11428	1.44	0.56	12.37
28.17142	1.44	0.58	12.4
28.22858	1.46	0.56	12.43
28.28572	1.46	0.57	12.49
28.34286	1.47	0.58	12.54
28.4	1.5	0.57	12.57
28.45714	1.49	0.59	12.62
28.51428	1.46	0.59	12.67
28.57142	1.48	0.59	12.7
28.62858	1.51	0.57	12.73
28.68572	1.5	0.55	12.77
28.74286	1.53	0.54	12.79
28.8	1.52	0.55	12.81
28.85714	1.54	0.54	12.82
28.91428	1.54	0.5	12.84
28.97142	1.51	0.48	12.86
29.02858	1.53	0.45	12.86
29.08572	1.55	0.44	12.86
29.14286	1.55	0.44	12.85
29.2	1.53	0.43	12.83
29.25714	1.55	0.43	12.81
29.31428	1.54	0.44	12.79
29.37142	1.55	0.43	12.76

29.42858	1.56	0.44	12.75
29.48572	1.59	0.44	12.74
29.54286	1.59	0.49	12.73
29.6	1.55	0.51	12.75
29.65714	1.55	0.5	12.74
29.71428	1.58	0.5	12.73
29.77142	1.6	0.51	12.73
29.82858	1.6	0.53	12.71
29.88572	1.61	0.52	12.71
29.94286	1.59	0.54	12.74
30	1.6	0.5	12.77
30.05714	1.57	0.56	12.78
30.11428	1.55	0.56	12.79
30.17142	1.58	0.56	12.82
30.22858	1.6	0.56	12.87
30.28572	1.6	0.57	12.91
30.34286	1.58	0.57	12.95
30.4	1.6	0.57	12.98
30.45714	1.58	0.59	13
30.51428	1.61	0.59	13.03
30.57142	1.63	0.63	13.08
30.62858	1.65	0.6	13.11
30.68572	1.68	0.59	13.15
30.74286	1.67	0.59	13.2
30.8	1.68	0.6	13.21
30.85714	1.65	0.6	13.22
30.91428	1.66	0.59	13.21
30.97142	1.66	0.58	13.24
31.02858	1.68	0.57	13.23
31.08572	1.66	0.55	13.24
31.14286	1.64	0.54	13.21
31.2	1.64	0.53	13.21
31.25714	1.64	0.52	13.21
31.31428	1.67	0.52	13.22
31.37142	1.64	0.52	13.22
31.42858	1.64	0.51	13.23
31.48572	1.65	0.55	13.21
31.54286	1.68	0.54	13.22
31.6	1.69	0.52	13.19
31.65714	1.71	0.52	13.16
31.71428	1.73	0.51	13.15
31.77142	1.74	0.49	13.14
31.82858	1.74	0.54	13.12
31.88572	1.73	0.53	13.12
31.94286	1.76	0.53	13.17
32	1.79	0.52	13.21
32.05714	1.79	0.52	13.25

32.11428	1.79	0.55	13.29
32.17142	1.8	0.52	13.32
32.22858	1.78	0.54	13.35
32.28572	1.78	0.57	13.4
32.34286	1.78	0.58	13.41
32.4	1.81	0.54	13.45
32.45714	1.83	0.55	13.5
32.51428	1.84	0.55	13.54
32.57142	1.85	0.56	13.6
32.62858	1.84	0.54	13.64
32.68572	1.8	0.55	13.67
32.74286	1.81	0.57	13.71
32.8	1.83	0.55	13.73
32.85714	1.85	0.52	13.73
32.91428	1.81	0.53	13.69
32.97142	1.8	0.55	13.67
33.02858	1.79	0.55	13.64
33.08572	1.77	0.57	13.63
33.14286	1.76	0.57	13.62
33.2	1.78	0.57	13.61
33.25714	1.81	0.55	13.6
33.31428	1.79	0.58	13.59
33.37142	1.78	0.6	13.56
33.42858	1.78	0.6	13.52
33.48572	1.81	0.61	13.5
33.54286	1.84	0.59	13.51
33.6	1.84	0.59	13.51
33.65714	1.85	0.61	13.52
33.71428	1.86	0.62	13.5
33.77142	1.87	0.64	13.49
33.82858	1.87	0.66	13.52
33.88572	1.89	0.66	13.55
33.94286	1.9	0.65	13.58
34	1.91	0.65	13.61
34.05714	1.88	0.64	13.64
34.11428	1.92	0.6	13.66
34.17142	1.94	0.59	13.69
34.22858	1.93	0.57	13.72
34.28572	1.95	0.54	13.77
34.34286	1.96	0.53	13.83
34.4	1.98	0.52	13.87
34.45714	1.98	0.49	13.9
34.51428	1.97	0.51	13.93
34.57142	1.96	0.5	13.95
34.62858	1.96	0.53	14.01
34.68572	1.95	0.55	14.05
34.74286	1.91	0.56	14.06

34.8	1.92	0.55	14.08
34.85714	1.91	0.55	14.1
34.91428	1.92	0.57	14.09
34.97142	1.89	0.57	14.08
35.02858	1.89	0.57	14.08
35.08572	1.91	0.55	14.06
35.14286	1.92	0.56	14.08
35.2	1.94	0.53	14.06
35.25714	1.93	0.54	14.06
35.31428	1.95	0.53	14.04
35.37142	1.98	0.54	14.05
35.42858	1.97	0.59	14.03
35.48572	1.96	0.6	14.04
35.54286	1.96	0.61	14.02
35.6	1.97	0.62	14
35.65714	1.97	0.65	13.99
35.71428	1.99	0.62	13.98
35.77142	1.96	0.63	13.98
35.82858	1.99	0.65	13.99
35.88572	1.98	0.64	14
35.94286	1.98	0.64	14.04
36	1.98	0.6	14.09
36.05714	1.98	0.6	14.11
36.11428	2	0.57	14.15
36.17142	2.04	0.52	14.17
36.22858	2.05	0.51	14.2
36.28572	2.06	0.53	14.21
36.34286	2.1	0.5	14.24
36.4	2.08	0.5	14.27
36.45714	2.08	0.52	14.29
36.51428	2.09	0.51	14.33
36.57142	2.12	0.53	14.37
36.62858	2.15	0.49	14.39
36.68572	2.17	0.52	14.4
36.74286	2.2	0.55	14.41
36.8	2.19	0.56	14.43
36.85714	2.17	0.57	14.41
36.91428	2.15	0.58	14.41
36.97142	2.18	0.6	14.38
37.02858	2.18	0.57	14.38
37.08572	2.19	0.58	14.38
37.14286	2.14	0.59	14.39
37.2	2.15	0.65	14.4
37.25714	2.12	0.64	14.41
37.31428	2.11	0.65	14.41
37.37142	2.11	0.65	14.41
37.42858	2.12	0.64	14.4

37.48572	2.1	0.66	14.4
37.54286	2.11	0.65	14.38
37.6	2.12	0.67	14.38
37.65714	2.13	0.66	14.37
37.71428	2.15	0.69	14.36
37.77142	2.16	0.66	14.38
37.82858	2.21	0.64	14.41
37.88572	2.2	0.65	14.45
37.94286	2.19	0.64	14.47
38	2.21	0.63	14.46
38.05714	2.26	0.58	14.49
38.11428	2.26	0.58	14.51
38.17142	2.23	0.6	14.53
38.22858	2.22	0.62	14.55
38.28572	2.24	0.58	14.58
38.34286	2.22	0.6	14.61
38.4	2.19	0.63	14.63
38.45714	2.16	0.61	14.68
38.51428	2.22	0.62	14.71
38.57142	2.22	0.63	14.74
38.62858	2.22	0.67	14.78
38.68572	2.24	0.67	14.79
38.74286	2.27	0.65	14.79
38.8	2.29	0.64	14.78
38.85714	2.3	0.64	14.79
38.91428	2.3	0.65	14.81
38.97142	2.33	0.62	14.78
39.02858	2.4	0.59	14.74
39.08572	2.35	0.62	14.72
39.14286	2.35	0.61	14.69
39.2	2.35	0.6	14.68
39.25714	2.31	0.58	14.66
39.31428	2.31	0.59	14.66
39.37142	2.31	0.59	14.63
39.42858	2.29	0.59	14.62
39.48572	2.3	0.54	14.59
39.54286	2.3	0.55	14.58
39.6	2.25	0.58	14.56
39.65714	2.27	0.59	14.55
39.71428	2.27	0.63	14.54
39.77142	2.28	0.63	14.53
39.82858	2.3	0.69	14.56
39.88572	2.28	0.7	14.6
39.94286	2.28	0.69	14.67
40	2.31	0.72	14.71
40.05714	2.31	0.74	14.76
40.11428	2.32	0.73	14.79

40.17142	2.32	0.73	14.85
40.22858	2.33	0.7	14.88
40.28572	2.33	0.68	14.93
40.34286	2.34	0.69	14.98
40.4	2.31	0.69	15.02
40.45714	2.35	0.67	15.07
40.51428	2.35	0.69	15.1
40.57142	2.34	0.7	15.13
40.62858	2.34	0.7	15.15
40.68572	2.38	0.71	15.17
40.74286	2.38	0.72	15.16
40.8	2.36	0.72	15.14
40.85714	2.37	0.72	15.11
40.91428	2.38	0.73	15.09
40.97142	2.38	0.73	15.07
41.02858	2.39	0.68	15.04
41.08572	2.38	0.71	15.03
41.14286	2.38	0.71	15
41.2	2.4	0.69	14.98
41.25714	2.38	0.69	14.98
41.31428	2.38	0.68	14.96
41.37142	2.39	0.7	14.95
41.42858	2.41	0.7	14.94
41.48572	2.42	0.69	14.94
41.54286	2.46	0.67	14.94
41.6	2.45	0.72	14.92
41.65714	2.47	0.66	14.94
41.71428	2.46	0.67	14.97
41.77142	2.48	0.69	15
41.82858	2.5	0.67	15.04
41.88572	2.5	0.67	15.08
41.94286	2.5	0.66	15.14
42	2.5	0.66	15.17
42.05714	2.48	0.65	15.18
42.11428	2.48	0.65	15.21
42.17142	2.49	0.62	15.23
42.22858	2.5	0.66	15.28
42.28572	2.5	0.65	15.29
42.34286	2.48	0.64	15.34
42.4	2.48	0.67	15.38
42.45714	2.5	0.64	15.39
42.51428	2.52	0.64	15.45
42.57142	2.52	0.62	15.48
42.62858	2.55	0.66	15.48
42.68572	2.52	0.68	15.5
42.74286	2.52	0.7	15.5
42.8	2.52	0.69	15.49

42.85714	2.57	0.66	15.49
42.91428	2.56	0.66	15.48
42.97142	2.54	0.67	15.48
43.02858	2.53	0.71	15.47
43.08572	2.53	0.72	15.46
43.14286	2.56	0.73	15.42
43.2	2.55	0.71	15.4
43.25714	2.58	0.68	15.38
43.31428	2.58	0.66	15.35
43.37142	2.56	0.68	15.35
43.42858	2.57	0.7	15.32
43.48572	2.58	0.7	15.29
43.54286	2.59	0.69	15.24
43.6	2.62	0.64	15.22
43.65714	2.61	0.63	15.22
43.71428	2.6	0.62	15.27
43.77142	2.64	0.6	15.28
43.82858	2.64	0.61	15.3
43.88572	2.64	0.59	15.32
43.94286	2.65	0.61	15.35
44	2.65	0.6	15.39
44.05714	2.63	0.59	15.44
44.11428	2.63	0.58	15.47
44.17142	2.62	0.62	15.52
44.22858	2.63	0.62	15.56
44.28572	2.63	0.66	15.6
44.34286	2.61	0.68	15.62
44.4	2.61	0.67	15.68
44.45714	2.63	0.7	15.74
44.51428	2.65	0.66	15.76
44.57142	2.68	0.63	15.8
44.62858	2.72	0.63	15.8
44.68572	2.73	0.63	15.78
44.74286	2.71	0.61	15.78
44.8	2.72	0.63	15.76
44.85714	2.72	0.61	15.77
44.91428	2.75	0.6	15.77
44.97142	2.76	0.59	15.75
45.02858	2.75	0.59	15.73
45.08572	2.74	0.61	15.71
45.14286	2.69	0.64	15.7
45.2	2.69	0.65	15.66
45.25714	2.69	0.65	15.64
45.31428	2.71	0.68	15.6
45.37142	2.71	0.68	15.61
45.42858	2.7	0.69	15.61
45.48572	2.66	0.71	15.59

45.54286	2.66	0.75	15.55
45.6	2.66	0.73	15.53
45.65714	2.68	0.73	15.55
45.71428	2.69	0.74	15.6
45.77142	2.71	0.72	15.64
45.82858	2.72	0.73	15.64
45.88572	2.69	0.74	15.68
45.94286	2.7	0.76	15.72
46	2.72	0.77	15.75
46.05714	2.76	0.79	15.79
46.11428	2.79	0.76	15.82
46.17142	2.8	0.8	15.87
46.22858	2.8	0.79	15.9
46.28572	2.82	0.79	15.92
46.34286	2.82	0.8	15.94
46.4	2.83	0.78	15.98
46.45714	2.84	0.8	16.02
46.51428	2.8	0.81	16.07
46.57142	2.81	0.79	16.06
46.62858	2.8	0.75	16.04
46.68572	2.8	0.74	16.02
46.74286	2.78	0.71	16
46.8	2.77	0.71	15.99
46.85714	2.77	0.71	15.99
46.91428	2.77	0.72	15.98
46.97142	2.74	0.75	15.97
47.02858	2.75	0.74	15.95
47.08572	2.79	0.69	15.94
47.14286	2.8	0.69	15.92
47.2	2.81	0.71	15.93
47.25714	2.8	0.74	15.94
47.31428	2.79	0.76	15.93
47.37142	2.78	0.79	15.9
47.42858	2.79	0.81	15.87
47.48572	2.79	0.79	15.85
47.54286	2.84	0.76	15.85
47.6	2.84	0.75	15.87
47.65714	2.83	0.76	15.88
47.71428	2.83	0.75	15.91
47.77142	2.82	0.73	15.94
47.82858	2.84	0.72	15.96
47.88572	2.86	0.71	15.98
47.94286	2.88	0.69	16.03
48	2.89	0.68	16.07
48.05714	2.9	0.68	16.1
48.11428	2.9	0.68	16.15
48.17142	2.88	0.7	16.17

48.22858	2.9	0.71	16.22
48.28572	2.91	0.71	16.26
48.34286	2.91	0.73	16.31
48.4	2.91	0.72	16.37
48.45714	2.89	0.74	16.42
48.51428	2.92	0.74	16.42
48.57142	2.9	0.77	16.45
48.62858	2.9	0.78	16.44
48.68572	2.9	0.79	16.43
48.74286	2.93	0.8	16.44
48.8	2.95	0.77	16.43
48.85714	2.93	0.78	16.42
48.91428	2.92	0.77	16.4
48.97142	2.93	0.77	16.39
49.02858	2.92	0.76	16.37
49.08572	2.9	0.77	16.33
49.14286	2.94	0.75	16.32
49.2	2.94	0.76	16.29
49.25714	2.97	0.75	16.29
49.31428	2.95	0.74	16.25
49.37142	2.93	0.75	16.21
49.42858	2.93	0.72	16.19
49.48572	2.96	0.72	16.18
49.54286	2.98	0.72	16.2
49.6	2.99	0.74	16.22
49.65714	3.03	0.74	16.22
49.71428	3.02	0.74	16.25
49.77142	3.04	0.74	16.26
49.82858	3.05	0.73	16.3
49.88572	3.07	0.73	16.33
49.94286	3.06	0.75	16.36
50	3.06	0.78	16.4
50.05714	3.05	0.79	16.43
50.11428	3.01	0.82	16.46
50.17142	3.01	0.82	16.45
50.22858	3.02	0.82	16.48
50.28572	3.04	0.81	16.54
50.34286	3.05	0.78	16.57
50.4	3	0.81	16.61
50.45714	2.98	0.83	16.62
50.51428	3	0.85	16.63
50.57142	3.01	0.86	16.62
50.62858	3	0.87	16.6
50.68572	3.02	0.86	16.58
50.74286	3.03	0.86	16.56
50.8	3.02	0.85	16.54
50.85714	3.01	0.84	16.52

50.91428	3.01	0.83	16.5
50.97142	3.05	0.82	16.47
51.02858	3.09	0.8	16.48
51.08572	3.09	0.76	16.47
51.14286	3.13	0.72	16.49
51.2	3.13	0.74	16.47
51.25714	3.14	0.74	16.47
51.31428	3.15	0.74	16.44
51.37142	3.19	0.73	16.42
51.42858	3.22	0.72	16.4
51.48572	3.18	0.76	16.41
51.54286	3.18	0.78	16.41
51.6	3.16	0.78	16.4
51.65714	3.18	0.77	16.43
51.71428	3.15	0.8	16.45
51.77142	3.17	0.79	16.49
51.82858	3.17	0.76	16.52
51.88572	3.19	0.77	16.57
51.94286	3.17	0.78	16.6
52	3.16	0.8	16.62
52.05714	3.18	0.8	16.62
52.11428	3.19	0.79	16.62
52.17142	3.19	0.8	16.64
52.22858	3.19	0.81	16.68
52.28572	3.2	0.81	16.72
52.34286	3.2	0.8	16.76
52.4	3.22	0.82	16.79
52.45714	3.23	0.81	16.81
52.51428	3.24	0.79	16.83
52.57142	3.24	0.8	16.83
52.62858	3.25	0.81	16.8
52.68572	3.25	0.81	16.79
52.74286	3.25	0.81	16.76
52.8	3.23	0.83	16.74
52.85714	3.23	0.83	16.73
52.91428	3.23	0.83	16.73
52.97142	3.24	0.8	16.72
53.02858	3.24	0.8	16.73
53.08572	3.21	0.82	16.72
53.14286	3.21	0.82	16.72
53.2	3.22	0.78	16.68
53.25714	3.22	0.77	16.66
53.31428	3.24	0.78	16.63
53.37142	3.25	0.77	16.61
53.42858	3.26	0.77	16.58
53.48572	3.29	0.77	16.59
53.54286	3.26	0.81	16.62

53.6	3.28	0.83	16.65
53.65714	3.29	0.81	16.68
53.71428	3.29	0.81	16.7
53.77142	3.3	0.83	16.73
53.82858	3.29	0.82	16.75
53.88572	3.29	0.83	16.78
53.94286	3.3	0.8	16.8
54	3.31	0.81	16.82
54.05714	3.32	0.79	16.84
54.11428	3.35	0.77	16.89
54.17142	3.35	0.76	16.92
54.22858	3.34	0.77	16.96
54.28572	3.39	0.77	16.98
54.34286	3.4	0.76	17.02
54.4	3.45	0.73	17.04
54.45714	3.44	0.74	17.05
54.51428	3.44	0.76	17.05
54.57142	3.44	0.75	17.04
54.62858	3.41	0.8	17.04
54.68572	3.38	0.83	17.04
54.74286	3.37	0.82	17.04
54.8	3.4	0.81	17.01
54.85714	3.39	0.8	16.99
54.91428	3.35	0.84	16.99
54.97142	3.32	0.85	16.98
55.02858	3.33	0.82	16.96
55.08572	3.35	0.84	16.93
55.14286	3.33	0.83	16.93
55.2	3.35	0.81	16.93
55.25714	3.36	0.78	16.92
55.31428	3.37	0.79	16.9
55.37142	3.33	0.81	16.86
55.42858	3.32	0.83	16.86
55.48572	3.33	0.84	16.86
55.54286	3.33	0.87	16.88
55.6	3.31	0.9	16.88
55.65714	3.34	0.89	16.9
55.71428	3.33	0.91	16.93
55.77142	3.32	0.89	16.95
55.82858	3.32	0.92	16.98
55.88572	3.33	0.93	17
55.94286	3.35	0.93	17.01
56	3.34	0.92	17.03
56.05714	3.34	0.92	17.03
56.11428	3.35	0.93	17.06
56.17142	3.36	0.93	17.08
56.22858	3.36	0.92	17.11

56.28572	3.36	0.94	17.14
56.34286	3.35	0.93	17.21
56.4	3.38	0.93	17.25
56.45714	3.41	0.91	17.25
56.51428	3.4	0.9	17.26
56.57142	3.42	0.86	17.26
56.62858	3.45	0.87	17.27
56.68572	3.47	0.84	17.26
56.74286	3.5	0.8	17.25
56.8	3.53	0.79	17.24
56.85714	3.54	0.76	17.24
56.91428	3.54	0.79	17.23
56.97142	3.54	0.79	17.22
57.02858	3.54	0.81	17.22
57.08572	3.56	0.8	17.19
57.14286	3.57	0.79	17.17
57.2	3.55	0.8	17.17
57.25714	3.54	0.82	17.16
57.31428	3.51	0.85	17.14
57.37142	3.52	0.85	17.12
57.42858	3.53	0.87	17.12
57.48572	3.51	0.86	17.13
57.54286	3.53	0.87	17.13
57.6	3.55	0.85	17.15
57.65714	3.55	0.87	17.17
57.71428	3.53	0.88	17.2
57.77142	3.56	0.88	17.26
57.82858	3.56	0.88	17.26
57.88572	3.54	0.88	17.29
57.94286	3.51	0.91	17.32
58	3.51	0.92	17.37
58.05714	3.54	0.9	17.4
58.11428	3.55	0.88	17.42
58.17142	3.54	0.9	17.44
58.22858	3.56	0.88	17.46
58.28572	3.57	0.91	17.48
58.34286	3.53	0.89	17.48
58.4	3.54	0.89	17.46
58.45714	3.55	0.89	17.46
58.51428	3.59	0.84	17.45
58.57142	3.6	0.85	17.44
58.62858	3.59	0.85	17.43
58.68572	3.57	0.87	17.4
58.74286	3.55	0.87	17.41
58.8	3.55	0.88	17.4
58.85714	3.57	0.86	17.36
58.91428	3.59	0.88	17.33

58.97142	3.59	0.87	17.31
59.02858	3.64	0.84	17.3
59.08572	3.64	0.86	17.28
59.14286	3.62	0.83	17.24
59.2	3.66	0.82	17.23
59.25714	3.64	0.82	17.22
59.31428	3.65	0.81	17.21
59.37142	3.67	0.81	17.22
59.42858	3.67	0.83	17.25
59.48572	3.69	0.83	17.26
59.54286	3.69	0.83	17.3
59.6	3.65	0.86	17.31
59.65714	3.66	0.87	17.32
59.71428	3.67	0.9	17.35
59.77142	3.64	0.92	17.39
59.82858	3.67	0.93	17.42
59.88572	3.69	0.92	17.45
59.94286	3.7	0.9	17.46
60	3.67	0.9	17.48
60.05714	3.67	0.91	17.51
60.11428	3.67	0.94	17.54
60.17142	3.69	0.92	17.59
60.22858	3.69	0.93	17.63
60.28572	3.68	0.94	17.65
60.34286	3.7	0.95	17.65
60.4	3.69	0.96	17.63
60.45714	3.7	0.98	17.62
60.51428	3.69	1.01	17.62
60.57142	3.71	1	17.63
60.62858	3.71	0.99	17.61
60.68572	3.72	0.97	17.61
60.74286	3.73	0.99	17.61
60.8	3.73	1	17.59
60.85714	3.74	0.96	17.6
60.91428	3.73	0.95	17.6
60.97142	3.72	0.95	17.58
61.02858	3.72	0.94	17.56
61.08572	3.7	0.97	17.57
61.14286	3.67	1	17.54
61.2	3.66	0.99	17.51
61.25714	3.66	0.97	17.51
61.31428	3.68	0.96	17.51
61.37142	3.71	0.94	17.53
61.42858	3.73	0.93	17.55
61.48572	3.76	0.93	17.56
61.54286	3.78	0.94	17.57
61.6	3.78	0.94	17.59

61.65714	3.78	0.95	17.61
61.71428	3.79	0.95	17.63
61.77142	3.79	0.94	17.67
61.82858	3.77	0.96	17.7
61.88572	3.76	0.96	17.72
61.94286	3.77	1	17.76
62	3.74	1.02	17.77
62.05714	3.72	1.04	17.8
62.11428	3.72	1.04	17.87
62.17142	3.72	1.05	17.9
62.22858	3.75	1.02	17.93
62.28572	3.78	0.97	17.93
62.34286	3.81	0.98	17.94
62.4	3.81	0.98	17.94
62.45714	3.81	1	17.93
62.51428	3.79	0.98	17.92
62.57142	3.79	0.98	17.93
62.62858	3.81	0.98	17.9
62.68572	3.81	0.95	17.88
62.74286	3.8	0.95	17.85
62.8	3.76	0.97	17.84
62.85714	3.75	1.01	17.83
62.91428	3.77	0.98	17.81
62.97142	3.8	0.97	17.8
63.02858	3.79	0.97	17.78
63.08572	3.78	0.98	17.75
63.14286	3.81	0.99	17.73
63.2	3.81	0.96	17.72
63.25714	3.85	0.97	17.7
63.31428	3.84	0.98	17.68
63.37142	3.87	0.99	17.71
63.42858	3.86	0.98	17.72
63.48572	3.86	1	17.73
63.54286	3.85	1.03	17.78
63.6	3.86	1.02	17.81
63.65714	3.87	1	17.84
63.71428	3.87	0.97	17.88
63.77142	3.86	1.01	17.9
63.82858	3.82	1.03	17.93
63.88572	3.86	1.02	17.96
63.94286	3.86	1.02	17.97
64	3.89	1.04	17.99
64.05714	3.88	1.05	18.03
64.11428	3.87	1.05	18.04
64.17142	3.9	1.05	18.06
64.22858	3.91	1.06	18.09
64.28572	3.93	1.07	18.11

64.34286	3.9	1.04	18.12
64.4	3.95	1.03	18.13
64.45714	3.92	1.01	18.09
64.51428	3.94	0.98	18.04
64.57142	3.92	0.97	18.04
64.62858	3.93	0.98	18.05
64.68572	3.94	0.95	18.03
64.74286	3.92	0.98	18.02
64.8	3.92	0.99	18
64.85714	3.9	1	18.02
64.91428	3.94	1	18.01
64.97142	3.92	1.01	18
65.02858	3.93	1.02	17.97
65.08572	3.95	1	17.95
65.14286	3.95	0.99	17.92
65.2	3.92	1	17.89
65.25714	3.9	1.01	17.84
65.31428	3.93	0.98	17.84
65.37142	3.92	0.99	17.87
65.42858	3.95	0.98	17.93
65.48572	3.92	0.99	17.95
65.54286	3.94	1.02	17.98
65.6	3.91	1.03	18
65.65714	3.91	1.07	18.03
65.71428	3.9	1.09	18.07
65.77142	3.93	1.08	18.08
65.82858	3.96	1.07	18.09
65.88572	3.96	1.08	18.1
65.94286	3.97	1.08	18.14
66	3.94	1.1	18.17
66.05714	3.95	1.11	18.21
66.11428	3.97	1.07	18.23
66.17142	4	1.06	18.28
66.22858	3.99	1.04	18.3
66.28572	3.98	1.04	18.28
66.34286	3.99	1.03	18.29
66.4	4.02	1.03	18.29
66.45714	4.01	1.03	18.27
66.51428	4.03	1.04	18.25
66.57142	4.05	1.03	18.24
66.62858	4.05	1.02	18.23
66.68572	4.06	1.03	18.22
66.74286	4.07	1.02	18.19
66.8	4.08	1.04	18.18
66.85714	4.09	1.04	18.18
66.91428	4.1	1.05	18.17
66.97142	4.05	1.05	18.15

67.02858	4.08	1.04	18.13
67.08572	4.06	1.05	18.14
67.14286	4.07	1.07	18.11
67.2	4.08	1.05	18.12
67.25714	4.07	1.05	18.11
67.31428	4.07	1.06	18.12
67.37142	4.08	1.05	18.13
67.42858	4.09	1.07	18.15
67.48572	4.1	1.09	18.17
67.54286	4.12	1.1	18.19
67.6	4.11	1.11	18.2
67.65714	4.12	1.11	18.25
67.71428	4.11	1.09	18.29
67.77142	4.13	1.09	18.32
67.82858	4.12	1.09	18.35
67.88572	4.14	1.1	18.37
67.94286	4.13	1.12	18.4
68	4.12	1.11	18.41
68.05714	4.12	1.08	18.45
68.11428	4.12	1.06	18.49
68.17142	4.11	1.06	18.51
68.22858	4.1	1.05	18.5
68.28572	4.08	1.06	18.5
68.34286	4.06	1.07	18.47
68.4	4.09	1.1	18.48
68.45714	4.07	1.12	18.47
68.51428	4.07	1.12	18.46
68.57142	4.04	1.12	18.45
68.62858	4.07	1.13	18.44
68.68572	4.07	1.13	18.44
68.74286	4.08	1.14	18.44
68.8	4.1	1.15	18.42
68.85714	4.12	1.13	18.4
68.91428	4.13	1.14	18.39
68.97142	4.1	1.12	18.36
69.02858	4.12	1.12	18.34
69.08572	4.1	1.13	18.32
69.14286	4.14	1.13	18.31
69.2	4.13	1.14	18.31
69.25714	4.12	1.16	18.31
69.31428	4.12	1.17	18.34
69.37142	4.13	1.18	18.35
69.42858	4.15	1.21	18.37
69.48572	4.16	1.21	18.39
69.54286	4.21	1.2	18.42
69.6	4.22	1.2	18.43
69.65714	4.24	1.19	18.43

69.71428	4.23	1.18	18.45
69.77142	4.23	1.19	18.47
69.82858	4.23	1.19	18.47
69.88572	4.26	1.19	18.5
69.94286	4.28	1.18	18.54
70	4.26	1.17	18.58
70.05714	4.26	1.15	18.58
70.11428	4.27	1.11	18.58
70.17142	4.26	1.12	18.62
70.22858	4.25	1.1	18.61
70.28572	4.24	1.09	18.62
70.34286	4.21	1.12	18.61
70.4	4.21	1.12	18.61
70.45714	4.22	1.11	18.6
70.51428	4.22	1.11	18.58
70.57142	4.2	1.13	18.55
70.62858	4.24	1.14	18.54
70.68572	4.24	1.15	18.53
70.74286	4.24	1.17	18.53
70.8	4.23	1.19	18.53
70.85714	4.21	1.21	18.5
70.91428	4.23	1.21	18.47
70.97142	4.23	1.19	18.44
71.02858	4.24	1.2	18.44
71.08572	4.23	1.2	18.45
71.14286	4.23	1.19	18.44
71.2	4.22	1.17	18.44
71.25714	4.22	1.15	18.44
71.31428	4.23	1.15	18.45
71.37142	4.2	1.17	18.48
71.42858	4.21	1.17	18.51
71.48572	4.19	1.22	18.55
71.54286	4.21	1.21	18.57
71.6	4.19	1.21	18.59
71.65714	4.22	1.19	18.62
71.71428	4.24	1.18	18.64
71.77142	4.25	1.2	18.68
71.82858	4.26	1.2	18.71
71.88572	4.23	1.22	18.73
71.94286	4.24	1.19	18.75
72	4.25	1.19	18.78
72.05714	4.26	1.17	18.82
72.11428	4.27	1.2	18.83
72.17142	4.29	1.2	18.82
72.22858	4.29	1.22	18.83
72.28572	4.27	1.22	18.84
72.34286	4.26	1.22	18.81

72.4	4.25	1.22	18.8
72.45714	4.29	1.21	18.8
72.51428	4.3	1.23	18.78
72.57142	4.31	1.22	18.76
72.62858	4.33	1.21	18.73
72.68572	4.35	1.19	18.71
72.74286	4.34	1.17	18.7
72.8	4.31	1.16	18.68
72.85714	4.34	1.16	18.66
72.91428	4.33	1.15	18.63
72.97142	4.33	1.16	18.59
73.02858	4.36	1.12	18.58
73.08572	4.36	1.13	18.58
73.14286	4.37	1.14	18.59
73.2	4.36	1.15	18.58
73.25714	4.37	1.17	18.58
73.31428	4.4	1.15	18.6
73.37142	4.43	1.15	18.6
73.42858	4.45	1.14	18.62
73.48572	4.5	1.14	18.65
73.54286	4.51	1.13	18.67
73.6	4.48	1.15	18.7
73.65714	4.48	1.12	18.71
73.71428	4.47	1.14	18.75
73.77142	4.47	1.13	18.82
73.82858	4.48	1.14	18.86
73.88572	4.46	1.15	18.9
73.94286	4.42	1.16	18.9
74	4.41	1.17	18.92
74.05714	4.36	1.19	18.95
74.11428	4.35	1.2	18.97
74.17142	4.36	1.22	18.96
74.22858	4.36	1.26	18.98
74.28572	4.36	1.26	18.99
74.34286	4.4	1.29	18.98
74.4	4.39	1.27	18.97
74.45714	4.38	1.28	18.95
74.51428	4.37	1.26	18.95
74.57142	4.39	1.26	18.96
74.62858	4.41	1.28	18.95
74.68572	4.41	1.27	18.92
74.74286	4.43	1.24	18.91
74.8	4.42	1.21	18.89
74.85714	4.46	1.18	18.88
74.91428	4.45	1.15	18.87
74.97142	4.48	1.16	18.84
75.02858	4.48	1.18	18.83

75.08572	4.51	1.19	18.83
75.14286	4.51	1.19	18.82
75.2	4.5	1.18	18.85
75.25714	4.52	1.22	18.87
75.31428	4.52	1.24	18.88
75.37142	4.55	1.25	18.9
75.42858	4.5	1.27	18.93
75.48572	4.5	1.29	18.95
75.54286	4.49	1.3	18.96
75.6	4.49	1.28	18.98
75.65714	4.5	1.27	18.98
75.71428	4.52	1.26	19
75.77142	4.54	1.27	19.02
75.82858	4.56	1.24	19.05
75.88572	4.52	1.26	19.08
75.94286	4.54	1.24	19.09
76	4.57	1.21	19.1
76.05714	4.57	1.21	19.13
76.11428	4.55	1.18	19.12
76.17142	4.56	1.19	19.11
76.22858	4.56	1.2	19.11
76.28572	4.56	1.21	19.1
76.34286	4.56	1.19	19.09
76.4	4.56	1.17	19.07
76.45714	4.57	1.16	19.05
76.51428	4.56	1.18	19.07
76.57142	4.54	1.19	19.05
76.62858	4.54	1.17	19.03
76.68572	4.57	1.19	19.01
76.74286	4.56	1.18	19
76.8	4.56	1.19	18.99
76.85714	4.58	1.19	18.96
76.91428	4.57	1.21	18.95
76.97142	4.56	1.22	18.89
77.02858	4.55	1.23	18.88
77.08572	4.56	1.23	18.88
77.14286	4.54	1.26	18.9
77.2	4.56	1.29	18.91
77.25714	4.54	1.3	18.93
77.31428	4.56	1.3	18.95
77.37142	4.56	1.28	18.99
77.42858	4.56	1.31	19
77.48572	4.58	1.28	19.02
77.54286	4.58	1.28	19.06
77.6	4.61	1.25	19.09
77.65714	4.62	1.26	19.12
77.71428	4.62	1.26	19.16

77.77142	4.61	1.27	19.17
77.82858	4.6	1.28	19.22
77.88572	4.64	1.3	19.27
77.94286	4.62	1.32	19.29
78	4.62	1.29	19.3
78.05714	4.63	1.31	19.29
78.11428	4.64	1.34	19.28
78.17142	4.64	1.34	19.29
78.22858	4.67	1.33	19.31
78.28572	4.69	1.31	19.3
78.34286	4.72	1.28	19.29
78.4	4.73	1.28	19.26
78.45714	4.7	1.26	19.23
78.51428	4.72	1.24	19.22
78.57142	4.74	1.26	19.2
78.62858	4.72	1.27	19.18
78.68572	4.69	1.27	19.18
78.74286	4.71	1.28	19.17
78.8	4.68	1.31	19.15
78.85714	4.69	1.31	19.13
78.91428	4.68	1.34	19.1
78.97142	4.65	1.34	19.08
79.02858	4.67	1.34	19.07
79.08572	4.66	1.35	19.07
79.14286	4.65	1.36	19.03
79.2	4.65	1.35	19.03
79.25714	4.7	1.31	19.04
79.31428	4.67	1.32	19.08
79.37142	4.65	1.31	19.12
79.42858	4.64	1.34	19.16
79.48572	4.64	1.34	19.2
79.54286	4.66	1.33	19.2
79.6	4.67	1.34	19.23
79.65714	4.66	1.33	19.27
79.71428	4.66	1.31	19.29
79.77142	4.66	1.32	19.33
79.82858	4.62	1.34	19.35
79.88572	4.62	1.34	19.38
79.94286	4.65	1.33	19.42
80	4.67	1.3	19.43
80.05714	4.66	1.3	19.46
80.11428	4.68	1.29	19.48
80.17142	4.68	1.29	19.46
80.22858	4.69	1.32	19.44
80.28572	4.68	1.35	19.43
80.34286	4.72	1.33	19.4
80.4	4.75	1.31	19.37

80.45714	4.75	1.34	19.34
80.51428	4.73	1.36	19.33
80.57142	4.73	1.35	19.29
80.62858	4.73	1.35	19.28
80.68572	4.76	1.33	19.24
80.74286	4.75	1.35	19.23
80.8	4.76	1.33	19.21
80.85714	4.78	1.32	19.18
80.91428	4.77	1.34	19.16
80.97142	4.73	1.39	19.15
81.02858	4.75	1.37	19.13
81.08572	4.78	1.35	19.14
81.14286	4.79	1.39	19.16
81.2	4.79	1.39	19.18
81.25714	4.75	1.41	19.19
81.31428	4.74	1.41	19.21
81.37142	4.75	1.41	19.23
81.42858	4.74	1.42	19.26
81.48572	4.76	1.4	19.32
81.54286	4.78	1.38	19.36
81.6	4.79	1.39	19.4
81.65714	4.78	1.41	19.43
81.71428	4.81	1.37	19.47
81.77142	4.83	1.36	19.52
81.82858	4.84	1.35	19.56
81.88572	4.84	1.37	19.54
81.94286	4.84	1.39	19.59
82	4.85	1.39	19.59
82.05714	4.83	1.4	19.6
82.11428	4.81	1.39	19.59
82.17142	4.79	1.4	19.61
82.22858	4.79	1.37	19.6
82.28572	4.77	1.42	19.59
82.34286	4.74	1.42	19.6
82.4	4.75	1.43	19.58
82.45714	4.75	1.42	19.56
82.51428	4.78	1.4	19.52
82.57142	4.77	1.42	19.49
82.62858	4.77	1.42	19.48
82.68572	4.78	1.42	19.43
82.74286	4.75	1.43	19.41
82.8	4.75	1.43	19.41
82.85714	4.71	1.42	19.38
82.91428	4.73	1.42	19.36
82.97142	4.76	1.45	19.35
83.02858	4.78	1.46	19.35
83.08572	4.76	1.45	19.36

83.14286	4.77	1.42	19.39
83.2	4.81	1.4	19.43
83.25714	4.83	1.4	19.45
83.31428	4.89	1.35	19.47
83.37142	4.95	1.35	19.5
83.42858	5.02	1.31	19.54
83.48572	5.04	1.29	19.56
83.54286	5.05	1.24	19.61
83.6	5.04	1.25	19.63
83.65714	5.04	1.26	19.67
83.71428	5.03	1.26	19.69
83.77142	5.02	1.28	19.72
83.82858	5.01	1.3	19.76
83.88572	4.97	1.35	19.78
83.94286	4.92	1.35	19.77
84	4.91	1.38	19.77
84.05714	4.9	1.39	19.77
84.11428	4.91	1.42	19.75
84.17142	4.91	1.41	19.74
84.22858	4.91	1.42	19.72
84.28572	4.93	1.41	19.72
84.34286	4.91	1.43	19.68
84.4	4.9	1.42	19.67
84.45714	4.93	1.42	19.67
84.51428	4.94	1.44	19.63
84.57142	4.92	1.45	19.6
84.62858	4.92	1.46	19.57
84.68572	4.89	1.48	19.55
84.74286	4.89	1.47	19.53
84.8	4.91	1.45	19.51
84.85714	4.9	1.47	19.5
84.91428	4.91	1.46	19.5
84.97142	4.92	1.47	19.49
85.02858	4.93	1.43	19.49
85.08572	4.9	1.43	19.5
85.14286	4.91	1.41	19.52
85.2	4.9	1.39	19.55
85.25714	4.91	1.39	19.55
85.31428	4.91	1.39	19.58
85.37142	4.92	1.37	19.61
85.42858	4.94	1.35	19.64
85.48572	4.93	1.37	19.67
85.54286	4.94	1.38	19.72
85.6	4.93	1.42	19.75
85.65714	4.93	1.41	19.76
85.71428	4.95	1.44	19.78
85.77142	4.97	1.43	19.82

85.82858	4.99	1.44	19.86
85.88572	4.99	1.42	19.87
85.94286	5	1.47	19.87
86	4.99	1.49	19.86
86.05714	5	1.48	19.86
86.11428	5.01	1.48	19.81
86.17142	5.01	1.49	19.82
86.22858	5.01	1.48	19.82
86.28572	5.01	1.45	19.82
86.34286	5.04	1.46	19.79
86.4	5.03	1.46	19.79
86.45714	5.04	1.48	19.78
86.51428	5.01	1.51	19.75
86.57142	5.01	1.5	19.74
86.62858	5.04	1.5	19.75
86.68572	5.05	1.49	19.7
86.74286	5.08	1.45	19.69
86.8	5.08	1.46	19.69
86.85714	5.09	1.47	19.7
86.91428	5.04	1.49	19.71
86.97142	5.03	1.48	19.71
87.02858	5.02	1.49	19.74
87.08572	5.02	1.45	19.74
87.14286	5.04	1.46	19.76
87.2	5	1.45	19.79
87.25714	5.01	1.45	19.81
87.31428	4.97	1.49	19.83
87.37142	5	1.49	19.85
87.42858	5.01	1.49	19.87
87.48572	5.06	1.48	19.9
87.54286	5.09	1.48	19.92
87.6	5.09	1.49	19.95
87.65714	5.11	1.49	19.99
87.71428	5.11	1.5	19.99
87.77142	5.09	1.53	20
87.82858	5.08	1.53	19.98
87.88572	5.12	1.53	19.98
87.94286	5.12	1.53	19.97
88	5.12	1.54	19.97
88.05714	5.09	1.56	19.97
88.11428	5.09	1.57	19.95
88.17142	5.08	1.54	19.93
88.22858	5.07	1.54	19.92
88.28572	5.09	1.52	19.91
88.34286	5.1	1.49	19.89
88.4	5.13	1.49	19.88
88.45714	5.14	1.49	19.87

88.51428	5.11	1.48	19.84
88.57142	5.09	1.49	19.82
88.62858	5.09	1.48	19.82
88.68572	5.08	1.47	19.77
88.74286	5.08	1.49	19.77
88.8	5.1	1.49	19.76
88.85714	5.09	1.52	19.78
88.91428	5.07	1.52	19.77
88.97142	5.05	1.54	19.8
89.02858	5.04	1.52	19.82
89.08572	5.07	1.52	19.84
89.14286	5.1	1.53	19.85
89.2	5.12	1.54	19.88
89.25714	5.14	1.54	19.89
89.31428	5.14	1.53	19.94
89.37142	5.16	1.52	19.96
89.42858	5.16	1.5	19.99
89.48572	5.19	1.49	20.02
89.54286	5.21	1.49	20.04
89.6	5.21	1.52	20.08
89.65714	5.19	1.51	20.13
89.71428	5.19	1.51	20.15
89.77142	5.21	1.5	20.16
89.82858	5.21	1.52	20.18
89.88572	5.22	1.52	20.18
89.94286	5.22	1.53	20.17
90	5.22	1.53	20.14
90.05714	5.24	1.55	20.14
90.11428	5.22	1.52	20.11
90.17142	5.22	1.51	20.1
90.22858	5.21	1.57	20.1
90.28572	5.23	1.54	20.07
90.34286	5.22	1.53	20.07
90.4	5.17	1.52	20.04
90.45714	5.18	1.53	20.04
90.51428	5.17	1.54	20.03
90.57142	5.2	1.55	20.01
90.62858	5.22	1.56	19.99
90.68572	5.2	1.56	19.97
90.74286	5.2	1.55	19.96
90.8	5.22	1.55	19.93
90.85714	5.21	1.54	19.94
90.91428	5.25	1.54	19.96
90.97142	5.22	1.55	19.97
91.02858	5.26	1.53	19.98
91.08572	5.25	1.53	20.01
91.14286	5.26	1.5	20.04

91.2	5.22	1.52	20.04
91.25714	5.24	1.53	20.08
91.31428	5.22	1.57	20.13
91.37142	5.22	1.57	20.15
91.42858	5.25	1.58	20.16
91.48572	5.22	1.57	20.2
91.54286	5.23	1.57	20.25
91.6	5.21	1.6	20.28
91.65714	5.24	1.6	20.31
91.71428	5.23	1.62	20.33
91.77142	5.27	1.59	20.32
91.82858	5.3	1.56	20.32
91.88572	5.31	1.56	20.28
91.94286	5.33	1.55	20.26
92	5.31	1.56	20.25
92.05714	5.32	1.57	20.23
92.11428	5.34	1.56	20.24
92.17142	5.37	1.55	20.21
92.22858	5.38	1.55	20.17
92.28572	5.36	1.55	20.12
92.34286	5.34	1.57	20.13
92.4	5.33	1.6	20.1
92.45714	5.3	1.59	20.07
92.51428	5.29	1.59	20.04
92.57142	5.31	1.56	20
92.62858	5.3	1.57	19.97
92.68572	5.29	1.59	19.97
92.74286	5.27	1.6	19.93
92.8	5.27	1.59	19.93
92.85714	5.28	1.6	19.98
92.91428	5.28	1.6	20.01
92.97142	5.3	1.58	20.04
93.02858	5.34	1.58	20.05
93.08572	5.34	1.56	20.07
93.14286	5.34	1.57	20.09
93.2	5.36	1.55	20.13
93.25714	5.34	1.54	20.15
93.31428	5.34	1.57	20.18
93.37142	5.34	1.57	20.22
93.42858	5.34	1.59	20.23
93.48572	5.32	1.6	20.24
93.54286	5.3	1.64	20.3
93.6	5.29	1.65	20.31
93.65714	5.32	1.69	20.34
93.71428	5.33	1.69	20.35
93.77142	5.31	1.69	20.36
93.82858	5.29	1.7	20.36

93.88572	5.31	1.7	20.35
93.94286	5.28	1.71	20.35
94	5.29	1.69	20.34
94.05714	5.29	1.67	20.31
94.11428	5.31	1.65	20.3
94.17142	5.31	1.67	20.28
94.22858	5.31	1.65	20.25
94.28572	5.28	1.68	20.22
94.34286	5.31	1.69	20.18
94.4	5.33	1.67	20.17
94.45714	5.31	1.66	20.17
94.51428	5.35	1.64	20.13
94.57142	5.36	1.63	20.12
94.62858	5.38	1.65	20.1
94.68572	5.39	1.64	20.07
94.74286	5.41	1.64	20.05
94.8	5.41	1.63	20.05
94.85714	5.39	1.63	20.06
94.91428	5.4	1.6	20.08
94.97142	5.42	1.6	20.11
95.02858	5.44	1.61	20.16
95.08572	5.41	1.62	20.19
95.14286	5.4	1.6	20.21
95.2	5.41	1.56	20.23
95.25714	5.4	1.57	20.28
95.31428	5.39	1.56	20.33
95.37142	5.4	1.58	20.35
95.42858	5.39	1.58	20.38
95.48572	5.38	1.59	20.42
95.54286	5.38	1.59	20.44
95.6	5.38	1.6	20.47
95.65714	5.41	1.56	20.51
95.71428	5.41	1.59	20.51
95.77142	5.4	1.62	20.51
95.82858	5.42	1.62	20.48
95.88572	5.41	1.67	20.45
95.94286	5.44	1.64	20.42
96	5.44	1.65	20.39
96.05714	5.46	1.65	20.38
96.11428	5.48	1.66	20.38
96.17142	5.45	1.68	20.38
96.22858	5.46	1.7	20.36
96.28572	5.48	1.69	20.36
96.34286	5.49	1.66	20.36
96.4	5.49	1.65	20.33
96.45714	5.49	1.63	20.32
96.51428	5.47	1.65	20.31

96.57142	5.46	1.64	20.28
96.62858	5.47	1.64	20.25
96.68572	5.45	1.66	20.23
96.74286	5.49	1.63	20.22
96.8	5.47	1.66	20.24
96.85714	5.47	1.67	20.26
96.91428	5.46	1.71	20.27
96.97142	5.44	1.72	20.28
97.02858	5.46	1.69	20.31
97.08572	5.47	1.67	20.32
97.14286	5.49	1.66	20.33
97.2	5.5	1.65	20.36
97.25714	5.49	1.65	20.37
97.31428	5.49	1.64	20.38
97.37142	5.5	1.65	20.42
97.42858	5.51	1.64	20.47
97.48572	5.51	1.61	20.52
97.54286	5.53	1.6	20.58
97.6	5.53	1.6	20.61
97.65714	5.53	1.63	20.62
97.71428	5.52	1.66	20.63
97.77142	5.5	1.7	20.64
97.82858	5.49	1.69	20.64
97.88572	5.51	1.71	20.65
97.94286	5.51	1.7	20.62
98	5.49	1.71	20.58
98.05714	5.49	1.71	20.56
98.11428	5.5	1.73	20.56
98.17142	5.48	1.76	20.56
98.22858	5.48	1.75	20.54
98.28572	5.5	1.75	20.52
98.34286	5.49	1.75	20.49
98.4	5.5	1.76	20.44
98.45714	5.49	1.79	20.39
98.51428	5.51	1.79	20.35
98.57142	5.52	1.79	20.31
98.62858	5.55	1.79	20.29
98.68572	5.54	1.79	20.27
98.74286	5.55	1.78	20.29
98.8	5.56	1.76	20.3
98.85714	5.55	1.75	20.34
98.91428	5.58	1.72	20.35
98.97142	5.59	1.71	20.38
99.02858	5.62	1.69	20.41
99.08572	5.61	1.68	20.42
99.14286	5.58	1.65	20.45
99.2	5.58	1.66	20.49

99.25714	5.58	1.65	20.5
99.31428	5.6	1.66	20.53
99.37142	5.58	1.67	20.56
99.42858	5.61	1.68	20.62
99.48572	5.61	1.68	20.64
99.54286	5.58	1.68	20.7
99.6	5.56	1.68	20.73
99.65714	5.56	1.69	20.74
99.71428	5.55	1.72	20.73
99.77142	5.53	1.73	20.7
99.82858	5.54	1.72	20.67
99.88572	5.56	1.73	20.65
99.94286	5.57	1.75	20.64
100	5.56	1.75	20.62
100.0571	5.58	1.76	20.58
100.1143	5.56	1.77	20.51
100.1714	5.58	1.77	20.5
100.2286	5.59	1.78	20.49
100.2857	5.62	1.75	20.49
100.3429	5.63	1.73	20.46
100.4	5.64	1.73	20.44
100.4571	5.59	1.77	20.43
100.5143	5.57	1.78	20.42
100.5714	5.56	1.79	20.4
100.6286	5.55	1.78	20.39
100.6857	5.58	1.77	20.42
100.7429	5.59	1.78	20.44
100.8	5.59	1.78	20.47
100.8571	5.55	1.8	20.5
100.9143	5.54	1.82	20.53
100.9714	5.55	1.82	20.55
101.0286	5.59	1.8	20.62
101.0857	5.61	1.77	20.65
101.1429	5.63	1.78	20.66
101.2	5.65	1.78	20.65
101.2571	5.63	1.8	20.69
101.3143	5.63	1.77	20.72
101.3714	5.65	1.77	20.75
101.4286	5.68	1.76	20.76
101.4857	5.68	1.78	20.81
101.5429	5.66	1.79	20.82
101.6	5.65	1.76	20.84
101.6571	5.65	1.78	20.86
101.7143	5.66	1.79	20.86
101.7714	5.65	1.77	20.84
101.8286	5.65	1.77	20.81
101.8857	5.65	1.79	20.81

101.9429	5.64	1.79	20.8
102	5.62	1.79	20.8
102.0571	5.64	1.77	20.79
102.1143	5.64	1.8	20.8
102.1714	5.66	1.84	20.79
102.2286	5.68	1.83	20.77
102.2857	5.67	1.82	20.73
102.3429	5.67	1.83	20.72
102.4	5.66	1.81	20.69
102.4571	5.69	1.79	20.64
102.5143	5.66	1.8	20.6
102.5714	5.7	1.78	20.55
102.6286	5.69	1.79	20.57
102.6857	5.7	1.75	20.58
102.7429	5.7	1.75	20.62
102.8	5.7	1.76	20.64
102.8571	5.7	1.76	20.64
102.9143	5.72	1.78	20.64
102.9714	5.72	1.79	20.66
103.0286	5.71	1.83	20.68
103.0857	5.74	1.82	20.71
103.1429	5.74	1.85	20.73
103.2	5.75	1.84	20.78
103.2571	5.75	1.85	20.81
103.3143	5.74	1.84	20.83
103.3714	5.72	1.84	20.89
103.4286	5.74	1.83	20.92
103.4857	5.73	1.79	20.95
103.5429	5.73	1.79	20.96
103.6	5.75	1.76	20.96
103.6571	5.76	1.77	20.94
103.7143	5.78	1.75	20.89
103.7714	5.79	1.74	20.88
103.8286	5.78	1.73	20.89
103.8857	5.8	1.71	20.87
103.9429	5.82	1.7	20.85
104	5.83	1.71	20.83
104.0571	5.82	1.74	20.81
104.1143	5.79	1.76	20.79
104.1714	5.8	1.74	20.78
104.2286	5.81	1.74	20.75
104.2857	5.79	1.73	20.73
104.3429	5.77	1.75	20.72
104.4	5.77	1.78	20.7
104.4571	5.74	1.83	20.7
104.5143	5.73	1.86	20.71
104.5714	5.74	1.83	20.73

104.6286	5.75	1.84	20.76
104.6857	5.76	1.85	20.77
104.7429	5.76	1.88	20.77
104.8	5.75	1.9	20.79
104.8571	5.75	1.9	20.81
104.9143	5.75	1.89	20.82
104.9714	5.75	1.89	20.85
105.0286	5.76	1.86	20.88
105.0857	5.78	1.84	20.89
105.1429	5.76	1.84	20.91
105.2	5.76	1.84	20.93
105.2571	5.76	1.83	20.94
105.3143	5.77	1.81	20.95
105.3714	5.76	1.84	20.97
105.4286	5.76	1.86	20.98
105.4857	5.77	1.87	21
105.5429	5.81	1.85	21
105.6	5.84	1.82	21.01
105.6571	5.84	1.82	21.01
105.7143	5.84	1.84	20.99
105.7714	5.85	1.83	21
105.8286	5.85	1.85	20.98
105.8857	5.84	1.85	20.97
105.9429	5.87	1.82	20.95
106	5.87	1.82	20.93
106.0571	5.87	1.82	20.92
106.1143	5.83	1.83	20.91
106.1714	5.81	1.84	20.89
106.2286	5.82	1.84	20.88
106.2857	5.83	1.83	20.88
106.3429	5.81	1.86	20.85
106.4	5.8	1.82	20.83
106.4571	5.79	1.83	20.78
106.5143	5.77	1.82	20.75
106.5714	5.77	1.83	20.77
106.6286	5.76	1.81	20.79
106.6857	5.76	1.84	20.8
106.7429	5.79	1.82	20.83
106.8	5.79	1.82	20.85
106.8571	5.79	1.84	20.89
106.9143	5.79	1.83	20.9
106.9714	5.79	1.84	20.92
107.0286	5.83	1.85	20.97
107.0857	5.86	1.85	20.99
107.1429	5.88	1.83	21.02
107.2	5.89	1.86	21.02
107.2571	5.91	1.83	21.04

107.3143	5.91	1.84	21.04
107.3714	5.92	1.87	21.07
107.4286	5.94	1.83	21.11
107.4857	5.97	1.82	21.14
107.5429	6	1.81	21.11
107.6	6.03	1.79	21.12
107.6571	6.02	1.79	21.13
107.7143	6	1.8	21.13
107.7714	6.02	1.78	21.09
107.8286	5.99	1.81	21.06
107.8857	6	1.82	21.05
107.9429	5.96	1.84	21.01
108	5.93	1.88	20.96
108.0571	5.93	1.88	20.95
108.1143	5.91	1.88	20.96
108.1714	5.89	1.9	20.94
108.2286	5.89	1.9	20.94
108.2857	5.91	1.91	20.92
108.3429	5.89	1.93	20.91
108.4	5.93	1.89	20.87
108.4571	5.95	1.86	20.87
108.5143	5.97	1.81	20.87
108.5714	5.98	1.81	20.85
108.6286	5.94	1.84	20.85
108.6857	5.92	1.86	20.87
108.7429	5.91	1.84	20.93
108.8	5.94	1.85	20.95
108.8571	5.95	1.83	20.96
108.9143	5.93	1.85	21.01
108.9714	5.92	1.87	21.05
109.0286	5.93	1.87	21.07
109.0857	5.94	1.9	21.12
109.1429	5.97	1.89	21.16
109.2	6.01	1.83	21.21
109.2571	6.04	1.83	21.25
109.3143	6.06	1.86	21.28
109.3714	6.02	1.88	21.27
109.4286	6	1.88	21.26
109.4857	6.02	1.87	21.26
109.5429	6.02	1.86	21.26
109.6	6.02	1.87	21.25
109.6571	6	1.86	21.23
109.7143	6	1.87	21.19
109.7714	6	1.91	21.2
109.8286	6	1.92	21.21
109.8857	5.99	1.93	21.2
109.9429	6.03	1.91	21.19

110	6.04	1.91	21.16
110.0571	6.03	1.89	21.13
110.1143	6.04	1.92	21.1
110.1714	6.05	1.93	21.06
110.2286	6.04	1.94	21.02
110.2857	6.01	1.96	21.03
110.3429	6	1.95	21.04
110.4	6.01	1.94	21.02
110.4571	6.03	1.92	21.04
110.5143	6.05	1.91	21.07
110.5714	6.06	1.89	21.07
110.6286	6.07	1.88	21.09
110.6857	6.05	1.9	21.13
110.7429	6.04	1.91	21.15
110.8	6.04	1.91	21.14
110.8571	6.08	1.9	21.14
110.9143	6.1	1.88	21.15
110.9714	6.09	1.89	21.18
111.0286	6.06	1.91	21.21
111.0857	6.04	1.94	21.24
111.1429	6.05	1.96	21.29
111.2	6.04	1.98	21.3
111.2571	6.06	1.94	21.32
111.3143	6.06	1.94	21.36
111.3714	6.08	1.91	21.39
111.4286	6.05	1.91	21.4
111.4857	6.03	1.94	21.37
111.5429	6.03	1.94	21.37
111.6	6.04	1.95	21.37
111.6571	6.07	1.94	21.37
111.7143	6.06	1.94	21.36
111.7714	6.07	1.95	21.34
111.8286	6.09	1.95	21.32
111.8857	6.11	1.93	21.31
111.9429	6.09	1.93	21.27
112	6.11	1.92	21.26
112.0571	6.14	1.9	21.23
112.1143	6.18	1.88	21.22
112.1714	6.16	1.88	21.2
112.2286	6.17	1.86	21.18
112.2857	6.16	1.85	21.13
112.3429	6.16	1.83	21.09
112.4	6.15	1.82	21.08
112.4571	6.15	1.83	21.07
112.5143	6.18	1.81	21.08
112.5714	6.17	1.85	21.07
112.6286	6.15	1.85	21.07

112.6857	6.15	1.85	21.1
112.7429	6.14	1.86	21.13
112.8	6.16	1.88	21.16
112.8571	6.17	1.89	21.2
112.9143	6.18	1.91	21.21
112.9714	6.15	1.93	21.25
113.0286	6.14	1.93	21.26
113.0857	6.11	1.95	21.28
113.1429	6.14	1.94	21.32
113.2	6.15	1.95	21.34
113.2571	6.13	1.95	21.39
113.3143	6.12	1.94	21.41
113.3714	6.1	1.93	21.41
113.4286	6.08	1.94	21.43
113.4857	6.06	1.93	21.42
113.5429	6.07	1.9	21.41
113.6	6.05	1.91	21.42
113.6571	6.05	1.92	21.39
113.7143	6.05	1.92	21.4
113.7714	6.02	1.93	21.4
113.8286	6.03	1.93	21.38
113.8857	6.04	1.92	21.38
113.9429	6.05	1.93	21.38
114	6.08	1.92	21.37
114.0571	6.11	1.94	21.33
114.1143	6.13	1.97	21.3
114.1714	6.16	1.96	21.26
114.2286	6.18	1.94	21.24
114.2857	6.2	1.94	21.23
114.3429	6.2	1.95	21.22
114.4	6.21	1.96	21.22
114.4571	6.23	1.95	21.26
114.5143	6.25	1.94	21.25
114.5714	6.24	1.96	21.27
114.6286	6.25	1.94	21.31
114.6857	6.23	1.94	21.31
114.7429	6.25	1.92	21.3
114.8	6.25	1.93	21.31
114.8571	6.23	1.94	21.3
114.9143	6.23	1.94	21.31
114.9714	6.25	1.92	21.37
115.0286	6.24	1.92	21.41
115.0857	6.23	1.95	21.46
115.1429	6.2	1.96	21.49
115.2	6.2	1.97	21.5
115.2571	6.21	1.97	21.52
115.3143	6.23	1.95	21.53

115.3714	6.22	1.95	21.52
115.4286	6.25	1.92	21.5
115.4857	6.25	1.93	21.5
115.5429	6.28	1.95	21.5
115.6	6.28	1.96	21.46
115.6571	6.29	1.94	21.47
115.7143	6.33	1.92	21.45
115.7714	6.31	1.93	21.43
115.8286	6.31	1.91	21.43
115.8857	6.3	1.93	21.4
115.9429	6.27	1.95	21.35
116	6.28	1.96	21.34
116.0571	6.26	1.97	21.32
116.1143	6.22	1.97	21.29
116.1714	6.2	2	21.28
116.2286	6.2	2.01	21.24
116.2857	6.18	1.99	21.23
116.3429	6.18	2	21.22
116.4	6.19	2	21.25
116.4571	6.17	2.01	21.25
116.5143	6.19	2	21.26
116.5714	6.17	2.03	21.25
116.6286	6.15	2.03	21.3
116.6857	6.2	2.03	21.34
116.7429	6.19	2.03	21.37
116.8	6.21	2.03	21.38
116.8571	6.22	2.06	21.41
116.9143	6.22	2.06	21.43
116.9714	6.23	2.06	21.46
117.0286	6.24	2.05	21.52
117.0857	6.25	2.05	21.58
117.1429	6.28	2.02	21.59
117.2	6.32	2.02	21.59
117.2571	6.32	2	21.62
117.3143	6.35	1.99	21.63
117.3714	6.36	2	21.62
117.4286	6.36	1.99	21.61
117.4857	6.38	1.97	21.63
117.5429	6.38	1.99	21.61
117.6	6.37	1.98	21.55
117.6571	6.36	2.01	21.52
117.7143	6.36	2.05	21.52
117.7714	6.33	2.05	21.5
117.8286	6.33	2.08	21.48
117.8857	6.32	2.09	21.47
117.9429	6.31	2.08	21.43
118	6.31	2.05	21.4

118.0571	6.28	2.07	21.39
118.1143	6.28	2.09	21.37
118.1714	6.29	2.09	21.38
118.2286	6.3	2.08	21.36
118.2857	6.29	2.07	21.33
118.3429	6.27	2.09	21.36
118.4	6.27	2.06	21.37
118.4571	6.28	2.04	21.38
118.5143	6.27	2.05	21.4
118.5714	6.27	2.08	21.45
118.6286	6.25	2.07	21.46
118.6857	6.27	2.05	21.48
118.7429	6.26	2.08	21.51
118.8	6.27	2.08	21.52
118.8571	6.28	2.06	21.55
118.9143	6.3	2.03	21.56
118.9714	6.31	2.05	21.56
119.0286	6.29	2.04	21.6
119.0857	6.28	2.05	21.63
119.1429	6.28	2.03	21.64
119.2	6.29	2.07	21.65
119.2571	6.29	2.09	21.66
119.3143	6.27	2.1	21.62
119.3714	6.27	2.09	21.63
119.4286	6.26	2.09	21.64
119.4857	6.25	2.09	21.64
119.5429	6.27	2.07	21.62
119.6	6.29	2.08	21.62
119.6571	6.33	2.07	21.61
119.7143	6.36	2.09	21.62
119.7714	6.37	2.06	21.58
119.8286	6.4	2.04	21.56
119.8857	6.42	2.02	21.56
119.9429	6.42	2.05	21.53
120	6.45	2.07	21.49
120.0571	6.48	2.06	21.46
120.1143	6.47	2.06	21.44
120.1714	6.47	2.08	21.44
120.2286	6.47	2.09	21.43
120.2857	6.48	2.08	21.45
120.3429	6.47	2.1	21.45
120.4	6.43	2.13	21.45
120.4571	6.43	2.14	21.45
120.5143	6.43	2.09	21.46
120.5714	6.42	2.09	21.47
120.6286	6.4	2.1	21.49
120.6857	6.4	2.1	21.51

120.7429	6.39	2.07	21.55
120.8	6.37	2.09	21.58
120.8571	6.39	2.08	21.6
120.9143	6.39	2.04	21.63
120.9714	6.42	2	21.67
121.0286	6.41	2.01	21.71
121.0857	6.42	2.05	21.71
121.1429	6.45	2.04	21.75
121.2	6.45	2.06	21.74
121.2571	6.44	2.08	21.72
121.3143	6.44	2.09	21.72
121.3714	6.44	2.09	21.71
121.4286	6.4	2.12	21.7
121.4857	6.4	2.15	21.7
121.5429	6.38	2.18	21.66
121.6	6.37	2.18	21.65
121.6571	6.37	2.18	21.66
121.7143	6.34	2.18	21.61
121.7714	6.34	2.19	21.59
121.8286	6.39	2.18	21.58
121.8857	6.4	2.18	21.55
121.9429	6.4	2.17	21.54
122	6.43	2.14	21.55
122.0571	6.45	2.12	21.52
122.1143	6.45	2.11	21.49
122.1714	6.48	2.12	21.49
122.2286	6.49	2.07	21.5
122.2857	6.5	2.08	21.53
122.3429	6.5	2.06	21.54
122.4	6.49	2.07	21.55
122.4571	6.47	2.09	21.56
122.5143	6.51	2.08	21.56
122.5714	6.52	2.08	21.58
122.6286	6.51	2.11	21.61
122.6857	6.53	2.11	21.66
122.7429	6.53	2.12	21.69
122.8	6.52	2.13	21.72
122.8571	6.5	2.13	21.75
122.9143	6.54	2.13	21.75
122.9714	6.52	2.13	21.77
123.0286	6.51	2.11	21.81
123.0857	6.52	2.12	21.86
123.1429	6.49	2.13	21.86
123.2	6.47	2.13	21.87
123.2571	6.48	2.13	21.89
123.3143	6.49	2.12	21.87
123.3714	6.52	2.1	21.85

123.4286	6.53	2.13	21.86
123.4857	6.5	2.17	21.86
123.5429	6.53	2.15	21.83
123.6	6.52	2.18	21.8
123.6571	6.51	2.18	21.78
123.7143	6.55	2.17	21.77
123.7714	6.56	2.17	21.74
123.8286	6.54	2.2	21.73
123.8857	6.52	2.17	21.71
123.9429	6.49	2.19	21.69
124	6.49	2.18	21.67
124.0571	6.49	2.16	21.67
124.1143	6.5	2.16	21.64
124.1714	6.48	2.17	21.58
124.2286	6.48	2.17	21.59
124.2857	6.42	2.18	21.62
124.3429	6.43	2.18	21.66
124.4	6.42	2.17	21.66
124.4571	6.44	2.21	21.67
124.5143	6.44	2.22	21.69
124.5714	6.47	2.18	21.72
124.6286	6.5	2.17	21.75
124.6857	6.49	2.19	21.78
124.7429	6.54	2.17	21.8
124.8	6.56	2.16	21.83
124.8571	6.58	2.16	21.86
124.9143	6.58	2.16	21.86
124.9714	6.6	2.18	21.88
125.0286	6.62	2.18	21.89
125.0857	6.62	2.17	21.88
125.1429	6.63	2.15	21.9
125.2	6.61	2.17	21.91
125.2571	6.57	2.21	21.91
125.3143	6.58	2.21	21.89
125.3714	6.57	2.21	21.86
125.4286	6.52	2.22	21.86
125.4857	6.48	2.25	21.86
125.5429	6.45	2.25	21.84
125.6	6.47	2.23	21.81
125.6571	6.46	2.22	21.76
125.7143	6.47	2.24	21.75
125.7714	6.49	2.23	21.74
125.8286	6.48	2.22	21.71
125.8857	6.48	2.23	21.69
125.9429	6.47	2.23	21.66
126	6.48	2.25	21.64
126.0571	6.48	2.2	21.65

126.1143	6.51	2.18	21.65
126.1714	6.47	2.19	21.62
126.2286	6.51	2.2	21.63
126.2857	6.51	2.19	21.66
126.3429	6.5	2.19	21.67
126.4	6.5	2.19	21.7
126.4571	6.52	2.18	21.71
126.5143	6.52	2.16	21.74
126.5714	6.54	2.13	21.8
126.6286	6.59	2.13	21.84
126.6857	6.62	2.14	21.85
126.7429	6.6	2.15	21.88
126.8	6.59	2.18	21.9
126.8571	6.61	2.19	21.93
126.9143	6.63	2.19	21.95
126.9714	6.66	2.18	21.99
127.0286	6.63	2.2	22.01
127.0857	6.64	2.21	22.02
127.1429	6.62	2.26	22.04
127.2	6.59	2.29	22.02
127.2571	6.6	2.28	22
127.3143	6.6	2.27	21.98
127.3714	6.59	2.26	21.98
127.4286	6.58	2.23	21.97
127.4857	6.54	2.27	21.94
127.5429	6.51	2.27	21.88
127.6	6.52	2.27	21.88
127.6571	6.53	2.29	21.86
127.7143	6.53	2.26	21.83
127.7714	6.52	2.25	21.83
127.8286	6.53	2.28	21.81
127.8857	6.53	2.28	21.8
127.9429	6.53	2.31	21.76
128	6.5	2.32	21.74
128.0571	6.52	2.31	21.73
128.1143	6.51	2.31	21.72
128.1714	6.54	2.3	21.73
128.2286	6.54	2.3	21.76
128.2857	6.58	2.3	21.78
128.3429	6.59	2.33	21.78
128.4	6.57	2.32	21.83
128.4571	6.55	2.33	21.86
128.5143	6.57	2.31	21.9
128.5714	6.58	2.29	21.91
128.6286	6.61	2.28	21.93
128.6857	6.59	2.29	21.94
128.7429	6.6	2.27	21.98

128.8	6.58	2.27	22.01
128.8571	6.57	2.28	22.05
128.9143	6.58	2.3	22.08
128.9714	6.59	2.29	22.12
129.0286	6.62	2.28	22.13
129.0857	6.59	2.28	22.14
129.1429	6.59	2.28	22.15
129.2	6.6	2.27	22.15
129.2571	6.61	2.24	22.13
129.3143	6.62	2.26	22.1
129.3714	6.62	2.25	22.07
129.4286	6.65	2.23	22.07
129.4857	6.66	2.21	22.03
129.5429	6.63	2.22	22.04
129.6	6.62	2.23	22.04
129.6571	6.66	2.22	22.02
129.7143	6.7	2.22	22
129.7714	6.69	2.25	21.98
129.8286	6.65	2.28	21.95
129.8857	6.65	2.3	21.95
129.9429	6.65	2.3	21.96
130	6.62	2.32	21.94
130.0571	6.63	2.29	21.91
130.1143	6.65	2.29	21.89
130.1714	6.66	2.26	21.91
130.2286	6.63	2.26	21.91
130.2857	6.63	2.27	21.92
130.3429	6.64	2.26	21.92
130.4	6.68	2.25	21.95
130.4571	6.7	2.22	21.96
130.5143	6.72	2.23	21.97
130.5714	6.74	2.24	22
130.6286	6.73	2.26	22.04
130.6857	6.73	2.24	22.05
130.7429	6.72	2.25	22.07
130.8	6.72	2.27	22.11
130.8571	6.71	2.27	22.09
130.9143	6.68	2.3	22.11
130.9714	6.65	2.31	22.13
131.0286	6.65	2.32	22.12
131.0857	6.66	2.32	22.13
131.1429	6.67	2.29	22.13
131.2	6.67	2.3	22.14
131.2571	6.67	2.32	22.14
131.3143	6.68	2.33	22.12
131.3714	6.7	2.32	22.08
131.4286	6.69	2.34	22.09

131.4857	6.7	2.35	22.07
131.5429	6.74	2.34	22.04
131.6	6.74	2.31	22.01
131.6571	6.72	2.3	22.01
131.7143	6.67	2.32	22
131.7714	6.66	2.31	21.99
131.8286	6.67	2.29	21.99
131.8857	6.69	2.29	21.97
131.9429	6.7	2.27	21.99
132	6.72	2.23	21.97
132.0571	6.75	2.18	21.96
132.1143	6.74	2.18	21.96
132.1714	6.72	2.21	21.95
132.2286	6.76	2.19	21.97
132.2857	6.78	2.2	22
132.3429	6.8	2.21	22.02
132.4	6.82	2.24	22
132.4571	6.84	2.26	22.02
132.5143	6.83	2.27	22.05
132.5714	6.85	2.27	22.06
132.6286	6.82	2.28	22.06
132.6857	6.84	2.29	22.11
132.7429	6.85	2.28	22.1
132.8	6.83	2.3	22.14
132.8571	6.86	2.3	22.15
132.9143	6.88	2.29	22.17
132.9714	6.88	2.27	22.17
133.0286	6.85	2.24	22.19
133.0857	6.83	2.26	22.21
133.1429	6.84	2.28	22.21
133.2	6.79	2.32	22.23
133.2571	6.8	2.31	22.23
133.3143	6.78	2.33	22.22
133.3714	6.79	2.36	22.22
133.4286	6.79	2.35	22.19
133.4857	6.76	2.37	22.19
133.5429	6.73	2.37	22.16
133.6	6.75	2.42	22.14
133.6571	6.76	2.4	22.14
133.7143	6.75	2.4	22.12
133.7714	6.77	2.36	22.11
133.8286	6.79	2.38	22.09
133.8857	6.79	2.37	22.07
133.9429	6.81	2.33	22.05
134	6.8	2.33	22.03
134.0571	6.79	2.33	22
134.1143	6.81	2.32	21.99

134.1714	6.81	2.31	21.97
134.2286	6.77	2.34	21.98
134.2857	6.77	2.36	22
134.3429	6.74	2.37	22.03
134.4	6.74	2.37	22.07
134.4571	6.74	2.37	22.11
134.5143	6.71	2.41	22.12
134.5714	6.72	2.43	22.13
134.6286	6.73	2.41	22.14
134.6857	6.75	2.4	22.16
134.7429	6.74	2.39	22.17
134.8	6.76	2.38	22.22
134.8571	6.78	2.37	22.27
134.9143	6.81	2.37	22.29
134.9714	6.82	2.34	22.32
135.0286	6.83	2.34	22.35
135.0857	6.84	2.35	22.35
135.1429	6.86	2.35	22.35
135.2	6.88	2.34	22.35
135.2571	6.85	2.37	22.35
135.3143	6.84	2.38	22.3
135.3714	6.86	2.35	22.27
135.4286	6.86	2.34	22.28
135.4857	6.87	2.35	22.27
135.5429	6.87	2.38	22.25
135.6	6.89	2.38	22.23
135.6571	6.9	2.36	22.22
135.7143	6.89	2.36	22.2
135.7714	6.85	2.38	22.16
135.8286	6.88	2.37	22.14
135.8857	6.89	2.37	22.12
135.9429	6.87	2.39	22.09
136	6.87	2.39	22.07
136.0571	6.85	2.41	22.08
136.1143	6.84	2.4	22.09
136.1714	6.83	2.41	22.11
136.2286	6.82	2.42	22.12
136.2857	6.81	2.42	22.14
136.3429	6.82	2.39	22.13
136.4	6.82	2.39	22.13
136.4571	6.83	2.37	22.16
136.5143	6.87	2.36	22.19
136.5714	6.86	2.38	22.22
136.6286	6.87	2.37	22.24
136.6857	6.88	2.39	22.29
136.7429	6.92	2.38	22.3
136.8	6.9	2.37	22.33

136.8571	6.91	2.36	22.39
136.9143	6.92	2.37	22.42
136.9714	6.92	2.4	22.44
137.0286	6.94	2.41	22.44
137.0857	6.9	2.43	22.44
137.1429	6.92	2.42	22.41
137.2	6.92	2.38	22.43
137.2571	6.9	2.35	22.42
137.3143	6.9	2.37	22.44
137.3714	6.87	2.38	22.42
137.4286	6.89	2.4	22.39
137.4857	6.92	2.39	22.37
137.5429	6.93	2.38	22.34
137.6	6.92	2.4	22.31
137.6571	6.93	2.41	22.28
137.7143	6.93	2.43	22.26
137.7714	6.94	2.44	22.24
137.8286	6.95	2.47	22.21
137.8857	6.97	2.44	22.16
137.9429	6.99	2.45	22.12
138	7.01	2.42	22.13
138.0571	7	2.41	22.15
138.1143	6.99	2.38	22.17
138.1714	7.03	2.37	22.17
138.2286	7.03	2.34	22.19
138.2857	7.01	2.34	22.19
138.3429	7.02	2.32	22.22
138.4	7	2.31	22.25
138.4571	6.97	2.33	22.27
138.5143	7	2.33	22.3
138.5714	6.99	2.31	22.35
138.6286	7.01	2.34	22.38
138.6857	7.01	2.34	22.4
138.7429	6.97	2.34	22.39
138.8	6.97	2.36	22.41
138.8571	6.99	2.36	22.42
138.9143	7.01	2.38	22.44
138.9714	7.03	2.39	22.41
139.0286	7.06	2.35	22.38
139.0857	7.07	2.37	22.36
139.1429	7.03	2.41	22.33
139.2	6.98	2.42	22.33
139.2571	6.96	2.45	22.34
139.3143	6.98	2.48	22.35
139.3714	6.95	2.48	22.31
139.4286	6.95	2.48	22.3
139.4857	6.92	2.49	22.28

139.5429	6.95	2.49	22.25
139.6	6.94	2.5	22.23
139.6571	6.94	2.49	22.23
139.7143	6.97	2.47	22.2
139.7714	7.02	2.46	22.2
139.8286	7.06	2.45	22.19
139.8857	7.05	2.44	22.18
139.9429	7.07	2.42	22.19
140	7.05	2.42	22.21
140.0571	7.06	2.41	22.24
140.1143	7.04	2.44	22.25
140.1714	7.05	2.42	22.24
140.2286	7.06	2.4	22.26
140.2857	7.02	2.42	22.25
140.3429	6.97	2.42	22.27
140.4	6.96	2.42	22.3
140.4571	6.95	2.43	22.32
140.5143	6.95	2.43	22.34
140.5714	6.93	2.45	22.39
140.6286	6.94	2.45	22.42
140.6857	6.95	2.41	22.45
140.7429	6.91	2.44	22.47
140.8	6.9	2.45	22.46
140.8571	6.91	2.47	22.48
140.9143	6.91	2.47	22.47
140.9714	6.93	2.46	22.47
141.0286	6.94	2.45	22.48
141.0857	6.95	2.46	22.48
141.1429	6.99	2.42	22.48
141.2	7.03	2.42	22.47
141.2571	7.04	2.41	22.47
141.3143	7.06	2.42	22.46
141.3714	7.05	2.44	22.44
141.4286	7.07	2.42	22.44
141.4857	7.09	2.45	22.44
141.5429	7.1	2.46	22.42
141.6	7.09	2.45	22.41
141.6571	7.11	2.43	22.39
141.7143	7.12	2.43	22.39
141.7714	7.09	2.46	22.4
141.8286	7.08	2.48	22.39
141.8857	7.09	2.46	22.4
141.9429	7.08	2.46	22.38
142	7.1	2.46	22.36
142.0571	7.09	2.45	22.38
142.1143	7.08	2.44	22.4
142.1714	7.09	2.44	22.43

142.2286	7.08	2.45	22.44
142.2857	7.08	2.45	22.47
142.3429	7.11	2.45	22.48
142.4	7.09	2.46	22.47
142.4571	7.08	2.47	22.48
142.5143	7.08	2.48	22.49
142.5714	7.1	2.5	22.51
142.6286	7.12	2.49	22.56
142.6857	7.14	2.51	22.57
142.7429	7.15	2.48	22.59
142.8	7.15	2.49	22.62
142.8571	7.1	2.5	22.63
142.9143	7.06	2.51	22.64
142.9714	7.05	2.49	22.66
143.0286	7.05	2.51	22.64
143.0857	7.03	2.5	22.62
143.1429	7.01	2.45	22.62
143.2	7	2.46	22.58
143.2571	6.97	2.44	22.58
143.3143	6.95	2.48	22.59
143.3714	6.93	2.5	22.56
143.4286	6.93	2.47	22.52
143.4857	6.94	2.48	22.5
143.5429	6.95	2.48	22.47
143.6	6.98	2.46	22.46
143.6571	7.01	2.44	22.45
143.7143	7.02	2.47	22.42
143.7714	7.04	2.45	22.38
143.8286	7.07	2.45	22.36
143.8857	7.08	2.45	22.33
143.9429	7.09	2.45	22.31
144	7.11	2.44	22.32
144.0571	7.15	2.41	22.33
144.1143	7.14	2.42	22.34
144.1714	7.15	2.46	22.36
144.2286	7.14	2.46	22.36
144.2857	7.13	2.48	22.39
144.3429	7.09	2.52	22.44
144.4	7.08	2.54	22.5
144.4571	7.09	2.54	22.54
144.5143	7.09	2.52	22.57
144.5714	7.1	2.53	22.58
144.6286	7.09	2.54	22.58
144.6857	7.09	2.52	22.62
144.7429	7.08	2.52	22.62
144.8	7.05	2.53	22.65
144.8571	7.06	2.52	22.66

144.9143	7.09	2.49	22.66
144.9714	7.07	2.48	22.65
145.0286	7.09	2.46	22.63
145.0857	7.13	2.48	22.62
145.1429	7.14	2.5	22.61
145.2	7.15	2.51	22.61
145.2571	7.14	2.53	22.56
145.3143	7.14	2.51	22.52
145.3714	7.14	2.5	22.46
145.4286	7.15	2.51	22.43
145.4857	7.16	2.52	22.41
145.5429	7.18	2.52	22.45
145.6	7.17	2.54	22.42
145.6571	7.14	2.55	22.4
145.7143	7.14	2.53	22.38
145.7714	7.13	2.52	22.37
145.8286	7.15	2.52	22.35
145.8857	7.14	2.53	22.38
145.9429	7.15	2.58	22.38
146	7.15	2.58	22.39
146.0571	7.16	2.57	22.41
146.1143	7.14	2.56	22.45
146.1714	7.13	2.57	22.49
146.2286	7.14	2.55	22.52
146.2857	7.13	2.59	22.55
146.3429	7.14	2.6	22.57
146.4	7.12	2.61	22.61
146.4571	7.15	2.58	22.61
146.5143	7.21	2.55	22.61
146.5714	7.2	2.54	22.65
146.6286	7.22	2.55	22.71
146.6857	7.23	2.53	22.76
146.7429	7.25	2.54	22.79
146.8	7.26	2.52	22.79
146.8571	7.24	2.5	22.79
146.9143	7.22	2.54	22.79
146.9714	7.22	2.56	22.75
147.0286	7.22	2.57	22.73
147.0857	7.2	2.57	22.69
147.1429	7.2	2.56	22.68
147.2	7.19	2.58	22.67
147.2571	7.18	2.6	22.65
147.3143	7.17	2.64	22.66
147.3714	7.16	2.66	22.64
147.4286	7.16	2.67	22.64
147.4857	7.18	2.63	22.61
147.5429	7.2	2.59	22.57

147.6	7.2	2.57	22.58
147.6571	7.15	2.59	22.53
147.7143	7.14	2.6	22.52
147.7714	7.14	2.6	22.51
147.8286	7.13	2.59	22.49
147.8857	7.12	2.56	22.53
147.9429	7.11	2.55	22.58
148	7.12	2.56	22.59
148.0571	7.13	2.56	22.59
148.1143	7.13	2.57	22.63
148.1714	7.15	2.56	22.67
148.2286	7.2	2.54	22.67
148.2857	7.22	2.52	22.66
148.3429	7.22	2.5	22.68
148.4	7.26	2.47	22.71
148.4571	7.29	2.46	22.76
148.5143	7.3	2.5	22.76
148.5714	7.31	2.5	22.78
148.6286	7.32	2.48	22.81
148.6857	7.33	2.48	22.82
148.7429	7.29	2.53	22.85
148.8	7.25	2.54	22.86
148.8571	7.23	2.57	22.82
148.9143	7.23	2.59	22.79
148.9714	7.22	2.63	22.78
149.0286	7.2	2.64	22.78
149.0857	7.19	2.61	22.76
149.1429	7.18	2.6	22.75
149.2	7.21	2.63	22.74
149.2571	7.19	2.62	22.74
149.3143	7.21	2.61	22.74
149.3714	7.25	2.62	22.7
149.4286	7.23	2.62	22.68
149.4857	7.24	2.6	22.62
149.5429	7.24	2.61	22.6
149.6	7.25	2.61	22.58
149.6571	7.25	2.61	22.56
149.7143	7.25	2.61	22.54
149.7714	7.26	2.59	22.55
149.8286	7.26	2.62	22.59
149.8857	7.25	2.63	22.6
149.9429	7.21	2.61	22.63
150	7.23	2.62	22.63
150.0571	7.22	2.63	22.64
150.1143	7.23	2.63	22.67
150.1714	7.23	2.65	22.71
150.2286	7.25	2.64	22.72

150.2857	7.28	2.64	22.73
150.3429	7.27	2.64	22.76
150.4	7.26	2.63	22.81
150.4571	7.26	2.63	22.83
150.5143	7.29	2.63	22.86
150.5714	7.31	2.61	22.89
150.6286	7.31	2.61	22.93
150.6857	7.33	2.6	22.95
150.7429	7.32	2.59	22.92
150.8	7.33	2.61	22.94
150.8571	7.3	2.61	22.93
150.9143	7.26	2.65	22.9
150.9714	7.29	2.63	22.9
151.0286	7.28	2.63	22.89
151.0857	7.3	2.62	22.87
151.1429	7.31	2.6	22.83
151.2	7.34	2.61	22.85
151.2571	7.31	2.61	22.85
151.3143	7.32	2.63	22.84
151.3714	7.32	2.61	22.8
151.4286	7.35	2.59	22.81
151.4857	7.35	2.59	22.78
151.5429	7.34	2.59	22.75
151.6	7.38	2.58	22.72
151.6571	7.37	2.58	22.71
151.7143	7.35	2.6	22.69
151.7714	7.37	2.59	22.68
151.8286	7.37	2.6	22.73
151.8857	7.37	2.62	22.74
151.9429	7.37	2.62	22.75
152	7.36	2.65	22.75
152.0571	7.36	2.66	22.8
152.1143	7.38	2.69	22.78
152.1714	7.36	2.7	22.78
152.2286	7.36	2.74	22.78
152.2857	7.32	2.74	22.81
152.3429	7.33	2.74	22.83
152.4	7.29	2.76	22.86
152.4571	7.32	2.74	22.89
152.5143	7.31	2.74	22.9
152.5714	7.31	2.74	22.93
152.6286	7.32	2.71	22.93
152.6857	7.32	2.67	22.92
152.7429	7.35	2.64	22.91
152.8	7.33	2.61	22.91
152.8571	7.35	2.59	22.89
152.9143	7.37	2.58	22.87

152.9714	7.39	2.54	22.86
153.0286	7.39	2.53	22.84
153.0857	7.39	2.54	22.84
153.1429	7.38	2.54	22.82
153.2	7.36	2.55	22.82
153.2571	7.34	2.58	22.79
153.3143	7.33	2.6	22.77
153.3714	7.32	2.62	22.76
153.4286	7.31	2.64	22.76
153.4857	7.31	2.65	22.74
153.5429	7.31	2.7	22.72
153.6	7.28	2.73	22.71
153.6571	7.27	2.75	22.69
153.7143	7.28	2.73	22.68
153.7714	7.27	2.74	22.67
153.8286	7.26	2.72	22.69
153.8857	7.27	2.69	22.72
153.9429	7.29	2.69	22.73
154	7.32	2.67	22.74
154.0571	7.33	2.65	22.75
154.1143	7.35	2.62	22.79
154.1714	7.38	2.58	22.82
154.2286	7.39	2.55	22.85
154.2857	7.39	2.55	22.87
154.3429	7.4	2.57	22.88
154.4	7.41	2.58	22.91
154.4571	7.43	2.61	22.96
154.5143	7.42	2.63	23
154.5714	7.4	2.68	23.04
154.6286	7.4	2.69	23.07
154.6857	7.39	2.69	23.09
154.7429	7.37	2.7	23.1
154.8	7.38	2.68	23.08
154.8571	7.39	2.68	23.06
154.9143	7.39	2.67	23.06
154.9714	7.41	2.66	23.07
155.0286	7.38	2.69	23.04
155.0857	7.36	2.68	23.04
155.1429	7.37	2.62	23.04
155.2	7.37	2.62	23
155.2571	7.37	2.62	22.99
155.3143	7.35	2.63	22.95
155.3714	7.36	2.66	22.92
155.4286	7.34	2.67	22.88
155.4857	7.34	2.69	22.83
155.5429	7.35	2.69	22.8
155.6	7.35	2.65	22.77

155.6571	7.33	2.65	22.75
155.7143	7.31	2.68	22.74
155.7714	7.33	2.71	22.78
155.8286	7.36	2.71	22.8
155.8857	7.37	2.7	22.83
155.9429	7.36	2.71	22.85
156	7.39	2.72	22.85
156.0571	7.37	2.7	22.84
156.1143	7.4	2.71	22.87
156.1714	7.43	2.71	22.89
156.2286	7.48	2.7	22.89
156.2857	7.52	2.68	22.92
156.3429	7.53	2.66	22.94
156.4	7.52	2.66	22.99
156.4571	7.5	2.71	23.01
156.5143	7.51	2.68	23.03
156.5714	7.51	2.67	23.05
156.6286	7.49	2.68	23.07
156.6857	7.5	2.67	23.06
156.7429	7.5	2.68	23.06
156.8	7.49	2.68	23.02
156.8571	7.46	2.7	23.01
156.9143	7.45	2.71	23.01
156.9714	7.45	2.72	23.01
157.0286	7.46	2.71	22.98
157.0857	7.47	2.7	22.98
157.1429	7.44	2.7	22.96
157.2	7.48	2.68	22.97
157.2571	7.47	2.69	22.96
157.3143	7.44	2.7	22.92
157.3714	7.42	2.69	22.9
157.4286	7.46	2.69	22.88
157.4857	7.48	2.69	22.85
157.5429	7.48	2.71	22.82
157.6	7.46	2.71	22.8
157.6571	7.46	2.76	22.79
157.7143	7.47	2.75	22.81
157.7714	7.48	2.78	22.83
157.8286	7.48	2.77	22.83
157.8857	7.53	2.72	22.82
157.9429	7.5	2.75	22.84
158	7.46	2.75	22.84
158.0571	7.45	2.77	22.85
158.1143	7.43	2.75	22.86
158.1714	7.42	2.74	22.84
158.2286	7.41	2.73	22.88
158.2857	7.39	2.74	22.92

158.3429	7.41	2.74	22.93
158.4	7.44	2.73	22.95
158.4571	7.42	2.78	22.99
158.5143	7.44	2.77	23.02
158.5714	7.45	2.74	23.02
158.6286	7.48	2.73	23
158.6857	7.48	2.72	22.96
158.7429	7.51	2.71	22.95
158.8	7.53	2.7	22.94
158.8571	7.54	2.7	22.96
158.9143	7.52	2.7	22.96
158.9714	7.48	2.73	22.96
159.0286	7.47	2.74	22.96
159.0857	7.49	2.74	22.94
159.1429	7.47	2.76	22.94
159.2	7.46	2.79	22.91
159.2571	7.47	2.8	22.89
159.3143	7.47	2.8	22.89
159.3714	7.49	2.8	22.89
159.4286	7.48	2.8	22.9
159.4857	7.5	2.78	22.84
159.5429	7.53	2.76	22.83
159.6	7.57	2.73	22.85
159.6571	7.58	2.71	22.88
159.7143	7.55	2.71	22.9
159.7714	7.55	2.73	22.92
159.8286	7.57	2.73	22.93
159.8857	7.56	2.74	22.94
159.9429	7.52	2.76	22.95
160	7.5	2.78	22.95
160.0571	7.5	2.79	22.96
160.1143	7.5	2.84	23.01
160.1714	7.47	2.85	23.06
160.2286	7.45	2.87	23.08
160.2857	7.44	2.87	23.12
160.3429	7.45	2.88	23.14
160.4	7.48	2.85	23.17
160.4571	7.49	2.85	23.21
160.5143	7.49	2.83	23.22
160.5714	7.55	2.81	23.22
160.6286	7.56	2.79	23.21
160.6857	7.56	2.78	23.19
160.7429	7.56	2.79	23.18
160.8	7.56	2.77	23.17
160.8571	7.57	2.77	23.16
160.9143	7.58	2.77	23.16
160.9714	7.57	2.77	23.17

161.0286	7.59	2.73	23.14
161.0857	7.59	2.76	23.1
161.1429	7.57	2.77	23.05
161.2	7.58	2.78	23.02
161.2571	7.57	2.76	22.98
161.3143	7.57	2.75	22.97
161.3714	7.59	2.76	22.96
161.4286	7.59	2.73	22.96
161.4857	7.59	2.72	22.93
161.5429	7.56	2.72	22.92
161.6	7.54	2.77	22.94
161.6571	7.53	2.76	22.96
161.7143	7.54	2.76	23.01
161.7714	7.52	2.77	23.03
161.8286	7.51	2.79	23.06
161.8857	7.52	2.78	23.07
161.9429	7.51	2.81	23.07
162	7.53	2.83	23.1
162.0571	7.52	2.86	23.13
162.1143	7.53	2.85	23.16
162.1714	7.56	2.83	23.18
162.2286	7.61	2.8	23.22
162.2857	7.61	2.81	23.24
162.3429	7.61	2.81	23.25
162.4	7.64	2.79	23.28
162.4571	7.65	2.8	23.32
162.5143	7.67	2.76	23.33
162.5714	7.64	2.75	23.3
162.6286	7.65	2.74	23.29
162.6857	7.66	2.74	23.28
162.7429	7.63	2.76	23.25
162.8	7.61	2.8	23.25
162.8571	7.59	2.79	23.24
162.9143	7.58	2.81	23.23
162.9714	7.56	2.81	23.2
163.0286	7.58	2.8	23.17
163.0857	7.57	2.81	23.17
163.1429	7.58	2.81	23.14
163.2	7.6	2.78	23.12
163.2571	7.58	2.79	23.09
163.3143	7.58	2.76	23.08
163.3714	7.61	2.77	23.04
163.4286	7.63	2.76	22.99
163.4857	7.66	2.75	22.98
163.5429	7.69	2.73	22.97
163.6	7.67	2.75	22.97
163.6571	7.68	2.75	22.99

163.7143	7.68	2.76	22.98
163.7714	7.65	2.77	23.01
163.8286	7.67	2.8	23.05
163.8857	7.69	2.79	23.06
163.9429	7.7	2.78	23.09
164	7.68	2.77	23.13
164.0571	7.68	2.77	23.13
164.1143	7.64	2.78	23.15
164.1714	7.65	2.76	23.2
164.2286	7.62	2.74	23.21
164.2857	7.64	2.73	23.22
164.3429	7.64	2.74	23.27
164.4	7.64	2.73	23.27
164.4571	7.61	2.78	23.29
164.5143	7.57	2.8	23.34
164.5714	7.57	2.82	23.33
164.6286	7.56	2.81	23.35
164.6857	7.57	2.8	23.36
164.7429	7.55	2.8	23.32
164.8	7.56	2.82	23.29
164.8571	7.56	2.83	23.28
164.9143	7.54	2.86	23.28
164.9714	7.56	2.86	23.27
165.0286	7.57	2.82	23.27
165.0857	7.57	2.81	23.25
165.1429	7.57	2.79	23.21
165.2	7.57	2.79	23.2
165.2571	7.56	2.8	23.16
165.3143	7.55	2.81	23.16
165.3714	7.54	2.83	23.15
165.4286	7.52	2.84	23.14
165.4857	7.54	2.82	23.11
165.5429	7.51	2.84	23.09
165.6	7.53	2.87	23.05
165.6571	7.54	2.86	23.04
165.7143	7.56	2.85	23.06
165.7714	7.58	2.86	23.07
165.8286	7.61	2.86	23.07
165.8857	7.62	2.87	23.08
165.9429	7.66	2.85	23.1
166	7.68	2.86	23.14
166.0571	7.69	2.87	23.15
166.1143	7.73	2.84	23.17
166.1714	7.73	2.83	23.21
166.2286	7.72	2.82	23.26
166.2857	7.75	2.85	23.29
166.3429	7.76	2.83	23.3

166.4	7.75	2.83	23.31
166.4571	7.72	2.85	23.32
166.5143	7.69	2.86	23.34
166.5714	7.68	2.87	23.35
166.6286	7.68	2.85	23.35
166.6857	7.66	2.87	23.36
166.7429	7.65	2.88	23.37
166.8	7.62	2.89	23.35
166.8571	7.56	2.89	23.32
166.9143	7.54	2.92	23.32
166.9714	7.54	2.93	23.3
167.0286	7.58	2.94	23.27
167.0857	7.61	2.93	23.24
167.1429	7.62	2.91	23.21
167.2	7.61	2.94	23.18
167.2571	7.59	2.93	23.16
167.3143	7.59	2.92	23.13
167.3714	7.62	2.93	23.12
167.4286	7.62	2.96	23.09
167.4857	7.64	2.97	23.09
167.5429	7.66	2.97	23.08
167.6	7.65	2.92	23.1
167.6571	7.65	2.93	23.1
167.7143	7.65	2.95	23.1
167.7714	7.67	2.94	23.14
167.8286	7.68	2.95	23.18
167.8857	7.7	2.95	23.2
167.9429	7.7	2.93	23.22
168	7.72	2.89	23.25
168.0571	7.72	2.86	23.27
168.1143	7.72	2.84	23.31
168.1714	7.71	2.88	23.33
168.2286	7.72	2.86	23.35
168.2857	7.7	2.87	23.38
168.3429	7.68	2.87	23.4
168.4	7.7	2.86	23.42
168.4571	7.69	2.88	23.43
168.5143	7.68	2.9	23.45
168.5714	7.68	2.94	23.43
168.6286	7.67	2.95	23.42
168.6857	7.68	2.97	23.4
168.7429	7.7	2.93	23.35
168.8	7.73	2.93	23.32
168.8571	7.74	2.92	23.3
168.9143	7.75	2.94	23.3
168.9714	7.72	2.94	23.27
169.0286	7.72	2.93	23.24

169.0857	7.72	2.92	23.22
169.1429	7.75	2.9	23.18
169.2	7.78	2.91	23.16
169.2571	7.78	2.92	23.14
169.3143	7.74	2.96	23.1
169.3714	7.71	2.98	23.09
169.4286	7.71	2.99	23.07
169.4857	7.69	2.96	23.07
169.5429	7.71	2.95	23.1
169.6	7.74	2.96	23.12
169.6571	7.74	2.96	23.15
169.7143	7.71	2.94	23.19
169.7714	7.7	2.94	23.21
169.8286	7.71	2.9	23.21
169.8857	7.76	2.89	23.24
169.9429	7.78	2.86	23.28
170	7.79	2.85	23.29
170.0571	7.83	2.85	23.3
170.1143	7.81	2.84	23.32
170.1714	7.82	2.81	23.37
170.2286	7.83	2.79	23.41
170.2857	7.85	2.81	23.43
170.3429	7.84	2.8	23.44
170.4	7.82	2.85	23.46
170.4571	7.82	2.85	23.45
170.5143	7.8	2.87	23.44
170.5714	7.76	2.87	23.42
170.6286	7.74	2.87	23.38
170.6857	7.76	2.87	23.37
170.7429	7.74	2.91	23.38
170.8	7.71	2.94	23.39
170.8571	7.68	2.97	23.37
170.9143	7.69	2.98	23.37
170.9714	7.7	2.96	23.38
171.0286	7.67	2.97	23.39
171.0857	7.67	2.97	23.39
171.1429	7.7	2.97	23.33
171.2	7.68	2.99	23.31
171.2571	7.69	3.01	23.3
171.3143	7.73	2.99	23.26
171.3714	7.74	3.01	23.24
171.4286	7.75	2.99	23.24
171.4857	7.8	2.95	23.24
171.5429	7.79	2.95	23.27
171.6	7.8	2.96	23.28
171.6571	7.82	2.98	23.28
171.7143	7.81	3.01	23.29

171.7714	7.84	2.96	23.3
171.8286	7.85	2.96	23.33
171.8857	7.84	2.96	23.33
171.9429	7.81	2.96	23.34
172	7.83	2.95	23.36
172.0571	7.78	2.96	23.37
172.1143	7.79	2.98	23.42
172.1714	7.82	2.93	23.44
172.2286	7.83	2.9	23.47
172.2857	7.81	2.89	23.48
172.3429	7.83	2.9	23.49
172.4	7.84	2.89	23.51
172.4571	7.85	2.9	23.49
172.5143	7.86	2.88	23.47
172.5714	7.85	2.88	23.48
172.6286	7.82	2.91	23.47
172.6857	7.82	2.89	23.43
172.7429	7.79	2.94	23.41
172.8	7.78	2.95	23.39
172.8571	7.77	2.95	23.38
172.9143	7.76	2.96	23.34
172.9714	7.72	2.99	23.34
173.0286	7.71	3.01	23.33
173.0857	7.69	3.01	23.31
173.1429	7.71	3.04	23.3
173.2	7.71	3.04	23.3
173.2571	7.73	3.04	23.29
173.3143	7.75	3	23.29
173.3714	7.75	3.04	23.28
173.4286	7.77	3.04	23.29
173.4857	7.76	3.05	23.29
173.5429	7.81	3.02	23.3
173.6	7.81	2.98	23.33
173.6571	7.83	2.99	23.37
173.7143	7.85	2.95	23.39
173.7714	7.86	2.94	23.42
173.8286	7.86	2.94	23.45
173.8857	7.84	2.99	23.46
173.9429	7.86	2.94	23.48
174	7.86	2.93	23.5
174.0571	7.87	2.92	23.52
174.1143	7.83	2.94	23.53
174.1714	7.83	2.97	23.55
174.2286	7.83	2.95	23.56
174.2857	7.81	2.97	23.56
174.3429	7.79	2.98	23.57
174.4	7.8	3	23.6

174.4571	7.82	3	23.59
174.5143	7.85	3.02	23.55
174.5714	7.85	3.01	23.56
174.6286	7.83	3.04	23.56
174.6857	7.85	3.02	23.54
174.7429	7.85	3.03	23.52
174.8	7.83	3.05	23.49
174.8571	7.84	3.04	23.5
174.9143	7.86	3.02	23.48
174.9714	7.87	3.01	23.46
175.0286	7.86	3	23.46
175.0857	7.85	2.98	23.44
175.1429	7.83	3.02	23.45
175.2	7.86	2.99	23.44
175.2571	7.84	3	23.44
175.3143	7.84	3.01	23.43
175.3714	7.85	3	23.42
175.4286	7.88	3	23.45
175.4857	7.86	3.01	23.46
175.5429	7.87	3.01	23.45
175.6	7.89	2.99	23.45
175.6571	7.89	3	23.49
175.7143	7.89	2.96	23.51
175.7714	7.95	2.97	23.53
175.8286	7.94	2.96	23.52
175.8857	7.96	2.94	23.54
175.9429	7.96	2.93	23.55
176	7.95	2.93	23.56
176.0571	7.95	2.91	23.57
176.1143	7.97	2.93	23.59
176.1714	7.95	2.93	23.61
176.2286	7.97	2.92	23.62
176.2857	7.97	2.94	23.64
176.3429	7.94	2.99	23.63
176.4	7.94	2.98	23.64
176.4571	7.96	2.98	23.61
176.5143	7.96	3.01	23.59
176.5714	7.95	3	23.59
176.6286	7.95	3.02	23.59
176.6857	7.94	3.01	23.57
176.7429	7.94	3.02	23.56
176.8	7.93	3.04	23.55
176.8571	7.89	3.07	23.52
176.9143	7.88	3.03	23.5
176.9714	7.9	3.04	23.46
177.0286	7.87	3.05	23.42
177.0857	7.91	3.04	23.39

177.1429	7.88	3.05	23.36
177.2	7.9	3.06	23.34
177.2571	7.9	3.07	23.31
177.3143	7.9	3.07	23.31
177.3714	7.94	3.08	23.31
177.4286	7.97	3.08	23.33
177.4857	8	3.09	23.34
177.5429	8.02	3.07	23.33
177.6	8.06	3.06	23.33
177.6571	8.07	3.02	23.37
177.7143	8.09	3.01	23.39
177.7714	8.11	3	23.42
177.8286	8.12	3	23.47
177.8857	8.1	2.99	23.5
177.9429	8.1	2.97	23.54
178	8.06	2.99	23.56
178.0571	8.04	3.01	23.59
178.1143	8.03	3.04	23.62
178.1714	7.98	3.06	23.64
178.2286	7.93	3.11	23.66
178.2857	7.89	3.15	23.66
178.3429	7.89	3.15	23.66
178.4	7.87	3.16	23.64
178.4571	7.9	3.14	23.63
178.5143	7.89	3.14	23.63
178.5714	7.89	3.12	23.62
178.6286	7.91	3.12	23.58
178.6857	7.91	3.1	23.55
178.7429	7.9	3.1	23.53
178.8	7.93	3.09	23.52
178.8571	7.95	3.06	23.5
178.9143	7.97	3.06	23.49
178.9714	7.97	3.05	23.48
179.0286	7.96	3.07	23.48
179.0857	7.96	3.1	23.45
179.1429	7.95	3.08	23.43
179.2	7.98	3.06	23.41
179.2571	7.99	3.07	23.41
179.3143	8	3.05	23.41
179.3714	7.98	3.05	23.42
179.4286	7.99	3.04	23.45
179.4857	7.97	3.03	23.46
179.5429	7.98	3.07	23.49
179.6	7.98	3.06	23.52
179.6571	8	3.05	23.55
179.7143	7.99	3.07	23.55
179.7714	7.99	3.06	23.58

179.8286	8	3.04	23.62
179.8857	8.04	3.05	23.62
179.9429	8.07	3.05	23.65
180	8.06	3.05	23.65
180.0571	8.08	3.04	23.68
180.1143	8.08	3.01	23.69
180.1714	8.06	3.02	23.73
180.2286	8.02	3.06	23.74
180.2857	8.04	3.06	23.75
180.3429	8.03	3.07	23.75
180.4	8.02	3.1	23.73
180.4571	8	3.1	23.71
180.5143	7.99	3.07	23.7
180.5714	8	3.07	23.7
180.6286	8.01	3.07	23.7
180.6857	8.01	3.09	23.7
180.7429	8.01	3.08	23.68
180.8	8.05	3.07	23.68
180.8571	8.05	3.06	23.67
180.9143	8.07	3.06	23.64
180.9714	8.05	3.07	23.63
181.0286	8.06	3.08	23.62
181.0857	8.05	3.1	23.59
181.1429	8.03	3.13	23.54
181.2	8.01	3.16	23.54
181.2571	8.02	3.16	23.52
181.3143	8.02	3.17	23.52
181.3714	8.02	3.14	23.54
181.4286	8.03	3.13	23.55
181.4857	8.02	3.14	23.56
181.5429	8.05	3.13	23.55
181.6	8.04	3.13	23.55
181.6571	8.05	3.13	23.59
181.7143	8.05	3.11	23.59
181.7714	8.06	3.08	23.6
181.8286	8.07	3.07	23.62
181.8857	8.08	3.04	23.64
181.9429	8.08	3.06	23.68
182	8.06	3.06	23.71
182.0571	8.04	3.07	23.76
182.1143	8.03	3.06	23.8
182.1714	8.03	3.04	23.8
182.2286	7.99	3.05	23.79
182.2857	8.01	3.09	23.79
182.3429	8.01	3.07	23.8
182.4	8.04	3.07	23.78
182.4571	8.04	3.08	23.79

182.5143	8.03	3.08	23.76
182.5714	8.05	3.1	23.73
182.6286	8.05	3.11	23.71
182.6857	8.01	3.13	23.7
182.7429	8.02	3.15	23.66
182.8	8.05	3.15	23.65
182.8571	8.06	3.12	23.63
182.9143	8.08	3.13	23.59
182.9714	8.05	3.16	23.58
183.0286	8.08	3.14	23.53
183.0857	8.06	3.15	23.52
183.1429	8.05	3.16	23.51
183.2	8.08	3.15	23.53
183.2571	8.12	3.14	23.54
183.3143	8.13	3.13	23.54
183.3714	8.15	3.13	23.57
183.4286	8.13	3.13	23.61
183.4857	8.16	3.12	23.65
183.5429	8.15	3.13	23.66
183.6	8.14	3.15	23.68
183.6571	8.13	3.13	23.74
183.7143	8.12	3.13	23.78
183.7714	8.12	3.12	23.8
183.8286	8.11	3.12	23.82
183.8857	8.12	3.1	23.86
183.9429	8.13	3.1	23.88
184	8.12	3.15	23.92
184.0571	8.1	3.16	23.94
184.1143	8.11	3.15	23.95
184.1714	8.07	3.14	23.93
184.2286	8.09	3.14	23.93
184.2857	8.1	3.14	23.92
184.3429	8.09	3.17	23.88
184.4	8.07	3.15	23.86
184.4571	8.06	3.19	23.85
184.5143	8.07	3.2	23.83
184.5714	8.06	3.2	23.81
184.6286	8.04	3.23	23.76
184.6857	8.01	3.24	23.73
184.7429	8.05	3.24	23.69
184.8	8.03	3.27	23.64
184.8571	8.03	3.27	23.62
184.9143	8.01	3.25	23.61
184.9714	8.02	3.26	23.58
185.0286	8.02	3.23	23.53
185.0857	8.01	3.23	23.51
185.1429	7.99	3.23	23.51

185.2	7.99	3.21	23.51
185.2571	8.01	3.21	23.52
185.3143	8	3.23	23.53
185.3714	7.99	3.23	23.55
185.4286	8.01	3.23	23.6
185.4857	8.03	3.25	23.62
185.5429	8.04	3.22	23.64
185.6	8.03	3.21	23.67
185.6571	8.04	3.17	23.72
185.7143	8.03	3.16	23.75
185.7714	8.05	3.13	23.77
185.8286	8.07	3.14	23.82
185.8857	8.11	3.08	23.85
185.9429	8.11	3.07	23.88
186	8.11	3.06	23.92
186.0571	8.14	3.04	23.91
186.1143	8.12	3.06	23.91
186.1714	8.12	3.07	23.9
186.2286	8.11	3.09	23.9
186.2857	8.14	3.08	23.89
186.3429	8.14	3.08	23.85
186.4	8.12	3.06	23.81
186.4571	8.1	3.08	23.8
186.5143	8.12	3.06	23.77
186.5714	8.11	3.06	23.75
186.6286	8.08	3.07	23.73
186.6857	8.1	3.08	23.73
186.7429	8.14	3.06	23.71
186.8	8.16	3.08	23.68
186.8571	8.16	3.08	23.67
186.9143	8.16	3.11	23.66
186.9714	8.16	3.1	23.67
187.0286	8.17	3.09	23.67
187.0857	8.16	3.1	23.68
187.1429	8.16	3.12	23.69
187.2	8.19	3.13	23.69
187.2571	8.15	3.16	23.72
187.3143	8.14	3.16	23.76
187.3714	8.11	3.19	23.79
187.4286	8.11	3.18	23.82
187.4857	8.09	3.19	23.85
187.5429	8.09	3.21	23.87
187.6	8.07	3.25	23.85
187.6571	8.05	3.26	23.88
187.7143	8.07	3.25	23.92
187.7714	8.06	3.26	23.95
187.8286	8.08	3.24	23.99

187.8857	8.11	3.24	23.98
187.9429	8.12	3.22	23.99
188	8.13	3.24	24.02
188.0571	8.14	3.2	24
188.1143	8.13	3.23	23.98
188.1714	8.13	3.21	23.97
188.2286	8.15	3.21	23.94
188.2857	8.15	3.21	23.92
188.3429	8.14	3.2	23.91
188.4	8.14	3.17	23.86
188.4571	8.15	3.18	23.86
188.5143	8.13	3.16	23.85
188.5714	8.12	3.14	23.85
188.6286	8.11	3.17	23.82
188.6857	8.12	3.15	23.78
188.7429	8.15	3.17	23.75
188.8	8.14	3.18	23.73
188.8571	8.13	3.19	23.73
188.9143	8.15	3.19	23.69
188.9714	8.14	3.21	23.67
189.0286	8.15	3.19	23.66
189.0857	8.17	3.21	23.67
189.1429	8.18	3.22	23.69
189.2	8.19	3.21	23.7
189.2571	8.21	3.18	23.71
189.3143	8.23	3.16	23.73
189.3714	8.27	3.15	23.77
189.4286	8.26	3.13	23.78
189.4857	8.25	3.12	23.79
189.5429	8.23	3.15	23.8
189.6	8.23	3.16	23.81
189.6571	8.26	3.15	23.85
189.7143	8.27	3.13	23.85
189.7714	8.25	3.16	23.88
189.8286	8.24	3.18	23.9
189.8857	8.25	3.2	23.94
189.9429	8.21	3.21	23.96
190	8.22	3.19	23.98
190.0571	8.23	3.21	23.96
190.1143	8.24	3.19	23.97
190.1714	8.23	3.21	23.95
190.2286	8.2	3.22	23.95
190.2857	8.2	3.23	23.95
190.3429	8.24	3.23	23.95
190.4	8.25	3.22	23.95
190.4571	8.26	3.19	23.94
190.5143	8.29	3.17	23.91

190.5714	8.3	3.16	23.9
190.6286	8.31	3.17	23.88
190.6857	8.28	3.18	23.88
190.7429	8.26	3.18	23.86
190.8	8.27	3.17	23.83
190.8571	8.27	3.18	23.82
190.9143	8.24	3.17	23.79
190.9714	8.21	3.19	23.78
191.0286	8.17	3.21	23.78
191.0857	8.15	3.22	23.78
191.1429	8.16	3.24	23.78
191.2	8.16	3.23	23.78
191.2571	8.18	3.23	23.79
191.3143	8.2	3.2	23.8
191.3714	8.18	3.22	23.82
191.4286	8.17	3.2	23.84
191.4857	8.16	3.21	23.89
191.5429	8.18	3.2	23.89
191.6	8.22	3.2	23.91
191.6571	8.24	3.2	23.95
191.7143	8.23	3.22	23.98
191.7714	8.21	3.22	24.01
191.8286	8.21	3.22	24.03
191.8857	8.24	3.22	24.05
191.9429	8.26	3.22	24.06
192	8.29	3.22	24.07
192.0571	8.32	3.21	24.08
192.1143	8.35	3.19	24.08
192.1714	8.34	3.18	24.07
192.2286	8.34	3.19	24.03
192.2857	8.34	3.17	24.02
192.3429	8.35	3.16	24.02
192.4	8.34	3.13	24.01
192.4571	8.34	3.12	23.98
192.5143	8.31	3.12	23.98
192.5714	8.3	3.14	23.92
192.6286	8.27	3.17	23.9
192.6857	8.25	3.17	23.87
192.7429	8.25	3.17	23.85
192.8	8.27	3.17	23.84
192.8571	8.27	3.19	23.83
192.9143	8.26	3.2	23.81
192.9714	8.27	3.23	23.78
193.0286	8.27	3.25	23.78
193.0857	8.28	3.26	23.79
193.1429	8.28	3.23	23.82
193.2	8.3	3.21	23.84

193.2571	8.3	3.23	23.86
193.3143	8.27	3.24	23.86
193.3714	8.26	3.24	23.88
193.4286	8.26	3.26	23.9
193.4857	8.23	3.27	23.94
193.5429	8.25	3.28	23.97
193.6	8.29	3.28	23.98
193.6571	8.29	3.26	24.01
193.7143	8.29	3.26	24.03
193.7714	8.29	3.26	24.03
193.8286	8.27	3.28	24.06
193.8857	8.27	3.27	24.07
193.9429	8.26	3.26	24.07
194	8.28	3.26	24.08
194.0571	8.28	3.26	24.09
194.1143	8.31	3.25	24.07
194.1714	8.34	3.24	24.05
194.2286	8.32	3.24	24.03
194.2857	8.32	3.24	24.03
194.3429	8.33	3.25	24.04
194.4	8.33	3.26	24.02
194.4571	8.31	3.27	24
194.5143	8.32	3.27	23.98
194.5714	8.34	3.26	23.97
194.6286	8.34	3.29	23.93
194.6857	8.32	3.29	23.91
194.7429	8.29	3.3	23.9
194.8	8.28	3.3	23.89
194.8571	8.24	3.31	23.87
194.9143	8.24	3.3	23.86
194.9714	8.25	3.3	23.86
195.0286	8.26	3.31	23.86
195.0857	8.26	3.3	23.9
195.1429	8.25	3.33	23.93
195.2	8.24	3.31	23.94
195.2571	8.26	3.3	23.94
195.3143	8.29	3.26	23.94
195.3714	8.31	3.25	23.97
195.4286	8.33	3.26	23.98
195.4857	8.33	3.26	24
195.5429	8.3	3.28	24.02
195.6	8.33	3.28	24.03
195.6571	8.3	3.34	24.04
195.7143	8.29	3.31	24.04
195.7714	8.28	3.32	24.06
195.8286	8.27	3.33	24.09
195.8857	8.26	3.35	24.12

195.9429	8.26	3.38	24.12
196	8.25	3.36	24.12
196.0571	8.26	3.37	24.1
196.1143	8.3	3.33	24.1
196.1714	8.3	3.35	24.09
196.2286	8.33	3.31	24.09
196.2857	8.36	3.35	24.07
196.3429	8.36	3.34	24.05
196.4	8.36	3.34	24.04
196.4571	8.34	3.37	24.03
196.5143	8.35	3.37	24.03
196.5714	8.38	3.38	24.01
196.6286	8.35	3.4	23.98
196.6857	8.34	3.44	23.98
196.7429	8.34	3.42	23.94
196.8	8.33	3.43	23.95
196.8571	8.35	3.41	23.93
196.9143	8.36	3.41	23.93
196.9714	8.35	3.42	23.89
197.0286	8.37	3.41	23.83
197.0857	8.38	3.38	23.81
197.1429	8.38	3.35	23.78
197.2	8.39	3.37	23.75
197.2571	8.4	3.35	23.73
197.3143	8.39	3.32	23.72
197.3714	8.39	3.3	23.66
197.4286	8.38	3.28	23.63
197.4857	8.35	3.27	23.63
197.5429	8.39	3.22	23.63
197.6	8.36	3.24	23.59
197.6571	8.36	3.27	23.56
197.7143	8.38	3.28	23.54
197.7714	8.37	3.26	23.47
197.8286	8.36	3.27	23.43
197.8857	8.37	3.3	23.39
197.9429	8.36	3.32	23.38
198	8.38	3.31	23.37
198.0571	8.4	3.3	23.35
198.1143	8.36	3.34	23.29
198.1714	8.38	3.34	23.25
198.2286	8.4	3.31	23.22
198.2857	8.4	3.31	23.22
198.3429	8.4	3.3	23.23
198.4	8.4	3.29	23.21
198.4571	8.41	3.3	23.15
198.5143	8.4	3.31	23.12
198.5714	8.38	3.34	23.12

198.6286	8.37	3.33	23.09
198.6857	8.37	3.31	23.06
198.7429	8.37	3.29	23.03
198.8	8.36	3.29	23
198.8571	8.36	3.28	22.96
198.9143	8.38	3.28	22.92
198.9714	8.36	3.29	22.9
199.0286	8.37	3.27	22.89
199.0857	8.39	3.26	22.85
199.1429	8.37	3.29	22.83
199.2	8.38	3.29	22.78
199.2571	8.38	3.31	22.71
199.3143	8.38	3.34	22.65
199.3714	8.38	3.32	22.63
199.4286	8.35	3.36	22.6
199.4857	8.34	3.39	22.52
199.5429	8.34	3.38	22.5
199.6	8.31	3.38	22.47
199.6571	8.28	3.4	22.43
199.7143	8.31	3.35	22.41
199.7714	8.32	3.36	22.37
199.8286	8.29	3.37	22.34
199.8857	8.3	3.36	22.29
199.9429	8.31	3.37	22.27
200	8.33	3.35	22.25
200.0572	8.31	3.35	22.22
200.1142	8.33	3.33	22.18
200.1714	8.35	3.32	22.17
200.2286	8.36	3.27	22.13
200.2858	8.33	3.31	22.09
200.3428	8.3	3.32	22.04
200.4	8.34	3.3	22.01
200.4572	8.35	3.3	21.98
200.5142	8.33	3.31	21.92
200.5714	8.29	3.35	21.89
200.6286	8.28	3.37	21.87
200.6858	8.27	3.38	21.84
200.7428	8.25	3.42	21.8
200.8	8.23	3.44	21.77
200.8572	8.24	3.41	21.74
200.9142	8.27	3.4	21.69
200.9714	8.27	3.39	21.65
201.0286	8.26	3.4	21.62
201.0858	8.27	3.38	21.61
201.1428	8.29	3.35	21.59
201.2	8.32	3.33	21.55
201.2572	8.3	3.35	21.53

201.3142	8.31	3.34	21.5
201.3714	8.32	3.35	21.48
201.4286	8.34	3.37	21.44
201.4858	8.31	3.38	21.41
201.5428	8.32	3.41	21.37
201.6	8.34	3.39	21.36
201.6572	8.32	3.39	21.34
201.7142	8.31	3.38	21.31
201.7714	8.31	3.37	21.28
201.8286	8.29	3.36	21.23
201.8858	8.3	3.34	21.18
201.9428	8.31	3.34	21.13
202	8.29	3.32	21.11
202.0572	8.29	3.33	21.06
202.1142	8.28	3.31	21.01
202.1714	8.28	3.32	20.97
202.2286	8.26	3.34	20.95
202.2858	8.25	3.34	20.93
202.3428	8.27	3.36	20.93
202.4	8.27	3.35	20.92
202.4572	8.26	3.36	20.88
202.5142	8.23	3.38	20.83
202.5714	8.24	3.38	20.78
202.6286	8.25	3.35	20.73
202.6858	8.25	3.36	20.69
202.7428	8.26	3.37	20.67
202.8	8.31	3.35	20.64
202.8572	8.35	3.34	20.62
202.9142	8.35	3.32	20.59
202.9714	8.34	3.33	20.57
203.0286	8.36	3.32	20.54
203.0858	8.38	3.28	20.55
203.1428	8.36	3.29	20.52
203.2	8.36	3.28	20.49
203.2572	8.34	3.29	20.44
203.3142	8.34	3.29	20.41
203.3714	8.31	3.3	20.36
203.4286	8.3	3.32	20.33
203.4858	8.27	3.35	20.31
203.5428	8.27	3.36	20.29
203.6	8.23	3.37	20.25
203.6572	8.25	3.38	20.22
203.7142	8.27	3.38	20.17
203.7714	8.27	3.4	20.12
203.8286	8.3	3.39	20.1
203.8858	8.31	3.39	20.05
203.9428	8.3	3.37	20

204	8.3	3.39	19.95
204.0572	8.32	3.38	19.92
204.1142	8.32	3.37	19.88
204.1714	8.33	3.36	19.84
204.2286	8.32	3.36	19.78
204.2858	8.31	3.37	19.75
204.3428	8.31	3.35	19.71
204.4	8.34	3.36	19.69
204.4572	8.33	3.34	19.65
204.5142	8.32	3.37	19.63
204.5714	8.33	3.35	19.59
204.6286	8.35	3.34	19.58
204.6858	8.37	3.31	19.58
204.7428	8.4	3.32	19.54
204.8	8.41	3.34	19.49
204.8572	8.4	3.33	19.46
204.9142	8.39	3.35	19.43
204.9714	8.35	3.34	19.41
205.0286	8.34	3.35	19.39
205.0858	8.34	3.33	19.38
205.1428	8.34	3.36	19.35
205.2	8.31	3.35	19.32
205.2572	8.28	3.39	19.3
205.3142	8.26	3.4	19.29
205.3714	8.24	3.4	19.26
205.4286	8.24	3.41	19.22
205.4858	8.24	3.41	19.19
205.5428	8.25	3.42	19.2
205.6	8.27	3.42	19.19
205.6572	8.29	3.44	19.17
205.7142	8.31	3.42	19.15
205.7714	8.34	3.41	19.12
205.8286	8.3	3.42	19.09
205.8858	8.29	3.41	19.06
205.9428	8.3	3.41	19.04
206	8.31	3.39	19
206.0572	8.32	3.38	18.97
206.1142	8.34	3.36	18.96
206.1714	8.32	3.36	18.95
206.2286	8.29	3.37	18.91
206.2858	8.26	3.36	18.9
206.3428	8.24	3.38	18.89
206.4	8.28	3.37	18.85
206.4572	8.3	3.36	18.82
206.5142	8.32	3.35	18.78
206.5714	8.33	3.37	18.76
206.6286	8.32	3.36	18.75

206.6858	8.31	3.37	18.71
206.7428	8.33	3.36	18.68
206.8	8.36	3.37	18.66
206.8572	8.34	3.39	18.63
206.9142	8.33	3.38	18.63
206.9714	8.31	3.35	18.61
207.0286	8.3	3.34	18.58
207.0858	8.28	3.33	18.54
207.1428	8.28	3.32	18.49
207.2	8.27	3.37	18.46
207.2572	8.25	3.38	18.44
207.3142	8.2	3.41	18.43
207.3714	8.17	3.39	18.41
207.4286	8.2	3.4	18.39
207.4858	8.24	3.38	18.33
207.5428	8.27	3.38	18.28
207.6	8.26	3.41	18.27
207.6572	8.25	3.46	18.25
207.7142	8.25	3.42	18.23
207.7714	8.25	3.4	18.21
207.8286	8.27	3.39	18.17
207.8858	8.28	3.39	18.15
207.9428	8.3	3.39	18.14
208	8.29	3.37	18.14
208.0572	8.25	3.4	18.12
208.1142	8.21	3.41	18.09
208.1714	8.22	3.38	18.07
208.2286	8.2	3.36	18.03
208.2858	8.17	3.39	18.01
208.3428	8.17	3.4	17.98
208.4	8.17	3.41	17.99
208.4572	8.17	3.4	17.98
208.5142	8.16	3.39	17.95
208.5714	8.16	3.4	17.93
208.6286	8.16	3.4	17.9
208.6858	8.18	3.4	17.87
208.7428	8.19	3.42	17.84
208.8	8.22	3.44	17.78
208.8572	8.22	3.46	17.73
208.9142	8.24	3.45	17.68
208.9714	8.22	3.42	17.67
209.0286	8.21	3.42	17.66
209.0858	8.19	3.44	17.62
209.1428	8.2	3.42	17.6
209.2	8.22	3.41	17.58
209.2572	8.18	3.41	17.55
209.3142	8.14	3.47	17.52

209.3714	8.09	3.46	17.51
209.4286	8.12	3.45	17.49
209.4858	8.15	3.45	17.47
209.5428	8.12	3.48	17.46
209.6	8.1	3.48	17.43
209.6572	8.12	3.47	17.42
209.7142	8.1	3.49	17.4
209.7714	8.11	3.48	17.38
209.8286	8.1	3.5	17.38
209.8858	8.11	3.45	17.35
209.9428	8.16	3.41	17.33
210	8.14	3.4	17.32
210.0572	8.11	3.41	17.31
210.1142	8.11	3.41	17.25
210.1714	8.1	3.43	17.22
210.2286	8.13	3.41	17.21
210.2858	8.16	3.38	17.16
210.3428	8.14	3.4	17.11
210.4	8.16	3.38	17.11
210.4572	8.2	3.37	17.09
210.5142	8.2	3.4	17.07
210.5714	8.19	3.4	17.06
210.6286	8.2	3.39	17.03
210.6858	8.23	3.35	17.03
210.7428	8.23	3.35	16.99
210.8	8.25	3.36	16.96
210.8572	8.21	3.38	16.92
210.9142	8.2	3.41	16.9
210.9714	8.19	3.43	16.86
211.0286	8.16	3.45	16.85
211.0858	8.12	3.47	16.83
211.1428	8.1	3.45	16.79
211.2	8.09	3.47	16.76
211.2572	8.03	3.5	16.74
211.3142	8.05	3.49	16.72
211.3714	8.04	3.48	16.7
211.4286	8.03	3.49	16.68
211.4858	8.03	3.45	16.65
211.5428	8.05	3.44	16.63
211.6	8.06	3.43	16.59
211.6572	8.12	3.4	16.56
211.7142	8.13	3.4	16.55
211.7714	8.14	3.4	16.56
211.8286	8.15	3.42	16.52
211.8858	8.14	3.43	16.52
211.9428	8.16	3.42	16.53
212	8.17	3.43	16.52

212.0572	8.17	3.44	16.51
212.1142	8.15	3.46	16.51
212.1714	8.14	3.47	16.5
212.2286	8.09	3.47	16.49
212.2858	8.08	3.48	16.47
212.3428	8.09	3.46	16.45
212.4	8.1	3.44	16.43
212.4572	8.11	3.44	16.39
212.5142	8.09	3.43	16.4
212.5714	8.07	3.41	16.41
212.6286	8.05	3.4	16.39
212.6858	8.06	3.37	16.37
212.7428	8.06	3.37	16.36
212.8	8.07	3.37	16.33
212.8572	8.07	3.37	16.3
212.9142	8.04	3.41	16.24
212.9714	8.04	3.39	16.23
213.0286	8.02	3.4	16.2
213.0858	8.01	3.42	16.16
213.1428	7.99	3.46	16.13
213.2	8.02	3.46	16.1
213.2572	8.02	3.46	16.08
213.3142	8.03	3.47	16.07
213.3714	8.02	3.48	16.05
213.4286	8.01	3.5	16.01
213.4858	8	3.5	15.97
213.5428	8	3.51	15.96
213.6	8.01	3.48	15.94
213.6572	8.03	3.47	15.91
213.7142	8.05	3.44	15.89
213.7714	8.06	3.41	15.85
213.8286	8.04	3.42	15.86
213.8858	8.03	3.39	15.84
213.9428	8.04	3.41	15.85
214	8.04	3.41	15.86
214.0572	8.05	3.42	15.85
214.1142	8.04	3.39	15.83
214.1714	8.03	3.42	15.81
214.2286	8.03	3.43	15.78
214.2858	8.01	3.45	15.75
214.3428	7.99	3.47	15.75
214.4	7.98	3.48	15.73
214.4572	7.96	3.51	15.7
214.5142	7.95	3.48	15.69
214.5714	7.98	3.49	15.67
214.6286	7.96	3.49	15.64
214.6858	7.96	3.49	15.62

214.7428	7.96	3.47	15.59
214.8	7.96	3.46	15.59
214.8572	7.94	3.47	15.55
214.9142	7.94	3.5	15.51
214.9714	7.95	3.5	15.48
215.0286	7.95	3.49	15.47
215.0858	7.95	3.53	15.45
215.1428	7.93	3.53	15.43
215.2	7.94	3.54	15.41
215.2572	7.95	3.55	15.4
215.3142	7.94	3.56	15.4
215.3714	7.91	3.58	15.37
215.4286	7.93	3.57	15.37
215.4858	7.92	3.57	15.38
215.5428	7.92	3.59	15.38
215.6	7.91	3.6	15.4
215.6572	7.92	3.59	15.38
215.7142	7.91	3.57	15.35
215.7714	7.93	3.52	15.35
215.8286	7.94	3.51	15.35
215.8858	7.94	3.49	15.34
215.9428	7.98	3.47	15.31
216	7.96	3.45	15.31
216.0572	7.98	3.42	15.3
216.1142	7.98	3.4	15.27
216.1714	8.01	3.36	15.25
216.2286	8.02	3.35	15.22
216.2858	8.02	3.34	15.21
216.3428	8.01	3.36	15.18
216.4	7.96	3.39	15.17
216.4572	7.97	3.37	15.14
216.5142	7.97	3.35	15.12
216.5714	7.95	3.37	15.11
216.6286	7.91	3.41	15.1
216.6858	7.9	3.4	15.07
216.7428	7.91	3.4	15.05
216.8	7.92	3.42	15.05
216.8572	7.9	3.4	15.01
216.9142	7.91	3.38	14.98
216.9714	7.91	3.37	14.95
217.0286	7.9	3.4	14.93
217.0858	7.86	3.41	14.93
217.1428	7.87	3.41	14.91
217.2	7.9	3.39	14.89
217.2572	7.92	3.38	14.86
217.3142	7.9	3.4	14.83
217.3714	7.9	3.36	14.8

217.4286	7.88	3.39	14.76
217.4858	7.85	3.4	14.74
217.5428	7.85	3.41	14.73
217.6	7.86	3.38	14.72
217.6572	7.85	3.39	14.71
217.7142	7.84	3.4	14.67
217.7714	7.83	3.42	14.68
217.8286	7.8	3.43	14.67
217.8858	7.82	3.43	14.65
217.9428	7.78	3.45	14.64
218	7.79	3.44	14.62
218.0572	7.79	3.45	14.6
218.1142	7.8	3.42	14.59
218.1714	7.8	3.42	14.58
218.2286	7.81	3.42	14.57
218.2858	7.83	3.4	14.58
218.3428	7.86	3.35	14.58
218.4	7.87	3.32	14.55
218.4572	7.85	3.32	14.53
218.5142	7.85	3.32	14.5
218.5714	7.83	3.34	14.48
218.6286	7.84	3.34	14.47
218.6858	7.86	3.35	14.45
218.7428	7.83	3.36	14.45
218.8	7.77	3.38	14.43
218.8572	7.76	3.39	14.43
218.9142	7.74	3.44	14.42
218.9714	7.71	3.49	14.43
219.0286	7.73	3.5	14.41
219.0858	7.74	3.52	14.42
219.1428	7.76	3.51	14.4
219.2	7.75	3.5	14.37
219.2572	7.74	3.54	14.35
219.3142	7.74	3.54	14.35
219.3714	7.78	3.54	14.32
219.4286	7.79	3.53	14.32
219.4858	7.75	3.55	14.31
219.5428	7.77	3.53	14.29
219.6	7.8	3.47	14.27
219.6572	7.78	3.46	14.23
219.7142	7.76	3.45	14.21
219.7714	7.78	3.45	14.2
219.8286	7.79	3.42	14.2
219.8858	7.82	3.41	14.16
219.9428	7.8	3.38	14.14
220	7.77	3.37	14.11
220.0572	7.81	3.32	14.07

220.1142	7.82	3.3	14.06
220.1714	7.8	3.31	14.03
220.2286	7.8	3.34	14.01
220.2858	7.81	3.37	13.99
220.3428	7.76	3.38	13.94
220.4	7.75	3.43	13.92
220.4572	7.72	3.43	13.91
220.5142	7.72	3.43	13.91
220.5714	7.72	3.43	13.9
220.6286	7.71	3.44	13.89
220.6858	7.72	3.43	13.87
220.7428	7.72	3.44	13.85
220.8	7.72	3.44	13.86
220.8572	7.71	3.43	13.85
220.9142	7.72	3.44	13.83
220.9714	7.69	3.44	13.8
221.0286	7.69	3.46	13.79
221.0858	7.69	3.47	13.77
221.1428	7.67	3.5	13.77
221.2	7.64	3.52	13.78
221.2572	7.62	3.57	13.79
221.3142	7.59	3.59	13.79
221.3714	7.62	3.56	13.78
221.4286	7.61	3.55	13.74
221.4858	7.61	3.54	13.73
221.5428	7.62	3.56	13.7
221.6	7.64	3.53	13.68
221.6572	7.65	3.51	13.66
221.7142	7.65	3.49	13.64
221.7714	7.65	3.49	13.62
221.8286	7.64	3.48	13.63
221.8858	7.65	3.48	13.63
221.9428	7.6	3.5	13.63
222	7.58	3.51	13.63
222.0572	7.61	3.48	13.65
222.1142	7.6	3.47	13.62
222.1714	7.56	3.48	13.59
222.2286	7.54	3.52	13.56
222.2858	7.52	3.54	13.55
222.3428	7.54	3.52	13.56
222.4	7.55	3.52	13.56
222.4572	7.56	3.49	13.57
222.5142	7.57	3.49	13.56
222.5714	7.58	3.47	13.53
222.6286	7.54	3.5	13.51
222.6858	7.54	3.47	13.49
222.7428	7.56	3.46	13.48

222.8	7.59	3.43	13.45
222.8572	7.61	3.4	13.44
222.9142	7.61	3.41	13.41
222.9714	7.62	3.41	13.35
223.0286	7.62	3.41	13.32
223.0858	7.62	3.4	13.31
223.1428	7.62	3.42	13.3
223.2	7.64	3.41	13.29
223.2572	7.63	3.44	13.23
223.3142	7.62	3.45	13.23
223.3714	7.6	3.49	13.21
223.4286	7.57	3.52	13.16
223.4858	7.57	3.5	13.16
223.5428	7.56	3.51	13.15
223.6	7.55	3.51	13.13
223.6572	7.56	3.5	13.12
223.7142	7.54	3.52	13.12
223.7714	7.51	3.54	13.1
223.8286	7.51	3.53	13.09
223.8858	7.53	3.51	13.1
223.9428	7.52	3.5	13.09
224	7.54	3.47	13.09
224.0572	7.54	3.48	13.08
224.1142	7.53	3.51	13.06
224.1714	7.49	3.54	13.07
224.2286	7.47	3.55	13.05
224.2858	7.48	3.55	13.03
224.3428	7.46	3.55	13.04
224.4	7.51	3.54	13.04
224.4572	7.46	3.56	13.01
224.5142	7.47	3.55	12.99
224.5714	7.49	3.56	12.99
224.6286	7.47	3.59	12.98
224.6858	7.47	3.55	12.98
224.7428	7.48	3.51	12.97
224.8	7.49	3.52	12.96
224.8572	7.48	3.51	12.97
224.9142	7.52	3.49	12.96
224.9714	7.52	3.47	12.94
225.0286	7.52	3.48	12.93
225.0858	7.51	3.47	12.94
225.1428	7.5	3.44	12.94
225.2	7.52	3.42	12.93
225.2572	7.53	3.42	12.92
225.3142	7.52	3.45	12.91
225.3714	7.52	3.45	12.88
225.4286	7.53	3.46	12.88

225.4858	7.47	3.46	12.84
225.5428	7.46	3.49	12.83
225.6	7.44	3.5	12.8
225.6572	7.45	3.5	12.79
225.7142	7.44	3.52	12.8
225.7714	7.41	3.52	12.79
225.8286	7.4	3.52	12.77
225.8858	7.4	3.5	12.76
225.9428	7.42	3.5	12.75
226	7.41	3.52	12.75
226.0572	7.45	3.52	12.75
226.1142	7.45	3.51	12.73
226.1714	7.46	3.51	12.73
226.2286	7.47	3.48	12.72
226.2858	7.47	3.47	12.73
226.3428	7.48	3.49	12.72
226.4	7.48	3.49	12.73
226.4572	7.47	3.52	12.73
226.5142	7.42	3.5	12.7
226.5714	7.42	3.48	12.7
226.6286	7.39	3.45	12.67
226.6858	7.36	3.46	12.64
226.7428	7.36	3.45	12.62
226.8	7.34	3.49	12.61
226.8572	7.32	3.52	12.61
226.9142	7.33	3.49	12.59
226.9714	7.33	3.47	12.56
227.0286	7.33	3.43	12.54
227.0858	7.33	3.44	12.51
227.1428	7.33	3.45	12.5
227.2	7.33	3.48	12.48
227.2572	7.33	3.47	12.48
227.3142	7.32	3.47	12.49
227.3714	7.31	3.44	12.46
227.4286	7.33	3.42	12.46
227.4858	7.35	3.43	12.42
227.5428	7.34	3.44	12.4
227.6	7.33	3.47	12.36
227.6572	7.35	3.5	12.39
227.7142	7.36	3.48	12.37
227.7714	7.34	3.47	12.35
227.8286	7.34	3.48	12.33
227.8858	7.36	3.47	12.35
227.9428	7.37	3.47	12.34
228	7.34	3.51	12.33
228.0572	7.3	3.54	12.32
228.1142	7.28	3.56	12.3

228.1714	7.28	3.56	12.3
228.2286	7.28	3.55	12.27
228.2858	7.26	3.55	12.22
228.3428	7.24	3.56	12.21
228.4	7.23	3.58	12.19
228.4572	7.26	3.57	12.19
228.5142	7.27	3.55	12.18
228.5714	7.27	3.54	12.17
228.6286	7.26	3.54	12.14
228.6858	7.28	3.52	12.1
228.7428	7.3	3.52	12.06
228.8	7.31	3.52	12.06
228.8572	7.32	3.53	12.03
228.9142	7.34	3.53	12.01
228.9714	7.33	3.52	12.01
229.0286	7.33	3.51	12
229.0858	7.31	3.51	12
229.1428	7.29	3.53	12.01
229.2	7.29	3.5	12
229.2572	7.31	3.48	11.99
229.3142	7.29	3.48	11.99
229.3714	7.27	3.46	11.99
229.4286	7.28	3.43	11.99
229.4858	7.24	3.45	11.99
229.5428	7.25	3.45	11.99
229.6	7.24	3.46	12
229.6572	7.27	3.45	12.05
229.7142	7.28	3.44	12.04
229.7714	7.28	3.46	12.02
229.8286	7.23	3.47	12
229.8858	7.24	3.49	11.96
229.9428	7.26	3.49	11.93
230	7.27	3.5	11.91
230.0572	7.28	3.48	11.91
230.1142	7.29	3.47	11.91
230.1714	7.28	3.48	11.89
230.2286	7.28	3.48	11.87
230.2858	7.27	3.47	11.86
230.3428	7.26	3.46	11.85
230.4	7.27	3.47	11.84
230.4572	7.25	3.44	11.84
230.5142	7.24	3.44	11.83
230.5714	7.19	3.46	11.79
230.6286	7.18	3.47	11.77
230.6858	7.18	3.48	11.75
230.7428	7.17	3.47	11.75
230.8	7.14	3.48	11.75

230.8572	7.14	3.49	11.76
230.9142	7.16	3.5	11.73
230.9714	7.14	3.53	11.73
231.0286	7.16	3.55	11.72
231.0858	7.14	3.55	11.71
231.1428	7.15	3.55	11.71
231.2	7.14	3.53	11.69
231.2572	7.16	3.53	11.66
231.3142	7.12	3.53	11.66
231.3714	7.11	3.52	11.64
231.4286	7.1	3.53	11.62
231.4858	7.08	3.51	11.6
231.5428	7.1	3.49	11.58
231.6	7.09	3.46	11.55
231.6572	7.08	3.49	11.53
231.7142	7.08	3.49	11.53
231.7714	7.07	3.51	11.51
231.8286	7.08	3.49	11.52
231.8858	7.09	3.52	11.5
231.9428	7.08	3.54	11.5
232	7.07	3.56	11.5
232.0572	7.09	3.59	11.48
232.1142	7.07	3.61	11.49
232.1714	7.08	3.61	11.5
232.2286	7.1	3.57	11.49
232.2858	7.12	3.58	11.47
232.3428	7.14	3.54	11.47
232.4	7.11	3.59	11.51
232.4572	7.08	3.57	11.52
232.5142	7.08	3.56	11.54
232.5714	7.09	3.56	11.53
232.6286	7.12	3.5	11.52
232.6858	7.13	3.5	11.5
232.7428	7.14	3.49	11.52
232.8	7.11	3.52	11.54
232.8572	7.06	3.52	11.53
232.9142	7.04	3.55	11.51
232.9714	7.04	3.53	11.52
233.0286	7.07	3.51	11.48
233.0858	7.1	3.5	11.46
233.1428	7.1	3.47	11.48
233.2	7.06	3.51	11.46
233.2572	7.05	3.47	11.45
233.3142	7.05	3.48	11.41
233.3714	7.04	3.47	11.38
233.4286	7.07	3.45	11.36
233.4858	7.08	3.43	11.37

233.5428	7.07	3.42	11.36
233.6	7.05	3.44	11.36
233.6572	7.04	3.45	11.32
233.7142	7.06	3.45	11.28
233.7714	7.09	3.43	11.25
233.8286	7.09	3.46	11.26
233.8858	7.09	3.45	11.24
233.9428	7.1	3.44	11.23
234	7.11	3.45	11.22
234.0572	7.12	3.47	11.2
234.1142	7.11	3.48	11.2
234.1714	7.13	3.46	11.2
234.2286	7.12	3.48	11.19
234.2858	7.08	3.5	11.2
234.3428	7.06	3.55	11.15
234.4	7.04	3.56	11.13
234.4572	7.02	3.57	11.11
234.5142	6.98	3.6	11.11
234.5714	6.98	3.6	11.12
234.6286	7	3.57	11.11
234.6858	7	3.57	11.11
234.7428	6.99	3.58	11.09
234.8	6.98	3.56	11.1
234.8572	7	3.52	11.08
234.9142	7	3.48	11.07
234.9714	6.98	3.47	11.05
235.0286	6.98	3.45	11.04
235.0858	7	3.45	11.03
235.1428	7	3.44	11.04
235.2	6.98	3.43	11.05
235.2572	6.97	3.44	11.08
235.3142	6.96	3.46	11.09
235.3714	6.99	3.46	11.11
235.4286	6.96	3.48	11.08
235.4858	6.97	3.46	11.05
235.5428	6.94	3.48	11.05
235.6	6.94	3.51	11.03
235.6572	6.95	3.48	11.02
235.7142	6.95	3.49	11.03
235.7714	6.95	3.51	11.04
235.8286	6.95	3.51	11.04
235.8858	6.96	3.52	11.03
235.9428	6.94	3.5	11.03
236	6.94	3.51	11.02
236.0572	6.93	3.51	11
236.1142	6.97	3.48	10.98
236.1714	7	3.44	10.96

236.2286	7.01	3.43	10.94
236.2858	7.02	3.41	10.92
236.3428	6.97	3.42	10.93
236.4	6.97	3.42	10.94
236.4572	6.97	3.41	10.9
236.5142	6.98	3.43	10.88
236.5714	6.98	3.43	10.86
236.6286	6.99	3.46	10.83
236.6858	6.96	3.47	10.81
236.7428	6.9	3.5	10.79
236.8	6.88	3.52	10.8
236.8572	6.84	3.52	10.77
236.9142	6.84	3.51	10.77
236.9714	6.84	3.52	10.75
237.0286	6.84	3.5	10.75
237.0858	6.8	3.5	10.72
237.1428	6.8	3.52	10.74
237.2	6.77	3.51	10.75
237.2572	6.72	3.54	10.72
237.3142	6.75	3.53	10.72
237.3714	6.77	3.51	10.73
237.4286	6.77	3.51	10.73
237.4858	6.78	3.52	10.72
237.5428	6.78	3.51	10.7
237.6	6.75	3.53	10.7
237.6572	6.75	3.54	10.71
237.7142	6.76	3.54	10.69
237.7714	6.77	3.52	10.69
237.8286	6.82	3.49	10.7
237.8858	6.82	3.49	10.68
237.9428	6.79	3.52	10.68
238	6.79	3.54	10.67
238.0572	6.79	3.54	10.66
238.1142	6.78	3.56	10.63
238.1714	6.8	3.57	10.64
238.2286	6.8	3.54	10.62
238.2858	6.8	3.5	10.61
238.3428	6.78	3.53	10.6
238.4	6.77	3.54	10.59
238.4572	6.75	3.52	10.58
238.5142	6.73	3.54	10.56
238.5714	6.77	3.52	10.54
238.6286	6.77	3.51	10.51
238.6858	6.77	3.46	10.5
238.7428	6.78	3.42	10.47
238.8	6.78	3.46	10.47
238.8572	6.75	3.47	10.44

238.9142	6.76	3.44	10.44
238.9714	6.75	3.43	10.43
239.0286	6.74	3.46	10.43
239.0858	6.78	3.41	10.42
239.1428	6.78	3.4	10.42
239.2	6.76	3.42	10.41
239.2572	6.73	3.44	10.4
239.3142	6.72	3.46	10.41
239.3714	6.71	3.44	10.42
239.4286	6.73	3.44	10.42
239.4858	6.73	3.46	10.41
239.5428	6.74	3.47	10.39
239.6	6.74	3.47	10.36
239.6572	6.73	3.48	10.37
239.7142	6.72	3.48	10.35
239.7714	6.73	3.47	10.35
239.8286	6.73	3.45	10.34
239.8858	6.71	3.46	10.34
239.9428	6.71	3.47	10.34
240	6.69	3.51	10.32
240.0572	6.69	3.51	10.32
240.1142	6.67	3.53	10.32
240.1714	6.67	3.54	10.33
240.2286	6.65	3.57	10.32
240.2858	6.64	3.61	10.32
240.3428	6.63	3.63	10.3
240.4	6.65	3.61	10.3
240.4572	6.66	3.61	10.3
240.5142	6.67	3.58	10.3
240.5714	6.68	3.54	10.28
240.6286	6.7	3.54	10.28
240.6858	6.7	3.52	10.26
240.7428	6.72	3.5	10.25
240.8	6.75	3.47	10.23
240.8572	6.74	3.45	10.21
240.9142	6.74	3.43	10.2
240.9714	6.73	3.44	10.18
241.0286	6.74	3.44	10.17
241.0858	6.7	3.48	10.13
241.1428	6.72	3.48	10.11
241.2	6.72	3.47	10.1
241.2572	6.69	3.49	10.13
241.3142	6.67	3.47	10.14
241.3714	6.65	3.46	10.13
241.4286	6.65	3.45	10.12
241.4858	6.62	3.46	10.13
241.5428	6.6	3.47	10.12

241.6	6.6	3.45	10.12
241.6572	6.64	3.43	10.12
241.7142	6.62	3.42	10.1
241.7714	6.6	3.43	10.1
241.8286	6.6	3.42	10.08
241.8858	6.6	3.46	10.08
241.9428	6.59	3.48	10.07
242	6.58	3.49	10.07
242.0572	6.58	3.47	10.09
242.1142	6.62	3.46	10.09
242.1714	6.62	3.45	10.05
242.2286	6.6	3.46	10.04
242.2858	6.59	3.44	10.03
242.3428	6.59	3.4	10.02
242.4	6.59	3.41	10.01
242.4572	6.58	3.4	10.01
242.5142	6.57	3.41	10.02
242.5714	6.59	3.4	10
242.6286	6.58	3.41	10.02
242.6858	6.56	3.43	10
242.7428	6.55	3.45	10
242.8	6.53	3.47	10
242.8572	6.53	3.49	9.99
242.9142	6.53	3.51	9.98
242.9714	6.54	3.51	9.98
243.0286	6.53	3.5	9.96
243.0858	6.53	3.48	9.95
243.1428	6.51	3.52	9.94
243.2	6.51	3.49	9.94
243.2572	6.52	3.48	9.95
243.3142	6.52	3.47	9.92
243.3714	6.52	3.49	9.9
243.4286	6.53	3.47	9.89
243.4858	6.53	3.51	9.87
243.5428	6.52	3.5	9.83
243.6	6.53	3.5	9.83
243.6572	6.56	3.49	9.83
243.7142	6.6	3.46	9.83
243.7714	6.6	3.48	9.81
243.8286	6.59	3.48	9.8
243.8858	6.58	3.49	9.76
243.9428	6.59	3.46	9.76
244	6.6	3.5	9.76
244.0572	6.62	3.44	9.72
244.1142	6.6	3.45	9.74
244.1714	6.6	3.43	9.72
244.2286	6.56	3.44	9.73

244.2858	6.53	3.46	9.72
244.3428	6.52	3.46	9.7
244.4	6.49	3.45	9.71
244.4572	6.48	3.43	9.73
244.5142	6.46	3.42	9.73
244.5714	6.45	3.38	9.72
244.6286	6.44	3.37	9.71
244.6858	6.44	3.36	9.73
244.7428	6.44	3.37	9.74
244.8	6.45	3.35	9.72
244.8572	6.47	3.35	9.72
244.9142	6.46	3.36	9.71
244.9714	6.48	3.35	9.72
245.0286	6.49	3.37	9.69
245.0858	6.5	3.37	9.68
245.1428	6.5	3.41	9.67
245.2	6.5	3.41	9.67
245.2572	6.5	3.44	9.67
245.3142	6.5	3.43	9.67
245.3714	6.49	3.46	9.67
245.4286	6.47	3.46	9.67
245.4858	6.46	3.45	9.66
245.5428	6.46	3.46	9.64
245.6	6.45	3.48	9.63
245.6572	6.44	3.49	9.63
245.7142	6.44	3.48	9.66
245.7714	6.43	3.48	9.63
245.8286	6.43	3.47	9.64
245.8858	6.45	3.45	9.63
245.9428	6.43	3.44	9.64
246	6.43	3.45	9.66
246.0572	6.45	3.43	9.66
246.1142	6.44	3.44	9.64
246.1714	6.44	3.42	9.62
246.2286	6.44	3.39	9.61
246.2858	6.44	3.42	9.59
246.3428	6.44	3.41	9.56
246.4	6.43	3.41	9.58
246.4572	6.42	3.46	9.58
246.5142	6.39	3.47	9.56
246.5714	6.39	3.45	9.53
246.6286	6.36	3.5	9.49
246.6858	6.36	3.48	9.47
246.7428	6.36	3.48	9.46
246.8	6.37	3.49	9.47
246.8572	6.38	3.46	9.45
246.9142	6.38	3.45	9.41

246.9714	6.37	3.45	9.4
247.0286	6.33	3.46	9.41
247.0858	6.35	3.44	9.41
247.1428	6.37	3.44	9.43
247.2	6.39	3.43	9.42
247.2572	6.41	3.42	9.42
247.3142	6.41	3.44	9.38
247.3714	6.4	3.41	9.4
247.4286	6.42	3.42	9.42
247.4858	6.38	3.45	9.43
247.5428	6.39	3.45	9.44
247.6	6.42	3.42	9.45
247.6572	6.43	3.41	9.46
247.7142	6.4	3.44	9.43
247.7714	6.41	3.43	9.41
247.8286	6.4	3.42	9.43
247.8858	6.36	3.43	9.42
247.9428	6.34	3.47	9.42
248	6.31	3.47	9.42
248.0572	6.35	3.45	9.39
248.1142	6.36	3.47	9.4
248.1714	6.34	3.45	9.42
248.2286	6.31	3.48	9.41
248.2858	6.32	3.46	9.37
248.3428	6.3	3.44	9.34
248.4	6.31	3.46	9.34
248.4572	6.31	3.47	9.34
248.5142	6.31	3.46	9.32
248.5714	6.34	3.46	9.32
248.6286	6.3	3.45	9.33
248.6858	6.29	3.46	9.33
248.7428	6.29	3.48	9.31
248.8	6.32	3.45	9.29
248.8572	6.31	3.45	9.28
248.9142	6.32	3.45	9.28
248.9714	6.29	3.45	9.28
249.0286	6.31	3.43	9.27
249.0858	6.33	3.4	9.26
249.1428	6.29	3.39	9.25
249.2	6.27	3.41	9.25
249.2572	6.21	3.41	9.24
249.3142	6.16	3.44	9.23
249.3714	6.14	3.45	9.22
249.4286	6.16	3.44	9.22
249.4858	6.16	3.43	9.2
249.5428	6.18	3.46	9.16
249.6	6.15	3.47	9.16

249.6572	6.14	3.49	9.13
249.7142	6.13	3.51	9.13
249.7714	6.17	3.5	9.13
249.8286	6.21	3.49	9.14
249.8858	6.22	3.49	9.14
249.9428	6.23	3.5	9.14
250	6.21	3.53	9.13
250.0572	6.2	3.54	9.13
250.1142	6.22	3.5	9.12
250.1714	6.24	3.49	9.12
250.2286	6.22	3.51	9.13
250.2858	6.22	3.52	9.12
250.3428	6.2	3.52	9.12
250.4	6.2	3.54	9.09
250.4572	6.21	3.5	9.11
250.5142	6.25	3.47	9.12
250.5714	6.23	3.44	9.12
250.6286	6.24	3.41	9.1
250.6858	6.18	3.44	9.09
250.7428	6.16	3.45	9.08
250.8	6.18	3.42	9.08
250.8572	6.17	3.4	9.07
250.9142	6.18	3.4	9.05
250.9714	6.18	3.39	9.04
251.0286	6.19	3.37	9.04
251.0858	6.17	3.37	9.04
251.1428	6.2	3.39	9.04
251.2	6.19	3.4	9.05
251.2572	6.2	3.4	9.04
251.3142	6.19	3.4	9.05
251.3714	6.15	3.4	9.05
251.4286	6.19	3.4	9.02
251.4858	6.2	3.39	9.03
251.5428	6.19	3.37	9.06
251.6	6.14	3.41	9.04
251.6572	6.14	3.43	9.03
251.7142	6.11	3.44	9.01
251.7714	6.12	3.45	9
251.8286	6.12	3.43	9.02
251.8858	6.13	3.4	9.01
251.9428	6.12	3.4	8.98
252	6.11	3.41	8.96
252.0572	6.11	3.41	8.95
252.1142	6.12	3.42	8.94
252.1714	6.13	3.39	8.92
252.2286	6.12	3.36	8.9
252.2858	6.12	3.35	8.88

252.3428	6.11	3.34	8.87
252.4	6.08	3.37	8.86
252.4572	6.06	3.41	8.85
252.5142	6.07	3.4	8.86
252.5714	6.06	3.4	8.85
252.6286	6.06	3.41	8.86
252.6858	6.03	3.4	8.85
252.7428	6.04	3.4	8.85
252.8	6.04	3.41	8.86
252.8572	6.05	3.43	8.86
252.9142	6.03	3.44	8.84
252.9714	6.03	3.42	8.82
253.0286	6.03	3.4	8.83
253.0858	6.04	3.39	8.83
253.1428	6.05	3.39	8.85
253.2	6.05	3.37	8.86
253.2572	6.03	3.4	8.87
253.3142	6.02	3.4	8.87
253.3714	6.01	3.39	8.86
253.4286	6.03	3.38	8.85
253.4858	6.05	3.35	8.86
253.5428	6.06	3.34	8.86
253.6	6.07	3.36	8.83
253.6572	6.05	3.36	8.82
253.7142	6.03	3.39	8.8
253.7714	6.04	3.38	8.8
253.8286	6.05	3.37	8.79
253.8858	6.05	3.4	8.78
253.9428	6.04	3.41	8.76
254	6.02	3.4	8.76
254.0572	6	3.41	8.75
254.1142	6.01	3.43	8.75
254.1714	6.02	3.41	8.73
254.2286	6.01	3.44	8.71
254.2858	6.05	3.42	8.71
254.3428	6.05	3.44	8.69
254.4	6.04	3.42	8.69
254.4572	6.05	3.39	8.7
254.5142	6.03	3.38	8.71
254.5714	6.05	3.4	8.7
254.6286	6.04	3.38	8.7
254.6858	6.04	3.38	8.7
254.7428	6.04	3.36	8.71
254.8	6.05	3.32	8.72
254.8572	6.03	3.31	8.69
254.9142	6.02	3.31	8.68
254.9714	6.02	3.32	8.67

255.0286	6.02	3.33	8.66
255.0858	6.05	3.34	8.65
255.1428	6.05	3.37	8.63
255.2	6.06	3.38	8.62
255.2572	6.04	3.38	8.62
255.3142	6.01	3.4	8.58
255.3714	6.01	3.41	8.57
255.4286	6.02	3.43	8.55
255.4858	5.99	3.44	8.57
255.5428	5.98	3.44	8.56
255.6	5.96	3.44	8.54
255.6572	5.94	3.43	8.52
255.7142	5.94	3.39	8.51
255.7714	5.95	3.37	8.53
255.8286	5.96	3.4	8.5
255.8858	5.95	3.42	8.49
255.9428	5.95	3.43	8.5
256	5.94	3.44	8.51
256.0572	5.96	3.42	8.52
256.1142	5.97	3.44	8.51
256.1714	5.97	3.46	8.49
256.2286	5.98	3.45	8.5
256.2858	5.97	3.46	8.48
256.3428	5.94	3.47	8.47
256.4	5.93	3.45	8.44
256.4572	5.93	3.44	8.44
256.5142	5.93	3.44	8.44
256.5714	5.93	3.43	8.45
256.6286	5.92	3.43	8.45
256.6858	5.93	3.41	8.45
256.7428	5.93	3.41	8.48
256.8	5.9	3.45	8.47
256.8572	5.89	3.45	8.47
256.9142	5.9	3.44	8.46
256.9714	5.91	3.45	8.46
257.0286	5.9	3.44	8.47
257.0858	5.9	3.42	8.47
257.1428	5.87	3.43	8.46
257.2	5.89	3.41	8.45
257.2572	5.9	3.38	8.44
257.3142	5.9	3.39	8.42
257.3714	5.92	3.33	8.41
257.4286	5.93	3.35	8.4
257.4858	5.9	3.38	8.39
257.5428	5.9	3.38	8.37
257.6	5.91	3.38	8.38
257.6572	5.9	3.36	8.36

257.7142	5.92	3.35	8.35
257.7714	5.91	3.36	8.34
257.8286	5.9	3.37	8.33
257.8858	5.89	3.37	8.3
257.9428	5.86	3.4	8.3
258	5.82	3.43	8.32
258.0572	5.84	3.41	8.32
258.1142	5.83	3.4	8.31
258.1714	5.81	3.41	8.31
258.2286	5.8	3.41	8.33
258.2858	5.82	3.38	8.34
258.3428	5.81	3.39	8.35
258.4	5.81	3.4	8.34
258.4572	5.8	3.38	8.33
258.5142	5.82	3.38	8.31
258.5714	5.85	3.34	8.31
258.6286	5.84	3.33	8.32
258.6858	5.85	3.36	8.32
258.7428	5.85	3.37	8.31
258.8	5.85	3.38	8.33
258.8572	5.82	3.39	8.3
258.9142	5.8	3.41	8.28
258.9714	5.79	3.42	8.28
259.0286	5.79	3.42	8.3
259.0858	5.77	3.45	8.29
259.1428	5.76	3.45	8.28
259.2	5.77	3.47	8.25
259.2572	5.76	3.47	8.24
259.3142	5.79	3.44	8.25
259.3714	5.82	3.43	8.26
259.4286	5.83	3.45	8.25
259.4858	5.84	3.45	8.24
259.5428	5.86	3.44	8.22
259.6	5.86	3.43	8.2
259.6572	5.87	3.41	8.19
259.7142	5.86	3.42	8.17
259.7714	5.85	3.42	8.16
259.8286	5.85	3.4	8.14
259.8858	5.83	3.41	8.13
259.9428	5.77	3.46	8.12
260	5.75	3.45	8.12
260.0572	5.74	3.44	8.12
260.1142	5.73	3.41	8.14
260.1714	5.69	3.42	8.13
260.2286	5.67	3.43	8.12
260.2858	5.68	3.4	8.1
260.3428	5.7	3.39	8.1

260.4	5.71	3.37	8.07
260.4572	5.72	3.36	8.07
260.5142	5.75	3.28	8.07
260.5714	5.76	3.27	8.07
260.6286	5.75	3.27	8.07
260.6858	5.74	3.3	8.08
260.7428	5.75	3.3	8.07
260.8	5.75	3.3	8.06
260.8572	5.75	3.3	8.06
260.9142	5.72	3.31	8.07
260.9714	5.69	3.31	8.04
261.0286	5.69	3.32	8.01
261.0858	5.67	3.41	8
261.1428	5.64	3.44	8.01
261.2	5.63	3.44	8.01
261.2572	5.63	3.45	8.01
261.3142	5.65	3.45	8.04
261.3714	5.68	3.44	8.05
261.4286	5.66	3.47	8.06
261.4858	5.66	3.48	8.05
261.5428	5.69	3.47	8.05
261.6	5.67	3.48	8.04
261.6572	5.67	3.45	8.05
261.7142	5.69	3.42	8.07
261.7714	5.71	3.4	8.06
261.8286	5.72	3.4	8.04
261.8858	5.72	3.39	8.03
261.9428	5.7	3.4	8.06
262	5.7	3.37	8.06
262.0572	5.7	3.37	8.04
262.1142	5.74	3.35	8.01
262.1714	5.74	3.34	8.01
262.2286	5.75	3.34	8
262.2858	5.72	3.34	7.97
262.3428	5.74	3.35	7.95
262.4	5.72	3.34	7.97
262.4572	5.71	3.35	7.96
262.5142	5.72	3.33	7.95
262.5714	5.73	3.33	7.95
262.6286	5.74	3.33	7.95
262.6858	5.74	3.3	7.92
262.7428	5.73	3.33	7.92
262.8	5.73	3.32	7.93
262.8572	5.7	3.33	7.94
262.9142	5.7	3.34	7.91
262.9714	5.7	3.33	7.9
263.0286	5.65	3.35	7.9

263.0858	5.66	3.36	7.92
263.1428	5.64	3.36	7.92
263.2	5.63	3.35	7.91
263.2572	5.63	3.36	7.93
263.3142	5.6	3.36	7.93
263.3714	5.56	3.4	7.92
263.4286	5.55	3.35	7.91
263.4858	5.58	3.33	7.92
263.5428	5.57	3.32	7.9
263.6	5.57	3.31	7.89
263.6572	5.61	3.3	7.87
263.7142	5.62	3.31	7.84
263.7714	5.61	3.32	7.83
263.8286	5.6	3.35	7.83
263.8858	5.62	3.35	7.83
263.9428	5.62	3.31	7.81
264	5.63	3.35	7.81
264.0572	5.61	3.36	7.79
264.1142	5.6	3.39	7.78
264.1714	5.62	3.4	7.75
264.2286	5.6	3.39	7.75
264.2858	5.58	3.36	7.72
264.3428	5.59	3.36	7.74
264.4	5.59	3.34	7.72
264.4572	5.6	3.33	7.72
264.5142	5.6	3.35	7.74
264.5714	5.61	3.37	7.73
264.6286	5.63	3.35	7.73
264.6858	5.66	3.32	7.73
264.7428	5.66	3.3	7.73
264.8	5.66	3.29	7.73
264.8572	5.67	3.3	7.74
264.9142	5.69	3.29	7.73
264.9714	5.67	3.3	7.74
265.0286	5.66	3.28	7.73
265.0858	5.65	3.27	7.73
265.1428	5.64	3.25	7.72
265.2	5.61	3.27	7.75
265.2572	5.57	3.27	7.74
265.3142	5.55	3.3	7.75
265.3714	5.57	3.31	7.74
265.4286	5.57	3.31	7.72
265.4858	5.54	3.32	7.71
265.5428	5.53	3.31	7.71
265.6	5.53	3.34	7.69
265.6572	5.49	3.35	7.67
265.7142	5.51	3.35	7.67

265.7714	5.53	3.35	7.67
265.8286	5.53	3.36	7.65
265.8858	5.5	3.37	7.65
265.9428	5.48	3.37	7.65
266	5.48	3.38	7.66
266.0572	5.5	3.39	7.64
266.1142	5.5	3.42	7.65
266.1714	5.46	3.41	7.63
266.2286	5.53	3.38	7.61
266.2858	5.5	3.38	7.62
266.3428	5.5	3.37	7.61
266.4	5.51	3.37	7.61
266.4572	5.55	3.34	7.61
266.5142	5.57	3.34	7.61
266.5714	5.56	3.34	7.62
266.6286	5.52	3.34	7.63
266.6858	5.52	3.31	7.63
266.7428	5.54	3.29	7.64
266.8	5.51	3.27	7.63
266.8572	5.51	3.26	7.65
266.9142	5.47	3.28	7.67
266.9714	5.47	3.28	7.66
267.0286	5.43	3.28	7.65
267.0858	5.39	3.3	7.66
267.1428	5.4	3.3	7.67
267.2	5.41	3.3	7.66
267.2572	5.43	3.3	7.67
267.3142	5.45	3.29	7.69
267.3714	5.48	3.3	7.7
267.4286	5.46	3.3	7.72
267.4858	5.5	3.29	7.74
267.5428	5.52	3.28	7.7
267.6	5.55	3.27	7.68
267.6572	5.55	3.28	7.68
267.7142	5.56	3.28	7.68
267.7714	5.58	3.24	7.64
267.8286	5.56	3.24	7.6
267.8858	5.54	3.25	7.6
267.9428	5.53	3.26	7.6
268	5.52	3.26	7.6
268.0572	5.52	3.27	7.59
268.1142	5.52	3.28	7.59
268.1714	5.5	3.3	7.58
268.2286	5.51	3.28	7.56
268.2858	5.5	3.28	7.55
268.3428	5.49	3.28	7.54
268.4	5.47	3.27	7.54

268.4572	5.47	3.28	7.56
268.5142	5.48	3.27	7.58
268.5714	5.46	3.28	7.57
268.6286	5.45	3.29	7.57
268.6858	5.46	3.28	7.58
268.7428	5.46	3.26	7.57
268.8	5.47	3.26	7.55
268.8572	5.47	3.24	7.51
268.9142	5.48	3.25	7.5
268.9714	5.46	3.26	7.46
269.0286	5.47	3.23	7.46
269.0858	5.49	3.21	7.45
269.1428	5.49	3.19	7.45
269.2	5.5	3.17	7.44
269.2572	5.47	3.17	7.43
269.3142	5.44	3.18	7.42
269.3714	5.45	3.19	7.43
269.4286	5.46	3.22	7.41
269.4858	5.46	3.21	7.41
269.5428	5.47	3.23	7.39
269.6	5.48	3.24	7.37
269.6572	5.44	3.26	7.39
269.7142	5.45	3.25	7.4
269.7714	5.44	3.27	7.4
269.8286	5.46	3.26	7.39
269.8858	5.44	3.26	7.41
269.9428	5.42	3.25	7.4
270	5.4	3.25	7.39
270.0572	5.39	3.25	7.39
270.1142	5.38	3.25	7.38
270.1714	5.36	3.26	7.37
270.2286	5.35	3.27	7.36
270.2858	5.33	3.29	7.33
270.3428	5.34	3.25	7.33
270.4	5.33	3.28	7.32
270.4572	5.33	3.27	7.33
270.5142	5.33	3.26	7.33
270.5714	5.3	3.27	7.32
270.6286	5.3	3.25	7.32
270.6858	5.29	3.25	7.31
270.7428	5.33	3.26	7.33
270.8	5.33	3.25	7.34
270.8572	5.32	3.27	7.34
270.9142	5.31	3.29	7.34
270.9714	5.29	3.28	7.33
271.0286	5.32	3.29	7.34
271.0858	5.34	3.27	7.33

271.1428	5.32	3.29	7.32
271.2	5.31	3.3	7.31
271.2572	5.34	3.3	7.31
271.3142	5.33	3.28	7.28
271.3714	5.34	3.27	7.26
271.4286	5.34	3.26	7.27
271.4858	5.33	3.27	7.26
271.5428	5.34	3.29	7.26
271.6	5.35	3.28	7.27
271.6572	5.33	3.31	7.27
271.7142	5.37	3.26	7.24
271.7714	5.4	3.25	7.24
271.8286	5.38	3.23	7.24
271.8858	5.37	3.26	7.24
271.9428	5.35	3.25	7.22
272	5.35	3.24	7.23
272.0572	5.38	3.22	7.23
272.1142	5.38	3.21	7.22
272.1714	5.36	3.2	7.23
272.2286	5.38	3.18	7.26
272.2858	5.39	3.19	7.26
272.3428	5.35	3.21	7.23
272.4	5.34	3.24	7.25
272.4572	5.34	3.23	7.23
272.5142	5.33	3.23	7.2
272.5714	5.33	3.24	7.19
272.6286	5.29	3.26	7.18
272.6858	5.26	3.28	7.19
272.7428	5.27	3.26	7.19
272.8	5.22	3.3	7.2
272.8572	5.21	3.32	7.2
272.9142	5.23	3.28	7.19
272.9714	5.22	3.26	7.18
273.0286	5.22	3.27	7.18
273.0858	5.21	3.27	7.17
273.1428	5.24	3.25	7.16
273.2	5.25	3.23	7.15
273.2572	5.27	3.23	7.16
273.3142	5.23	3.27	7.13
273.3714	5.27	3.25	7.12
273.4286	5.23	3.24	7.16
273.4858	5.22	3.26	7.16
273.5428	5.21	3.25	7.16
273.6	5.22	3.26	7.15
273.6572	5.23	3.26	7.12
273.7142	5.21	3.27	7.09
273.7714	5.22	3.27	7.08

273.8286	5.23	3.27	7.09
273.8858	5.25	3.26	7.08
273.9428	5.22	3.26	7.08
274	5.26	3.27	7.06
274.0572	5.28	3.26	7.05
274.1142	5.28	3.3	7.06
274.1714	5.26	3.29	7.06
274.2286	5.25	3.29	7.07
274.2858	5.22	3.3	7.08
274.3428	5.23	3.29	7.06
274.4	5.24	3.29	7.06
274.4572	5.25	3.27	7.06
274.5142	5.29	3.25	7.05
274.5714	5.26	3.22	7.06
274.6286	5.25	3.22	7.06
274.6858	5.26	3.21	7.06
274.7428	5.28	3.21	7.04
274.8	5.3	3.22	7.06
274.8572	5.33	3.19	7.06
274.9142	5.31	3.2	7.06
274.9714	5.28	3.2	7.08
275.0286	5.28	3.2	7.05
275.0858	5.24	3.2	7.05
275.1428	5.23	3.25	7.05
275.2	5.21	3.25	7.05
275.2572	5.19	3.25	7.05
275.3142	5.15	3.26	7.05
275.3714	5.16	3.25	7.06
275.4286	5.14	3.25	7.06
275.4858	5.16	3.25	7.07
275.5428	5.15	3.23	7.07
275.6	5.14	3.23	7.06
275.6572	5.14	3.23	7.07
275.7142	5.15	3.23	7.05
275.7714	5.13	3.23	7.05
275.8286	5.13	3.22	7.03
275.8858	5.13	3.2	7.01
275.9428	5.12	3.19	7.02
276	5.13	3.2	7
276.0572	5.11	3.23	6.97
276.1142	5.1	3.27	6.95
276.1714	5.08	3.28	6.91
276.2286	5.06	3.27	6.89
276.2858	5.06	3.24	6.88
276.3428	5.07	3.25	6.87
276.4	5.05	3.24	6.86
276.4572	5.08	3.27	6.84

276.5142	5.07	3.28	6.82
276.5714	5.05	3.28	6.83
276.6286	5.04	3.26	6.82
276.6858	5.05	3.26	6.82
276.7428	5.06	3.26	6.84
276.8	5.09	3.24	6.84
276.8572	5.11	3.26	6.82
276.9142	5.13	3.25	6.84
276.9714	5.18	3.23	6.85
277.0286	5.19	3.21	6.87
277.0858	5.19	3.2	6.87
277.1428	5.22	3.2	6.85
277.2	5.21	3.22	6.84
277.2572	5.2	3.19	6.85
277.3142	5.23	3.17	6.85
277.3714	5.25	3.18	6.84
277.4286	5.22	3.17	6.86
277.4858	5.21	3.16	6.83
277.5428	5.19	3.18	6.84
277.6	5.18	3.22	6.84
277.6572	5.17	3.21	6.84
277.7142	5.18	3.2	6.85
277.7714	5.15	3.19	6.85
277.8286	5.15	3.22	6.84
277.8858	5.11	3.23	6.84
277.9428	5.07	3.24	6.84
278	5.05	3.25	6.87
278.0572	5.04	3.27	6.89
278.1142	5.02	3.29	6.91
278.1714	5	3.25	6.9
278.2286	4.97	3.26	6.89
278.2858	4.95	3.27	6.91
278.3428	4.97	3.28	6.91
278.4	4.96	3.27	6.94
278.4572	4.97	3.27	6.94
278.5142	4.97	3.27	6.92
278.5714	4.99	3.29	6.92
278.6286	4.99	3.31	6.91
278.6858	5.03	3.29	6.92
278.7428	5.04	3.27	6.92
278.8	5.07	3.25	6.93
278.8572	5.07	3.27	6.91
278.9142	5.06	3.25	6.88
278.9714	5.07	3.25	6.87
279.0286	5.06	3.24	6.85
279.0858	5.07	3.23	6.83
279.1428	5.08	3.17	6.84

279.2	5.1	3.15	6.82
279.2572	5.08	3.16	6.81
279.3142	5.06	3.18	6.79
279.3714	5.05	3.2	6.78
279.4286	5.05	3.21	6.78
279.4858	5.05	3.23	6.77
279.5428	5.04	3.24	6.76
279.6	5.04	3.27	6.76
279.6572	5.02	3.26	6.74
279.7142	5.01	3.26	6.7
279.7714	4.99	3.26	6.71
279.8286	5.01	3.24	6.7
279.8858	5.03	3.23	6.69
279.9428	5.04	3.21	6.69
280	5.02	3.19	6.69
280.0572	5.01	3.16	6.68
280.1142	5.02	3.17	6.67
280.1714	5.02	3.15	6.65
280.2286	5.04	3.16	6.61
280.2858	5.05	3.18	6.59
280.3428	5.04	3.22	6.61
280.4	5.04	3.23	6.61
280.4572	5.02	3.24	6.6
280.5142	5.01	3.24	6.59
280.5714	5.01	3.25	6.59
280.6286	4.99	3.29	6.59
280.6858	4.99	3.28	6.59
280.7428	4.97	3.28	6.6
280.8	4.94	3.28	6.62
280.8572	4.93	3.28	6.63
280.9142	4.89	3.27	6.64
280.9714	4.87	3.28	6.65
281.0286	4.86	3.28	6.65
281.0858	4.84	3.3	6.67
281.1428	4.85	3.29	6.7
281.2	4.86	3.27	6.72
281.2572	4.87	3.27	6.71
281.3142	4.88	3.25	6.7
281.3714	4.87	3.25	6.68
281.4286	4.86	3.25	6.67
281.4858	4.86	3.27	6.67
281.5428	4.85	3.26	6.68
281.6	4.85	3.26	6.66
281.6572	4.86	3.28	6.65
281.7142	4.86	3.28	6.64
281.7714	4.86	3.27	6.64
281.8286	4.82	3.26	6.64

281.8858	4.81	3.26	6.61
281.9428	4.84	3.24	6.61
282	4.89	3.21	6.6
282.0572	4.9	3.19	6.61
282.1142	4.91	3.19	6.58
282.1714	4.91	3.19	6.55
282.2286	4.91	3.16	6.55
282.2858	4.92	3.14	6.56
282.3428	4.92	3.15	6.54
282.4	4.94	3.15	6.54
282.4572	4.92	3.18	6.53
282.5142	4.88	3.2	6.54
282.5714	4.86	3.21	6.54
282.6286	4.88	3.2	6.53
282.6858	4.88	3.19	6.52
282.7428	4.88	3.18	6.51
282.8	4.91	3.14	6.51
282.8572	4.91	3.14	6.52
282.9142	4.91	3.12	6.52
282.9714	4.94	3.12	6.5
283.0286	4.96	3.1	6.51
283.0858	4.97	3.1	6.51
283.1428	4.99	3.09	6.5
283.2	5	3.07	6.51
283.2572	5	3.07	6.5
283.3142	4.98	3.09	6.49
283.3714	4.96	3.12	6.47
283.4286	4.98	3.13	6.46
283.4858	4.96	3.14	6.47
283.5428	4.93	3.13	6.46
283.6	4.93	3.12	6.45
283.6572	4.93	3.1	6.43
283.7142	4.91	3.12	6.43
283.7714	4.88	3.12	6.41
283.8286	4.88	3.15	6.42
283.8858	4.91	3.12	6.41
283.9428	4.91	3.1	6.41
284	4.87	3.11	6.42
284.0572	4.84	3.13	6.42
284.1142	4.83	3.15	6.42
284.1714	4.81	3.18	6.45
284.2286	4.79	3.2	6.45
284.2858	4.78	3.18	6.47
284.3428	4.78	3.18	6.48
284.4	4.77	3.18	6.49
284.4572	4.77	3.16	6.5
284.5142	4.76	3.17	6.5

284.5714	4.76	3.15	6.51
284.6286	4.79	3.15	6.52
284.6858	4.81	3.16	6.51
284.7428	4.79	3.14	6.48
284.8	4.8	3.12	6.48
284.8572	4.82	3.12	6.48
284.9142	4.83	3.15	6.47
284.9714	4.84	3.13	6.47
285.0286	4.81	3.16	6.46
285.0858	4.81	3.18	6.46
285.1428	4.8	3.22	6.44
285.2	4.77	3.22	6.43
285.2572	4.78	3.18	6.43
285.3142	4.78	3.19	6.42
285.3714	4.76	3.23	6.41
285.4286	4.76	3.21	6.42
285.4858	4.77	3.2	6.41
285.5428	4.76	3.21	6.41
285.6	4.76	3.22	6.4
285.6572	4.73	3.21	6.39
285.7142	4.74	3.19	6.41
285.7714	4.72	3.21	6.39
285.8286	4.71	3.23	6.39
285.8858	4.72	3.23	6.39
285.9428	4.75	3.21	6.4
286	4.73	3.22	6.39
286.0572	4.72	3.22	6.38
286.1142	4.74	3.24	6.37
286.1714	4.72	3.22	6.36
286.2286	4.76	3.19	6.34
286.2858	4.77	3.22	6.33
286.3428	4.78	3.21	6.33
286.4	4.77	3.21	6.33
286.4572	4.75	3.21	6.32
286.5142	4.76	3.22	6.32
286.5714	4.77	3.2	6.35
286.6286	4.78	3.22	6.33
286.6858	4.77	3.21	6.35
286.7428	4.77	3.24	6.34
286.8	4.77	3.25	6.33
286.8572	4.78	3.22	6.32
286.9142	4.79	3.2	6.31
286.9714	4.81	3.21	6.3
287.0286	4.82	3.18	6.31
287.0858	4.81	3.14	6.3
287.1428	4.81	3.12	6.31
287.2	4.83	3.07	6.31

287.2572	4.83	3.04	6.28
287.3142	4.84	3.03	6.28
287.3714	4.84	3.03	6.25
287.4286	4.85	3.02	6.23
287.4858	4.85	3.01	6.23
287.5428	4.83	2.99	6.25
287.6	4.83	3	6.24
287.6572	4.81	3.04	6.24
287.7142	4.79	3.08	6.23
287.7714	4.74	3.12	6.23
287.8286	4.73	3.13	6.21
287.8858	4.72	3.12	6.23
287.9428	4.74	3.12	6.2
288	4.72	3.12	6.19
288.0572	4.69	3.14	6.17
288.1142	4.68	3.15	6.17
288.1714	4.66	3.14	6.19
288.2286	4.67	3.17	6.2
288.2858	4.68	3.18	6.23
288.3428	4.67	3.19	6.25
288.4	4.66	3.2	6.23
288.4572	4.66	3.19	6.22
288.5142	4.65	3.17	6.22
288.5714	4.65	3.17	6.21
288.6286	4.65	3.14	6.2
288.6858	4.68	3.12	6.19
288.7428	4.7	3.12	6.21
288.8	4.73	3.06	6.22
288.8572	4.77	3.03	6.24
288.9142	4.82	3	6.25
288.9714	4.81	3.02	6.27
289.0286	4.79	3.04	6.27
289.0858	4.79	3.05	6.25
289.1428	4.78	3.06	6.25
289.2	4.77	3.09	6.23
289.2572	4.78	3.08	6.22
289.3142	4.76	3.09	6.23
289.3714	4.73	3.09	6.22
289.4286	4.7	3.11	6.21
289.4858	4.68	3.1	6.22
289.5428	4.7	3.06	6.23
289.6	4.71	3.08	6.24
289.6572	4.69	3.09	6.24
289.7142	4.7	3.09	6.22
289.7714	4.72	3.06	6.21
289.8286	4.7	3.1	6.21
289.8858	4.71	3.09	6.19

289.9428	4.74	3.11	6.18
290	4.71	3.09	6.16
290.0572	4.69	3.1	6.16
290.1142	4.69	3.12	6.14
290.1714	4.7	3.1	6.13
290.2286	4.69	3.07	6.11
290.2858	4.7	3.07	6.11
290.3428	4.68	3.09	6.08
290.4	4.69	3.08	6.07
290.4572	4.69	3.07	6.05
290.5142	4.69	3.07	6.04
290.5714	4.72	3.06	6.03
290.6286	4.73	3.06	6.04
290.6858	4.71	3.08	6.05
290.7428	4.67	3.1	6.06
290.8	4.68	3.12	6.07
290.8572	4.66	3.12	6.09
290.9142	4.64	3.12	6.11
290.9714	4.61	3.13	6.1
291.0286	4.61	3.15	6.11
291.0858	4.61	3.13	6.11
291.1428	4.58	3.16	6.1
291.2	4.57	3.17	6.11
291.2572	4.57	3.17	6.12
291.3142	4.61	3.15	6.11
291.3714	4.6	3.14	6.12
291.4286	4.6	3.15	6.11
291.4858	4.61	3.15	6.12
291.5428	4.65	3.15	6.1
291.6	4.67	3.12	6.09
291.6572	4.68	3.15	6.07
291.7142	4.67	3.16	6.06
291.7714	4.67	3.12	6.06
291.8286	4.69	3.11	6.04
291.8858	4.66	3.09	6.02
291.9428	4.72	3.09	6.04
292	4.75	3.1	6.04
292.0572	4.74	3.12	6.04
292.1142	4.72	3.1	6.05
292.1714	4.7	3.13	6.05
292.2286	4.68	3.11	6.06
292.2858	4.67	3.1	6.06
292.3428	4.66	3.13	6.06
292.4	4.66	3.13	6.04
292.4572	4.66	3.12	6.06
292.5142	4.63	3.13	6.07
292.5714	4.58	3.11	6.07

292.6286	4.57	3.07	6.06
292.6858	4.57	3.06	6.05
292.7428	4.6	3.05	6.07
292.8	4.6	3.06	6.09
292.8572	4.62	3.04	6.09
292.9142	4.65	3.06	6.08
292.9714	4.64	3.07	6.09
293.0286	4.62	3.08	6.08
293.0858	4.62	3.08	6.06
293.1428	4.61	3.11	6.05
293.2	4.61	3.14	6.02
293.2572	4.6	3.17	6.02
293.3142	4.58	3.16	6.02
293.3714	4.56	3.15	6.01
293.4286	4.55	3.14	6
293.4858	4.53	3.13	6
293.5428	4.51	3.13	5.97
293.6	4.53	3.13	5.94
293.6572	4.53	3.13	5.91
293.7142	4.53	3.13	5.92
293.7714	4.53	3.11	5.91
293.8286	4.55	3.09	5.94
293.8858	4.55	3.08	5.94
293.9428	4.57	3.06	5.94
294	4.59	3.09	5.94
294.0572	4.57	3.1	5.95
294.1142	4.59	3.08	5.95
294.1714	4.6	3.03	5.94
294.2286	4.63	3.03	5.93
294.2858	4.62	3.04	5.94
294.3428	4.62	3.05	5.93
294.4	4.59	3.05	5.92
294.4572	4.57	3.08	5.94
294.5142	4.57	3.09	5.96
294.5714	4.51	3.12	5.95
294.6286	4.49	3.1	5.94
294.6858	4.5	3.09	5.91
294.7428	4.53	3.08	5.88
294.8	4.52	3.06	5.85
294.8572	4.54	3.02	5.83
294.9142	4.54	3	5.82
294.9714	4.56	2.99	5.81
295.0286	4.56	2.99	5.83
295.0858	4.57	2.98	5.84
295.1428	4.58	2.97	5.85
295.2	4.6	2.98	5.83
295.2572	4.6	2.99	5.83

295.3142	4.55	3.06	5.82
295.3714	4.52	3.09	5.82
295.4286	4.48	3.13	5.82
295.4858	4.47	3.14	5.84
295.5428	4.46	3.13	5.84
295.6	4.47	3.14	5.82
295.6572	4.48	3.16	5.84
295.7142	4.51	3.12	5.85
295.7714	4.51	3.11	5.87
295.8286	4.48	3.12	5.87
295.8858	4.48	3.09	5.87
295.9428	4.47	3.08	5.85
296	4.49	3.09	5.84
296.0572	4.49	3.07	5.84
296.1142	4.47	3.09	5.83
296.1714	4.45	3.07	5.83
296.2286	4.45	3.06	5.85
296.2858	4.42	3.09	5.86
296.3428	4.41	3.08	5.85
296.4	4.41	3.07	5.84
296.4572	4.39	3.1	5.84
296.5142	4.41	3.07	5.85
296.5714	4.42	3.06	5.83
296.6286	4.43	3.06	5.83
296.6858	4.42	3.07	5.81
296.7428	4.43	3.08	5.8
296.8	4.43	3.06	5.8
296.8572	4.43	3.06	5.84
296.9142	4.41	3.1	5.84
296.9714	4.42	3.11	5.82
297.0286	4.42	3.11	5.82
297.0858	4.43	3.08	5.82
297.1428	4.44	3.09	5.82
297.2	4.42	3.07	5.82
297.2572	4.4	3.07	5.83
297.3142	4.39	3.06	5.83
297.3714	4.39	3.08	5.83
297.4286	4.37	3.08	5.82
297.4858	4.37	3.08	5.84
297.5428	4.38	3.05	5.83
297.6	4.4	3.03	5.83
297.6572	4.39	3.02	5.84
297.7142	4.37	3.07	5.85
297.7714	4.38	3.09	5.84
297.8286	4.37	3.12	5.82
297.8858	4.38	3.11	5.85
297.9428	4.4	3.1	5.87

298	4.41	3.11	5.85
298.0572	4.44	3.1	5.85
298.1142	4.43	3.1	5.85
298.1714	4.41	3.1	5.83
298.2286	4.42	3.11	5.83
298.2858	4.44	3.08	5.84
298.3428	4.43	3.07	5.82
298.4	4.48	3.04	5.81
298.4572	4.49	3.02	5.81
298.5142	4.47	3.04	5.8
298.5714	4.47	2.99	5.78
298.6286	4.46	3.01	5.79
298.6858	4.44	3.05	5.78
298.7428	4.42	3.05	5.75
298.8	4.41	3.06	5.7
298.8572	4.39	3.04	5.69
298.9142	4.41	3.04	5.72
298.9714	4.37	3.05	5.71
299.0286	4.36	3.09	5.68
299.0858	4.37	3.07	5.66
299.1428	4.36	3.1	5.66
299.2	4.36	3.1	5.65
299.2572	4.38	3.08	5.65
299.3142	4.39	3.09	5.64
299.3714	4.41	3.09	5.64
299.4286	4.4	3.13	5.65
299.4858	4.38	3.13	5.65
299.5428	4.36	3.12	5.65
299.6	4.35	3.09	5.66
299.6572	4.34	3.1	5.68
299.7142	4.33	3.09	5.7
299.7714	4.33	3.08	5.7
299.8286	4.32	3.08	5.7
299.8858	4.34	3.07	5.72
299.9428	4.32	3.06	5.74
300	4.34	3.04	5.74
300.0572	4.38	3.01	5.7
300.1142	4.41	3	5.7
300.1714	4.4	2.99	5.7
300.2286	4.39	3	5.68
300.2858	4.41	2.99	5.67
300.3428	4.41	2.98	5.67
300.4	4.4	2.99	5.66
300.4572	4.39	2.99	5.66
300.5142	4.4	2.99	5.63
300.5714	4.38	2.99	5.62
300.6286	4.35	2.98	5.6

300.6858	4.33	2.98	5.58
300.7428	4.34	2.98	5.56
300.8	4.4	2.97	5.55
300.8572	4.38	2.95	5.52
300.9142	4.38	2.98	5.51
300.9714	4.38	2.98	5.55
301.0286	4.39	2.98	5.55
301.0858	4.34	3.04	5.56
301.1428	4.35	3.05	5.59
301.2	4.38	3.08	5.59
301.2572	4.41	3.09	5.61
301.3142	4.4	3.1	5.61
301.3714	4.37	3.12	5.6
301.4286	4.37	3.12	5.61
301.4858	4.36	3.13	5.61
301.5428	4.36	3.15	5.62
301.6	4.36	3.14	5.64
301.6572	4.39	3.06	5.63
301.7142	4.39	3.04	5.64
301.7714	4.37	3.05	5.63
301.8286	4.34	3.05	5.62
301.8858	4.31	3.06	5.6
301.9428	4.32	3.05	5.58
302	4.32	3.06	5.59
302.0572	4.34	3.03	5.59
302.1142	4.36	2.99	5.57
302.1714	4.34	3	5.54
302.2286	4.34	3.03	5.53
302.2858	4.35	3	5.53
302.3428	4.37	3	5.54
302.4	4.37	3	5.53
302.4572	4.39	2.97	5.52
302.5142	4.38	3	5.53
302.5714	4.35	3	5.53
302.6286	4.36	2.99	5.53
302.6858	4.34	2.99	5.53
302.7428	4.34	2.98	5.55
302.8	4.33	2.98	5.56
302.8572	4.32	2.99	5.58
302.9142	4.29	3.01	5.58
302.9714	4.31	3.01	5.57
303.0286	4.28	3.02	5.58
303.0858	4.27	2.99	5.59
303.1428	4.25	2.99	5.58
303.2	4.24	3	5.57
303.2572	4.23	3	5.59
303.3142	4.21	3.02	5.59

303.3714	4.25	2.98	5.58
303.4286	4.23	2.99	5.57
303.4858	4.23	2.98	5.57
303.5428	4.18	2.99	5.57
303.6	4.2	2.98	5.58
303.6572	4.22	2.96	5.58
303.7142	4.28	2.93	5.56
303.7714	4.29	2.92	5.56
303.8286	4.29	2.91	5.55
303.8858	4.3	2.86	5.54
303.9428	4.32	2.89	5.54
304	4.31	2.89	5.55
304.0572	4.33	2.86	5.56
304.1142	4.35	2.83	5.59
304.1714	4.38	2.84	5.59
304.2286	4.35	2.87	5.59
304.2858	4.34	2.91	5.61
304.3428	4.32	2.94	5.6
304.4	4.31	2.96	5.58
304.4572	4.29	3	5.57
304.5142	4.29	2.98	5.55
304.5714	4.31	2.99	5.54
304.6286	4.33	2.97	5.56
304.6858	4.34	2.99	5.57
304.7428	4.33	2.97	5.56
304.8	4.32	2.97	5.56
304.8572	4.32	2.94	5.55
304.9142	4.32	2.9	5.56
304.9714	4.33	2.89	5.53
305.0286	4.34	2.88	5.48
305.0858	4.32	2.87	5.47
305.1428	4.3	2.84	5.47
305.2	4.26	2.85	5.43
305.2572	4.27	2.82	5.43
305.3142	4.25	2.85	5.45
305.3714	4.27	2.84	5.46
305.4286	4.24	2.85	5.47
305.4858	4.23	2.87	5.47
305.5428	4.23	2.86	5.45
305.6	4.23	2.87	5.44
305.6572	4.22	2.92	5.46
305.7142	4.2	2.95	5.46
305.7714	4.2	2.96	5.46
305.8286	4.16	3	5.45
305.8858	4.15	3.01	5.45
305.9428	4.12	3.05	5.45
306	4.15	3.06	5.43

306.0572	4.15	3.08	5.44
306.1142	4.14	3.11	5.43
306.1714	4.12	3.1	5.41
306.2286	4.1	3.09	5.4
306.2858	4.13	3.08	5.4
306.3428	4.11	3.07	5.39
306.4	4.11	3.06	5.37
306.4572	4.11	3.04	5.36
306.5142	4.13	3	5.34
306.5714	4.14	2.94	5.35
306.6286	4.16	2.91	5.34
306.6858	4.16	2.91	5.33
306.7428	4.16	2.93	5.32
306.8	4.2	2.89	5.32
306.8572	4.22	2.91	5.33
306.9142	4.22	2.91	5.34
306.9714	4.25	2.91	5.33
307.0286	4.21	2.94	5.34
307.0858	4.21	2.96	5.36
307.1428	4.21	2.98	5.36
307.2	4.19	2.96	5.38
307.2572	4.23	2.95	5.38
307.3142	4.26	2.94	5.39
307.3714	4.22	2.95	5.4
307.4286	4.18	2.94	5.41
307.4858	4.2	2.94	5.4
307.5428	4.2	2.94	5.4
307.6	4.23	2.92	5.39
307.6572	4.24	2.9	5.38
307.7142	4.21	2.91	5.4
307.7714	4.19	2.92	5.41
307.8286	4.21	2.89	5.42
307.8858	4.15	2.89	5.42
307.9428	4.19	2.87	5.41
308	4.21	2.86	5.41
308.0572	4.21	2.88	5.4
308.1142	4.2	2.87	5.38
308.1714	4.21	2.85	5.37
308.2286	4.2	2.86	5.38
308.2858	4.2	2.87	5.37
308.3428	4.21	2.87	5.36
308.4	4.13	2.94	5.37
308.4572	4.15	2.95	5.37
308.5142	4.1	2.98	5.38
308.5714	4.1	2.98	5.37
308.6286	4.09	2.94	5.36
308.6858	4.1	2.95	5.35

308.7428	4.08	2.95	5.35
308.8	4.08	2.98	5.37
308.8572	4.07	2.94	5.37
308.9142	4.07	2.94	5.36
308.9714	4.12	2.9	5.38
309.0286	4.14	2.9	5.37
309.0858	4.15	2.89	5.36
309.1428	4.17	2.88	5.35
309.2	4.18	2.9	5.36
309.2572	4.2	2.87	5.34
309.3142	4.2	2.89	5.31
309.3714	4.21	2.86	5.31
309.4286	4.19	2.88	5.31
309.4858	4.17	2.91	5.32
309.5428	4.14	2.96	5.32
309.6	4.13	2.94	5.32
309.6572	4.14	2.94	5.31
309.7142	4.13	2.95	5.31
309.7714	4.12	2.95	5.31
309.8286	4.1	2.98	5.28
309.8858	4.11	2.96	5.28
309.9428	4.08	2.98	5.28
310	4.09	2.98	5.29
310.0572	4.12	2.93	5.3
310.1142	4.13	2.92	5.31
310.1714	4.13	2.91	5.32
310.2286	4.11	2.9	5.32
310.2858	4.1	2.92	5.34
310.3428	4.09	2.93	5.32
310.4	4.08	2.93	5.31
310.4572	4.08	2.93	5.29
310.5142	4.09	2.94	5.29
310.5714	4.1	2.93	5.27
310.6286	4.08	2.95	5.25
310.6858	4.08	2.94	5.25
310.7428	4.09	2.95	5.27
310.8	4.06	2.94	5.25
310.8572	4.06	2.93	5.25
310.9142	4.05	2.92	5.24
310.9714	4.05	2.93	5.22
311.0286	4.06	2.91	5.21
311.0858	4.08	2.91	5.23
311.1428	4.1	2.89	5.24
311.2	4.11	2.9	5.24
311.2572	4.1	2.88	5.25
311.3142	4.11	2.87	5.25
311.3714	4.13	2.85	5.23

311.4286	4.12	2.87	5.21
311.4858	4.12	2.89	5.21
311.5428	4.13	2.89	5.22
311.6	4.16	2.9	5.21
311.6572	4.11	2.89	5.2
311.7142	4.09	2.9	5.19
311.7714	4.11	2.89	5.2
311.8286	4.1	2.93	5.21
311.8858	4.07	2.94	5.19
311.9428	4.06	2.99	5.18
312	4.08	2.97	5.16
312.0572	4.08	2.95	5.16
312.1142	4.09	2.92	5.15
312.1714	4.06	2.92	5.15
312.2286	4.05	2.95	5.14
312.2858	4.03	2.95	5.16
312.3428	4.01	2.98	5.17
312.4	3.97	2.96	5.19
312.4572	3.98	2.96	5.2
312.5142	3.96	2.94	5.23
312.5714	3.96	2.93	5.23
312.6286	3.94	2.94	5.22
312.6858	3.96	2.95	5.2
312.7428	3.95	2.97	5.21
312.8	3.96	2.97	5.22
312.8572	3.99	2.97	5.24
312.9142	4.01	2.92	5.27
312.9714	4.04	2.93	5.26
313.0286	4.05	2.91	5.28
313.0858	4.06	2.92	5.26
313.1428	4.06	2.92	5.28
313.2	4.04	2.93	5.26
313.2572	3.98	2.96	5.25
313.3142	3.99	2.93	5.24
313.3714	4	2.94	5.21
313.4286	4	2.95	5.19
313.4858	3.96	2.98	5.18
313.5428	3.94	2.97	5.18
313.6	3.93	2.98	5.17
313.6572	3.94	2.99	5.16
313.7142	3.94	2.97	5.16
313.7714	3.97	2.94	5.14
313.8286	4	2.9	5.1
313.8858	3.97	2.9	5.1
313.9428	4	2.87	5.08
314	3.97	2.86	5.09
314.0572	3.97	2.88	5.08

314.1142	3.99	2.87	5.1
314.1714	4	2.87	5.09
314.2286	3.99	2.87	5.08
314.2858	3.98	2.9	5.06
314.3428	3.97	2.93	5.05
314.4	3.95	2.96	5.04
314.4572	3.94	2.98	5.05
314.5142	3.91	2.97	5.03
314.5714	3.92	2.95	5.03
314.6286	3.9	2.94	5.02
314.6858	3.91	2.95	5.01
314.7428	3.91	2.97	5.02
314.8	3.91	2.96	5.02
314.8572	3.92	2.96	5.01
314.9142	3.92	2.94	4.97
314.9714	3.92	2.92	4.96
315.0286	3.94	2.91	4.95
315.0858	3.94	2.93	4.95
315.1428	3.96	2.95	4.96
315.2	3.96	2.93	4.99
315.2572	3.97	2.94	4.98
315.3142	3.97	2.88	4.97
315.3714	3.97	2.9	4.98
315.4286	3.95	2.87	4.99
315.4858	3.96	2.88	5
315.5428	3.96	2.87	5.01
315.6	3.99	2.86	5.02
315.6572	4	2.83	5.01
315.7142	4.02	2.8	4.99
315.7714	4.01	2.81	5
315.8286	4.03	2.82	5.05
315.8858	4	2.84	5.06
315.9428	3.97	2.83	5.08
316	3.97	2.85	5.09
316.0572	3.98	2.83	5.1
316.1142	3.97	2.82	5.1
316.1714	3.94	2.84	5.12
316.2286	3.93	2.87	5.12
316.2858	3.91	2.89	5.1
316.3428	3.92	2.9	5.11
316.4	3.9	2.87	5.09
316.4572	3.89	2.89	5.09
316.5142	3.91	2.89	5.08
316.5714	3.93	2.9	5.06
316.6286	3.9	2.94	5.07
316.6858	3.91	2.95	5.07
316.7428	3.94	2.92	5.06

316.8	3.97	2.92	5.03
316.8572	3.99	2.89	5
316.9142	4	2.89	4.98
316.9714	3.98	2.93	4.95
317.0286	4.02	2.88	4.94
317.0858	4.01	2.88	4.94
317.1428	3.99	2.88	4.93
317.2	4.02	2.83	4.94
317.2572	4	2.85	4.95
317.3142	3.99	2.85	4.94
317.3714	3.95	2.85	4.92
317.4286	3.92	2.86	4.93
317.4858	3.92	2.84	4.95
317.5428	3.93	2.84	4.91
317.6	3.92	2.84	4.89
317.6572	3.93	2.84	4.9
317.7142	3.93	2.84	4.92
317.7714	3.92	2.86	4.93
317.8286	3.91	2.86	4.93
317.8858	3.9	2.87	4.95
317.9428	3.88	2.89	4.93
318	3.87	2.89	4.94
318.0572	3.83	2.91	4.97
318.1142	3.82	2.89	4.94
318.1714	3.81	2.89	4.93
318.2286	3.81	2.89	4.94
318.2858	3.81	2.89	4.95
318.3428	3.79	2.88	4.94
318.4	3.79	2.86	4.94
318.4572	3.78	2.86	4.97
318.5142	3.8	2.85	5
318.5714	3.79	2.85	4.99
318.6286	3.83	2.81	5
318.6858	3.83	2.8	5.01
318.7428	3.83	2.81	5.02
318.8	3.84	2.83	5.04
318.8572	3.84	2.81	5.05
318.9142	3.87	2.8	5.02
318.9714	3.89	2.8	5.01
319.0286	3.89	2.81	5.01
319.0858	3.9	2.79	5.03
319.1428	3.93	2.78	5.03
319.2	3.92	2.8	5.03
319.2572	3.9	2.83	5.03
319.3142	3.89	2.86	5.02
319.3714	3.87	2.83	5.01
319.4286	3.88	2.82	5

319.4858	3.87	2.81	4.98
319.5428	3.9	2.8	4.97
319.6	3.9	2.79	4.96
319.6572	3.88	2.8	4.94
319.7142	3.89	2.81	4.94
319.7714	3.88	2.8	4.94
319.8286	3.89	2.81	4.93
319.8858	3.9	2.78	4.93
319.9428	3.9	2.81	4.94
320	3.89	2.83	4.91
320.0572	3.89	2.86	4.9
320.1142	3.86	2.88	4.9
320.1714	3.85	2.89	4.91
320.2286	3.86	2.87	4.93
320.2858	3.83	2.87	4.92
320.3428	3.79	2.89	4.92
320.4	3.79	2.87	4.91
320.4572	3.79	2.88	4.9
320.5142	3.79	2.86	4.89
320.5714	3.81	2.84	4.89
320.6286	3.8	2.82	4.87
320.6858	3.78	2.83	4.87
320.7428	3.78	2.83	4.87
320.8	3.76	2.83	4.86
320.8572	3.77	2.87	4.86
320.9142	3.82	2.82	4.86
320.9714	3.82	2.85	4.87
321.0286	3.81	2.84	4.86
321.0858	3.82	2.82	4.87
321.1428	3.8	2.84	4.87
321.2	3.79	2.85	4.88
321.2572	3.82	2.85	4.88
321.3142	3.83	2.86	4.9
321.3714	3.84	2.86	4.89
321.4286	3.83	2.83	4.89
321.4858	3.82	2.86	4.89
321.5428	3.8	2.87	4.91
321.6	3.84	2.85	4.9
321.6572	3.82	2.87	4.89
321.7142	3.81	2.87	4.89
321.7714	3.81	2.82	4.89
321.8286	3.81	2.79	4.91
321.8858	3.82	2.77	4.92
321.9428	3.85	2.77	4.92
322	3.86	2.76	4.9
322.0572	3.84	2.77	4.88
322.1142	3.83	2.75	4.85

322.1714	3.83	2.77	4.84
322.2286	3.81	2.77	4.84
322.2858	3.83	2.77	4.85
322.3428	3.83	2.77	4.86
322.4	3.81	2.82	4.86
322.4572	3.81	2.84	4.85
322.5142	3.8	2.82	4.86
322.5714	3.8	2.82	4.87
322.6286	3.81	2.81	4.84
322.6858	3.83	2.81	4.83
322.7428	3.82	2.79	4.8
322.8	3.84	2.76	4.8
322.8572	3.85	2.75	4.77
322.9142	3.85	2.75	4.77
322.9714	3.88	2.71	4.77
323.0286	3.87	2.7	4.78
323.0858	3.87	2.7	4.77
323.1428	3.86	2.72	4.77
323.2	3.85	2.71	4.77
323.2572	3.85	2.72	4.76
323.3142	3.85	2.76	4.74
323.3714	3.82	2.75	4.73
323.4286	3.8	2.77	4.72
323.4858	3.78	2.79	4.71
323.5428	3.75	2.79	4.73
323.6	3.71	2.82	4.73
323.6572	3.7	2.85	4.73
323.7142	3.68	2.86	4.72
323.7714	3.68	2.88	4.74
323.8286	3.65	2.89	4.74
323.8858	3.67	2.86	4.74
323.9428	3.67	2.89	4.74
324	3.69	2.92	4.74
324.0572	3.69	2.9	4.73
324.1142	3.67	2.95	4.72
324.1714	3.69	2.93	4.72
324.2286	3.69	2.91	4.74
324.2858	3.68	2.89	4.74
324.3428	3.68	2.89	4.76
324.4	3.7	2.89	4.77
324.4572	3.69	2.9	4.76
324.5142	3.71	2.87	4.77
324.5714	3.74	2.84	4.77
324.6286	3.75	2.86	4.78
324.6858	3.77	2.82	4.77
324.7428	3.74	2.83	4.77
324.8	3.72	2.85	4.77

324.8572	3.75	2.83	4.79
324.9142	3.76	2.82	4.8
324.9714	3.77	2.79	4.8
325.0286	3.77	2.76	4.81
325.0858	3.77	2.76	4.8
325.1428	3.74	2.77	4.81
325.2	3.74	2.75	4.8
325.2572	3.75	2.74	4.77
325.3142	3.77	2.76	4.76
325.3714	3.78	2.73	4.76
325.4286	3.77	2.73	4.78
325.4858	3.75	2.74	4.78
325.5428	3.73	2.75	4.77
325.6	3.72	2.76	4.78
325.6572	3.7	2.79	4.78
325.7142	3.67	2.79	4.8
325.7714	3.67	2.8	4.78
325.8286	3.66	2.79	4.75
325.8858	3.67	2.77	4.74
325.9428	3.65	2.79	4.72
326	3.67	2.78	4.73
326.0572	3.69	2.78	4.72
326.1142	3.7	2.78	4.73
326.1714	3.7	2.81	4.75
326.2286	3.67	2.84	4.75
326.2858	3.66	2.84	4.75
326.3428	3.65	2.84	4.72
326.4	3.66	2.85	4.69
326.4572	3.65	2.88	4.68
326.5142	3.67	2.85	4.66
326.5714	3.66	2.87	4.64
326.6286	3.66	2.87	4.64
326.6858	3.67	2.88	4.64
326.7428	3.68	2.87	4.67
326.8	3.69	2.84	4.67
326.8572	3.74	2.8	4.68
326.9142	3.75	2.78	4.66
326.9714	3.74	2.79	4.65
327.0286	3.76	2.74	4.64
327.0858	3.76	2.75	4.61
327.1428	3.75	2.73	4.61
327.2	3.76	2.75	4.61
327.2572	3.77	2.71	4.64
327.3142	3.78	2.7	4.66
327.3714	3.79	2.69	4.67
327.4286	3.77	2.69	4.66
327.4858	3.77	2.7	4.67

327.5428	3.77	2.69	4.66
327.6	3.76	2.7	4.65
327.6572	3.73	2.7	4.61
327.7142	3.72	2.73	4.6
327.7714	3.72	2.71	4.6
327.8286	3.72	2.74	4.59
327.8858	3.73	2.71	4.59
327.9428	3.75	2.69	4.58
328	3.78	2.69	4.6
328.0572	3.77	2.74	4.62
328.1142	3.74	2.75	4.61
328.1714	3.72	2.78	4.59
328.2286	3.74	2.76	4.59
328.2858	3.72	2.76	4.58
328.3428	3.72	2.75	4.6
328.4	3.7	2.75	4.59
328.4572	3.67	2.75	4.59
328.5142	3.61	2.76	4.61
328.5714	3.56	2.79	4.63
328.6286	3.56	2.76	4.63
328.6858	3.57	2.73	4.64
328.7428	3.57	2.7	4.67
328.8	3.58	2.72	4.68
328.8572	3.61	2.71	4.69
328.9142	3.6	2.72	4.66
328.9714	3.6	2.69	4.64
329.0286	3.61	2.69	4.62
329.0858	3.65	2.7	4.63
329.1428	3.69	2.68	4.63
329.2	3.67	2.69	4.61
329.2572	3.69	2.73	4.61
329.3142	3.69	2.74	4.6
329.3714	3.68	2.73	4.61
329.4286	3.67	2.71	4.59
329.4858	3.67	2.71	4.57
329.5428	3.67	2.71	4.57
329.6	3.65	2.76	4.55
329.6572	3.63	2.78	4.53
329.7142	3.6	2.83	4.55
329.7714	3.62	2.81	4.58
329.8286	3.65	2.78	4.58
329.8858	3.67	2.77	4.58
329.9428	3.65	2.79	4.58
330	3.64	2.78	4.58
330.0572	3.64	2.78	4.59
330.1142	3.63	2.76	4.58
330.1714	3.64	2.75	4.57

330.2286	3.65	2.74	4.59
330.2858	3.67	2.69	4.59
330.3428	3.62	2.7	4.6
330.4	3.57	2.73	4.62
330.4572	3.57	2.74	4.6
330.5142	3.59	2.73	4.62
330.5714	3.57	2.75	4.63
330.6286	3.56	2.75	4.61
330.6858	3.55	2.76	4.6
330.7428	3.53	2.76	4.6
330.8	3.52	2.79	4.61
330.8572	3.54	2.79	4.61
330.9142	3.59	2.76	4.6
330.9714	3.6	2.75	4.6
331.0286	3.62	2.76	4.6
331.0858	3.61	2.74	4.62
331.1428	3.64	2.74	4.61
331.2	3.65	2.73	4.6
331.2572	3.65	2.72	4.59
331.3142	3.66	2.71	4.58
331.3714	3.68	2.68	4.59
331.4286	3.67	2.68	4.59
331.4858	3.64	2.68	4.58
331.5428	3.64	2.69	4.55
331.6	3.61	2.68	4.53
331.6572	3.61	2.66	4.52
331.7142	3.6	2.66	4.53
331.7714	3.6	2.66	4.52
331.8286	3.6	2.65	4.53
331.8858	3.6	2.68	4.53
331.9428	3.6	2.68	4.5
332	3.61	2.69	4.51
332.0572	3.62	2.71	4.49
332.1142	3.62	2.71	4.47
332.1714	3.63	2.72	4.47
332.2286	3.63	2.71	4.48
332.2858	3.62	2.7	4.52
332.3428	3.61	2.7	4.49
332.4	3.6	2.72	4.5
332.4572	3.56	2.72	4.52
332.5142	3.56	2.72	4.54
332.5714	3.52	2.72	4.53
332.6286	3.55	2.69	4.53
332.6858	3.55	2.68	4.53
332.7428	3.53	2.72	4.52
332.8	3.51	2.73	4.53
332.8572	3.51	2.75	4.55

332.9142	3.51	2.78	4.54
332.9714	3.54	2.77	4.54
333.0286	3.56	2.75	4.53
333.0858	3.56	2.75	4.54
333.1428	3.57	2.73	4.54
333.2	3.56	2.77	4.51
333.2572	3.52	2.79	4.54
333.3142	3.54	2.77	4.51
333.3714	3.53	2.77	4.49
333.4286	3.52	2.77	4.44
333.4858	3.51	2.73	4.45
333.5428	3.47	2.72	4.45
333.6	3.48	2.71	4.44
333.6572	3.47	2.71	4.42
333.7142	3.47	2.72	4.41
333.7714	3.45	2.72	4.39
333.8286	3.49	2.73	4.42
333.8858	3.49	2.72	4.44
333.9428	3.49	2.7	4.42
334	3.5	2.68	4.41
334.0572	3.51	2.68	4.4
334.1142	3.53	2.71	4.42
334.1714	3.54	2.72	4.39
334.2286	3.56	2.72	4.4
334.2858	3.59	2.72	4.43
334.3428	3.6	2.7	4.46
334.4	3.63	2.66	4.45
334.4572	3.6	2.65	4.45
334.5142	3.58	2.68	4.44
334.5714	3.59	2.67	4.44
334.6286	3.6	2.65	4.44
334.6858	3.56	2.66	4.45
334.7428	3.54	2.68	4.43
334.8	3.52	2.69	4.44
334.8572	3.5	2.69	4.45
334.9142	3.48	2.71	4.44
334.9714	3.44	2.74	4.43
335.0286	3.44	2.74	4.43
335.0858	3.47	2.7	4.45
335.1428	3.47	2.69	4.43
335.2	3.46	2.72	4.4
335.2572	3.49	2.7	4.37
335.3142	3.48	2.7	4.36
335.3714	3.5	2.68	4.36
335.4286	3.47	2.68	4.36
335.4858	3.49	2.66	4.35
335.5428	3.49	2.63	4.37

335.6	3.51	2.64	4.35
335.6572	3.53	2.65	4.34
335.7142	3.54	2.65	4.32
335.7714	3.54	2.65	4.32
335.8286	3.55	2.63	4.33
335.8858	3.57	2.61	4.33
335.9428	3.56	2.62	4.31
336	3.6	2.59	4.3
336.0572	3.63	2.58	4.31
336.1142	3.63	2.56	4.33
336.1714	3.65	2.55	4.34
336.2286	3.62	2.57	4.32
336.2858	3.6	2.59	4.32
336.3428	3.56	2.6	4.31
336.4	3.56	2.62	4.3
336.4572	3.59	2.62	4.29
336.5142	3.58	2.62	4.3
336.5714	3.56	2.65	4.31
336.6286	3.54	2.66	4.32
336.6858	3.53	2.68	4.32
336.7428	3.53	2.7	4.32
336.8	3.55	2.68	4.33
336.8572	3.55	2.68	4.34
336.9142	3.57	2.66	4.34
336.9714	3.57	2.67	4.34
337.0286	3.55	2.68	4.34
337.0858	3.53	2.69	4.37
337.1428	3.55	2.68	4.37
337.2	3.57	2.69	4.37
337.2572	3.56	2.67	4.39
337.3142	3.55	2.66	4.41
337.3714	3.5	2.68	4.41
337.4286	3.49	2.7	4.4
337.4858	3.49	2.71	4.38
337.5428	3.51	2.69	4.36
337.6	3.5	2.67	4.36
337.6572	3.51	2.68	4.39
337.7142	3.49	2.68	4.37
337.7714	3.48	2.67	4.37
337.8286	3.46	2.69	4.36
337.8858	3.45	2.69	4.35
337.9428	3.46	2.69	4.35
338	3.48	2.66	4.36
338.0572	3.5	2.66	4.38
338.1142	3.46	2.67	4.36
338.1714	3.46	2.69	4.36
338.2286	3.46	2.71	4.38

338.2858	3.46	2.71	4.38
338.3428	3.43	2.72	4.37
338.4	3.45	2.74	4.38
338.4572	3.46	2.73	4.38
338.5142	3.48	2.69	4.36
338.5714	3.47	2.7	4.35
338.6286	3.45	2.69	4.37
338.6858	3.5	2.64	4.35
338.7428	3.51	2.63	4.36
338.8	3.5	2.61	4.37
338.8572	3.52	2.6	4.35
338.9142	3.53	2.58	4.33
338.9714	3.51	2.6	4.34
339.0286	3.51	2.6	4.34
339.0858	3.51	2.65	4.34
339.1428	3.51	2.62	4.31
339.2	3.51	2.63	4.3
339.2572	3.46	2.65	4.31
339.3142	3.45	2.65	4.3
339.3714	3.46	2.63	4.28
339.4286	3.43	2.63	4.27
339.4858	3.45	2.61	4.24
339.5428	3.48	2.59	4.23
339.6	3.47	2.58	4.23
339.6572	3.44	2.58	4.2
339.7142	3.44	2.6	4.21
339.7714	3.46	2.6	4.22
339.8286	3.43	2.62	4.22
339.8858	3.42	2.64	4.22
339.9428	3.41	2.64	4.22
340	3.42	2.66	4.22
340.0572	3.4	2.68	4.23
340.1142	3.37	2.67	4.23
340.1714	3.38	2.67	4.25
340.2286	3.4	2.64	4.26
340.2858	3.4	2.67	4.26
340.3428	3.38	2.67	4.26
340.4	3.41	2.65	4.28
340.4572	3.41	2.64	4.28
340.5142	3.42	2.66	4.29
340.5714	3.46	2.62	4.3
340.6286	3.47	2.62	4.28
340.6858	3.47	2.62	4.27
340.7428	3.5	2.61	4.28
340.8	3.49	2.61	4.28
340.8572	3.51	2.59	4.28
340.9142	3.5	2.58	4.26

340.9714	3.49	2.6	4.27
341.0286	3.51	2.57	4.25
341.0858	3.51	2.56	4.27
341.1428	3.49	2.58	4.27
341.2	3.47	2.59	4.28
341.2572	3.46	2.61	4.29
341.3142	3.45	2.6	4.29
341.3714	3.44	2.61	4.28
341.4286	3.4	2.63	4.26
341.4858	3.4	2.61	4.26
341.5428	3.43	2.6	4.25
341.6	3.44	2.59	4.27
341.6572	3.45	2.59	4.25
341.7142	3.45	2.59	4.22
341.7714	3.43	2.58	4.23
341.8286	3.44	2.54	4.22
341.8858	3.45	2.55	4.22
341.9428	3.42	2.56	4.21
342	3.47	2.57	4.18
342.0572	3.46	2.59	4.16
342.1142	3.41	2.64	4.14
342.1714	3.4	2.65	4.14
342.2286	3.37	2.65	4.13
342.2858	3.34	2.66	4.14
342.3428	3.34	2.66	4.14
342.4	3.35	2.65	4.17
342.4572	3.31	2.69	4.17
342.5142	3.31	2.72	4.17
342.5714	3.33	2.71	4.15
342.6286	3.31	2.73	4.16
342.6858	3.3	2.71	4.17
342.7428	3.31	2.67	4.16
342.8	3.31	2.66	4.16
342.8572	3.3	2.66	4.15
342.9142	3.3	2.66	4.15
342.9714	3.33	2.64	4.17
343.0286	3.35	2.63	4.17
343.0858	3.38	2.61	4.17
343.1428	3.42	2.58	4.16
343.2	3.39	2.58	4.15
343.2572	3.36	2.56	4.16
343.3142	3.38	2.54	4.14
343.3714	3.38	2.54	4.11
343.4286	3.41	2.53	4.1
343.4858	3.4	2.54	4.1
343.5428	3.41	2.54	4.1
343.6	3.4	2.52	4.11

343.6572	3.43	2.54	4.1
343.7142	3.41	2.58	4.1
343.7714	3.4	2.58	4.1
343.8286	3.39	2.6	4.12
343.8858	3.4	2.61	4.12
343.9428	3.39	2.61	4.11
344	3.38	2.61	4.13
344.0572	3.36	2.62	4.15
344.1142	3.35	2.63	4.15
344.1714	3.33	2.64	4.16
344.2286	3.31	2.63	4.19
344.2858	3.33	2.59	4.21
344.3428	3.35	2.58	4.22
344.4	3.37	2.56	4.2
344.4572	3.37	2.56	4.2
344.5142	3.38	2.57	4.21
344.5714	3.38	2.58	4.2
344.6286	3.39	2.59	4.19
344.6858	3.38	2.56	4.18
344.7428	3.39	2.58	4.15
344.8	3.38	2.58	4.16
344.8572	3.34	2.6	4.16
344.9142	3.36	2.59	4.16
344.9714	3.31	2.62	4.15
345.0286	3.29	2.64	4.15
345.0858	3.25	2.66	4.14
345.1428	3.25	2.65	4.14
345.2	3.24	2.65	4.13
345.2572	3.24	2.66	4.12
345.3142	3.27	2.63	4.11
345.3714	3.24	2.65	4.11
345.4286	3.27	2.65	4.11
345.4858	3.3	2.66	4.09
345.5428	3.37	2.62	4.12
345.6	3.39	2.6	4.12
345.6572	3.4	2.59	4.12
345.7142	3.39	2.59	4.11
345.7714	3.39	2.59	4.11
345.8286	3.41	2.58	4.11
345.8858	3.46	2.6	4.08
345.9428	3.43	2.64	4.09
346	3.44	2.62	4.11
346.0572	3.42	2.63	4.11
346.1142	3.41	2.61	4.1
346.1714	3.38	2.61	4.1
346.2286	3.34	2.64	4.11
346.2858	3.34	2.61	4.12

346.3428	3.34	2.61	4.12
346.4	3.34	2.62	4.11
346.4572	3.36	2.65	4.12
346.5142	3.3	2.65	4.14
346.5714	3.29	2.61	4.14
346.6286	3.29	2.61	4.14
346.6858	3.28	2.59	4.14
346.7428	3.28	2.6	4.15
346.8	3.25	2.61	4.15
346.8572	3.26	2.59	4.17
346.9142	3.26	2.59	4.16
346.9714	3.26	2.59	4.15
347.0286	3.21	2.58	4.17
347.0858	3.21	2.58	4.16
347.1428	3.22	2.55	4.16
347.2	3.22	2.57	4.16
347.2572	3.18	2.58	4.17
347.3142	3.2	2.57	4.17
347.3714	3.22	2.55	4.13
347.4286	3.22	2.54	4.12
347.4858	3.21	2.53	4.1
347.5428	3.21	2.54	4.09
347.6	3.23	2.5	4.11
347.6572	3.25	2.48	4.11
347.7142	3.25	2.5	4.1
347.7714	3.22	2.52	4.11
347.8286	3.23	2.5	4.09
347.8858	3.24	2.51	4.05
347.9428	3.23	2.53	4.04
348	3.21	2.56	4.06
348.0572	3.22	2.57	4.07
348.1142	3.22	2.56	4.04
348.1714	3.21	2.6	4.03
348.2286	3.22	2.58	4.02
348.2858	3.21	2.57	4.02
348.3428	3.24	2.55	4.01
348.4	3.26	2.53	4.02
348.4572	3.24	2.51	4.02
348.5142	3.24	2.5	4.01
348.5714	3.25	2.48	4
348.6286	3.24	2.48	4.01
348.6858	3.25	2.5	3.98
348.7428	3.28	2.48	3.98
348.8	3.27	2.48	3.98
348.8572	3.29	2.48	3.96
348.9142	3.28	2.5	3.95
348.9714	3.26	2.52	3.94

349.0286	3.27	2.54	3.96
349.0858	3.25	2.54	3.96
349.1428	3.24	2.57	3.94
349.2	3.24	2.59	3.94
349.2572	3.23	2.59	3.95
349.3142	3.22	2.59	3.96
349.3714	3.21	2.63	3.96
349.4286	3.21	2.65	3.97
349.4858	3.24	2.61	3.99
349.5428	3.26	2.6	4
349.6	3.24	2.59	4.02
349.6572	3.25	2.61	4.02
349.7142	3.25	2.61	4.04
349.7714	3.26	2.59	4.05
349.8286	3.29	2.55	4.06
349.8858	3.25	2.56	4.08
349.9428	3.27	2.54	4.06
350	3.25	2.53	4.08
350.0572	3.24	2.53	4.12
350.1142	3.22	2.54	4.11
350.1714	3.24	2.54	4.1
350.2286	3.27	2.53	4.1
350.2858	3.24	2.52	4.1
350.3428	3.22	2.54	4.09
350.4	3.18	2.57	4.08
350.4572	3.18	2.61	4.05
350.5142	3.18	2.6	4.05
350.5714	3.16	2.62	4.04
350.6286	3.18	2.59	4.02
350.6858	3.19	2.58	4.02
350.7428	3.18	2.6	4.01
350.8	3.17	2.59	3.99
350.8572	3.16	2.58	4
350.9142	3.17	2.58	3.98
350.9714	3.16	2.57	3.97
351.0286	3.16	2.57	3.97
351.0858	3.18	2.56	3.98
351.1428	3.21	2.53	3.96
351.2	3.19	2.55	3.94
351.2572	3.18	2.57	3.92
351.3142	3.18	2.55	3.91
351.3714	3.2	2.53	3.93
351.4286	3.23	2.53	3.91
351.4858	3.22	2.51	3.92
351.5428	3.24	2.51	3.93
351.6	3.25	2.48	3.93
351.6572	3.22	2.47	3.92

351.7142	3.2	2.48	3.93
351.7714	3.2	2.48	3.9
351.8286	3.2	2.47	3.92
351.8858	3.22	2.46	3.91
351.9428	3.19	2.47	3.92
352	3.22	2.47	3.92
352.0572	3.26	2.46	3.93
352.1142	3.25	2.47	3.94
352.1714	3.27	2.47	3.95
352.2286	3.28	2.48	3.95
352.2858	3.27	2.5	3.95
352.3428	3.28	2.51	3.96
352.4	3.3	2.51	3.96
352.4572	3.29	2.53	3.96
352.5142	3.3	2.54	3.96
352.5714	3.29	2.56	3.95
352.6286	3.26	2.56	3.97
352.6858	3.26	2.56	3.98
352.7428	3.23	2.55	3.97
352.8	3.22	2.55	3.97
352.8572	3.24	2.51	3.97
352.9142	3.22	2.49	3.95
352.9714	3.18	2.51	3.95
353.0286	3.17	2.52	3.95
353.0858	3.14	2.54	3.95
353.1428	3.12	2.55	3.95
353.2	3.11	2.54	3.96
353.2572	3.13	2.55	3.95
353.3142	3.13	2.54	3.94
353.3714	3.15	2.55	3.92
353.4286	3.15	2.55	3.92
353.4858	3.15	2.57	3.9
353.5428	3.17	2.56	3.9
353.6	3.16	2.55	3.9
353.6572	3.17	2.53	3.89
353.7142	3.19	2.5	3.88
353.7714	3.22	2.48	3.88
353.8286	3.21	2.47	3.88
353.8858	3.24	2.45	3.88
353.9428	3.22	2.45	3.86
354	3.21	2.45	3.86
354.0572	3.2	2.44	3.89
354.1142	3.2	2.45	3.89
354.1714	3.22	2.41	3.89
354.2286	3.22	2.43	3.87
354.2858	3.2	2.43	3.89
354.3428	3.19	2.45	3.91

354.4	3.17	2.44	3.92
354.4572	3.16	2.45	3.91
354.5142	3.17	2.46	3.9
354.5714	3.2	2.48	3.9
354.6286	3.19	2.5	3.89
354.6858	3.21	2.48	3.9
354.7428	3.18	2.49	3.91
354.8	3.18	2.49	3.89
354.8572	3.15	2.5	3.89
354.9142	3.14	2.51	3.89
354.9714	3.15	2.52	3.87
355.0286	3.12	2.53	3.85
355.0858	3.11	2.51	3.86
355.1428	3.09	2.5	3.88
355.2	3.1	2.46	3.88
355.2572	3.09	2.48	3.86
355.3142	3.1	2.51	3.84
355.3714	3.11	2.55	3.85
355.4286	3.16	2.53	3.84
355.4858	3.18	2.52	3.85
355.5428	3.16	2.55	3.86
355.6	3.18	2.53	3.86
355.6572	3.19	2.55	3.85
355.7142	3.18	2.56	3.85
355.7714	3.19	2.57	3.84
355.8286	3.17	2.56	3.82
355.8858	3.17	2.56	3.8
355.9428	3.12	2.5	3.8
356	3.1	2.5	3.78
356.0572	3.09	2.49	3.78
356.1142	3.11	2.44	3.78
356.1714	3.11	2.42	3.79
356.2286	3.12	2.41	3.81
356.2858	3.11	2.38	3.79
356.3428	3.1	2.41	3.81
356.4	3.08	2.43	3.81
356.4572	3.11	2.41	3.79
356.5142	3.11	2.43	3.8
356.5714	3.11	2.43	3.8
356.6286	3.12	2.45	3.79
356.6858	3.11	2.5	3.81
356.7428	3.06	2.55	3.83
356.8	3.06	2.57	3.87
356.8572	3.07	2.58	3.88
356.9142	3.06	2.56	3.88
356.9714	3.09	2.54	3.87
357.0286	3.08	2.52	3.86

357.0858	3.1	2.54	3.86
357.1428	3.12	2.53	3.84
357.2	3.12	2.51	3.84
357.2572	3.13	2.49	3.85
357.3142	3.15	2.46	3.85
357.3714	3.14	2.46	3.84
357.4286	3.16	2.45	3.82
357.4858	3.14	2.45	3.81
357.5428	3.13	2.47	3.79
357.6	3.12	2.46	3.78
357.6572	3.13	2.44	3.78
357.7142	3.1	2.45	3.75
357.7714	3.1	2.49	3.75
357.8286	3.11	2.47	3.77
357.8858	3.13	2.47	3.78
357.9428	3.12	2.45	3.77
358	3.11	2.45	3.75
358.0572	3.12	2.46	3.76
358.1142	3.1	2.46	3.75
358.1714	3.1	2.47	3.74
358.2286	3.08	2.48	3.75
358.2858	3.09	2.47	3.79
358.3428	3.12	2.44	3.83
358.4	3.12	2.45	3.83
358.4572	3.09	2.46	3.84
358.5142	3.08	2.48	3.84
358.5714	3.07	2.5	3.83
358.6286	3.07	2.5	3.83
358.6858	3.1	2.48	3.83
358.7428	3.12	2.48	3.83
358.8	3.12	2.47	3.84
358.8572	3.14	2.47	3.87
358.9142	3.09	2.5	3.88
358.9714	3.05	2.52	3.88
359.0286	3.04	2.51	3.89
359.0858	3.05	2.51	3.9
359.1428	3.06	2.49	3.89
359.2	3.05	2.47	3.84
359.2572	3.05	2.47	3.84
359.3142	3.01	2.49	3.85
359.3714	2.99	2.5	3.86
359.4286	2.95	2.51	3.86
359.4858	2.97	2.48	3.85
359.5428	3.02	2.46	3.84
359.6	3.03	2.44	3.83
359.6572	3.02	2.45	3.82
359.7142	3.03	2.44	3.82

359.7714	3.06	2.44	3.81
359.8286	3.04	2.44	3.81
359.8858	3.07	2.44	3.81
359.9428	3.06	2.43	3.8
360	3.08	2.39	3.8
360.0572	3.08	2.39	3.81
360.1142	3.07	2.41	3.79
360.1714	3.07	2.41	3.77
360.2286	3.1	2.39	3.76
360.2858	3.08	2.41	3.77
360.3428	3.09	2.42	3.79
360.4	3.09	2.41	3.79
360.4572	3.08	2.41	3.8
360.5142	3.12	2.41	3.8
360.5714	3.12	2.43	3.79
360.6286	3.12	2.45	3.78
360.6858	3.15	2.43	3.78
360.7428	3.12	2.42	3.75
360.8	3.13	2.42	3.74
360.8572	3.13	2.41	3.73
360.9142	3.09	2.43	3.73
360.9714	3.09	2.41	3.72
361.0286	3.11	2.38	3.73
361.0858	3.09	2.41	3.71
361.1428	3.1	2.41	3.7
361.2	3.11	2.4	3.69
361.2572	3.08	2.4	3.68
361.3142	3.11	2.41	3.68
361.3714	3.11	2.41	3.69
361.4286	3.11	2.4	3.69
361.4858	3.17	2.39	3.69
361.5428	3.16	2.41	3.69
361.6	3.13	2.43	3.69
361.6572	3.14	2.42	3.72
361.7142	3.11	2.41	3.72
361.7714	3.09	2.41	3.71
361.8286	3.09	2.43	3.71
361.8858	3.07	2.44	3.71
361.9428	3.06	2.45	3.71
362	3.07	2.44	3.73
362.0572	3.04	2.44	3.73
362.1142	3.04	2.45	3.71
362.1714	3.05	2.44	3.69
362.2286	3.03	2.45	3.69
362.2858	3.04	2.45	3.69
362.3428	3.05	2.44	3.67
362.4	3.03	2.43	3.66

362.4572	3.03	2.45	3.66
362.5142	2.99	2.45	3.66
362.5714	3.01	2.44	3.64
362.6286	3.03	2.41	3.65
362.6858	3.05	2.4	3.66
362.7428	3.06	2.4	3.65
362.8	3.07	2.38	3.65
362.8572	3.06	2.38	3.66
362.9142	3.03	2.42	3.65
362.9714	3.06	2.39	3.65
363.0286	3.07	2.38	3.65
363.0858	3.1	2.37	3.65
363.1428	3.08	2.39	3.65
363.2	3.07	2.44	3.66
363.2572	3.04	2.45	3.67
363.3142	3.02	2.44	3.66
363.3714	3.05	2.44	3.66
363.4286	3.06	2.42	3.65
363.4858	3.08	2.39	3.63
363.5428	3.07	2.43	3.65
363.6	3.05	2.43	3.64
363.6572	3.03	2.43	3.63
363.7142	3.01	2.4	3.65
363.7714	2.99	2.37	3.64
363.8286	3	2.37	3.64
363.8858	3.01	2.36	3.64
363.9428	2.99	2.39	3.64
364	3	2.38	3.63
364.0572	3.01	2.38	3.62
364.1142	2.98	2.39	3.61
364.1714	3	2.41	3.62
364.2286	2.98	2.42	3.61
364.2858	3.01	2.44	3.62
364.3428	3.02	2.45	3.62
364.4	3.03	2.48	3.63
364.4572	3.03	2.48	3.62
364.5142	3.04	2.48	3.63
364.5714	3.02	2.48	3.62
364.6286	3.02	2.49	3.6
364.6858	3.04	2.47	3.61
364.7428	3.05	2.46	3.63
364.8	3.07	2.42	3.63
364.8572	3.1	2.41	3.64
364.9142	3.11	2.37	3.63
364.9714	3.07	2.37	3.63
365.0286	3.07	2.37	3.63
365.0858	3.1	2.34	3.63

365.1428	3.12	2.34	3.66
365.2	3.11	2.34	3.65
365.2572	3.09	2.35	3.66
365.3142	3.07	2.36	3.67
365.3714	3.02	2.39	3.65
365.4286	2.98	2.39	3.65
365.4858	2.94	2.42	3.66
365.5428	2.96	2.39	3.65
365.6	2.97	2.39	3.65
365.6572	2.95	2.4	3.64
365.7142	2.93	2.39	3.63
365.7714	2.9	2.42	3.62
365.8286	2.89	2.42	3.61
365.8858	2.89	2.42	3.61
365.9428	2.93	2.4	3.62
366	2.93	2.4	3.62
366.0572	2.97	2.41	3.61
366.1142	2.98	2.41	3.59
366.1714	3	2.43	3.58
366.2286	3	2.46	3.58
366.2858	2.99	2.48	3.58
366.3428	2.99	2.47	3.58
366.4	3.02	2.45	3.59
366.4572	3.06	2.42	3.59
366.5142	3.06	2.42	3.58
366.5714	3.07	2.43	3.58
366.6286	3.08	2.43	3.59
366.6858	3.07	2.43	3.6
366.7428	3.06	2.4	3.62
366.8	3.08	2.38	3.6
366.8572	3.06	2.35	3.6
366.9142	3.05	2.36	3.61
366.9714	3.03	2.39	3.63
367.0286	3.01	2.4	3.63
367.0858	2.99	2.4	3.64
367.1428	3	2.37	3.65
367.2	2.99	2.37	3.64
367.2572	2.98	2.37	3.64
367.3142	2.96	2.38	3.63
367.3714	2.95	2.4	3.63
367.4286	2.94	2.41	3.66
367.4858	2.92	2.43	3.67
367.5428	2.91	2.43	3.66
367.6	2.93	2.43	3.67
367.6572	2.92	2.43	3.68
367.7142	2.92	2.46	3.69
367.7714	2.9	2.48	3.69

367.8286	2.9	2.49	3.67
367.8858	2.91	2.5	3.65
367.9428	2.9	2.49	3.65
368	2.89	2.47	3.64
368.0572	2.94	2.45	3.62
368.1142	2.93	2.45	3.65
368.1714	2.91	2.45	3.66
368.2286	2.93	2.44	3.67
368.2858	2.92	2.41	3.68
368.3428	2.91	2.38	3.67
368.4	2.93	2.37	3.66
368.4572	2.93	2.37	3.65
368.5142	2.95	2.39	3.64
368.5714	2.97	2.38	3.65
368.6286	2.98	2.37	3.67
368.6858	2.99	2.4	3.68
368.7428	3.02	2.37	3.7
368.8	3	2.37	3.71
368.8572	3.03	2.36	3.71
368.9142	3.05	2.39	3.7
368.9714	3	2.4	3.7
369.0286	3	2.37	3.67
369.0858	2.96	2.38	3.65
369.1428	2.94	2.39	3.62
369.2	2.89	2.43	3.58
369.2572	2.89	2.41	3.59
369.3142	2.89	2.39	3.58
369.3714	2.88	2.43	3.57
369.4286	2.86	2.46	3.56
369.4858	2.84	2.45	3.56
369.5428	2.85	2.47	3.52
369.6	2.87	2.45	3.5
369.6572	2.88	2.44	3.49
369.7142	2.92	2.42	3.52
369.7714	2.92	2.42	3.52
369.8286	2.92	2.41	3.54
369.8858	2.9	2.42	3.54
369.9428	2.92	2.38	3.53
370	2.9	2.37	3.53
370.0572	2.89	2.38	3.53
370.1142	2.89	2.35	3.53
370.1714	2.9	2.38	3.53
370.2286	2.89	2.35	3.55
370.2858	2.87	2.36	3.55
370.3428	2.87	2.34	3.54
370.4	2.87	2.34	3.55
370.4572	2.88	2.33	3.56

370.5142	2.85	2.35	3.55
370.5714	2.85	2.35	3.53
370.6286	2.88	2.32	3.51
370.6858	2.92	2.29	3.51
370.7428	2.89	2.28	3.49
370.8	2.88	2.3	3.47
370.8572	2.89	2.3	3.47
370.9142	2.91	2.29	3.49
370.9714	2.92	2.3	3.51
371.0286	2.9	2.3	3.51
371.0858	2.92	2.29	3.49
371.1428	2.92	2.28	3.48
371.2	2.94	2.28	3.49
371.2572	2.89	2.33	3.5
371.3142	2.88	2.34	3.49
371.3714	2.9	2.35	3.5
371.4286	2.89	2.33	3.52
371.4858	2.87	2.36	3.52
371.5428	2.88	2.37	3.53
371.6	2.88	2.37	3.54
371.6572	2.88	2.39	3.57
371.7142	2.88	2.41	3.58
371.7714	2.85	2.43	3.59
371.8286	2.87	2.45	3.58
371.8858	2.92	2.44	3.58
371.9428	2.92	2.45	3.59
372	2.92	2.44	3.58
372.0572	2.93	2.43	3.58
372.1142	2.93	2.42	3.55
372.1714	2.94	2.42	3.53
372.2286	2.92	2.42	3.52
372.2858	2.93	2.38	3.51
372.3428	2.93	2.37	3.48
372.4	2.94	2.35	3.47
372.4572	2.91	2.37	3.45
372.5142	2.91	2.38	3.45
372.5714	2.89	2.38	3.43
372.6286	2.89	2.4	3.46
372.6858	2.88	2.38	3.44
372.7428	2.88	2.38	3.44
372.8	2.88	2.39	3.45
372.8572	2.86	2.37	3.44
372.9142	2.88	2.39	3.44
372.9714	2.9	2.39	3.47
373.0286	2.88	2.39	3.48
373.0858	2.89	2.37	3.5
373.1428	2.88	2.37	3.5

373.2	2.88	2.36	3.51
373.2572	2.9	2.36	3.53
373.3142	2.9	2.36	3.53
373.3714	2.9	2.36	3.54
373.4286	2.88	2.38	3.56
373.4858	2.91	2.36	3.58
373.5428	2.9	2.4	3.55
373.6	2.87	2.43	3.54
373.6572	2.86	2.43	3.52
373.7142	2.86	2.42	3.54
373.7714	2.86	2.44	3.53
373.8286	2.86	2.44	3.54
373.8858	2.89	2.43	3.53
373.9428	2.91	2.43	3.5
374	2.91	2.4	3.48
374.0572	2.91	2.39	3.49
374.1142	2.92	2.36	3.5
374.1714	2.96	2.3	3.47
374.2286	2.96	2.31	3.47
374.2858	2.97	2.31	3.47
374.3428	2.95	2.29	3.47
374.4	2.94	2.3	3.47
374.4572	2.9	2.29	3.47
374.5142	2.88	2.31	3.46
374.5714	2.88	2.34	3.46
374.6286	2.84	2.36	3.45
374.6858	2.79	2.4	3.43
374.7428	2.78	2.43	3.43
374.8	2.76	2.46	3.45
374.8572	2.75	2.48	3.46
374.9142	2.76	2.45	3.48
374.9714	2.79	2.44	3.46
375.0286	2.8	2.44	3.46
375.0858	2.82	2.42	3.48
375.1428	2.79	2.4	3.46
375.2	2.81	2.39	3.45
375.2572	2.84	2.35	3.44
375.3142	2.81	2.38	3.43
375.3714	2.82	2.34	3.45
375.4286	2.83	2.3	3.45
375.4858	2.83	2.3	3.43
375.5428	2.81	2.33	3.44
375.6	2.83	2.32	3.43
375.6572	2.82	2.31	3.43
375.7142	2.83	2.3	3.42
375.7714	2.82	2.29	3.41
375.8286	2.8	2.32	3.39

375.8858	2.82	2.3	3.38
375.9428	2.82	2.29	3.4
376	2.81	2.34	3.38
376.0572	2.79	2.38	3.4
376.1142	2.81	2.36	3.4
376.1714	2.79	2.36	3.39
376.2286	2.78	2.38	3.39
376.2858	2.78	2.38	3.38
376.3428	2.79	2.39	3.36
376.4	2.78	2.41	3.37
376.4572	2.78	2.42	3.37
376.5142	2.76	2.41	3.37
376.5714	2.77	2.38	3.38
376.6286	2.76	2.37	3.36
376.6858	2.75	2.37	3.37
376.7428	2.75	2.38	3.39
376.8	2.75	2.38	3.4
376.8572	2.76	2.38	3.4
376.9142	2.76	2.38	3.4
376.9714	2.77	2.37	3.39
377.0286	2.76	2.36	3.39
377.0858	2.75	2.4	3.39
377.1428	2.76	2.43	3.37
377.2	2.76	2.42	3.38
377.2572	2.77	2.41	3.41
377.3142	2.76	2.44	3.4
377.3714	2.75	2.44	3.42
377.4286	2.76	2.44	3.42
377.4858	2.78	2.45	3.43
377.5428	2.75	2.43	3.44
377.6	2.74	2.39	3.43
377.6572	2.76	2.35	3.43
377.7142	2.76	2.31	3.42
377.7714	2.74	2.34	3.43
377.8286	2.75	2.31	3.44
377.8858	2.77	2.28	3.45
377.9428	2.78	2.27	3.45
378	2.76	2.28	3.45
378.0572	2.74	2.24	3.44
378.1142	2.78	2.23	3.43
378.1714	2.8	2.22	3.42
378.2286	2.81	2.23	3.43
378.2858	2.82	2.21	3.41
378.3428	2.83	2.2	3.41
378.4	2.8	2.24	3.38
378.4572	2.8	2.26	3.39
378.5142	2.79	2.29	3.4

378.5714	2.8	2.28	3.4
378.6286	2.78	2.32	3.39
378.6858	2.76	2.32	3.35
378.7428	2.74	2.36	3.35
378.8	2.74	2.37	3.35
378.8572	2.73	2.36	3.33
378.9142	2.74	2.34	3.35
378.9714	2.79	2.32	3.36
379.0286	2.78	2.32	3.35
379.0858	2.78	2.32	3.34
379.1428	2.79	2.31	3.32
379.2	2.79	2.31	3.33
379.2572	2.78	2.34	3.35
379.3142	2.78	2.34	3.37
379.3714	2.77	2.31	3.38
379.4286	2.75	2.34	3.36
379.4858	2.75	2.34	3.36
379.5428	2.74	2.36	3.38
379.6	2.74	2.34	3.41
379.6572	2.75	2.33	3.42
379.7142	2.72	2.37	3.42
379.7714	2.73	2.37	3.41
379.8286	2.78	2.34	3.4
379.8858	2.8	2.33	3.41
379.9428	2.82	2.35	3.43
380	2.79	2.36	3.44
380.0572	2.79	2.37	3.44
380.1142	2.79	2.38	3.43
380.1714	2.8	2.37	3.42
380.2286	2.8	2.38	3.42
380.2858	2.82	2.35	3.42
380.3428	2.8	2.35	3.42
380.4	2.79	2.37	3.41
380.4572	2.78	2.35	3.4
380.5142	2.77	2.37	3.38
380.5714	2.8	2.36	3.34
380.6286	2.8	2.37	3.36
380.6858	2.8	2.35	3.35
380.7428	2.76	2.35	3.36
380.8	2.74	2.36	3.37
380.8572	2.72	2.37	3.36
380.9142	2.73	2.34	3.36
380.9714	2.73	2.33	3.37
381.0286	2.74	2.33	3.38
381.0858	2.75	2.29	3.39
381.1428	2.76	2.27	3.39
381.2	2.76	2.27	3.38

381.2572	2.74	2.3	3.37
381.3142	2.76	2.3	3.37
381.3714	2.78	2.28	3.38
381.4286	2.8	2.25	3.39
381.4858	2.79	2.28	3.4
381.5428	2.79	2.29	3.39
381.6	2.78	2.28	3.37
381.6572	2.78	2.31	3.36
381.7142	2.78	2.33	3.34
381.7714	2.81	2.31	3.34
381.8286	2.81	2.29	3.33
381.8858	2.78	2.32	3.34
381.9428	2.79	2.32	3.34
382	2.77	2.35	3.34
382.0572	2.77	2.32	3.35
382.1142	2.8	2.32	3.35
382.1714	2.81	2.31	3.36
382.2286	2.78	2.32	3.37
382.2858	2.76	2.32	3.39
382.3428	2.76	2.33	3.39
382.4	2.76	2.33	3.38
382.4572	2.78	2.31	3.4
382.5142	2.76	2.33	3.41
382.5714	2.78	2.31	3.39
382.6286	2.8	2.31	3.37
382.6858	2.77	2.29	3.37
382.7428	2.76	2.29	3.38
382.8	2.77	2.29	3.36
382.8572	2.8	2.27	3.34
382.9142	2.77	2.28	3.32
382.9714	2.75	2.27	3.33
383.0286	2.75	2.28	3.34
383.0858	2.75	2.27	3.33
383.1428	2.73	2.29	3.3
383.2	2.74	2.28	3.27
383.2572	2.75	2.28	3.27
383.3142	2.75	2.3	3.27
383.3714	2.76	2.29	3.28
383.4286	2.72	2.3	3.27
383.4858	2.76	2.29	3.27
383.5428	2.78	2.27	3.28
383.6	2.81	2.23	3.28
383.6572	2.83	2.22	3.28
383.7142	2.84	2.22	3.32
383.7714	2.85	2.22	3.31
383.8286	2.82	2.26	3.31
383.8858	2.82	2.23	3.32

383.9428	2.81	2.25	3.3
384	2.83	2.25	3.32
384.0572	2.82	2.23	3.33
384.1142	2.79	2.25	3.34
384.1714	2.74	2.28	3.35
384.2286	2.71	2.3	3.36
384.2858	2.72	2.29	3.38
384.3428	2.72	2.29	3.37
384.4	2.71	2.26	3.4
384.4572	2.72	2.26	3.4
384.5142	2.73	2.24	3.41
384.5714	2.71	2.23	3.41
384.6286	2.72	2.23	3.39
384.6858	2.78	2.2	3.4
384.7428	2.79	2.19	3.39
384.8	2.81	2.17	3.37
384.8572	2.81	2.19	3.36
384.9142	2.79	2.21	3.34
384.9714	2.79	2.22	3.34
385.0286	2.77	2.26	3.32
385.0858	2.74	2.29	3.31
385.1428	2.75	2.28	3.29
385.2	2.71	2.29	3.27
385.2572	2.67	2.31	3.28
385.3142	2.67	2.32	3.27
385.3714	2.66	2.32	3.27
385.4286	2.65	2.28	3.27
385.4858	2.64	2.28	3.27
385.5428	2.68	2.25	3.28
385.6	2.71	2.22	3.26
385.6572	2.72	2.23	3.25
385.7142	2.7	2.26	3.26
385.7714	2.72	2.25	3.26
385.8286	2.73	2.27	3.24
385.8858	2.72	2.25	3.25
385.9428	2.72	2.27	3.28
386	2.73	2.28	3.28
386.0572	2.75	2.26	3.29
386.1142	2.74	2.27	3.29
386.1714	2.71	2.28	3.29
386.2286	2.7	2.27	3.29
386.2858	2.71	2.26	3.29
386.3428	2.67	2.3	3.26
386.4	2.66	2.31	3.26
386.4572	2.66	2.31	3.25
386.5142	2.67	2.3	3.26
386.5714	2.66	2.3	3.26

386.6286	2.65	2.3	3.25
386.6858	2.66	2.27	3.25
386.7428	2.64	2.29	3.26
386.8	2.64	2.29	3.25
386.8572	2.63	2.29	3.23
386.9142	2.68	2.25	3.21
386.9714	2.69	2.25	3.19
387.0286	2.67	2.25	3.18
387.0858	2.67	2.26	3.18
387.1428	2.68	2.22	3.16
387.2	2.68	2.24	3.16
387.2572	2.68	2.27	3.16
387.3142	2.7	2.24	3.16
387.3714	2.71	2.21	3.17
387.4286	2.71	2.22	3.14
387.4858	2.67	2.22	3.15
387.5428	2.68	2.22	3.14
387.6	2.69	2.21	3.15
387.6572	2.68	2.19	3.15
387.7142	2.68	2.21	3.15
387.7714	2.68	2.22	3.14
387.8286	2.66	2.23	3.16
387.8858	2.66	2.22	3.21
387.9428	2.67	2.22	3.22
388	2.67	2.21	3.22
388.0572	2.67	2.21	3.24
388.1142	2.68	2.18	3.23
388.1714	2.68	2.18	3.22
388.2286	2.69	2.18	3.21
388.2858	2.7	2.18	3.21
388.3428	2.69	2.17	3.2
388.4	2.7	2.15	3.21
388.4572	2.69	2.19	3.23
388.5142	2.68	2.19	3.24
388.5714	2.68	2.19	3.24
388.6286	2.66	2.19	3.25
388.6858	2.65	2.18	3.24
388.7428	2.65	2.19	3.24
388.8	2.63	2.21	3.22
388.8572	2.6	2.24	3.24
388.9142	2.63	2.25	3.23
388.9714	2.64	2.23	3.22
389.0286	2.64	2.2	3.24
389.0858	2.63	2.24	3.25
389.1428	2.64	2.23	3.26
389.2	2.67	2.27	3.29
389.2572	2.66	2.31	3.3

389.3142	2.65	2.3	3.3
389.3714	2.65	2.28	3.29
389.4286	2.63	2.28	3.29
389.4858	2.62	2.27	3.28
389.5428	2.59	2.31	3.27
389.6	2.58	2.33	3.24
389.6572	2.55	2.31	3.24
389.7142	2.57	2.33	3.24
389.7714	2.57	2.28	3.23
389.8286	2.58	2.25	3.22
389.8858	2.59	2.24	3.22
389.9428	2.63	2.21	3.22
390	2.63	2.21	3.23
390.0572	2.66	2.24	3.21
390.1142	2.65	2.22	3.21
390.1714	2.69	2.2	3.21
390.2286	2.7	2.2	3.2
390.2858	2.69	2.17	3.17
390.3428	2.68	2.19	3.15
390.4	2.64	2.22	3.14
390.4572	2.66	2.22	3.15
390.5142	2.63	2.25	3.16
390.5714	2.62	2.26	3.15
390.6286	2.61	2.26	3.14
390.6858	2.61	2.26	3.13
390.7428	2.6	2.28	3.12
390.8	2.61	2.25	3.11
390.8572	2.62	2.25	3.11
390.9142	2.61	2.26	3.11
390.9714	2.59	2.24	3.1
391.0286	2.58	2.27	3.1
391.0858	2.55	2.29	3.1
391.1428	2.57	2.28	3.11
391.2	2.57	2.24	3.11
391.2572	2.56	2.26	3.12
391.3142	2.54	2.25	3.13
391.3714	2.54	2.26	3.12
391.4286	2.54	2.27	3.12
391.4858	2.55	2.28	3.14
391.5428	2.58	2.26	3.13
391.6	2.62	2.24	3.14
391.6572	2.64	2.24	3.13
391.7142	2.65	2.23	3.16
391.7714	2.66	2.24	3.15
391.8286	2.7	2.22	3.15
391.8858	2.71	2.23	3.16
391.9428	2.7	2.23	3.14

392	2.71	2.23	3.15
392.0572	2.71	2.22	3.16
392.1142	2.71	2.22	3.17
392.1714	2.7	2.22	3.19
392.2286	2.68	2.21	3.19
392.2858	2.65	2.21	3.2
392.3428	2.64	2.2	3.2
392.4	2.63	2.2	3.19
392.4572	2.64	2.19	3.2
392.5142	2.66	2.18	3.21
392.5714	2.64	2.19	3.21
392.6286	2.65	2.21	3.19
392.6858	2.65	2.21	3.18
392.7428	2.69	2.21	3.18
392.8	2.66	2.25	3.17
392.8572	2.68	2.25	3.17
392.9142	2.68	2.27	3.18
392.9714	2.66	2.3	3.16
393.0286	2.64	2.32	3.16
393.0858	2.61	2.35	3.14
393.1428	2.61	2.36	3.14
393.2	2.58	2.38	3.14
393.2572	2.57	2.37	3.13
393.3142	2.53	2.41	3.13
393.3714	2.56	2.36	3.12
393.4286	2.59	2.35	3.11
393.4858	2.58	2.32	3.12
393.5428	2.59	2.29	3.13
393.6	2.6	2.26	3.13
393.6572	2.65	2.22	3.12
393.7142	2.68	2.18	3.14
393.7714	2.67	2.18	3.14
393.8286	2.68	2.18	3.14
393.8858	2.69	2.14	3.16
393.9428	2.67	2.12	3.17
394	2.67	2.1	3.15
394.0572	2.66	2.13	3.14
394.1142	2.63	2.16	3.14
394.1714	2.62	2.15	3.13
394.2286	2.6	2.14	3.15
394.2858	2.58	2.15	3.17
394.3428	2.6	2.15	3.17
394.4	2.59	2.17	3.17
394.4572	2.61	2.18	3.17
394.5142	2.61	2.2	3.18
394.5714	2.59	2.23	3.16
394.6286	2.61	2.19	3.16

394.6858	2.62	2.19	3.16
394.7428	2.61	2.19	3.14
394.8	2.61	2.22	3.13
394.8572	2.59	2.22	3.12
394.9142	2.59	2.19	3.12
394.9714	2.6	2.14	3.11
395.0286	2.58	2.15	3.11
395.0858	2.56	2.17	3.1
395.1428	2.57	2.16	3.08
395.2	2.57	2.17	3.07
395.2572	2.6	2.18	3.05
395.3142	2.62	2.19	3.05
395.3714	2.62	2.21	3.05
395.4286	2.62	2.21	3.08
395.4858	2.61	2.23	3.08
395.5428	2.59	2.25	3.06
395.6	2.59	2.24	3.08
395.6572	2.58	2.22	3.09
395.7142	2.57	2.23	3.09
395.7714	2.54	2.24	3.09
395.8286	2.53	2.25	3.12
395.8858	2.5	2.25	3.14
395.9428	2.51	2.22	3.14
396	2.49	2.23	3.14
396.0572	2.49	2.24	3.14
396.1142	2.49	2.25	3.14
396.1714	2.47	2.27	3.15
396.2286	2.45	2.27	3.15
396.2858	2.48	2.23	3.16
396.3428	2.48	2.24	3.15
396.4	2.45	2.23	3.15
396.4572	2.47	2.23	3.15
396.5142	2.44	2.25	3.14
396.5714	2.45	2.26	3.14
396.6286	2.45	2.27	3.12
396.6858	2.44	2.26	3.13
396.7428	2.48	2.26	3.12
396.8	2.53	2.24	3.12
396.8572	2.53	2.27	3.12
396.9142	2.54	2.24	3.13
396.9714	2.57	2.23	3.11
397.0286	2.57	2.22	3.11
397.0858	2.59	2.21	3.11
397.1428	2.57	2.21	3.09
397.2	2.59	2.2	3.05
397.2572	2.62	2.19	3.08
397.3142	2.61	2.19	3.1

397.3714	2.58	2.2	3.1
397.4286	2.56	2.22	3.11
397.4858	2.55	2.24	3.09
397.5428	2.55	2.23	3.11
397.6	2.54	2.23	3.07
397.6572	2.56	2.21	3.06
397.7142	2.59	2.18	3.07
397.7714	2.56	2.2	3.06
397.8286	2.54	2.22	3.05
397.8858	2.54	2.2	3.05
397.9428	2.54	2.21	3.04
398	2.55	2.2	3.03
398.0572	2.58	2.19	3.04
398.1142	2.57	2.18	3.05
398.1714	2.59	2.19	3.02
398.2286	2.55	2.19	2.98
398.2858	2.55	2.22	2.97
398.3428	2.55	2.2	2.95
398.4	2.56	2.2	2.95
398.4572	2.54	2.19	2.95
398.5142	2.52	2.21	2.97
398.5714	2.51	2.2	2.98
398.6286	2.49	2.21	2.98
398.6858	2.5	2.22	3.01
398.7428	2.49	2.21	3.03
398.8	2.5	2.22	3.05
398.8572	2.49	2.22	3.06
398.9142	2.51	2.21	3.08
398.9714	2.54	2.18	3.08
399.0286	2.56	2.18	3.08
399.0858	2.57	2.15	3.1
399.1428	2.58	2.13	3.11
399.2	2.57	2.12	3.11
399.2572	2.56	2.12	3.11
399.3142	2.59	2.12	3.12
399.3714	2.6	2.1	3.13
399.4286	2.64	2.08	3.12
399.4858	2.64	2.08	3.12
399.5428	2.62	2.11	3.1
399.6	2.58	2.09	3.09
399.6572	2.6	2.06	3.08
399.7142	2.62	2.08	3.08
399.7714	2.65	2.09	3.06
399.8286	2.65	2.11	3.05
399.8858	2.62	2.14	3.04
399.9428	2.6	2.14	3.03
400	2.59	2.14	3.01

400.0572	2.56	2.15	3
400.1142	2.54	2.15	2.99
400.1714	2.57	2.17	2.99
400.2286	2.54	2.21	3.01
400.2858	2.54	2.21	3.01
400.3428	2.52	2.22	3.02
400.4	2.52	2.21	3.03
400.4572	2.51	2.19	3.02
400.5142	2.49	2.17	3.03
400.5714	2.49	2.2	3.03
400.6286	2.45	2.25	3.02
400.6858	2.46	2.25	3.02
400.7428	2.49	2.24	3.04
400.8	2.48	2.25	3.06
400.8572	2.49	2.26	3.06
400.9142	2.49	2.24	3.04
400.9714	2.48	2.24	3.04
401.0286	2.47	2.26	3.06
401.0858	2.51	2.28	3.09
401.1428	2.51	2.28	3.09
401.2	2.52	2.27	3.07
401.2572	2.52	2.28	3.06
401.3142	2.51	2.31	3.05
401.3714	2.51	2.3	3.07
401.4286	2.49	2.29	3.05
401.4858	2.5	2.27	3.06
401.5428	2.5	2.29	3.07
401.6	2.51	2.27	3.07
401.6572	2.51	2.25	3.07
401.7142	2.48	2.22	3.06
401.7714	2.49	2.21	3.06
401.8286	2.49	2.19	3.1
401.8858	2.51	2.15	3.07
401.9428	2.5	2.13	3.06
402	2.5	2.13	3.04
402.0572	2.51	2.13	3.03
402.1142	2.53	2.13	3.03
402.1714	2.51	2.15	3.04
402.2286	2.49	2.17	3.04
402.2858	2.51	2.14	3.03
402.3428	2.52	2.16	3.02
402.4	2.54	2.15	3.01
402.4572	2.55	2.17	3
402.5142	2.55	2.17	3
402.5714	2.56	2.18	2.99
402.6286	2.54	2.2	2.99
402.6858	2.51	2.21	2.97

402.7428	2.5	2.19	2.97
402.8	2.51	2.18	2.99
402.8572	2.49	2.18	3
402.9142	2.51	2.15	3
402.9714	2.48	2.15	3.01
403.0286	2.48	2.16	3
403.0858	2.48	2.14	2.99
403.1428	2.48	2.13	2.99
403.2	2.45	2.13	3
403.2572	2.48	2.08	3.02
403.3142	2.51	2.06	3.01
403.3714	2.55	2.05	3
403.4286	2.53	2.08	3
403.4858	2.51	2.1	3.01
403.5428	2.51	2.09	3.02
403.6	2.5	2.08	3.02
403.6572	2.5	2.08	3.02
403.7142	2.51	2.09	3.01
403.7714	2.54	2.09	2.99
403.8286	2.54	2.09	2.97
403.8858	2.52	2.1	2.98
403.9428	2.49	2.1	2.97
404	2.53	2.08	2.96
404.0572	2.55	2.03	2.97
404.1142	2.55	2.04	2.97
404.1714	2.55	2.05	2.95
404.2286	2.58	2.06	2.95
404.2858	2.57	2.07	2.94
404.3428	2.55	2.09	2.91
404.4	2.53	2.1	2.89
404.4572	2.52	2.13	2.91
404.5142	2.5	2.13	2.92
404.5714	2.48	2.17	2.93
404.6286	2.44	2.21	2.94
404.6858	2.43	2.21	2.97
404.7428	2.41	2.2	2.97
404.8	2.37	2.2	2.97
404.8572	2.38	2.2	3
404.9142	2.4	2.16	3.02
404.9714	2.41	2.16	3.02
405.0286	2.4	2.15	3.01
405.0858	2.42	2.17	3.02
405.1428	2.43	2.15	3.03
405.2	2.44	2.15	3.05
405.2572	2.42	2.15	3.06
405.3142	2.43	2.15	3.05
405.3714	2.46	2.14	3.04

405.4286	2.47	2.14	3.03
405.4858	2.48	2.13	3.02
405.5428	2.5	2.15	3.01
405.6	2.52	2.13	2.99
405.6572	2.51	2.13	2.99
405.7142	2.51	2.11	2.99
405.7714	2.49	2.11	2.96
405.8286	2.52	2.12	2.95
405.8858	2.54	2.12	2.94
405.9428	2.53	2.12	2.95
406	2.5	2.14	2.95
406.0572	2.49	2.14	2.96
406.1142	2.47	2.11	2.96
406.1714	2.43	2.12	2.97
406.2286	2.43	2.15	2.97
406.2858	2.42	2.17	2.98
406.3428	2.44	2.15	3
406.4	2.43	2.17	3
406.4572	2.44	2.18	3.01
406.5142	2.42	2.2	3.03
406.5714	2.45	2.17	3.02
406.6286	2.42	2.17	3.02
406.6858	2.45	2.21	3.03
406.7428	2.48	2.2	3.03
406.8	2.49	2.17	3.04
406.8572	2.48	2.13	3.05
406.9142	2.5	2.13	3.02
406.9714	2.5	2.09	3
407.0286	2.51	2.08	2.99
407.0858	2.51	2.04	2.99
407.1428	2.46	2.07	3.01
407.2	2.47	2.07	2.99
407.2572	2.47	2.06	2.98
407.3142	2.46	2.07	2.98
407.3714	2.46	2.05	2.95
407.4286	2.5	2.05	2.94
407.4858	2.47	2.06	2.96
407.5428	2.47	2.07	2.97
407.6	2.45	2.1	2.96
407.6572	2.43	2.13	2.96
407.7142	2.44	2.12	2.94
407.7714	2.45	2.12	2.93
407.8286	2.44	2.11	2.95
407.8858	2.41	2.12	2.96
407.9428	2.42	2.14	2.97
408	2.4	2.16	2.94
408.0572	2.4	2.16	2.92

408.1142	2.41	2.18	2.94
408.1714	2.39	2.15	2.93
408.2286	2.39	2.16	2.93
408.2858	2.39	2.18	2.94
408.3428	2.38	2.2	2.94
408.4	2.39	2.19	2.92
408.4572	2.43	2.17	2.91
408.5142	2.44	2.16	2.91
408.5714	2.45	2.14	2.91
408.6286	2.45	2.13	2.94
408.6858	2.46	2.11	2.95
408.7428	2.48	2.09	2.93
408.8	2.51	2.05	2.91
408.8572	2.5	2.04	2.9
408.9142	2.51	2.03	2.91
408.9714	2.46	2.04	2.89
409.0286	2.43	2.07	2.87
409.0858	2.42	2.09	2.88
409.1428	2.42	2.1	2.86
409.2	2.4	2.09	2.87
409.2572	2.4	2.1	2.87
409.3142	2.4	2.11	2.87
409.3714	2.39	2.11	2.84
409.4286	2.39	2.11	2.84
409.4858	2.39	2.11	2.82
409.5428	2.4	2.13	2.81
409.6	2.41	2.1	2.8
409.6572	2.41	2.1	2.82
409.7142	2.4	2.14	2.81
409.7714	2.41	2.14	2.82
409.8286	2.4	2.16	2.82
409.8858	2.39	2.18	2.84
409.9428	2.4	2.2	2.83
410	2.42	2.18	2.84
410.0572	2.44	2.16	2.85
410.1142	2.42	2.18	2.84
410.1714	2.43	2.19	2.83
410.2286	2.43	2.18	2.84
410.2858	2.41	2.14	2.84
410.3428	2.4	2.13	2.85
410.4	2.38	2.13	2.84
410.4572	2.39	2.13	2.84
410.5142	2.38	2.13	2.83
410.5714	2.36	2.13	2.82
410.6286	2.34	2.14	2.84
410.6858	2.39	2.1	2.86
410.7428	2.39	2.1	2.87

410.8	2.38	2.09	2.86
410.8572	2.38	2.1	2.85
410.9142	2.42	2.11	2.86
410.9714	2.41	2.11	2.86
411.0286	2.37	2.14	2.87
411.0858	2.36	2.15	2.88
411.1428	2.35	2.15	2.88
411.2	2.36	2.14	2.91
411.2572	2.35	2.15	2.91
411.3142	2.34	2.15	2.92
411.3714	2.35	2.14	2.94
411.4286	2.37	2.13	2.96
411.4858	2.29	2.19	2.97
411.5428	2.31	2.19	2.96
411.6	2.34	2.16	2.93
411.6572	2.36	2.13	2.91
411.7142	2.34	2.13	2.92
411.7714	2.33	2.12	2.9
411.8286	2.35	2.1	2.89
411.8858	2.36	2.09	2.88
411.9428	2.36	2.1	2.87
412	2.35	2.1	2.88
412.0572	2.38	2.07	2.87
412.1142	2.38	2.06	2.86
412.1714	2.38	2.05	2.88
412.2286	2.38	2.06	2.87
412.2858	2.41	2.05	2.86
412.3428	2.42	2.05	2.86
412.4	2.39	2.07	2.85
412.4572	2.37	2.1	2.85
412.5142	2.36	2.11	2.86
412.5714	2.37	2.12	2.85
412.6286	2.39	2.1	2.87
412.6858	2.4	2.1	2.88
412.7428	2.43	2.1	2.87
412.8	2.42	2.1	2.84
412.8572	2.41	2.1	2.85
412.9142	2.42	2.09	2.84
412.9714	2.45	2.07	2.85
413.0286	2.45	2.06	2.86
413.0858	2.44	2.04	2.84
413.1428	2.42	2.04	2.82
413.2	2.4	2.05	2.83
413.2572	2.41	2.05	2.83
413.3142	2.37	2.07	2.84
413.3714	2.36	2.09	2.81
413.4286	2.36	2.11	2.8

413.4858	2.33	2.1	2.82
413.5428	2.3	2.14	2.81
413.6	2.31	2.13	2.81
413.6572	2.28	2.16	2.83
413.7142	2.29	2.15	2.85
413.7714	2.3	2.13	2.87
413.8286	2.3	2.13	2.88
413.8858	2.32	2.1	2.85
413.9428	2.33	2.07	2.85
414	2.31	2.09	2.85
414.0572	2.31	2.11	2.84
414.1142	2.31	2.1	2.84
414.1714	2.29	2.1	2.81
414.2286	2.32	2.09	2.81
414.2858	2.32	2.1	2.84
414.3428	2.3	2.14	2.85
414.4	2.33	2.14	2.85
414.4572	2.32	2.15	2.85
414.5142	2.31	2.16	2.84
414.5714	2.35	2.13	2.84
414.6286	2.37	2.13	2.83
414.6858	2.37	2.09	2.8
414.7428	2.37	2.11	2.79
414.8	2.36	2.09	2.8
414.8572	2.38	2.07	2.8
414.9142	2.39	2.05	2.8
414.9714	2.36	2.06	2.82
415.0286	2.33	2.08	2.83
415.0858	2.32	2.08	2.84
415.1428	2.32	2.1	2.83
415.2	2.3	2.12	2.84
415.2572	2.32	2.12	2.84
415.3142	2.35	2.09	2.83
415.3714	2.35	2.09	2.82
415.4286	2.35	2.08	2.81
415.4858	2.34	2.08	2.83
415.5428	2.32	2.05	2.82
415.6	2.33	2.01	2.83
415.6572	2.35	2.03	2.81
415.7142	2.36	2.02	2.8
415.7714	2.36	2.02	2.79
415.8286	2.36	2.02	2.77
415.8858	2.33	2.05	2.77
415.9428	2.33	2.06	2.78
416	2.34	2.09	2.78
416.0572	2.32	2.09	2.78
416.1142	2.35	2.11	2.74

416.1714	2.34	2.13	2.74
416.2286	2.33	2.13	2.76
416.2858	2.32	2.14	2.79
416.3428	2.32	2.15	2.79
416.4	2.31	2.19	2.77
416.4572	2.32	2.17	2.77
416.5142	2.34	2.2	2.78
416.5714	2.31	2.18	2.78
416.6286	2.31	2.19	2.77
416.6858	2.31	2.19	2.76
416.7428	2.35	2.18	2.79
416.8	2.35	2.15	2.79
416.8572	2.36	2.13	2.76
416.9142	2.36	2.11	2.79
416.9714	2.35	2.08	2.8
417.0286	2.33	2.06	2.81
417.0858	2.34	2.03	2.78
417.1428	2.35	2.03	2.76
417.2	2.36	2.02	2.74
417.2572	2.37	2.01	2.73
417.3142	2.34	2.03	2.72
417.3714	2.34	2.05	2.72
417.4286	2.32	2.08	2.73
417.4858	2.3	2.11	2.76
417.5428	2.29	2.11	2.77
417.6	2.31	2.12	2.78
417.6572	2.28	2.15	2.76
417.7142	2.25	2.14	2.77
417.7714	2.26	2.16	2.78
417.8286	2.24	2.14	2.76
417.8858	2.22	2.15	2.76
417.9428	2.24	2.12	2.77
418	2.25	2.09	2.78
418.0572	2.26	2.09	2.79
418.1142	2.23	2.09	2.78
418.1714	2.22	2.1	2.78
418.2286	2.25	2.08	2.76
418.2858	2.31	2.09	2.77
418.3428	2.26	2.09	2.77
418.4	2.27	2.1	2.78
418.4572	2.29	2.08	2.77
418.5142	2.29	2.1	2.79
418.5714	2.3	2.1	2.79
418.6286	2.31	2.07	2.78
418.6858	2.35	2.06	2.77
418.7428	2.38	2.02	2.77
418.8	2.37	2.03	2.77

418.8572	2.33	2.04	2.76
418.9142	2.34	2.04	2.75
418.9714	2.36	2.03	2.76
419.0286	2.36	2.03	2.79
419.0858	2.33	2.03	2.82
419.1428	2.32	2.03	2.86
419.2	2.32	2.03	2.85
419.2572	2.31	2.04	2.86
419.3142	2.3	2.05	2.87
419.3714	2.3	2.05	2.87
419.4286	2.32	2.03	2.86
419.4858	2.31	2.03	2.83
419.5428	2.29	2.05	2.84
419.6	2.29	2.07	2.84
419.6572	2.3	2.07	2.84
419.7142	2.31	2.06	2.85
419.7714	2.33	2.05	2.87
419.8286	2.33	2.07	2.86
419.8858	2.33	2.08	2.85
419.9428	2.33	2.08	2.85
420	2.3	2.11	2.82
420.0572	2.3	2.11	2.82
420.1142	2.3	2.09	2.8
420.1714	2.3	2.07	2.78
420.2286	2.3	2.06	2.76
420.2858	2.27	2.07	2.76
420.3428	2.24	2.08	2.76
420.4	2.23	2.05	2.79
420.4572	2.22	2.05	2.78
420.5142	2.23	2.07	2.79
420.5714	2.24	2.07	2.78
420.6286	2.26	2.07	2.78
420.6858	2.25	2.08	2.78
420.7428	2.27	2.08	2.79
420.8	2.3	2.07	2.8
420.8572	2.32	2.04	2.79
420.9142	2.36	2.04	2.79
420.9714	2.37	2.05	2.78
421.0286	2.39	2.06	2.77
421.0858	2.38	2.07	2.76
421.1428	2.4	2.05	2.78
421.2	2.38	2.05	2.79
421.2572	2.37	2.04	2.78
421.3142	2.36	2.08	2.77
421.3714	2.34	2.08	2.76
421.4286	2.3	2.09	2.75
421.4858	2.29	2.11	2.75

421.5428	2.28	2.14	2.76
421.6	2.26	2.14	2.77
421.6572	2.25	2.1	2.76
421.7142	2.23	2.09	2.75
421.7714	2.24	2.09	2.75
421.8286	2.26	2.08	2.76
421.8858	2.26	2.08	2.78
421.9428	2.23	2.11	2.79
422	2.22	2.13	2.81
422.0572	2.21	2.14	2.8
422.1142	2.21	2.1	2.79
422.1714	2.21	2.1	2.79
422.2286	2.22	2.09	2.79
422.2858	2.25	2.07	2.78
422.3428	2.23	2.09	2.78
422.4	2.2	2.1	2.78
422.4572	2.18	2.09	2.76
422.5142	2.2	2.07	2.74
422.5714	2.23	2.07	2.74
422.6286	2.21	2.05	2.75
422.6858	2.2	2.06	2.77
422.7428	2.2	2.08	2.76
422.8	2.21	2.07	2.75
422.8572	2.21	2.09	2.73
422.9142	2.22	2.09	2.71
422.9714	2.25	2.07	2.69
423.0286	2.24	2.07	2.71
423.0858	2.23	2.06	2.72
423.1428	2.25	2.05	2.71
423.2	2.3	2.05	2.72
423.2572	2.32	2.01	2.74
423.3142	2.32	2	2.75
423.3714	2.29	2.04	2.75
423.4286	2.29	2.02	2.76
423.4858	2.28	2	2.76
423.5428	2.26	2.02	2.75
423.6	2.28	2	2.74
423.6572	2.32	1.99	2.73
423.7142	2.3	2.02	2.73
423.7714	2.27	2.02	2.73
423.8286	2.25	2.06	2.73
423.8858	2.26	2.05	2.75
423.9428	2.27	2.03	2.74
424	2.25	2.02	2.7
424.0572	2.26	2.04	2.73
424.1142	2.26	2.04	2.74
424.1714	2.25	2.05	2.71

424.2286	2.23	2.05	2.7
424.2858	2.22	2.04	2.7
424.3428	2.21	2.03	2.7
424.4	2.23	2.03	2.69
424.4572	2.24	2.01	2.68
424.5142	2.26	2	2.68
424.5714	2.24	2.03	2.69
424.6286	2.22	2	2.71
424.6858	2.24	2	2.71
424.7428	2.24	2	2.72
424.8	2.24	2	2.71
424.8572	2.22	2.01	2.72
424.9142	2.23	2.01	2.74
424.9714	2.24	2.02	2.74
425.0286	2.24	2.06	2.73
425.0858	2.24	2.07	2.75
425.1428	2.25	2.07	2.75
425.2	2.26	2.11	2.74
425.2572	2.26	2.11	2.73
425.3142	2.29	2.09	2.74
425.3714	2.29	2.07	2.74
425.4286	2.29	2.04	2.74
425.4858	2.27	2.02	2.73
425.5428	2.26	2	2.71
425.6	2.27	2	2.69
425.6572	2.26	2	2.68
425.7142	2.25	1.99	2.68
425.7714	2.24	1.98	2.68
425.8286	2.22	1.97	2.65
425.8858	2.2	2.01	2.65
425.9428	2.19	2.02	2.65
426	2.2	2.05	2.64
426.0572	2.2	2.08	2.65
426.1142	2.23	2.08	2.66
426.1714	2.2	2.08	2.65
426.2286	2.18	2.09	2.65
426.2858	2.17	2.1	2.65
426.3428	2.18	2.11	2.64
426.4	2.18	2.1	2.65
426.4572	2.18	2.07	2.67
426.5142	2.17	2.06	2.69
426.5714	2.16	2.06	2.7
426.6286	2.18	2.05	2.69
426.6858	2.17	2.04	2.68
426.7428	2.15	2.05	2.69
426.8	2.16	2.04	2.67
426.8572	2.14	2.04	2.67

426.9142	2.15	2.04	2.67
426.9714	2.13	2.05	2.66
427.0286	2.12	2.07	2.65
427.0858	2.15	2.06	2.65
427.1428	2.16	2.06	2.63
427.2	2.17	2.07	2.64
427.2572	2.18	2.08	2.64
427.3142	2.18	2.08	2.65
427.3714	2.19	2.05	2.63
427.4286	2.27	2.01	2.59
427.4858	2.29	1.98	2.58
427.5428	2.29	1.99	2.58
427.6	2.33	1.98	2.58
427.6572	2.29	2	2.58
427.7142	2.27	2.02	2.6
427.7714	2.25	2.03	2.6
427.8286	2.23	2.02	2.61
427.8858	2.25	2	2.61
427.9428	2.22	2.01	2.64
428	2.17	2.05	2.65
428.0572	2.17	2.05	2.65
428.1142	2.17	2.03	2.66
428.1714	2.16	2.02	2.66
428.2286	2.16	2.02	2.66
428.2858	2.18	1.99	2.66
428.3428	2.19	1.97	2.67
428.4	2.2	1.99	2.69
428.4572	2.19	1.98	2.7
428.5142	2.19	1.99	2.71
428.5714	2.21	1.97	2.69
428.6286	2.2	1.96	2.69
428.6858	2.21	1.95	2.69
428.7428	2.2	1.94	2.69
428.8	2.22	1.96	2.68
428.8572	2.22	1.96	2.68
428.9142	2.23	1.95	2.67
428.9714	2.22	1.95	2.68
429.0286	2.23	1.96	2.67
429.0858	2.24	1.96	2.65
429.1428	2.25	1.98	2.65
429.2	2.26	1.98	2.66
429.2572	2.26	1.99	2.67
429.3142	2.26	1.99	2.67
429.3714	2.27	1.99	2.67
429.4286	2.26	1.98	2.68
429.4858	2.25	2	2.68
429.5428	2.25	2.01	2.67

429.6	2.26	2	2.64
429.6572	2.25	2.02	2.64
429.7142	2.24	2.02	2.63
429.7714	2.23	2.03	2.62
429.8286	2.21	2.04	2.61
429.8858	2.2	2.05	2.6
429.9428	2.19	2.04	2.62
430	2.17	2.04	2.63
430.0572	2.15	2.02	2.64
430.1142	2.16	2.01	2.63
430.1714	2.17	1.98	2.6
430.2286	2.17	1.95	2.59
430.2858	2.15	1.93	2.58
430.3428	2.16	1.93	2.58
430.4	2.15	1.93	2.58
430.4572	2.17	1.92	2.58
430.5142	2.19	1.91	2.57
430.5714	2.17	1.92	2.56
430.6286	2.17	1.94	2.56
430.6858	2.14	1.94	2.57
430.7428	2.16	1.96	2.58
430.8	2.15	1.97	2.58
430.8572	2.16	1.99	2.56
430.9142	2.15	2	2.56
430.9714	2.16	2	2.57
431.0286	2.17	1.99	2.58
431.0858	2.16	1.99	2.58
431.1428	2.19	1.98	2.59
431.2	2.21	1.96	2.58
431.2572	2.25	1.95	2.58
431.3142	2.23	1.97	2.58
431.3714	2.25	1.95	2.59
431.4286	2.25	1.95	2.6
431.4858	2.26	1.94	2.61
431.5428	2.24	1.92	2.6
431.6	2.23	1.93	2.6
431.6572	2.2	1.94	2.61
431.7142	2.19	1.93	2.61
431.7714	2.19	1.92	2.61
431.8286	2.16	1.93	2.62
431.8858	2.17	1.92	2.63
431.9428	2.14	1.93	2.64
432	2.17	1.89	2.64
432.0572	2.16	1.91	2.63
432.1142	2.15	1.96	2.63
432.1714	2.13	1.96	2.62
432.2286	2.15	1.95	2.62

432.2858	2.15	1.97	2.61
432.3428	2.12	1.99	2.63
432.4	2.11	2.02	2.64
432.4572	2.1	2.03	2.63
432.5142	2.13	2.02	2.61
432.5714	2.09	2.04	2.59
432.6286	2.1	2.01	2.59
432.6858	2.12	1.97	2.59
432.7428	2.14	1.96	2.56
432.8	2.12	1.97	2.55
432.8572	2.11	1.99	2.54
432.9142	2.14	1.96	2.56
432.9714	2.18	1.95	2.57
433.0286	2.18	1.91	2.6
433.0858	2.19	1.92	2.61
433.1428	2.2	1.92	2.59
433.2	2.21	1.94	2.59
433.2572	2.21	1.96	2.57
433.3142	2.18	1.97	2.58
433.3714	2.2	1.96	2.59
433.4286	2.2	1.94	2.6
433.4858	2.18	1.98	2.63
433.5428	2.18	1.95	2.66
433.6	2.2	1.98	2.66
433.6572	2.14	1.98	2.67
433.7142	2.15	1.97	2.68
433.7714	2.14	1.98	2.69
433.8286	2.15	1.96	2.68
433.8858	2.16	1.98	2.67
433.9428	2.18	1.98	2.67
434	2.2	1.97	2.66
434.0572	2.19	1.94	2.66
434.1142	2.18	1.95	2.65
434.1714	2.17	1.95	2.64
434.2286	2.19	1.94	2.64
434.2858	2.18	1.95	2.62
434.3428	2.19	1.93	2.6
434.4	2.2	1.93	2.57
434.4572	2.21	1.9	2.53
434.5142	2.18	1.91	2.53
434.5714	2.17	1.89	2.52
434.6286	2.19	1.89	2.52
434.6858	2.19	1.9	2.52
434.7428	2.19	1.91	2.52
434.8	2.19	1.88	2.52
434.8572	2.17	1.92	2.51
434.9142	2.19	1.91	2.49

434.9714	2.19	1.92	2.53
435.0286	2.18	1.91	2.53
435.0858	2.18	1.93	2.54
435.1428	2.2	1.92	2.54
435.2	2.21	1.91	2.55
435.2572	2.2	1.93	2.57
435.3142	2.19	1.93	2.57
435.3714	2.18	1.94	2.56
435.4286	2.2	1.93	2.56
435.4858	2.17	1.94	2.56
435.5428	2.17	1.96	2.56
435.6	2.16	1.98	2.53
435.6572	2.16	1.98	2.53
435.7142	2.14	2	2.54
435.7714	2.12	2.03	2.53
435.8286	2.12	2.04	2.53
435.8858	2.14	2.01	2.53
435.9428	2.14	2	2.51
436	2.12	2	2.53
436.0572	2.12	1.99	2.54
436.1142	2.12	1.95	2.52
436.1714	2.12	1.93	2.52
436.2286	2.09	1.92	2.53
436.2858	2.07	1.93	2.53
436.3428	2.07	1.93	2.55
436.4	2.1	1.89	2.54
436.4572	2.12	1.87	2.54
436.5142	2.16	1.86	2.56
436.5714	2.14	1.86	2.55
436.6286	2.16	1.82	2.56
436.6858	2.16	1.84	2.59
436.7428	2.16	1.83	2.62
436.8	2.21	1.84	2.61
436.8572	2.23	1.83	2.61
436.9142	2.25	1.81	2.61
436.9714	2.21	1.84	2.6
437.0286	2.17	1.88	2.6
437.0858	2.16	1.89	2.58
437.1428	2.17	1.88	2.58
437.2	2.17	1.9	2.57
437.2572	2.15	1.9	2.57
437.3142	2.16	1.9	2.58
437.3714	2.13	1.91	2.57
437.4286	2.13	1.91	2.56
437.4858	2.13	1.93	2.56
437.5428	2.18	1.91	2.55
437.6	2.21	1.92	2.52

437.6572	2.21	1.93	2.51
437.7142	2.19	1.94	2.5
437.7714	2.18	1.96	2.49
437.8286	2.16	1.99	2.5
437.8858	2.16	2	2.53
437.9428	2.17	1.98	2.54
438	2.16	1.96	2.55
438.0572	2.14	1.95	2.54
438.1142	2.1	1.99	2.55
438.1714	2.08	1.97	2.55
438.2286	2.07	1.96	2.54
438.2858	2.05	1.97	2.55
438.3428	2.05	1.99	2.56
438.4	2.07	1.96	2.52
438.4572	2.08	1.95	2.52
438.5142	2.08	1.97	2.52
438.5714	2.07	2	2.51
438.6286	2.11	1.98	2.52
438.6858	2.12	1.96	2.52
438.7428	2.12	1.95	2.5
438.8	2.14	1.94	2.48
438.8572	2.15	1.94	2.47
438.9142	2.18	1.9	2.49
438.9714	2.17	1.89	2.49
439.0286	2.17	1.87	2.48
439.0858	2.16	1.88	2.48
439.1428	2.16	1.89	2.47
439.2	2.15	1.9	2.47
439.2572	2.15	1.91	2.49
439.3142	2.15	1.95	2.51
439.3714	2.14	1.96	2.5
439.4286	2.13	1.96	2.52
439.4858	2.14	1.96	2.53
439.5428	2.13	1.98	2.53
439.6	2.12	2.01	2.5
439.6572	2.16	2	2.51
439.7142	2.14	2	2.52
439.7714	2.1	2.02	2.52
439.8286	2.09	2.02	2.49
439.8858	2.1	2.01	2.49
439.9428	2.12	2.01	2.5
440	2.11	1.98	2.51
440.0572	2.11	1.99	2.53
440.1142	2.11	1.99	2.52
440.1714	2.13	1.94	2.51
440.2286	2.11	1.95	2.54
440.2858	2.14	1.92	2.54

440.3428	2.17	1.9	2.52
440.4	2.17	1.91	2.51
440.4572	2.13	1.91	2.51
440.5142	2.12	1.9	2.52
440.5714	2.13	1.92	2.5
440.6286	2.13	1.94	2.5
440.6858	2.15	1.91	2.5
440.7428	2.14	1.92	2.52
440.8	2.13	1.91	2.52
440.8572	2.1	1.91	2.5
440.9142	2.08	1.92	2.48
440.9714	2.09	1.93	2.47
441.0286	2.11	1.91	2.47
441.0858	2.12	1.93	2.44
441.1428	2.14	1.89	2.41
441.2	2.14	1.88	2.41
441.2572	2.13	1.89	2.44
441.3142	2.11	1.91	2.44
441.3714	2.08	1.92	2.44
441.4286	2.11	1.9	2.46
441.4858	2.13	1.89	2.48
441.5428	2.13	1.86	2.49
441.6	2.13	1.85	2.49
441.6572	2.11	1.87	2.5
441.7142	2.07	1.91	2.5
441.7714	2.04	1.91	2.5
441.8286	2.02	1.93	2.51
441.8858	2.04	1.93	2.48
441.9428	2.08	1.91	2.49
442	2.07	1.9	2.49
442.0572	2.04	1.93	2.49
442.1142	2.05	1.92	2.5
442.1714	2.05	1.92	2.5
442.2286	2.07	1.95	2.5
442.2858	2.1	1.93	2.49
442.3428	2.1	1.9	2.49
442.4	2.11	1.91	2.47
442.4572	2.13	1.9	2.48
442.5142	2.16	1.89	2.48
442.5714	2.14	1.9	2.48
442.6286	2.17	1.89	2.44
442.6858	2.16	1.92	2.45
442.7428	2.16	1.92	2.45
442.8	2.16	1.91	2.47
442.8572	2.14	1.93	2.49
442.9142	2.11	1.93	2.51
442.9714	2.11	1.95	2.51

443.0286	2.13	1.92	2.53
443.0858	2.12	1.92	2.54
443.1428	2.08	1.93	2.54
443.2	2.07	1.94	2.55
443.2572	2.05	1.96	2.56
443.3142	2.06	1.96	2.55
443.3714	2.07	1.93	2.55
443.4286	2.07	1.95	2.54
443.4858	2.08	1.95	2.51
443.5428	2.09	1.95	2.5
443.6	2.08	1.96	2.5
443.6572	2.07	1.97	2.48
443.7142	2.07	1.99	2.47
443.7714	2.1	1.97	2.47
443.8286	2.1	1.97	2.46
443.8858	2.07	1.97	2.45
443.9428	2.05	1.99	2.45
444	2.06	1.97	2.43
444.0572	2.05	1.95	2.44
444.1142	2.04	1.94	2.42
444.1714	2.01	1.97	2.43
444.2286	2	1.97	2.43
444.2858	2.02	1.95	2.41
444.3428	2.03	1.92	2.41
444.4	2	1.94	2.44
444.4572	2.03	1.92	2.47
444.5142	2.06	1.91	2.48
444.5714	2.05	1.92	2.5
444.6286	2.03	1.94	2.48
444.6858	2.02	1.94	2.47
444.7428	2.06	1.92	2.49
444.8	2.1	1.89	2.5
444.8572	2.1	1.87	2.49
444.9142	2.1	1.89	2.49
444.9714	2.1	1.88	2.47
445.0286	2.09	1.89	2.47
445.0858	2.08	1.89	2.48
445.1428	2.08	1.88	2.5
445.2	2.1	1.86	2.49
445.2572	2.1	1.87	2.48
445.3142	2.07	1.88	2.48
445.3714	2.04	1.89	2.47
445.4286	2.03	1.91	2.45
445.4858	2	1.94	2.43
445.5428	2	1.94	2.44
445.6	2.01	1.94	2.43
445.6572	2.02	1.95	2.45

445.7142	2.04	1.95	2.45
445.7714	2	1.97	2.44
445.8286	1.99	1.98	2.44
445.8858	2.02	1.97	2.43
445.9428	2.03	1.98	2.44
446	2.06	1.99	2.42
446.0572	2.06	1.97	2.43
446.1142	2.07	1.93	2.45
446.1714	2.09	1.93	2.44
446.2286	2.08	1.91	2.41
446.2858	2.05	1.89	2.38
446.3428	2.06	1.88	2.39
446.4	2.1	1.9	2.4
446.4572	2.09	1.89	2.4
446.5142	2.07	1.9	2.38
446.5714	2.08	1.85	2.37
446.6286	2.07	1.85	2.36
446.6858	2.05	1.86	2.36
446.7428	2.05	1.88	2.36
446.8	2.05	1.89	2.37
446.8572	2.06	1.91	2.35
446.9142	2.12	1.88	2.35
446.9714	2.11	1.83	2.32
447.0286	2.11	1.86	2.31
447.0858	2.11	1.83	2.33
447.1428	2.08	1.88	2.34
447.2	2.09	1.9	2.34
447.2572	2.11	1.9	2.35
447.3142	2.09	1.9	2.36
447.3714	2.07	1.93	2.34
447.4286	2.09	1.92	2.36
447.4858	2.07	1.93	2.35
447.5428	2.03	1.96	2.37
447.6	2.04	1.94	2.38
447.6572	2.05	1.95	2.37
447.7142	2.07	1.92	2.36
447.7714	2.05	1.9	2.39
447.8286	2.03	1.92	2.4
447.8858	2.06	1.91	2.42
447.9428	2.07	1.89	2.42
448	2.03	1.91	2.4
448.0572	2.02	1.94	2.4
448.1142	2.03	1.95	2.42
448.1714	2.01	1.96	2.43
448.2286	2.01	1.95	2.41
448.2858	1.99	1.97	2.41
448.3428	2	1.96	2.4

448.4	2.05	1.92	2.41
448.4572	2	1.94	2.37
448.5142	1.96	1.94	2.36
448.5714	1.97	1.94	2.37
448.6286	1.98	1.92	2.38
448.6858	1.98	1.91	2.37
448.7428	1.98	1.89	2.37
448.8	2	1.87	2.38
448.8572	2.01	1.87	2.39
448.9142	2.04	1.88	2.4
448.9714	2.01	1.91	2.4
449.0286	2.03	1.88	2.38
449.0858	2.05	1.87	2.37
449.1428	2.06	1.84	2.38
449.2	2.03	1.85	2.39
449.2572	2.03	1.86	2.38
449.3142	2.02	1.9	2.38
449.3714	2.02	1.91	2.39
449.4286	2.02	1.89	2.42
449.4858	1.99	1.91	2.41
449.5428	2	1.91	2.39
449.6	2.01	1.91	2.38
449.6572	2.04	1.91	2.38
449.7142	2.05	1.93	2.37
449.7714	2.07	1.94	2.37
449.8286	2.07	1.93	2.37
449.8858	2.08	1.89	2.38
449.9428	2.08	1.88	2.36
450	2.1	1.89	2.35
450.0572	2.11	1.89	2.35
450.1142	2.09	1.88	2.35
450.1714	2.07	1.88	2.34
450.2286	2.03	1.89	2.35
450.2858	2.01	1.91	2.37
450.3428	2.02	1.9	2.36
450.4	2.04	1.89	2.37
450.4572	2.07	1.87	2.38
450.5142	2.06	1.89	2.37
450.5714	2.03	1.89	2.36
450.6286	2.07	1.85	2.37
450.6858	2.06	1.89	2.36
450.7428	2.04	1.91	2.37
450.8	2.05	1.93	2.38
450.8572	2.07	1.9	2.41
450.9142	2.05	1.89	2.41
450.9714	2.07	1.89	2.43
451.0286	2.03	1.91	2.43

451.0858	2.05	1.89	2.43
451.1428	2.07	1.88	2.41
451.2	2.04	1.9	2.39
451.2572	2.04	1.88	2.38
451.3142	2.05	1.87	2.38
451.3714	2.05	1.84	2.37
451.4286	2.07	1.84	2.38
451.4858	2.06	1.84	2.37
451.5428	2.03	1.88	2.39
451.6	2.03	1.9	2.38
451.6572	2	1.91	2.37
451.7142	1.98	1.92	2.36
451.7714	2.01	1.9	2.33
451.8286	2.02	1.93	2.36
451.8858	2.04	1.91	2.35
451.9428	2.06	1.91	2.35
452	2.03	1.92	2.36
452.0572	2.06	1.91	2.35
452.1142	2.07	1.87	2.37
452.1714	2.09	1.84	2.38
452.2286	2.09	1.83	2.37
452.2858	2.1	1.82	2.37
452.3428	2.08	1.83	2.36
452.4	2.07	1.83	2.35
452.4572	2.04	1.85	2.34
452.5142	2.05	1.85	2.35
452.5714	2.07	1.82	2.34
452.6286	2.07	1.83	2.34
452.6858	2.07	1.82	2.36
452.7428	2.02	1.86	2.34
452.8	2.04	1.88	2.35
452.8572	2.03	1.88	2.37
452.9142	2.01	1.89	2.38
452.9714	2.03	1.88	2.4
453.0286	2.01	1.9	2.38
453.0858	2	1.9	2.39
453.1428	1.95	1.92	2.41
453.2	1.94	1.94	2.42
453.2572	1.94	1.95	2.43
453.3142	1.93	1.92	2.42
453.3714	1.91	1.93	2.42
453.4286	1.89	1.94	2.42
453.4858	1.91	1.93	2.41
453.5428	1.89	1.93	2.42
453.6	1.91	1.89	2.41
453.6572	1.92	1.89	2.41
453.7142	1.96	1.88	2.4

453.7714	1.99	1.86	2.38
453.8286	2.02	1.85	2.38
453.8858	2.01	1.85	2.37
453.9428	2.03	1.84	2.37
454	2.05	1.82	2.37
454.0572	2.05	1.82	2.38
454.1142	2.03	1.82	2.36
454.1714	2.07	1.81	2.35
454.2286	2.05	1.81	2.36
454.2858	2.04	1.82	2.36
454.3428	2	1.83	2.37
454.4	1.95	1.88	2.36
454.4572	1.97	1.89	2.36
454.5142	1.98	1.91	2.36
454.5714	1.99	1.93	2.35
454.6286	1.95	1.95	2.36
454.6858	1.96	1.94	2.35
454.7428	1.96	1.94	2.33
454.8	1.96	1.91	2.33
454.8572	1.94	1.95	2.32
454.9142	1.95	1.92	2.3
454.9714	1.98	1.9	2.29
455.0286	1.97	1.92	2.32
455.0858	1.96	1.95	2.3
455.1428	1.93	1.93	2.3
455.2	1.97	1.91	2.3
455.2572	2.02	1.89	2.3
455.3142	2.02	1.88	2.31
455.3714	2.03	1.91	2.3
455.4286	2.05	1.86	2.3
455.4858	2.03	1.86	2.31
455.5428	1.99	1.88	2.32
455.6	1.98	1.87	2.34
455.6572	2.01	1.81	2.35
455.7142	2.01	1.81	2.34
455.7714	2	1.81	2.34
455.8286	1.99	1.83	2.33
455.8858	1.96	1.84	2.32
455.9428	1.95	1.85	2.31
456	1.94	1.89	2.32
456.0572	1.96	1.9	2.32
456.1142	1.97	1.91	2.32
456.1714	2	1.87	2.31
456.2286	1.98	1.87	2.33
456.2858	2	1.86	2.33
456.3428	2	1.87	2.34
456.4	1.99	1.88	2.32

456.4572	2	1.87	2.32
456.5142	2.02	1.83	2.31
456.5714	2.04	1.78	2.32
456.6286	2.02	1.79	2.33
456.6858	2.01	1.8	2.33
456.7428	1.99	1.83	2.34
456.8	1.98	1.85	2.35
456.8572	1.96	1.88	2.36
456.9142	1.95	1.87	2.37
456.9714	1.96	1.86	2.39
457.0286	1.94	1.87	2.37
457.0858	1.94	1.89	2.37
457.1428	1.91	1.93	2.36
457.2	1.91	1.9	2.34
457.2572	1.9	1.89	2.34
457.3142	1.9	1.89	2.33
457.3714	1.91	1.87	2.33
457.4286	1.92	1.86	2.32
457.4858	1.92	1.86	2.3
457.5428	1.92	1.85	2.3
457.6	1.92	1.86	2.29
457.6572	1.91	1.85	2.29
457.7142	1.93	1.82	2.28
457.7714	1.93	1.84	2.3
457.8286	1.97	1.85	2.27
457.8858	1.96	1.84	2.25
457.9428	1.96	1.83	2.26
458	1.97	1.85	2.24
458.0572	1.95	1.84	2.24
458.1142	1.92	1.87	2.24
458.1714	1.91	1.88	2.24
458.2286	1.91	1.89	2.24
458.2858	1.87	1.94	2.26
458.3428	1.87	1.91	2.28
458.4	1.84	1.89	2.29
458.4572	1.85	1.9	2.28
458.5142	1.83	1.91	2.3
458.5714	1.82	1.91	2.31
458.6286	1.84	1.89	2.3
458.6858	1.84	1.92	2.3
458.7428	1.83	1.93	2.32
458.8	1.83	1.9	2.32
458.8572	1.85	1.87	2.32
458.9142	1.86	1.9	2.34
458.9714	1.82	1.93	2.36
459.0286	1.83	1.93	2.37
459.0858	1.83	1.91	2.38

459.1428	1.83	1.91	2.37
459.2	1.84	1.9	2.35
459.2572	1.86	1.87	2.33
459.3142	1.87	1.85	2.32
459.3714	1.86	1.86	2.33
459.4286	1.85	1.88	2.32
459.4858	1.86	1.87	2.3
459.5428	1.91	1.81	2.28
459.6	1.93	1.77	2.27
459.6572	1.92	1.8	2.26
459.7142	1.93	1.79	2.28
459.7714	1.9	1.8	2.27
459.8286	1.9	1.81	2.25
459.8858	1.88	1.83	2.23
459.9428	1.88	1.82	2.23
460	1.89	1.81	2.24
460.0572	1.89	1.8	2.25
460.1142	1.84	1.82	2.24
460.1714	1.8	1.86	2.25
460.2286	1.82	1.86	2.23
460.2858	1.83	1.84	2.21
460.3428	1.87	1.83	2.21
460.4	1.88	1.81	2.23
460.4572	1.89	1.83	2.22
460.5142	1.91	1.83	2.23
460.5714	1.92	1.84	2.26
460.6286	1.93	1.85	2.25
460.6858	1.95	1.85	2.26
460.7428	1.97	1.83	2.27
460.8	1.98	1.82	2.29
460.8572	1.97	1.85	2.26
460.9142	1.95	1.84	2.24
460.9714	1.96	1.85	2.22
461.0286	1.96	1.85	2.21
461.0858	1.94	1.84	2.19
461.1428	1.91	1.85	2.22
461.2	1.9	1.87	2.25
461.2572	1.9	1.87	2.27
461.3142	1.92	1.88	2.3
461.3714	1.95	1.86	2.29
461.4286	1.93	1.84	2.3
461.4858	1.92	1.87	2.27
461.5428	1.89	1.86	2.25
461.6	1.91	1.83	2.24
461.6572	1.92	1.82	2.23
461.7142	1.95	1.81	2.23
461.7714	1.98	1.8	2.26

461.8286	1.96	1.81	2.26
461.8858	1.91	1.84	2.28
461.9428	1.86	1.86	2.3
462	1.86	1.86	2.32
462.0572	1.88	1.86	2.33
462.1142	1.89	1.87	2.32
462.1714	1.87	1.87	2.31
462.2286	1.84	1.89	2.3
462.2858	1.86	1.88	2.32
462.3428	1.85	1.88	2.32
462.4	1.88	1.87	2.32
462.4572	1.91	1.83	2.32
462.5142	1.91	1.85	2.33
462.5714	1.91	1.85	2.33
462.6286	1.91	1.85	2.32
462.6858	1.89	1.85	2.31
462.7428	1.88	1.85	2.32
462.8	1.89	1.87	2.3
462.8572	1.88	1.86	2.3
462.9142	1.86	1.86	2.28
462.9714	1.84	1.85	2.26
463.0286	1.83	1.84	2.25
463.0858	1.84	1.85	2.23
463.1428	1.88	1.82	2.2
463.2	1.88	1.83	2.2
463.2572	1.9	1.82	2.19
463.3142	1.92	1.82	2.2
463.3714	1.92	1.82	2.22
463.4286	1.9	1.8	2.22
463.4858	1.89	1.79	2.22
463.5428	1.89	1.79	2.23
463.6	1.85	1.82	2.23
463.6572	1.85	1.81	2.22
463.7142	1.82	1.84	2.22
463.7714	1.8	1.85	2.23
463.8286	1.8	1.85	2.24
463.8858	1.77	1.85	2.25
463.9428	1.78	1.85	2.24
464	1.79	1.87	2.23
464.0572	1.79	1.87	2.25
464.1142	1.8	1.87	2.24
464.1714	1.83	1.84	2.23
464.2286	1.86	1.83	2.24
464.2858	1.86	1.83	2.23
464.3428	1.86	1.83	2.24
464.4	1.86	1.84	2.22
464.4572	1.88	1.85	2.2

464.5142	1.9	1.83	2.18
464.5714	1.87	1.84	2.18
464.6286	1.86	1.88	2.17
464.6858	1.85	1.88	2.17
464.7428	1.86	1.89	2.15
464.8	1.85	1.9	2.14
464.8572	1.87	1.9	2.14
464.9142	1.86	1.91	2.14
464.9714	1.86	1.9	2.13
465.0286	1.86	1.88	2.14
465.0858	1.85	1.88	2.15
465.1428	1.86	1.85	2.14
465.2	1.88	1.84	2.13
465.2572	1.9	1.82	2.1
465.3142	1.92	1.83	2.13
465.3714	1.9	1.84	2.14
465.4286	1.89	1.82	2.14
465.4858	1.91	1.79	2.13
465.5428	1.91	1.79	2.13
465.6	1.93	1.78	2.11
465.6572	1.94	1.77	2.11
465.7142	1.96	1.76	2.14
465.7714	1.94	1.77	2.17
465.8286	1.93	1.79	2.19
465.8858	1.94	1.8	2.2
465.9428	1.94	1.8	2.19
466	1.93	1.8	2.2
466.0572	1.92	1.78	2.21
466.1142	1.92	1.74	2.22
466.1714	1.88	1.75	2.24
466.2286	1.86	1.76	2.22
466.2858	1.82	1.82	2.23
466.3428	1.83	1.83	2.24
466.4	1.85	1.8	2.22
466.4572	1.83	1.79	2.22
466.5142	1.82	1.77	2.23
466.5714	1.82	1.78	2.23
466.6286	1.79	1.82	2.21
466.6858	1.82	1.83	2.21
466.7428	1.85	1.84	2.18
466.8	1.85	1.85	2.19
466.8572	1.9	1.8	2.2
466.9142	1.9	1.79	2.2
466.9714	1.89	1.8	2.2
467.0286	1.88	1.78	2.2
467.0858	1.89	1.79	2.2
467.1428	1.91	1.79	2.19

467.2	1.94	1.79	2.19
467.2572	1.95	1.78	2.2
467.3142	1.92	1.8	2.24
467.3714	1.95	1.78	2.25
467.4286	1.92	1.8	2.25
467.4858	1.92	1.79	2.26
467.5428	1.94	1.8	2.26
467.6	1.93	1.81	2.24
467.6572	1.93	1.81	2.24
467.7142	1.94	1.82	2.24
467.7714	1.93	1.79	2.22
467.8286	1.89	1.82	2.21
467.8858	1.89	1.81	2.21
467.9428	1.88	1.8	2.22
468	1.88	1.78	2.22
468.0572	1.89	1.79	2.21
468.1142	1.89	1.77	2.22
468.1714	1.9	1.8	2.23
468.2286	1.91	1.79	2.21
468.2858	1.88	1.79	2.19
468.3428	1.88	1.83	2.18
468.4	1.9	1.81	2.19
468.4572	1.9	1.83	2.19
468.5142	1.87	1.85	2.17
468.5714	1.87	1.87	2.17
468.6286	1.86	1.88	2.18
468.6858	1.88	1.87	2.16
468.7428	1.85	1.88	2.16
468.8	1.86	1.86	2.15
468.8572	1.88	1.84	2.14
468.9142	1.89	1.82	2.17
468.9714	1.87	1.84	2.18
469.0286	1.85	1.83	2.17
469.0858	1.88	1.82	2.15
469.1428	1.88	1.82	2.15
469.2	1.88	1.85	2.18
469.2572	1.92	1.79	2.21
469.3142	1.89	1.81	2.23
469.3714	1.85	1.81	2.24
469.4286	1.8	1.81	2.25
469.4858	1.79	1.84	2.29
469.5428	1.78	1.87	2.29
469.6	1.78	1.87	2.29
469.6572	1.8	1.85	2.3
469.7142	1.8	1.85	2.31
469.7714	1.8	1.81	2.32
469.8286	1.77	1.84	2.3

469.8858	1.77	1.84	2.28
469.9428	1.77	1.85	2.29
470	1.82	1.85	2.29
470.0572	1.81	1.85	2.29
470.1142	1.78	1.85	2.26
470.1714	1.79	1.83	2.24
470.2286	1.76	1.84	2.23
470.2858	1.79	1.83	2.23
470.3428	1.78	1.82	2.2
470.4	1.77	1.82	2.19
470.4572	1.79	1.82	2.21
470.5142	1.81	1.81	2.22
470.5714	1.83	1.8	2.2
470.6286	1.86	1.77	2.19
470.6858	1.9	1.76	2.21
470.7428	1.89	1.77	2.2
470.8	1.92	1.78	2.21
470.8572	1.91	1.78	2.21
470.9142	1.88	1.81	2.23
470.9714	1.87	1.82	2.22
471.0286	1.89	1.81	2.23
471.0858	1.89	1.82	2.24
471.1428	1.89	1.83	2.24
471.2	1.85	1.87	2.24
471.2572	1.84	1.85	2.26
471.3142	1.86	1.84	2.26
471.3714	1.85	1.83	2.24
471.4286	1.85	1.85	2.23
471.4858	1.85	1.82	2.26
471.5428	1.87	1.82	2.25
471.6	1.84	1.85	2.26
471.6572	1.82	1.87	2.27
471.7142	1.79	1.86	2.24
471.7714	1.8	1.85	2.23
471.8286	1.75	1.88	2.2
471.8858	1.74	1.87	2.22
471.9428	1.74	1.88	2.24
472	1.74	1.85	2.22
472.0572	1.74	1.86	2.23
472.1142	1.74	1.85	2.23
472.1714	1.76	1.83	2.23
472.2286	1.76	1.82	2.25
472.2858	1.77	1.81	2.26
472.3428	1.77	1.79	2.24
472.4	1.82	1.77	2.22
472.4572	1.83	1.75	2.22
472.5142	1.82	1.77	2.22

472.5714	1.8	1.78	2.2
472.6286	1.79	1.78	2.23
472.6858	1.81	1.77	2.24
472.7428	1.79	1.77	2.23
472.8	1.81	1.74	2.18
472.8572	1.83	1.77	2.18
472.9142	1.84	1.76	2.19
472.9714	1.83	1.75	2.19
473.0286	1.81	1.77	2.19
473.0858	1.79	1.77	2.19
473.1428	1.8	1.75	2.17
473.2	1.82	1.74	2.17
473.2572	1.83	1.74	2.17
473.3142	1.85	1.74	2.17
473.3714	1.83	1.74	2.15
473.4286	1.81	1.72	2.16
473.4858	1.82	1.7	2.16
473.5428	1.79	1.71	2.16
473.6	1.8	1.74	2.17
473.6572	1.85	1.7	2.19
473.7142	1.85	1.71	2.22
473.7714	1.87	1.71	2.21
473.8286	1.85	1.74	2.21
473.8858	1.83	1.73	2.22
473.9428	1.83	1.74	2.21
474	1.83	1.73	2.2
474.0572	1.82	1.74	2.2
474.1142	1.82	1.75	2.19
474.1714	1.86	1.72	2.19
474.2286	1.86	1.74	2.21
474.2858	1.85	1.74	2.23
474.3428	1.83	1.75	2.24
474.4	1.84	1.73	2.25
474.4572	1.82	1.73	2.24
474.5142	1.87	1.73	2.24
474.5714	1.89	1.74	2.19
474.6286	1.89	1.75	2.2
474.6858	1.92	1.71	2.21
474.7428	1.91	1.72	2.19
474.8	1.88	1.73	2.18
474.8572	1.87	1.73	2.2
474.9142	1.86	1.73	2.19
474.9714	1.86	1.75	2.2
475.0286	1.9	1.73	2.19
475.0858	1.86	1.75	2.18
475.1428	1.86	1.73	2.16
475.2	1.84	1.71	2.16

475.2572	1.84	1.7	2.14
475.3142	1.85	1.71	2.14
475.3714	1.86	1.73	2.14
475.4286	1.86	1.73	2.15
475.4858	1.89	1.72	2.19
475.5428	1.9	1.7	2.2
475.6	1.9	1.71	2.19
475.6572	1.92	1.68	2.2
475.7142	1.93	1.7	2.19
475.7714	1.92	1.73	2.16
475.8286	1.93	1.76	2.15
475.8858	1.95	1.73	2.15
475.9428	1.96	1.69	2.19
476	1.94	1.71	2.2
476.0572	1.92	1.72	2.2
476.1142	1.89	1.74	2.19
476.1714	1.89	1.73	2.18
476.2286	1.86	1.76	2.15
476.2858	1.83	1.78	2.15
476.3428	1.83	1.76	2.14
476.4	1.83	1.74	2.12
476.4572	1.8	1.74	2.11
476.5142	1.78	1.75	2.12
476.5714	1.8	1.73	2.11
476.6286	1.8	1.76	2.11
476.6858	1.8	1.75	2.14
476.7428	1.79	1.77	2.16
476.8	1.8	1.74	2.18
476.8572	1.83	1.73	2.15
476.9142	1.84	1.73	2.15
476.9714	1.83	1.75	2.14
477.0286	1.83	1.76	2.15
477.0858	1.82	1.76	2.16
477.1428	1.81	1.78	2.17
477.2	1.83	1.74	2.17
477.2572	1.86	1.74	2.16
477.3142	1.88	1.74	2.18
477.3714	1.85	1.78	2.18
477.4286	1.83	1.77	2.16
477.4858	1.83	1.76	2.16
477.5428	1.83	1.75	2.17
477.6	1.83	1.75	2.16
477.6572	1.85	1.73	2.13
477.7142	1.88	1.7	2.1
477.7714	1.83	1.71	2.11
477.8286	1.8	1.73	2.13
477.8858	1.77	1.74	2.12

477.9428	1.78	1.73	2.12
478	1.76	1.77	2.14
478.0572	1.76	1.78	2.15
478.1142	1.77	1.76	2.16
478.1714	1.74	1.77	2.16
478.2286	1.72	1.8	2.15
478.2858	1.7	1.83	2.16
478.3428	1.69	1.84	2.17
478.4	1.71	1.82	2.19
478.4572	1.72	1.8	2.19
478.5142	1.74	1.79	2.2
478.5714	1.77	1.78	2.22
478.6286	1.75	1.77	2.23
478.6858	1.75	1.79	2.22
478.7428	1.74	1.79	2.2
478.8	1.73	1.81	2.19
478.8572	1.71	1.8	2.18
478.9142	1.74	1.79	2.15
478.9714	1.75	1.79	2.13
479.0286	1.72	1.8	2.13
479.0858	1.72	1.78	2.12
479.1428	1.73	1.76	2.11
479.2	1.71	1.76	2.1
479.2572	1.71	1.76	2.09
479.3142	1.73	1.75	2.07
479.3714	1.77	1.74	2.07
479.4286	1.78	1.74	2.05
479.4858	1.75	1.76	2.05
479.5428	1.75	1.75	2.06
479.6	1.77	1.72	2.05
479.6572	1.76	1.75	2.08
479.7142	1.73	1.75	2.08
479.7714	1.74	1.8	2.1
479.8286	1.71	1.82	2.09
479.8858	1.72	1.82	2.1
479.9428	1.7	1.81	2.07
480	1.68	1.81	2.08
480.0572	1.69	1.8	2.08
480.1142	1.71	1.82	2.09
480.1714	1.7	1.84	2.09
480.2286	1.7	1.83	2.11
480.2858	1.68	1.83	2.11
480.3428	1.7	1.79	2.11
480.4	1.72	1.76	2.11
480.4572	1.71	1.77	2.12
480.5142	1.72	1.78	2.11
480.5714	1.73	1.8	2.11

480.6286	1.73	1.81	2.1
480.6858	1.72	1.78	2.1
480.7428	1.74	1.75	2.1
480.8	1.74	1.74	2.09
480.8572	1.76	1.72	2.12
480.9142	1.75	1.74	2.11
480.9714	1.75	1.75	2.1
481.0286	1.74	1.76	2.1
481.0858	1.73	1.76	2.1
481.1428	1.72	1.74	2.11
481.2	1.73	1.76	2.11
481.2572	1.7	1.78	2.1
481.3142	1.67	1.82	2.1
481.3714	1.66	1.83	2.11
481.4286	1.66	1.84	2.11
481.4858	1.68	1.82	2.1
481.5428	1.66	1.83	2.11
481.6	1.65	1.84	2.12
481.6572	1.67	1.83	2.15
481.7142	1.69	1.83	2.15
481.7714	1.68	1.82	2.15
481.8286	1.67	1.81	2.14
481.8858	1.71	1.8	2.16
481.9428	1.72	1.78	2.17
482	1.72	1.81	2.16
482.0572	1.71	1.81	2.14
482.1142	1.74	1.79	2.14
482.1714	1.74	1.75	2.17
482.2286	1.75	1.74	2.16
482.2858	1.75	1.74	2.15
482.3428	1.78	1.72	2.16
482.4	1.79	1.72	2.15
482.4572	1.77	1.71	2.16
482.5142	1.77	1.71	2.18
482.5714	1.76	1.71	2.17
482.6286	1.75	1.72	2.16
482.6858	1.74	1.72	2.15
482.7428	1.73	1.74	2.15
482.8	1.74	1.74	2.14
482.8572	1.72	1.76	2.15
482.9142	1.73	1.74	2.15
482.9714	1.72	1.74	2.17
483.0286	1.71	1.75	2.14
483.0858	1.7	1.78	2.11
483.1428	1.72	1.74	2.12
483.2	1.74	1.72	2.11
483.2572	1.74	1.72	2.11

483.3142	1.73	1.73	2.13
483.3714	1.71	1.73	2.13
483.4286	1.71	1.72	2.11
483.4858	1.69	1.74	2.11
483.5428	1.68	1.75	2.12
483.6	1.69	1.73	2.13
483.6572	1.69	1.73	2.11
483.7142	1.68	1.74	2.11
483.7714	1.65	1.77	2.08
483.8286	1.65	1.77	2.06
483.8858	1.65	1.77	2.06
483.9428	1.67	1.79	2.05
484	1.66	1.79	2.05
484.0572	1.67	1.8	2.04
484.1142	1.69	1.8	2.03
484.1714	1.66	1.8	2.01
484.2286	1.66	1.81	2
484.2858	1.65	1.81	1.98
484.3428	1.66	1.81	1.98
484.4	1.68	1.81	1.98
484.4572	1.68	1.78	1.99
484.5142	1.67	1.74	1.98
484.5714	1.67	1.74	1.98
484.6286	1.68	1.74	2
484.6858	1.66	1.74	2.03
484.7428	1.69	1.71	2.04
484.8	1.71	1.69	2.06
484.8572	1.72	1.68	2.08
484.9142	1.72	1.66	2.08
484.9714	1.72	1.64	2.1
485.0286	1.7	1.65	2.12
485.0858	1.7	1.65	2.12
485.1428	1.72	1.62	2.14
485.2	1.72	1.61	2.14
485.2572	1.71	1.62	2.14
485.3142	1.68	1.64	2.11
485.3714	1.68	1.65	2.12
485.4286	1.67	1.67	2.13
485.4858	1.67	1.68	2.14
485.5428	1.66	1.7	2.14
485.6	1.65	1.72	2.13
485.6572	1.66	1.73	2.14
485.7142	1.64	1.76	2.13
485.7714	1.66	1.77	2.14
485.8286	1.7	1.73	2.15
485.8858	1.72	1.71	2.15
485.9428	1.72	1.71	2.16

486	1.73	1.72	2.14
486.0572	1.75	1.72	2.12
486.1142	1.75	1.73	2.12
486.1714	1.79	1.71	2.13
486.2286	1.79	1.71	2.15
486.2858	1.79	1.7	2.15
486.3428	1.78	1.7	2.14
486.4	1.74	1.72	2.13
486.4572	1.7	1.74	2.12
486.5142	1.72	1.74	2.11
486.5714	1.74	1.75	2.09
486.6286	1.72	1.75	2.08
486.6858	1.73	1.72	2.06
486.7428	1.71	1.72	2.03
486.8	1.71	1.7	2.01
486.8572	1.71	1.71	2.02
486.9142	1.71	1.73	2.02
486.9714	1.72	1.71	2.04
487.0286	1.75	1.68	2.04
487.0858	1.77	1.66	2.03
487.1428	1.76	1.64	2.03
487.2	1.75	1.65	2.01
487.2572	1.75	1.64	2
487.3142	1.76	1.63	1.99
487.3714	1.74	1.66	2
487.4286	1.76	1.62	1.99
487.4858	1.75	1.62	1.98
487.5428	1.75	1.64	2
487.6	1.76	1.67	2.01
487.6572	1.72	1.71	2.03
487.7142	1.72	1.72	2.03
487.7714	1.71	1.71	2.03
487.8286	1.73	1.73	2.04
487.8858	1.71	1.75	2.02
487.9428	1.72	1.73	2.01
488	1.72	1.76	2.02
488.0572	1.73	1.77	2
488.1142	1.69	1.79	2.01
488.1714	1.66	1.78	2.02
488.2286	1.68	1.75	2.03
488.2858	1.67	1.74	2.03
488.3428	1.67	1.75	2.03
488.4	1.68	1.72	2.04
488.4572	1.67	1.7	2
488.5142	1.66	1.72	1.98
488.5714	1.67	1.71	1.96
488.6286	1.66	1.69	1.94

488.6858	1.69	1.67	1.95
488.7428	1.72	1.66	1.93
488.8	1.71	1.65	1.94
488.8572	1.73	1.63	1.94
488.9142	1.7	1.65	1.93
488.9714	1.66	1.69	1.92
489.0286	1.66	1.71	1.93
489.0858	1.71	1.68	1.89
489.1428	1.7	1.69	1.88
489.2	1.69	1.71	1.87
489.2572	1.7	1.71	1.87
489.3142	1.7	1.72	1.89
489.3714	1.7	1.75	1.91
489.4286	1.69	1.78	1.93
489.4858	1.7	1.76	1.95
489.5428	1.71	1.78	1.97
489.6	1.72	1.78	1.95
489.6572	1.67	1.79	1.97
489.7142	1.66	1.81	1.96
489.7714	1.66	1.78	1.94
489.8286	1.65	1.79	1.95
489.8858	1.61	1.79	1.94
489.9428	1.63	1.76	1.95
490	1.61	1.74	1.95
490.0572	1.61	1.76	1.96
490.1142	1.63	1.74	1.97
490.1714	1.61	1.76	1.96
490.2286	1.6	1.76	1.97
490.2858	1.61	1.76	1.96
490.3428	1.62	1.77	1.97
490.4	1.62	1.78	1.94
490.4572	1.62	1.78	1.95
490.5142	1.61	1.8	1.96
490.5714	1.61	1.82	1.94
490.6286	1.62	1.81	1.96
490.6858	1.6	1.81	1.97
490.7428	1.61	1.79	1.97
490.8	1.64	1.79	1.98
490.8572	1.62	1.79	1.99
490.9142	1.62	1.75	1.99
490.9714	1.64	1.73	1.99
491.0286	1.63	1.7	1.99
491.0858	1.62	1.7	2
491.1428	1.63	1.67	2
491.2	1.64	1.68	2.02
491.2572	1.65	1.67	2.01
491.3142	1.66	1.67	2.02

491.3714	1.63	1.69	2
491.4286	1.65	1.67	2
491.4858	1.66	1.7	1.99
491.5428	1.65	1.72	1.99
491.6	1.64	1.73	2
491.6572	1.66	1.71	2.02
491.7142	1.66	1.73	2.02
491.7714	1.64	1.7	2.04
491.8286	1.62	1.71	2.04
491.8858	1.6	1.73	2.02
491.9428	1.6	1.7	2.02
492	1.59	1.69	2.03
492.0572	1.58	1.71	2.02
492.1142	1.58	1.68	2
492.1714	1.61	1.69	1.99
492.2286	1.6	1.7	1.99
492.2858	1.64	1.69	2.03
492.3428	1.64	1.7	2.04
492.4	1.65	1.69	2.07
492.4572	1.68	1.66	2.06
492.5142	1.7	1.65	2.05
492.5714	1.69	1.66	2.03
492.6286	1.69	1.66	2.04
492.6858	1.68	1.66	2.02
492.7428	1.67	1.66	2.05
492.8	1.66	1.66	2.08
492.8572	1.64	1.65	2.07
492.9142	1.62	1.66	2.05
492.9714	1.64	1.66	2.05
493.0286	1.63	1.66	2.07
493.0858	1.64	1.65	2.1
493.1428	1.67	1.63	2.11
493.2	1.67	1.63	2.08
493.2572	1.69	1.65	2.07
493.3142	1.66	1.68	2.08
493.3714	1.66	1.7	2.1
493.4286	1.66	1.73	2.11
493.4858	1.66	1.74	2.11
493.5428	1.64	1.74	2.09
493.6	1.68	1.77	2.06
493.6572	1.64	1.79	2.04
493.7142	1.66	1.8	2.02
493.7714	1.66	1.79	2.02
493.8286	1.66	1.76	2.04
493.8858	1.7	1.7	2.04
493.9428	1.72	1.67	2.03
494	1.7	1.66	1.99

494.0572	1.68	1.68	1.98
494.1142	1.68	1.68	2
494.1714	1.65	1.68	1.97
494.2286	1.66	1.69	1.96
494.2858	1.64	1.7	1.97
494.3428	1.66	1.69	1.97
494.4	1.64	1.71	1.96
494.4572	1.61	1.74	1.96
494.5142	1.59	1.73	1.95
494.5714	1.63	1.72	1.95
494.6286	1.68	1.67	1.94
494.6858	1.69	1.67	1.94
494.7428	1.69	1.67	1.94
494.8	1.7	1.67	1.95
494.8572	1.69	1.66	1.94
494.9142	1.68	1.7	1.95
494.9714	1.68	1.72	1.95
495.0286	1.69	1.71	1.94
495.0858	1.71	1.7	1.96
495.1428	1.71	1.7	1.96
495.2	1.71	1.7	1.94
495.2572	1.7	1.69	1.96
495.3142	1.7	1.67	2
495.3714	1.68	1.71	2.03
495.4286	1.7	1.7	2.05
495.4858	1.7	1.7	2.07
495.5428	1.71	1.67	2.08
495.6	1.69	1.67	2.05
495.6572	1.68	1.69	2.04
495.7142	1.68	1.69	2.03
495.7714	1.7	1.67	2.05
495.8286	1.69	1.68	2.08
495.8858	1.72	1.68	2.06
495.9428	1.72	1.63	2.06
496	1.69	1.66	2.04
496.0572	1.66	1.67	2.03
496.1142	1.66	1.68	2.02
496.1714	1.62	1.69	1.99
496.2286	1.63	1.69	1.94
496.2858	1.61	1.72	1.93
496.3428	1.57	1.76	1.91
496.4	1.56	1.76	1.89
496.4572	1.54	1.75	1.9
496.5142	1.5	1.78	1.92
496.5714	1.51	1.78	1.9
496.6286	1.53	1.76	1.89
496.6858	1.51	1.77	1.87

496.7428	1.57	1.75	1.85
496.8	1.56	1.75	1.84
496.8572	1.55	1.74	1.84
496.9142	1.57	1.75	1.84
496.9714	1.58	1.73	1.86
497.0286	1.58	1.76	1.87
497.0858	1.6	1.75	1.87
497.1428	1.62	1.76	1.9
497.2	1.64	1.77	1.92
497.2572	1.66	1.75	1.93
497.3142	1.68	1.75	1.93
497.3714	1.7	1.71	1.94
497.4286	1.72	1.69	1.94
497.4858	1.7	1.69	1.97
497.5428	1.68	1.71	1.97
497.6	1.68	1.71	1.98
497.6572	1.71	1.67	1.99
497.7142	1.73	1.65	2.02
497.7714	1.73	1.65	2.02
497.8286	1.71	1.66	2.03
497.8858	1.7	1.68	1.99
497.9428	1.66	1.71	2
498	1.66	1.7	1.99
498.0572	1.66	1.69	1.98
498.1142	1.67	1.68	1.96
498.1714	1.69	1.63	1.96
498.2286	1.66	1.67	1.97
498.2858	1.62	1.69	1.95
498.3428	1.63	1.65	1.96
498.4	1.62	1.64	1.94
498.4572	1.61	1.65	1.95
498.5142	1.63	1.64	1.96
498.5714	1.62	1.64	1.95
498.6286	1.58	1.64	1.95
498.6858	1.61	1.63	1.96
498.7428	1.59	1.64	1.96
498.8	1.6	1.65	1.98
498.8572	1.61	1.63	1.99
498.9142	1.57	1.66	2.01
498.9714	1.58	1.67	2.01
499.0286	1.59	1.65	2
499.0858	1.57	1.67	1.98
499.1428	1.53	1.67	1.98
499.2	1.52	1.69	1.98
499.2572	1.51	1.71	1.98
499.3142	1.51	1.7	1.99
499.3714	1.52	1.67	2.01

499.4286	1.51	1.69	2
499.4858	1.53	1.65	1.98
499.5428	1.54	1.63	1.98
499.6	1.52	1.65	1.97
499.6572	1.57	1.67	1.97
499.7142	1.58	1.68	1.95
499.7714	1.62	1.66	1.96
499.8286	1.62	1.66	1.95
499.8858	1.62	1.72	1.96
499.9428	1.59	1.74	1.99
500	1.63	1.7	1.98
500.0572	1.63	1.69	1.95
500.1142	1.62	1.71	1.97
500.1714	1.64	1.69	1.96
500.2286	1.59	1.68	1.95
500.2858	1.64	1.64	1.93
500.3428	1.63	1.64	1.94
500.4	1.61	1.67	1.94
500.4572	1.63	1.61	1.93
500.5142	1.68	1.58	1.93
500.5714	1.67	1.59	1.93
500.6286	1.66	1.62	1.94
500.6858	1.67	1.61	1.92
500.7428	1.68	1.62	1.91
500.8	1.71	1.63	1.91
500.8572	1.66	1.65	1.88
500.9142	1.66	1.61	1.9
500.9714	1.69	1.59	1.9
501.0286	1.69	1.6	1.91
501.0858	1.64	1.61	1.9
501.1428	1.61	1.62	1.88
501.2	1.6	1.61	1.87
501.2572	1.59	1.63	1.88
501.3142	1.56	1.64	1.9
501.3714	1.57	1.64	1.9
501.4286	1.56	1.66	1.9
501.4858	1.55	1.7	1.89
501.5428	1.55	1.72	1.9
501.6	1.55	1.73	1.88
501.6572	1.56	1.73	1.9
501.7142	1.58	1.74	1.91
501.7714	1.59	1.75	1.91
501.8286	1.61	1.71	1.91
501.8858	1.6	1.69	1.89
501.9428	1.59	1.7	1.9
502	1.6	1.67	1.92
502.0572	1.59	1.67	1.92

502.1142	1.59	1.63	1.93
502.1714	1.59	1.63	1.92
502.2286	1.58	1.63	1.94
502.2858	1.57	1.63	1.98
502.3428	1.58	1.6	1.97
502.4	1.55	1.63	1.94
502.4572	1.55	1.65	1.94
502.5142	1.55	1.63	1.95
502.5714	1.57	1.65	1.95
502.6286	1.58	1.66	1.97
502.6858	1.58	1.67	1.98
502.7428	1.58	1.67	1.99
502.8	1.56	1.68	2
502.8572	1.59	1.66	2.01
502.9142	1.58	1.69	2
502.9714	1.61	1.67	2.01
503.0286	1.64	1.66	2.02
503.0858	1.61	1.67	2.01
503.1428	1.58	1.67	1.98
503.2	1.54	1.7	1.96
503.2572	1.52	1.72	1.98
503.3142	1.52	1.71	2.02
503.3714	1.51	1.71	2.02
503.4286	1.49	1.7	2.03
503.4858	1.5	1.69	2.01
503.5428	1.5	1.68	1.99
503.6	1.48	1.69	1.99
503.6572	1.43	1.69	1.99
503.7142	1.45	1.7	1.98
503.7714	1.47	1.67	1.97
503.8286	1.52	1.65	1.94
503.8858	1.53	1.63	1.93
503.9428	1.55	1.64	1.92
504	1.55	1.6	1.91
504.0572	1.57	1.62	1.94
504.1142	1.56	1.62	1.94
504.1714	1.56	1.64	1.93
504.2286	1.58	1.64	1.94
504.2858	1.59	1.64	1.96
504.3428	1.59	1.65	1.96
504.4	1.58	1.65	1.94
504.4572	1.57	1.65	1.96
504.5142	1.58	1.65	1.97
504.5714	1.57	1.67	1.95
504.6286	1.57	1.67	1.95
504.6858	1.56	1.67	1.97
504.7428	1.58	1.66	1.97

504.8	1.59	1.64	1.95
504.8572	1.58	1.64	1.96
504.9142	1.58	1.63	1.95
504.9714	1.58	1.63	1.94
505.0286	1.6	1.63	1.93
505.0858	1.6	1.61	1.94
505.1428	1.64	1.62	1.93
505.2	1.65	1.62	1.91
505.2572	1.63	1.64	1.91
505.3142	1.62	1.64	1.92
505.3714	1.63	1.63	1.89
505.4286	1.62	1.6	1.88
505.4858	1.62	1.62	1.88
505.5428	1.61	1.6	1.9
505.6	1.59	1.59	1.89
505.6572	1.58	1.6	1.91
505.7142	1.56	1.61	1.93
505.7714	1.52	1.59	1.95
505.8286	1.55	1.57	1.98
505.8858	1.55	1.57	1.97
505.9428	1.52	1.58	1.97
506	1.54	1.6	1.96
506.0572	1.53	1.6	1.95
506.1142	1.52	1.63	1.95
506.1714	1.55	1.61	1.94
506.2286	1.58	1.61	1.93
506.2858	1.57	1.57	1.96
506.3428	1.58	1.55	1.96
506.4	1.57	1.53	1.97
506.4572	1.58	1.52	1.97
506.5142	1.61	1.5	1.99
506.5714	1.61	1.51	1.99
506.6286	1.64	1.49	1.99
506.6858	1.63	1.48	1.96
506.7428	1.61	1.48	1.94
506.8	1.6	1.47	1.94
506.8572	1.61	1.47	1.93
506.9142	1.6	1.49	1.93
506.9714	1.62	1.5	1.91
507.0286	1.64	1.5	1.92
507.0858	1.65	1.51	1.93
507.1428	1.62	1.51	1.95
507.2	1.61	1.55	1.92
507.2572	1.63	1.53	1.92
507.3142	1.65	1.51	1.93
507.3714	1.63	1.52	1.92
507.4286	1.65	1.53	1.9

507.4858	1.63	1.54	1.87
507.5428	1.64	1.56	1.87
507.6	1.61	1.56	1.88
507.6572	1.56	1.59	1.88
507.7142	1.58	1.58	1.87
507.7714	1.58	1.54	1.85
507.8286	1.55	1.57	1.84
507.8858	1.55	1.59	1.86
507.9428	1.53	1.6	1.83
508	1.49	1.61	1.85
508.0572	1.5	1.62	1.86
508.1142	1.49	1.65	1.87
508.1714	1.51	1.64	1.85
508.2286	1.53	1.63	1.84
508.2858	1.52	1.63	1.86
508.3428	1.53	1.65	1.86
508.4	1.54	1.63	1.87
508.4572	1.54	1.61	1.87
508.5142	1.55	1.61	1.88
508.5714	1.58	1.56	1.88
508.6286	1.59	1.56	1.88
508.6858	1.58	1.52	1.92
508.7428	1.56	1.52	1.93
508.8	1.57	1.49	1.92
508.8572	1.61	1.49	1.93
508.9142	1.6	1.51	1.92
508.9714	1.59	1.56	1.9
509.0286	1.57	1.59	1.91
509.0858	1.57	1.59	1.89
509.1428	1.54	1.63	1.89
509.2	1.54	1.63	1.88
509.2572	1.56	1.64	1.88
509.3142	1.58	1.63	1.88
509.3714	1.57	1.65	1.89
509.4286	1.53	1.64	1.86
509.4858	1.54	1.63	1.87
509.5428	1.54	1.61	1.88
509.6	1.59	1.6	1.88
509.6572	1.6	1.6	1.9
509.7142	1.62	1.6	1.9
509.7714	1.63	1.6	1.91
509.8286	1.6	1.63	1.92
509.8858	1.56	1.65	1.93
509.9428	1.56	1.66	1.93
510	1.56	1.66	1.92
510.0572	1.54	1.66	1.91
510.1142	1.53	1.65	1.91

510.1714	1.53	1.65	1.9
510.2286	1.52	1.65	1.89
510.2858	1.52	1.61	1.87
510.3428	1.51	1.63	1.89
510.4	1.53	1.62	1.89
510.4572	1.52	1.63	1.89
510.5142	1.53	1.62	1.88
510.5714	1.55	1.62	1.83
510.6286	1.55	1.63	1.83
510.6858	1.57	1.61	1.82
510.7428	1.56	1.59	1.78
510.8	1.57	1.6	1.78
510.8572	1.54	1.62	1.8
510.9142	1.54	1.61	1.81
510.9714	1.54	1.56	1.83
511.0286	1.58	1.57	1.86
511.0858	1.59	1.58	1.87
511.1428	1.57	1.57	1.86
511.2	1.59	1.56	1.86
511.2572	1.6	1.57	1.86
511.3142	1.59	1.61	1.87
511.3714	1.6	1.62	1.86
511.4286	1.57	1.63	1.85
511.4858	1.56	1.65	1.85
511.5428	1.57	1.7	1.84
511.6	1.55	1.7	1.87
511.6572	1.55	1.69	1.88
511.7142	1.54	1.69	1.85
511.7714	1.53	1.69	1.84
511.8286	1.52	1.7	1.85
511.8858	1.54	1.7	1.84
511.9428	1.5	1.68	1.83
512	1.53	1.65	1.84
512.0572	1.52	1.63	1.86
512.1142	1.51	1.61	1.87
512.1714	1.51	1.58	1.85
512.2286	1.49	1.58	1.85
512.2858	1.49	1.63	1.85
512.3428	1.48	1.67	1.87
512.4	1.49	1.66	1.89
512.4572	1.49	1.66	1.92
512.5142	1.51	1.65	1.9
512.5714	1.55	1.66	1.92
512.6286	1.57	1.68	1.92
512.6858	1.57	1.7	1.92
512.7428	1.6	1.69	1.92
512.8	1.59	1.68	1.92

512.8572	1.6	1.66	1.91
512.9142	1.61	1.65	1.89
512.9714	1.61	1.64	1.89
513.0286	1.58	1.64	1.88
513.0858	1.58	1.67	1.88
513.1428	1.56	1.65	1.89
513.2	1.52	1.63	1.88
513.2572	1.53	1.6	1.89
513.3142	1.54	1.61	1.9
513.3714	1.58	1.62	1.9
513.4286	1.58	1.59	1.89
513.4858	1.59	1.56	1.89
513.5428	1.59	1.56	1.88
513.6	1.62	1.54	1.86
513.6572	1.58	1.54	1.84
513.7142	1.55	1.58	1.83
513.7714	1.56	1.57	1.82
513.8286	1.55	1.58	1.84
513.8858	1.5	1.59	1.84
513.9428	1.45	1.58	1.85
514	1.43	1.62	1.85
514.0572	1.43	1.61	1.84
514.1142	1.43	1.63	1.83
514.1714	1.41	1.64	1.82
514.2286	1.46	1.62	1.81
514.2858	1.47	1.6	1.83
514.3428	1.48	1.61	1.84
514.4	1.46	1.62	1.84
514.4572	1.52	1.61	1.86
514.5142	1.54	1.6	1.87
514.5714	1.54	1.58	1.86
514.6286	1.52	1.6	1.88
514.6858	1.51	1.58	1.88
514.7428	1.51	1.57	1.88
514.8	1.49	1.55	1.88
514.8572	1.49	1.56	1.88
514.9142	1.48	1.55	1.88
514.9714	1.51	1.56	1.9
515.0286	1.49	1.55	1.91
515.0858	1.48	1.56	1.9
515.1428	1.49	1.55	1.86
515.2	1.54	1.54	1.82
515.2572	1.54	1.55	1.8
515.3142	1.53	1.56	1.79
515.3714	1.54	1.58	1.78
515.4286	1.58	1.57	1.79
515.4858	1.59	1.57	1.79

515.5428	1.56	1.56	1.8
515.6	1.55	1.58	1.8
515.6572	1.55	1.57	1.81
515.7142	1.55	1.57	1.81
515.7714	1.51	1.6	1.8
515.8286	1.54	1.56	1.79
515.8858	1.56	1.54	1.78
515.9428	1.56	1.55	1.78
516	1.52	1.55	1.77
516.0572	1.55	1.55	1.8
516.1142	1.56	1.56	1.83
516.1714	1.57	1.54	1.82
516.2286	1.56	1.53	1.82
516.2858	1.56	1.55	1.83
516.3428	1.56	1.55	1.82
516.4	1.57	1.57	1.82
516.4572	1.53	1.6	1.8
516.5142	1.54	1.59	1.81
516.5714	1.52	1.59	1.81
516.6286	1.51	1.57	1.83
516.6858	1.51	1.55	1.85
516.7428	1.53	1.56	1.85
516.8	1.54	1.57	1.85
516.8572	1.53	1.56	1.84
516.9142	1.55	1.55	1.85
516.9714	1.51	1.55	1.86
517.0286	1.51	1.55	1.87
517.0858	1.49	1.55	1.87
517.1428	1.47	1.58	1.89
517.2	1.48	1.56	1.88
517.2572	1.49	1.57	1.88
517.3142	1.48	1.55	1.9
517.3714	1.46	1.56	1.91
517.4286	1.47	1.58	1.89
517.4858	1.47	1.59	1.88
517.5428	1.48	1.6	1.87
517.6	1.5	1.59	1.87
517.6572	1.5	1.59	1.88
517.7142	1.54	1.56	1.87
517.7714	1.54	1.56	1.88
517.8286	1.52	1.54	1.88
517.8858	1.52	1.55	1.88
517.9428	1.52	1.54	1.86
518	1.51	1.54	1.87
518.0572	1.52	1.53	1.86
518.1142	1.5	1.53	1.86
518.1714	1.5	1.53	1.86

518.2286	1.5	1.52	1.86
518.2858	1.49	1.52	1.85
518.3428	1.47	1.56	1.85
518.4	1.48	1.59	1.86
518.4572	1.49	1.6	1.85
518.5142	1.46	1.61	1.84
518.5714	1.47	1.63	1.83
518.6286	1.45	1.62	1.83
518.6858	1.45	1.62	1.83
518.7428	1.43	1.64	1.8
518.8	1.44	1.62	1.8
518.8572	1.44	1.63	1.84
518.9142	1.46	1.61	1.83
518.9714	1.45	1.58	1.82
519.0286	1.45	1.58	1.83
519.0858	1.48	1.57	1.82
519.1428	1.49	1.54	1.82
519.2	1.51	1.54	1.82
519.2572	1.5	1.54	1.82
519.3142	1.51	1.55	1.81
519.3714	1.53	1.56	1.79
519.4286	1.56	1.56	1.79
519.4858	1.54	1.57	1.77
519.5428	1.54	1.62	1.76
519.6	1.53	1.63	1.78
519.6572	1.53	1.63	1.8
519.7142	1.53	1.65	1.8
519.7714	1.52	1.65	1.8
519.8286	1.5	1.66	1.79
519.8858	1.5	1.66	1.79
519.9428	1.48	1.65	1.81
520	1.48	1.66	1.84
520.0572	1.46	1.67	1.83
520.1142	1.47	1.64	1.83
520.1714	1.49	1.63	1.84
520.2286	1.47	1.64	1.85
520.2858	1.47	1.63	1.86
520.3428	1.48	1.62	1.87
520.4	1.52	1.61	1.9
520.4572	1.56	1.6	1.9
520.5142	1.57	1.61	1.89
520.5714	1.58	1.62	1.9
520.6286	1.6	1.56	1.88
520.6858	1.62	1.57	1.88
520.7428	1.61	1.57	1.89
520.8	1.63	1.57	1.89
520.8572	1.63	1.56	1.87

520.9142	1.63	1.53	1.85
520.9714	1.61	1.53	1.83
521.0286	1.6	1.51	1.82
521.0858	1.61	1.51	1.82
521.1428	1.61	1.51	1.81
521.2	1.59	1.56	1.81
521.2572	1.57	1.57	1.8
521.3142	1.58	1.56	1.77
521.3714	1.57	1.58	1.79
521.4286	1.56	1.59	1.79
521.4858	1.55	1.61	1.77
521.5428	1.54	1.63	1.78
521.6	1.54	1.63	1.77
521.6572	1.54	1.6	1.78
521.7142	1.56	1.57	1.74
521.7714	1.54	1.58	1.73
521.8286	1.54	1.58	1.76
521.8858	1.54	1.58	1.77
521.9428	1.58	1.58	1.76
522	1.58	1.6	1.77
522.0572	1.59	1.59	1.77
522.1142	1.58	1.58	1.76
522.1714	1.59	1.57	1.76
522.2286	1.56	1.59	1.79
522.2858	1.56	1.58	1.76
522.3428	1.58	1.58	1.74
522.4	1.57	1.58	1.77
522.4572	1.57	1.56	1.77
522.5142	1.52	1.58	1.77
522.5714	1.53	1.55	1.78
522.6286	1.53	1.55	1.81
522.6858	1.52	1.54	1.82
522.7428	1.51	1.56	1.82
522.8	1.5	1.57	1.83
522.8572	1.48	1.61	1.85
522.9142	1.45	1.61	1.82
522.9714	1.46	1.58	1.82
523.0286	1.44	1.59	1.83
523.0858	1.45	1.57	1.83
523.1428	1.43	1.6	1.81
523.2	1.41	1.58	1.81
523.2572	1.41	1.57	1.8
523.3142	1.4	1.56	1.77
523.3714	1.42	1.57	1.76
523.4286	1.4	1.55	1.76
523.4858	1.4	1.54	1.76
523.5428	1.4	1.55	1.74

523.6	1.39	1.56	1.74
523.6572	1.38	1.57	1.73
523.7142	1.4	1.54	1.73
523.7714	1.4	1.55	1.73
523.8286	1.43	1.55	1.75
523.8858	1.44	1.53	1.76
523.9428	1.44	1.51	1.79
524	1.49	1.48	1.79
524.0572	1.49	1.48	1.8
524.1142	1.47	1.5	1.82
524.1714	1.47	1.53	1.86
524.2286	1.48	1.53	1.86
524.2858	1.46	1.52	1.9
524.3428	1.47	1.52	1.92
524.4	1.45	1.54	1.91
524.4572	1.45	1.58	1.91
524.5142	1.46	1.58	1.9
524.5714	1.42	1.6	1.9
524.6286	1.43	1.6	1.88
524.6858	1.47	1.58	1.86
524.7428	1.49	1.55	1.86
524.8	1.5	1.54	1.85
524.8572	1.49	1.55	1.83
524.9142	1.48	1.56	1.83
524.9714	1.47	1.56	1.83
525.0286	1.48	1.51	1.81
525.0858	1.47	1.52	1.79
525.1428	1.46	1.53	1.77
525.2	1.44	1.53	1.75
525.2572	1.4	1.54	1.73
525.3142	1.4	1.55	1.73
525.3714	1.4	1.54	1.73
525.4286	1.39	1.56	1.73
525.4858	1.42	1.55	1.74
525.5428	1.45	1.54	1.75
525.6	1.43	1.55	1.76
525.6572	1.43	1.56	1.75
525.7142	1.43	1.57	1.78
525.7714	1.46	1.57	1.8
525.8286	1.48	1.55	1.81
525.8858	1.49	1.55	1.81
525.9428	1.47	1.56	1.81
526	1.5	1.56	1.81
526.0572	1.48	1.57	1.79
526.1142	1.47	1.58	1.81
526.1714	1.5	1.59	1.8
526.2286	1.49	1.58	1.8

526.2858	1.49	1.55	1.8
526.3428	1.49	1.55	1.8
526.4	1.47	1.58	1.8
526.4572	1.47	1.57	1.82
526.5142	1.47	1.58	1.84
526.5714	1.46	1.55	1.84
526.6286	1.45	1.57	1.81
526.6858	1.42	1.57	1.8
526.7428	1.41	1.56	1.8
526.8	1.39	1.59	1.81
526.8572	1.39	1.59	1.81
526.9142	1.38	1.58	1.8
526.9714	1.37	1.57	1.82
527.0286	1.36	1.56	1.81
527.0858	1.34	1.57	1.83
527.1428	1.37	1.58	1.83
527.2	1.39	1.57	1.85
527.2572	1.41	1.56	1.86
527.3142	1.38	1.56	1.83
527.3714	1.39	1.54	1.79
527.4286	1.42	1.54	1.77
527.4858	1.44	1.55	1.79
527.5428	1.44	1.54	1.8
527.6	1.43	1.54	1.81
527.6572	1.48	1.51	1.8
527.7142	1.47	1.5	1.8
527.7714	1.45	1.47	1.79
527.8286	1.44	1.49	1.79
527.8858	1.45	1.49	1.79
527.9428	1.45	1.53	1.79
528	1.44	1.53	1.78
528.0572	1.41	1.53	1.79
528.1142	1.41	1.53	1.77
528.1714	1.43	1.53	1.77
528.2286	1.4	1.55	1.79
528.2858	1.4	1.56	1.8
528.3428	1.4	1.56	1.8
528.4	1.45	1.51	1.78
528.4572	1.44	1.54	1.75
528.5142	1.43	1.51	1.74
528.5714	1.4	1.53	1.74
528.6286	1.39	1.54	1.74
528.6858	1.39	1.55	1.75
528.7428	1.39	1.55	1.75
528.8	1.41	1.55	1.73
528.8572	1.38	1.54	1.71
528.9142	1.4	1.55	1.71

528.9714	1.37	1.56	1.7
529.0286	1.36	1.52	1.71
529.0858	1.39	1.51	1.7
529.1428	1.44	1.48	1.7
529.2	1.48	1.46	1.68
529.2572	1.5	1.46	1.67
529.3142	1.5	1.44	1.67
529.3714	1.51	1.41	1.69
529.4286	1.52	1.41	1.7
529.4858	1.51	1.43	1.71
529.5428	1.5	1.45	1.71
529.6	1.49	1.47	1.71
529.6572	1.49	1.49	1.72
529.7142	1.46	1.51	1.74
529.7714	1.45	1.52	1.73
529.8286	1.45	1.5	1.73
529.8858	1.44	1.54	1.72
529.9428	1.42	1.55	1.72
530	1.41	1.57	1.75
530.0572	1.41	1.55	1.76
530.1142	1.42	1.54	1.79
530.1714	1.44	1.52	1.79
530.2286	1.45	1.5	1.79
530.2858	1.47	1.47	1.77
530.3428	1.48	1.45	1.76
530.4	1.46	1.5	1.73
530.4572	1.46	1.45	1.73
530.5142	1.45	1.46	1.75
530.5714	1.45	1.46	1.73
530.6286	1.49	1.45	1.75
530.6858	1.5	1.43	1.74
530.7428	1.48	1.46	1.74
530.8	1.47	1.47	1.74
530.8572	1.49	1.48	1.73
530.9142	1.44	1.5	1.7
530.9714	1.44	1.48	1.69
531.0286	1.41	1.53	1.67
531.0858	1.44	1.53	1.68
531.1428	1.45	1.54	1.7
531.2	1.42	1.57	1.7
531.2572	1.42	1.6	1.7
531.3142	1.41	1.6	1.73
531.3714	1.41	1.6	1.73
531.4286	1.38	1.61	1.72
531.4858	1.41	1.6	1.74
531.5428	1.44	1.58	1.73
531.6	1.47	1.57	1.73

531.6572	1.45	1.57	1.72
531.7142	1.49	1.54	1.75
531.7714	1.49	1.52	1.74
531.8286	1.51	1.5	1.76
531.8858	1.53	1.46	1.78
531.9428	1.54	1.48	1.78
532	1.51	1.49	1.77
532.0572	1.49	1.49	1.76
532.1142	1.46	1.5	1.77
532.1714	1.44	1.49	1.78
532.2286	1.44	1.49	1.76
532.2858	1.39	1.51	1.75
532.3428	1.36	1.5	1.76
532.4	1.33	1.5	1.74
532.4572	1.32	1.53	1.74
532.5142	1.32	1.53	1.74
532.5714	1.35	1.53	1.75
532.6286	1.36	1.53	1.75
532.6858	1.38	1.56	1.74
532.7428	1.39	1.54	1.72
532.8	1.39	1.53	1.73
532.8572	1.41	1.52	1.74
532.9142	1.45	1.52	1.73
532.9714	1.47	1.54	1.71
533.0286	1.48	1.52	1.71
533.0858	1.45	1.5	1.72
533.1428	1.46	1.48	1.72
533.2	1.48	1.46	1.71
533.2572	1.48	1.42	1.72
533.3142	1.51	1.41	1.74
533.3714	1.49	1.41	1.73
533.4286	1.49	1.42	1.73
533.4858	1.45	1.41	1.72
533.5428	1.44	1.41	1.72
533.6	1.43	1.41	1.71
533.6572	1.42	1.42	1.69
533.7142	1.4	1.43	1.67
533.7714	1.39	1.45	1.68
533.8286	1.4	1.47	1.66
533.8858	1.4	1.47	1.67
533.9428	1.4	1.5	1.65
534	1.41	1.49	1.64
534.0572	1.39	1.51	1.64
534.1142	1.37	1.53	1.63
534.1714	1.39	1.53	1.6
534.2286	1.4	1.52	1.59
534.2858	1.41	1.52	1.6

534.3428	1.39	1.51	1.6
534.4	1.36	1.5	1.61
534.4572	1.32	1.52	1.6
534.5142	1.34	1.53	1.62
534.5714	1.32	1.52	1.63
534.6286	1.36	1.51	1.63
534.6858	1.37	1.47	1.65
534.7428	1.37	1.48	1.66
534.8	1.32	1.52	1.64
534.8572	1.29	1.53	1.64
534.8572	1.3	1.53	1.64
534.9334	1.33	1.53	1.64
534.9824	1.35	1.54	1.65
535.0313	1.33	1.51	1.66
535.0803	1.32	1.54	1.66
535.1293	1.31	1.55	1.68
535.1783	1.32	1.56	1.66
535.2273	1.31	1.57	1.65
535.2763	1.32	1.55	1.64
535.3252	1.36	1.54	1.63
535.3742	1.39	1.52	1.63
535.4232	1.39	1.5	1.62
535.4722	1.39	1.49	1.62
535.5212	1.39	1.48	1.63
535.5702	1.38	1.48	1.63
535.6191	1.39	1.48	1.65
535.6681	1.39	1.5	1.65
535.7171	1.37	1.5	1.65
535.7661	1.36	1.51	1.63
535.8151	1.35	1.49	1.64
535.864	1.37	1.5	1.64
535.913	1.36	1.53	1.63
535.962	1.37	1.54	1.64
536.011	1.39	1.54	1.64
536.06	1.39	1.53	1.65
536.109	1.39	1.5	1.65
536.1579	1.4	1.47	1.65
536.2069	1.41	1.44	1.64
536.2559	1.43	1.44	1.62
536.3049	1.42	1.45	1.63
536.3539	1.4	1.44	1.65

Figure 4B raw data

60 Packets	100 Packets
17.82	21.17
23.39	26.25
20.27	23.24

Figure 4C rawa data

60 Packets	100 Packets
40.26660156	336.5267177
35.04949533	364.3704517
73.00559376	781.4980228
10.75720713	100.7483391
43.48501	574.0698
217.767	3085.581
