# Supplementary material for: Understanding the aliya pulsed electric field dose-response relationship: Implications for ablation size, thermal load, and immune response in an orthotopic murine breast cancer model
Source: PLoS One. 2025 Feb 13;20(2):e0318440. doi: 10.1371/journal.pone.0318440 (PMC11824980; doi:10.1371/journal.pone.0318440)
Supplement: S1 Raw data — (ZIP) [file pone.0318440.s005.zip › Fig 5 raw data.pdf]

Figure 5A raw data

Days Post Treatment					Sham (aPD-1)			
-2	62.96	54.01	40.75	40.6	67.01	128.01	97.75	98.5
0	149.91	179.82	85.29	99.59	105.3	139.08	186.53	146.51
3	164.68	158.44	143.94	114.03	189.73	251.42	198.38	186.28
5	284.97	264.43	231.28	233.08	405.2	225.67	267.62	326.26
7	488.75	282.35	316.24	373.76	637.55	375.2	462.23	518.01
11	835.58	490.89	460.89	556.35	956.27	569.71	716.85	646.31
14	1263.41	494.74	578.73	837.62	1351.94	788.77	1433.21	1091.69
17	1636.73	761.48	876.61	1080.41	1666.26	861.42	2020.39	1602.91
19	2195	1241.9	994.6	1572.3	2302	1569.6	2324.5	2543.8

Days Post Treatment					Sham (IgG)		
-2	64.13	66.71	75.37	47.72	88.4	125.06	102.6
0	106.73	205.94	171.21	79.58	269.22	202.3	127.67
3	174.83	200.84	219.87	84.52	302.48	248.98	208.53
5	305.46	451.75	361.97	208.04	387.59	427.25	321.75
7	547.39	725.01	585.05	440.64	668.43	665.29	672.88
11	738.55	1035.8	670.97	548.35	1102.31	728.09	722.05
14	1213.1	1299	764.59	954.77	1442.03	1256.58	1020.7
17	1625.3	2118.65	1268.66	1137.6	2234.18	2004.57	1685.83
19	2015.8	3045	2100.4	1625.4	2336.3	2261.3	2180.3

Days Post Treatment					PEF 100 packets					
	83.32	71.21	62.61	88.18	72.44	52.81	75.15	80.61	66.65	85.96
	98	166.86	117.42	210.3	125.61	105.85	122.95	168.91	111.36	182.68
	114.2	123.43	116.51	155.56	185.48	172.54	120.96	144.69	140.43	192.48
	0	0	0	0	0	0	0	0	0	0
	0	0	0	0	0	0	0	0	0	0
	0	0	0	0	0	0	0	0	0	0
	0	137.56	0	0	0	0	0	0	0	0
	0	271.98	0	0	0	0	0	0	0	0
	0	425.1	0	0	0	0	0	0	0	0

Days Post Treatment					PEF 100 packets + aPD-1					
		53.32	100.34	63.02	109.55		67.8	70.54	98.96	83.03
		68.6	154.36	181.05	197.72		202.7	178.07	129.08	184
		134.19	172.24	221.78	202.69		118.06	79.52	161.47	131.6
		0	0	0	0		0	0	0	0
		0	0	0	0		0	0	0	0
		0	0	0	0		0	0	0	0
		0	0	0	0		0	0	0	0
		0	0	0	0		0	0	0	0
		0	0	0	0		0	0	0	0

Days Post Treatment					PEF 60 packets					
	55.76	112.54	103.45	104.36	84.73	99.43	82.38	87.81	107.65	102.96
	185.2	146.11	148.94	195.7	180.71	138.92	126	257.4	194.04	220.89
	112.1	149.82	109.66	158.23	88.69	110.06	115.91	141.31	179.51	154.4
	0	0	0	0	0	0	0	0	0	0
	0	0	0	0	0	0	0	0	0	0
	0	0	0	0	0	0	0	0	154.48	0
	0	0	0	0	0	0	0	160.44	520.25	0
	0	0	0	0	87.42	0	0	359.15	765.17	0

	0	0	0	0	294.8	0	0	499.4	1092.6	0

Days Post Treatment					PEF 60 packets+ aPD-1					
	93.86	76.19	91.99	98.86	85.67	143.08	94.96	75.91	98.47	77.16
	176.28	119.38	170.11	155.24	142.88	99.76	139.99	119.59	183.41	133.1
	166.65	144.48	245.98	221.72	218.85	108.81	110.01	140.68	208	259.25
	0	0	0	0	0	0	0	0	0	0
	0	0	0	0	0	0	0	0	0	0
	0	0	0	0	0	0	0	0	0	0
	0	0	0	0	0	0	0	0	0	0
	0	0	0	0	0	0	0	0	0	0
	0	0	0	0	0	0	0	0	0	0

Figure 5B raw data

	Days post treatment	Sham (aPD-1)	Sham (IgG)	PEF 100 packets	100 packets+ a	PEF 60 packets	PEF 60 packets + aPD-1
c1m1 (day 19)	19	1					
c1m2 (day 24)	24	1					
c1m3 (day 26)	26	1					
c1m4 (day 24)	24	1					
c1m5 (day 19)	19	1					
c11m1 (day 21)	21	1					
c11m2 (day 19)	19	1					
c11m3 (day 19)	19	1					
c2m1 (day 19)	19		1				
c2m2 (day 19)	19		1				
c2m3 (day 19)	19		1				
c2m4 (day 24)	24		1				
c2m5 (day 19)	19		1				
c11m4 (day 19)	19		1				
c11m5 (day 19)	19		1				
c3m1	70			0			
c3m2 (day 28)	28			1			
c3m3	70			0			
c3m4 (day 31)	31			1			
c3m5 (day 31)	31			1			
c4m1 (day 33)	33			1			
c4m2	70			0			
c4m3 (day 35)	35			1			
c4m4 (day 28)	28			1			
c4m5	70			0			
c5m2	70				0		
c5m3	70				0		
c5m4	70				0		
c5m5	70				0		

c6m2 (day 38)	38	1		
c6m3	70	0		
c6m4	70	0		
c6m5	70	0		
c7m1 (day 33)	33		1	
c7m2 (day 33)	33		1	
c7m3 (day 31)	31		1	
c7m4	70		0	
c7m5 (day 33)	33		1	
c8m1	70		0	
c8m2 (day 35)	35		1	
c8m3 (day 28)	28		1	
c8m4 (day 24)	24		1	
c8m5	70		0	
c9m1 (day 35)	35			1
c9m2	70			0
c9m3	70			0
c9m4	70			0
c9m5	70			0
c10m1	70			0
c10m2	70			0
c10m3 (day 38)	38			1
c10m4	70			0
c10m5	70			0
