# Supplementary material for: Understanding the aliya pulsed electric field dose-response relationship: Implications for ablation size, thermal load, and immune response in an orthotopic murine breast cancer model
Source: PLoS One. 2025 Feb 13;20(2):e0318440. doi: 10.1371/journal.pone.0318440 (PMC11824980; doi:10.1371/journal.pone.0318440)
Supplement: S1 Raw data — (ZIP) [file pone.0318440.s005.zip › Fig 7 raw data.pdf]

Figure 7 raw data

	PEF 100 packets	PEF 60 packets
Eotaxin	0.991439272	1.0058855
G-CSF	0.767603191	0.72147069
GM-CSF	0.500560748	0.687383178
IFNγ	0.232412573	0.220574228
IL-1α	1.742322097	1.43670412
IL-1β	0.752350427	0.740598291
IL-2	0.273284314	0.330085784
IL-3	0.774344935	0.5078
IL-4	0.476926474	0.666813866
IL-5	0.508785816	0.747665031
IL-6	0.736818884	0.999558791
IL-7	0.185	0.810753599
IL-9	0.589384136	0.750419363
IL-10	0.35622904	0.388398957
IL-12p40	0.480183357	0.29471086
IL-12p70	0.613821138	1.218350755
IL-13	0.53235397	0.504022385
IL-15	1.12490743	1.218958282
IL-17	0.568269231	0.591
IP-10	0.752811621	0.776476101
KC	0.41965916	0.493761412
LIF	0.500392773	0.572924849
LIX	1.70746634	1.929620563
M-CSF	0.769191507	0.767660269
MCP-1	0.833753726	1.347855996
MIG	0.837317518	0.851733577
MIP-1α	0.416535185	0.470495424
MIP-1β	0.826006307	0.888332406
MIP-2	0.860201511	0.863979849
RANTES	0.923778851	1.220611916
TNFα	0.904840575	1.232116306
VEGF	0.691030755	0.748938361
