# Supplementary material for: Understanding the aliya pulsed electric field dose-response relationship: Implications for ablation size, thermal load, and immune response in an orthotopic murine breast cancer model
Source: PLoS One. 2025 Feb 13;20(2):e0318440. doi: 10.1371/journal.pone.0318440 (PMC11824980; doi:10.1371/journal.pone.0318440)
Supplement: S1 Raw data — (ZIP) [file pone.0318440.s005.zip › Fig 8 raw data.pdf]

Figure 8 raw data

PEF 100 packets VS Sham(IgG)	Overlap	PEF 60 packets VS Sham(IgG)
•IL-1β	•IFNγ	•IL-12p40
•IL-5	•IL-2	
•IL-12p70	•IL-13	
•VEGF	•IP-10	
•GM-CSF	•KC	
•M-CSF	•LIX	
•IL-4	•MIP-1α	
	•MIP-2	
