# Supplementary material for: Understanding the aliya pulsed electric field dose-response relationship: Implications for ablation size, thermal load, and immune response in an orthotopic murine breast cancer model
Source: PLoS One. 2025 Feb 13;20(2):e0318440. doi: 10.1371/journal.pone.0318440 (PMC11824980; doi:10.1371/journal.pone.0318440)
Supplement: S1 Raw data — (ZIP) [file pone.0318440.s005.zip › Fig 9 raw data.pdf]

Fig 9A raw data

Sham(aPD-1)

CD45(%)	98.31	96.56	97.26	98.27	98.5	97.94	96.47	97.81

Sham (IgG)

CD45(%)	98.84	99.28	98.3	98.71	98.01	96.31	96.21

PEF (100P)

CD45(%)	98.26	98.33	98.82	98.42	98.32	98.69	98.18	89.41	97.61	97.22

PEF (100P) + aPD-1

CD45(%)	95.89	96.63	86.03	96.66	97.16	96.2	82.66	97.46

PEF (60P)

CD45(%)	97.36	97.17	83.65	91.58	95.13	98.49	94.52	96.22	98.12	95.86

CD45(%) PEF (60P) +aPD-1

	1.74	93.34	95.46	96.91	93.31	97.29	97.76	95.98	96.82	96.46

Fig 9B raw data

Sham(aPD-1)

NK cells(%	0.23	0.81	0.88	0.58	0.4	0.87	0.78	0.9		

Sham (IgG)

NK cells(%	0.32	0.34	0.35	0.34	0.38	0.51	0.53			

PEF (100P)

NK cells(%	0.76	0.5	0.88	0.88	0.91	0.95	0.86	1.2	0.82	0.98

PEF (100P)

NK cells(%	0.76	0.5	0.88	0.88	0.91	0.95	0.86	1.2	0.82	0.98

PEF (100P) + aPD-1

NK cells(%	1.31	2.03	1.79	2.72	1.36	1.18	1.09	2.65		

PEF (60P)

NK cells(%	1.08	0.97		0.62	0	0.62	0	1.08	0.75	

PEF (60P) +aPD-1

NK cells(%)			2.41	1.38	1.14	0.96	0.87	1.56	0.43	0.62

Figure 9C raw data

					Sham(aPD-1)					
B cell(%)	1.46	5.64	4.81	3.51	1.85	3.5	3.56	3.33		
					Sham (IgG)					
B cell(%)	2.25	1.61	2.14	2.51	1.65	2.69	1.86			
					PEF (100P)					
B cell(%)	4.87	5.11	5.44	3.82	3.53	3.79	5.98	4.74	5.47	5.52
					PEF (100P) + aPD-1					
B cell(%)	4.53	6.9	3.96	5.55	7.26	5.41	8.09	5.38		
					PEF (60P)					
B cell(%)	8.06	7.55	9.15	10.07	4.5	4.38	2.23	7.5	2.08	4.58
					PEF (60P) +aPD-1					
B cell(%)	12.62	6.29	8.17	6.27	8.83	4.85	6.9	5.96	8.68	7.74
