# Supplementary material for: Understanding the aliya pulsed electric field dose-response relationship: Implications for ablation size, thermal load, and immune response in an orthotopic murine breast cancer model
Source: PLoS One. 2025 Feb 13;20(2):e0318440. doi: 10.1371/journal.pone.0318440 (PMC11824980; doi:10.1371/journal.pone.0318440)
Supplement: S1 Raw data — (ZIP) [file pone.0318440.s005.zip › Fig 10 raw data.pdf]

Figure 10A raw data

Sham(aPD-1)

CD3+(%)	70.7	72.43	73	74.56	70.3	65.11	67.56	61.55

Sham (IgG)

CD3+(%)	65.74	69.05	65.3	66.57	68.78	61.47	62.72

PEF (100P)

CD3+(%)	83.15	86.19	72.2	84.06	86.78	83.06	80.85	71.48	70.77	76.28

PEF (100P) + aPD-1

CD3+(%)	83.91	79.09	82.02	80.23	74.6	83.15	70.67	78.66

PEF (60P)

CD3+(%)	80.59	80.68	83.7	76.91	82.16	81.21	80.96	76.46	90.15	77.61

PEF (60P) +aPD-1

CD3+(%)		71.98	81.8	71.98	74.23	78	75.78	76.54	74.01	70.55

Figure 10B raw data

Sham(aPD-1)

%CD4	1.46	5.64	4.81	3.51	1.85	3.5	3.56	3.33

Sham (IgG)

%CD4	2.25	1.61	2.14	2.51	1.65	2.69	1.86

PEF (100P)

%CD4	4.87	5.11	5.44	3.82	3.53	3.79	5.98	4.74	5.47	5.52

PEF (100P) + aPD-1

%CD4	4.53	6.9	3.96	5.55	7.26	5.41	8.09	5.38

PEF (60P)

%CD4	8.06	7.55	9.15	10.07	4.5	4.38	2.23	7.5	2.08	4.58

PEF (60P) +aPD-1

%CD4	12.62	6.29	8.17	6.27	8.83	4.85	6.9	5.96	8.68	7.74

Figure 10C raw data

**CD4**					CM					
Sham(aPD-1)	15.85	6.55	6.48	4.09	6.69	9.65	7.36	6.27		
Sham (IgG)	7.07	9.87	6.41	2.14	2.49	5.38	10.09			
PEF (100P)	8.25	5.2	6.01	4.09	3.02	3.83	3.14	3.91	1.88	4.18
PEF (100P) + aPD-1	6.76	3.93	7.93	6.26	4.35	2.51	3.4	4.2		
PEF (60P)	4.35	4.52	7.01	2.47	3.61	4.53	9.64	5.71	4.82	7.76
PEF (60P) +aPD-1	0	4.84	3.44	4.45	2.89	4.69	4.16	5.27	5.47	4.31
					NAIVE					
Sham(aPD-1)	0.57	3.15	1.62	0.62	0.76	1.67	0.85	2.87		
Sham (IgG)	0.53	0.92	0.4	0.13	0.4	0.53	2.21			
PEF (100P)	0.08	0.14	0.11	0	0	0.05	0.12	0.13	0	0
PEF (100P) + aPD-1	0.04	0.08	0.05	0.03	0.19	0.03	0.05	0.03		
PEF (60P)	0.09	0.05	0.66	0.02	0.09	0	0	0.56	0	0.81
PEF (60P) +aPD-1	0	0.2	0.05	0.13	0.3	0.13	0.39	0.32	0.43	0.6
					EM					
Sham(aPD-1)	31.51	20.28	26.95	22.21	32.67	41.29	65.6	31.69		
Sham (IgG)	24.98	36.38	30.85	27.92	33.9	26.91	33.62			
PEF (100P)	76.2	79.02	76.34	84.9	76.85	79.22	73.71	82.76	72.14	83
PEF (100P) + aPD-1	80.73	71.87	80.74	71.62	74	72.93	84.04	71.45		
PEF (60P)	78.72	70.54	70.38	75.4	76.64	80.4	80.08	71.4	83.06	70.26
PEF (60P) +aPD-1	71.54	77.52	78.51	74.77	72.9	79.06	79.56	75.65	75.44	79.6
					DN					
Sham(aPD-1)	52.08	70.03	64.95	73.09	59.88	47.38	26.19	59.17		
Sham (IgG)	67.42	52.83	62.34	69.81	63.22	67.18	54.08			
PEF (100P)	15.47	15.64	17.54	11.01	20.13	16.91	23.03	13.21	25.98	12.82
PEF (100P) + aPD-1	12.47	24.12	11.29	22.09	21.46	24.52	12.51	24.31		
PEF (60P)	16.84	24.89	21.95	22.1	19.66	15.08	10.28	22.32	12.12	21.17

PEF (60P) +aPD-1	28.46	17.45	18.01	20.65	23.91	16.12	15.9	18.76	18.66	15.49

Figure 10D raw data

Sham(aPD-1)

%CD8	0.47	2.45	2.38	1.33	1.11	1.92	2.76	1.89		

Sham (IgG)

%CD8	0.99	0.78	0.95	0.91	0.61	1.16	0.96			

PEF (100P)

%CD8	3.14	2.26	2.98	1.84	1.54	1.95	3.47	2.3	2.59	2.56

PEF (100P) + aPD-1

%CD8	3.3	4.59	2.98	3.52	4.09	3.81	3.7	2.72		

PEF (60P)

%CD8	3.88	4.47	6.37	6.76	3.8	3.16	1.46	4.31	1.25	3.67

PEF (60P) +aPD-1

%CD8	5.74	4.24	3.81	2.99	5.39	3.76	4.26	3.24	5.15	3.69

Figure 10E raw data

CD8					CM					
Sham(aPD-1)	11.11	14.74	12.65	8.14	19.24	13.22	26.48	11.72		
Sham (IgG)	8.93	13.4	10.67	4.07	4.27	6.66	12.55			
PEF (100P)	10.14	19.48	21.4	11.07	8.39	8.05	9.98	11.95	6.11	6.82
PEF (100P) + aPD-1	8.11	7.38	11.61	10.43	12.03	9.11	4.26	9.35		
PEF (60P)	6	9.91	13.04	5.8	13.3	12.16	21.26	14.08	7.04	20.25
PEF (60P) +aPD-1	0	11.9	5.22	10.48	7.39	14.86	11.98	9.11	6.69	6.57
					NAIVE					
Sham(aPD-1)	5.26	3.92	2.08	2.13	0	1.93	1.38	7.12		
Sham (IgG)	0.69	4.04	1.27	0.55	0.27	0.62	3.37			
PEF (100P)	0.12	0.4	0.59	0.1	0.11	0.36	0.16	0.45	0.28	0.08
PEF (100P) + aPD-1	0.16	0.2	0	0.1	0.43	0.28	0	0.14		
PEF (60P)	0.1	0.08	0.13	0.06	0.16	0.11	0	0.31	0.13	0.25
PEF (60P) +aPD-1	0	0.59	0.54	0.75	0.34	0.33	0.76	0.33	0.68	0.22
					EM					
Sham(aPD-1)	26.9	33.04	37.5	27.53	57.47	40.08	54.69	24.65		
Sham (IgG)	31.79	21.7	36.17	26.25	42.13	39.01	31.27			
PEF (100P)	64.86	52.23	49.01	66.89	64.17	64.34	63.06	64.52	52.77	67.13
PEF (100P) + aPD-1	64.73	57.61	67.09	59.3	55.85	60.09	60.01	54.71		
PEF (60P)	48.02	55.51	60.82	61.51	58.24	66.11	64.52	58.95	78.27	55.35
PEF (60P) +aPD-1	55.36	68.29	56.75	65.03	65.09	66.32	68.9	62.91	66.68	69.87
					DN					
Sham(aPD-1)	56.73	48.29	47.77	62.2	23.29	44.76	17.45	56.51		
Sham (IgG)	58.59	60.85	51.9	69.13	53.33	53.72	52.81			
PEF (100P)	24.88	27.9	29	21.94	27.32	27.24	26.8	23.08	40.84	25.97
PEF (100P) + aPD-1	27	34.8	21.3	30.17	31.69	30.52	35.73	35.81		
PEF (60P)	45.88	34.5	26.01	32.63	28.31	21.62	14.22	26.65	14.57	24.15
PEF (60P) +aPD-1	44.64	19.22	37.49	23.74	27.18	18.49	18.36	27.65	25.94	23.34
